# Association between viral infections and glioma risk: a two-sample bidirectional Mendelian randomization analysis

**DOI:** 10.1186/s12916-023-03142-9

**Published:** 2023-12-05

**Authors:** Sheng Zhong, Wenzhuo Yang, Zhiyun Zhang, Yangyiran Xie, Lin Pan, Jiaxin Ren, Fei Ren, Yifan Li, Haoqun Xie, Hongyu Chen, Davy Deng, Jie Lu, Hui Li, Bo Wu, Youqi Chen, Fei Peng, Vinay K. Puduvalli, Ke Sai, Yunqian Li, Ye Cheng, Yonggao Mou

**Affiliations:** 1 State Key Laboratory of Oncology in South China, Guangdong Provincial Clinical Research Center for Cancer, Sun Yat-Sen University Cancer Center, Guangzhou, 510060 People’s Republic of China; 2 Department of Plastic Surgery, The First Hospital of Jilin University, Changchun, 130000 People’s Republic of China; 3 Vanderbilt University School of Medicine, Vanderbilt University, 1161 21St Ave S # D3300, Nashville, TN 37232 USA; 4 Clinical College, Jilin University, Street Xinmin 828, Changchun, People’s Republic of China; 5 Stroke Center, Department of Neurology, The First Hospital of Jilin University, Chang Chun, People’s Republic of China; 6 Dana Farber Cancer Institute, 450 Brookline Ave, Boston, MA 02215 USA; 7 Department of Orthopaedics, The First Hospital of Jilin University, No.71, Street Xinmin Road, Chaoyang District, Changchun, Jilin People’s Republic of China; 8 Department of Medicine, Division of Endocrinology, Diabetes and Metabolism, Baylor College of Medicine, Houston, TX USA; 9 Department of Neuro-Oncology, The University of Texas MD Anderson Cancer Center, Houston, TX USA; 10 Department of Neurosurgery, The First Hospital of Jilin University, Changchun, People’s Republic of China; 11 Department of Neurosurgery, Xuanwu Hospital, Capital Medical University, Beijing, People’s Republic of China

**Keywords:** Mendelian randomization, Viral infection, Risk, Glioma

## Abstract

**Background:**

Glioma is one of the leading types of brain tumor, but few etiologic factors of primary glioma have been identified. Previous observational research has shown an association between viral infection and glioma risk. In this study, we used Mendelian randomization (MR) analysis to explore the direction and magnitude of the causal relationship between viral infection and glioma.

**Methods:**

We conducted a two-sample bidirectional MR analysis using genome-wide association study (GWAS) data. Summary statistics data of glioma were collected from the largest meta-analysis GWAS, involving 12,488 cases and 18,169 controls. Single-nucleotide polymorphisms (SNPs) associated with exposures were used as instrumental variables to estimate the causal relationship between glioma and twelve types of viral infections from corresponding GWAS data. In addition, sensitivity analyses were performed.

**Results:**

After correcting for multiple tests and sensitivity analysis, we detected that genetically predicted herpes zoster (caused by Varicella zoster virus (VZV) infection) significantly decreased risk of low-grade glioma (LGG) development (OR = 0.85, 95% CI: 0.76–0.96, *P* = 0.01, FDR = 0.04). No causal effects of the other eleven viral infections on glioma and reverse causality were detected.

**Conclusions:**

This is one of the first and largest studies in this field. We show robust evidence supporting that genetically predicted herpes zoster caused by VZV infection reduces risk of LGG. The findings of our research advance understanding of the etiology of glioma.

**Supplementary Information:**

The online version contains supplementary material available at 10.1186/s12916-023-03142-9.

## Background

Glioma is a prevalent neurological tumor with an annual incidence of 5.6/100,000 in adults [1]. It comprises the most common malignant central nervous system (CNS) tumors in adults, including lower-grade gliomas (LGG, WHO grade I–III) and glioblastomas (GBM, WHO grade IV) [2]. Among these, GBM is the most aggressive subtype, accounting for 49% of all primary malignant brain tumors [3]. Prognosis of GBM is notoriously poor, with a 5-year survival rate of less than 5%. It causes much stress and pain for patients and their families [4]. Mean annual expenses for patients are €20,587.53; mean annual costs for caregivers are €5,581.49 [5, 6]. Identifying the cause of glioma allows for possible preventative measures to reduce the incidence of GBM and for targeted routine screening in highly susceptible individuals. Many previous studies have focused on finding the cause of glioma but failed to identify a clear etiology of gliomagenesis [7]. Several recent studies have consistently provided robust evidence that exposure to moderate or high levels of ionizing radiation is one of the environmental factors associated with glioma risk, even though this factor accounts for only a small fraction of cases [8, 9].

Prior viral infection is a risk factor for several cancers, including nasopharyngeal carcinoma, hepatic carcinoma, and cervical cancer [10]. In past decades, it was thought that viral infection is a potential etiological factor for gliomagenesis [10, 11]. Nevertheless, the relationship between viral infection and glioma incidence has remained unclear [12, 13], and the causal relationship between infection by various viruses and brain tumor development has only slowly been revealed [14, 15]. Several viruses (including herpes simplex virus (HSV), measles virus (MeV), and human cytomegalovirus (HCMV)) have been discovered in human glioma tissue and proven to cause brain tumors in animal models [14]. For many years, HCMV infection was suspected to be associated with gliomagenesis[15]. Indeed, a meta-analysis indicated that prior HCMV infection was significantly associated with increased glioma incidence (OR = 3, 95% CI:1.7–5.3) [16]. Furthermore, recent research indicates that among herpesviruses, apart from CMV, the virus most consistently associated with the risk of glioblastoma is varicella-zoster virus (VZV). VZV, a neurotropic alpha-herpesvirus, capable of causing chickenpox and herpes zoster, has been linked to approximately a 30% reduced risk of glioblastoma in individuals with prior infections (i.e., those who have experienced chickenpox or herpes zoster symptoms) [17, 18]. One proposed mechanism by which VZV may confer protective effects against glioblastoma is its ability to trigger virus-directed immune responses, coupled with cross-reactivity with proteins on glioblastoma cells, thus eliciting a protective immune response against newly emerging tumor cells in the brain [3]. Reported lower levels of anti-VZV IgG in glioblastoma cases compared to controls support the hypothesis that serological responses to VZV may have a protective role [19]. Moreover, in a prospective study investigating the association between prior viral infection and glioma risk, Coghill et al. found that Epstein Barr virus (EBV) infection is related to lower glioma risk (OR = 0.57, 95% CI, 0.38–0.85) [20]. In addition, Vidone et al. reported the presence of human papillomavirus (HPV) in GBM patient tumor tissues and that it is an unfavorable prognostic factor for glioma [21]. However, no definitive conclusion has been drawn about a causal relationship between these viral infections and glioma risk due to the methodological biases inherent in observational studies and small sample sizes [16]. Additionally, it is unethical to investigate a causal relationship between viral infection and glioma through randomized controlled trials [22, 23]. Furthermore, given that many coronavirus disease 2019 (COVID-19) patients have exhibited varying degrees of cognitive dysfunction, it would be of special interest to know whether its sequelae lead to glioma [24]. Overall, the relationships between some invasive viral infections, such as hepatitis B virus (HBV), mumps virus (MuV), and rubella virus (RuV), and glioma remains unknown. Hence, there is an urgent need for a comprehensive study with a rigorous approach that leads to a definitive conclusion about the causal relationship between viral infection and glioma.

Mendelian randomization (MR) is an effective method using genetic variation as an instrumental variable (IV) to assess the association between exposure and disease [25]. MR analysis reduces confounding and reverse causality due to the segregation and independent assortment of genes passed from parents to offspring. In the absence of pleiotropy (that is, genetic variation related to a disease via other pathways) and demographic stratification, MR can present a clear estimate of risk of disease [26]. MR analysis is increasingly used to determine a causal relationship between potentially modifiable risk factors and outcomes [27]. For example, MR analysis has been applied to propose the causal relationship between glioma and the length of leukocyte telomeres [28]. Recently, Saunders et al. used MR to assess the impact of potential risk factors on gliomagenesis, including diet and lifestyle, but found no causation [27]. To date, the causal relationship between viral infection and glioma remains poorly understood and unestablished.

In this study, a two-sample MR analysis was applied to assess the causal relationship between glioma and infection by 12 types of viruses (VZV, HSV, Severe acute respiratory syndrome coronavirus (SARS-CoV-2), MuV, poliovirus, human immunodeficiency virus (HIV), HBV, RuV, MeV, HPV, HCMV, and EBV) that have either previously been investigated using traditional observational studies or not researched. The ultimate goal of this MR analysis was to clarify the direction and magnitude of the causal relationship between viral infection and glioma risk. Due to the high degree of heterogeneity present in glioma, tumors with various grades and subtypes have different biological and genetic characteristics [29]. Therefore, we further divided outcome analysis into glioblastoma (grade IV glioma) or nonglioblastoma (LGG) cases for subtype analysis. In the current study, we performed a two-sample MR analysis to evaluate whether viral infection is causally associated with glioma risk, with specific attention to different glioma subtypes.

## Methods

### Exposure and glioma GWAS dataset

For VZV infection, genetic instrumental variants for herpes zoster (caused by VZV infection) were obtained from FinnGen Biobank. Infection with herpes zoster diagnoses were identified using International Classification of Diseases (ICD) codes in Finnish registries of inpatients, outpatients, and causes of death. The R5 release included 2080 cases, and 211,856 European pedigrees were used for the control sample.

To retrieve data for cold sores (caused by HSV infection), we applied GWAS summary statistics obtained from the 23andMe cohort [30]. Individuals included in the 23andMe GWAS analysis were selected based on a rigorous set of self-report questionnaires regarding history of infection (25,108 cases and 63,332 controls from Europe). In addition, GWAS summary statistics for 213,451 individuals (1595 cases and 211,856 controls) were obtained from FinnGen Biobank for herpesvirus infections.

The GWAS summary statistics of COVID-19 hospitalized patient data were obtained from COVID-19 Host Genetics Initiative (HGI) (https://www.covid19hg.org/results/r5/), consisting of 9986 cases and 1,877,672 controls from Europe [31].

GWAS summary statistics for mumps were obtained from the 23andMe cohort (31,227 cases and 54,153 controls of European ancestry) [30] and FinnGen Biobank, consisting of 436 cases and 213,666 controls [32].

GWAS summary statistics of acute poliomyelitis and HIV disease (caused by poliovirus infection and HIV infection, respectively) were obtained from FinnGen [32].

For hepatitis, rubella, and measles virus infection, GWAS summary statistics were procured from FinnGen [32] and 23andMe cohorts of European ancestry [30].

GWAS summary statistics of E7 antigen for HPV16 were retrieved from one GWAS conducted in the Karsten Suhre cohort [33], which included 1338 European individuals. The GWAS summary statistics of HCMV infections included 270 cases and 213,666 controls from FinnGen Biobank.

Genetic instrumental variants for mononucleosis (caused by EBV infection) were obtained from FinnGen Biobank (1238 cases and 213,666 controls) [32] and the 23andMe cohort (17,457 cases and 68,446 controls of European ancestry) [30].

Two-sample MR studies were based on the premise that exposure and outcome are independent samples. Thus, GWAS summary statistics of glioma phenotypes that significantly overlapped with the above viral infections were excluded. Finally, GWAS data for glioma were collected from the largest meta-analysis GWAS, involving 6183 GBM and 5820 LGG (non-GBM) cases and 18,169 controls of European ancestry from eight independent GWAS datasets [34] (Additional file 2).

### MR design

We performed a two-sample bidirectional MR study according to well-established large queues and consortia. Specifically, we considered 12 types of viral infections (VZV, HSV, SARS-CoV-2, MuV, poliovirus, HIV, HBV, RuV, MeV, HPV, HCMV, and EBV).

**Fig. 1:**
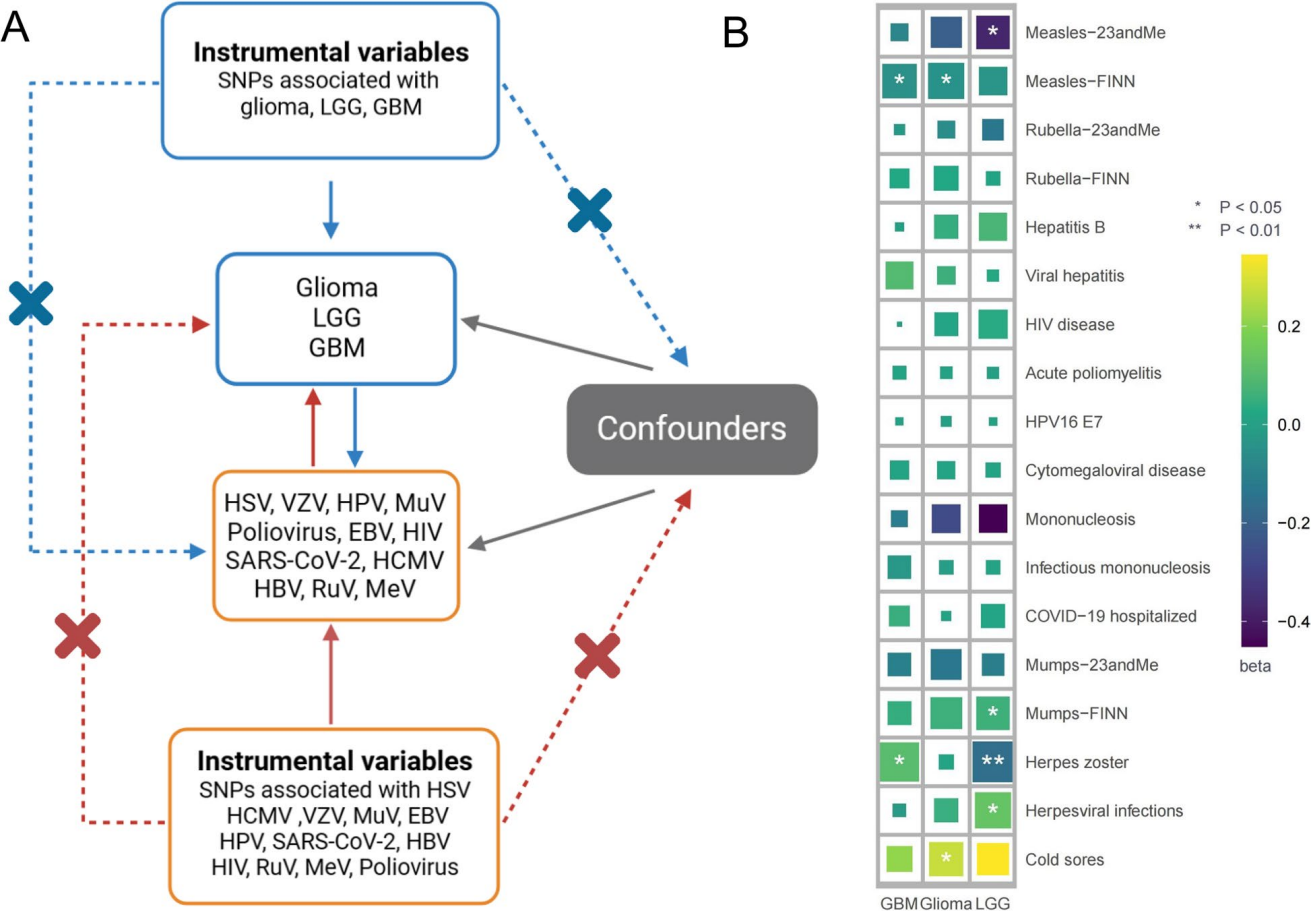
**A** Basic assumptions of Mendelian randomization. **B** Primary analysis of the association of viral infection with risk of LGG, GBM, and glioma

MR should be conducted under three fundamental premises: (1) genetic variations are strongly related to exposure; (2) genetic variations are independent of any potential confounders; and (3) genetic variations are independent of outcome, except by means of exposure (Fig. 1A). In addition, other assumptions should be met, which include the linearity and absence of statistical interactions [23]. To identify enough SNPs (number > 3) in common between exposure and outcome, SNPs with genome-wide suggestive significance *P* values (*P* < 5 × 10^–6^) were selected [35, 36]. SNPs with the largest effect sizes are robust and dependable for conducting MR, even though lowering the *P*-value threshold would introduce potential false positive SNPs as instruments. None of the instrumental SNPs were in linkage disequilibrium (LD), as this situation may cause a misleading outcome. To achieve this, a clumping process was employed, wherein SNPs were clumped based on LD in the given genome region. Independent SNPs were identified through the clumping process, using a threshold of *r*^2^ < 0.001 and a window size of 10,000 kb. We calculated the proportions of phenotypic variation interpreted by IV and assessed the intensity of the selected SNPs with the *F* statistic (*F* = beta^2^/se^2^) to present the strength of the instruments [25]. SNPs with strong instrumentation were identified as having an *F*-statistic > 10 (Additional files 1 and 6).

### Statistical analysis

We first evaluated the causality of each SNP using the Wald ratio. If more than one SNP could be used as a tool for IV, the inverse variance weighted (IVW) method was used for the meta-analysis of Wald estimates. The meta-analysis of Wald estimates (*β*) for each SNP was calculated by the IVW method as follows: $\upbeta = \frac{\sum\nolimits_{k} X_{k} Y_{k} {\sigma_{Y_{k}}}^{\!\!-2} }{\sum\nolimits_{k} X_{k}^{2} {\sigma_{Y_{k}}}^{\!\!-2} }$ where *X*_*k*_ represents the association of SNP_k_ with the exposure trait and *Y*_*k*_ corresponds to the association of SNP_k_ with outcome risk with the standard error ${\sigma }_{{Y}_{k}}$. IVW is the most valid method with the best available statistical power, but it presumes that all instrumental covariates are effective, and it deviates if the mean multifactor effect varies from zero. Additionally, MR–Egger and weighted median methods were utilized as a complement to IVW [37]. Under the assumption that at minimum, 50% of SNPs are effective, the weighted median method yields consistent causal estimates. When heterogeneity was high, a random effects model was used.

Additionally, the MR-Egger intercept [38] and MR-PRESSO [39] (Mendelian Randomization Pleiotropy Residual Sum and Outlier) tests were conducted to assess the presence of horizontal pleiotropy and outlier SNPs. A *P* value of the MR-Egger intercept of more than 0.05 indicates no horizontal pleiotropic effects. If outliers were detected, we present the MR causal estimate recalculated by the MR-PRESSO method as the main result; otherwise, we would adopt the IVW method. To further ensure the robustness of our MR analysis, we used Cochran *Q* statistics to calculate heterogeneity among SNPs [40]. To identify potentially influential SNPs, we performed a “leave-one-out” sensitivity analysis in which we excluded one SNP at a time and performed an IVW-random method on the remaining SNPs to identify the potential influence of outlying variants on the estimates. Forest and scatter plots were produced for further evaluation of heterogeneity. The analysis was performed by using the “TwoSampleMR” and “MRPRESSO” packages in the R 4.1.2 software.

For nonbinary exposure, we report MR results using odds ratios (ORs) and 95% confidence intervals (CIs) per standard deviation (SD) unit increase in each viral infection. For binary viral infection exposure and disease outcome (GBM, LGG, and all-glioma), ORs were converted to represent the OR per one-unit increase in logOR of viral infection. To rectify the bias from multiple comparisons, we used a Benjamini–Hochberg false discovery rate (FDR). A causal relationship was concluded if the direction and estimates of the causal effects of the IVW and weighted median methods were consistent and the *P* value with the FDR was less than 0.05 after correction for heterogeneity and horizontal polymorphism. A *P* value less than 0.05 but with an FDR greater than 0.05 was interpreted as a suggestive causal relationship. We meta-analyzed the estimate from different data sources if applicable. A random-effects model was employed if strong heterogeneity was detected. In addition, the causal association was also verified by the “GRAPPLE” and “mr.raps” packages [41, 42]. Finally, mRND was used to calculate the statistical power of Mendelian randomization (https://cnsgenoics.shinyapps.io/mRND/).

## Results

The summary information of the enrolled GWAS studies is shown in Table 1. In summary, 19 GWASs (18 GWASs of viral infections and 1 GWAS of glioma) and 12,488 glioma cases were enrolled in this MR study. The number of SNPs ranged from 4 to 48, and the variance explained ranged from 1.2 to 72.7% (Table 1). The *F* statistics of each SNP were all above the threshold of 10, indicating that all SNPs were robust (Additional file 1 and 6).

**Table 1:** A brief description of each GWAS summary statistics

Exposure/outcome	Unit	Sample size	Number of SNPs	Consortium/first author	Year of publication	PubMed ID	R2 (%)	NSNP	*F*
Cold sores	1 unit in logOR	88,440	1,840,001	23andMe	2017	28928442	1.91	7	27.86
Herpes viral infections	1 unit in logOR	213,451	16,380,457	FINN	2021	36653562	11.59	8	21.9
Herpes zoster	1 unit in logOR	213,936	16,380,433	FINN	2021	36653562	9.47	8	22.57
Mumps	1 unit in logOR	214,102	16,380,458	FINN	2021	36653562	35.09	6	21.72
Mumps	1 unit in logOR	85,350	1,840,001	23andMe	2017	28928442	5.33	15	46.21
Infectious mononucleosis	1 unit in logOR	214,904	16,380,461	FINN	2021	36653562	47.44	22	23.18
Mononucleosis	1 unit in logOR	85,903	1,840,001	23andMe	2017	28928442	1.22	8	26.25
Cytomegaloviral disease	1 unit in logOR	213,936	16,380,457	FINN	2021	36653562	63.66	7	21.89
COVID-19 hospitalized	1 unit in logOR	1,887,658	8,107,040	COVID-19 HGI	2020	32404885	18.01	29	35.02
HPV16 E7	SD	1388	508,253	Suhre	2019	28240269	16.62	4	21.87
Acute poliomyelitis	1 unit in logOR	217,867	16,380,460	FINN	2021	36653562	72.69	5	23.96
HIV disease	1 unit in logOR	218,792	16,380,460	FINN	2021	36653562	67.35	9	23.02
Viral hepatitis	1 unit in logOR	218,792	16,380,460	FINN	2021	36653562	15.32	7	22.62
Hepatitis B	1 unit in logOR	219,605	1,840,001	23andMe	2017	28928442	55.29	11	23.3
Rubella	1 unit in logOR	212,409	16,380,460	FINN	2021	36653562	43.05	9	22.82
Rubella	1 unit in logOR	83,597	1,840,001	23andMe	2017	28928442	2.63	9	22.31
Measles	1 unit in logOR	85,498	16,380,460	FINN	2021	36653562	70.14	8	23.41
Measles	1 unit in logOR	212,036	1,840,001	23andMe	2017	24880342	4.79	10	22.79
Glioma	1 unit in logOR	30,657	6,901,311	Melin	2017	283456443	19.78	45	22.76
LGG	1 unit in logOR	23,989	6,782,053	Melin	2017	283456443	36.19	48	26.17
GBM	1 unit in logOR	24,352	6,801,179	Melin	2017	283456443	22.18	37	24.57

*Abbreviations*: *NSNP* the number of single nucleotide polymorphism, *R*^*2*^ variance of phenotype explained by SNPs, *logOR* logarithm of odds ratio, *F* F statistics, *SD* standard deviation, *COVID-19* coronavirus disease 2019, *HPV* human papillomavirus, *HIV* human immunodeficiency virus, *LGG* lower-grade glioma, *GBM* glioblastoma

### Causal association of viral infection with glioma

**Fig. 2:**
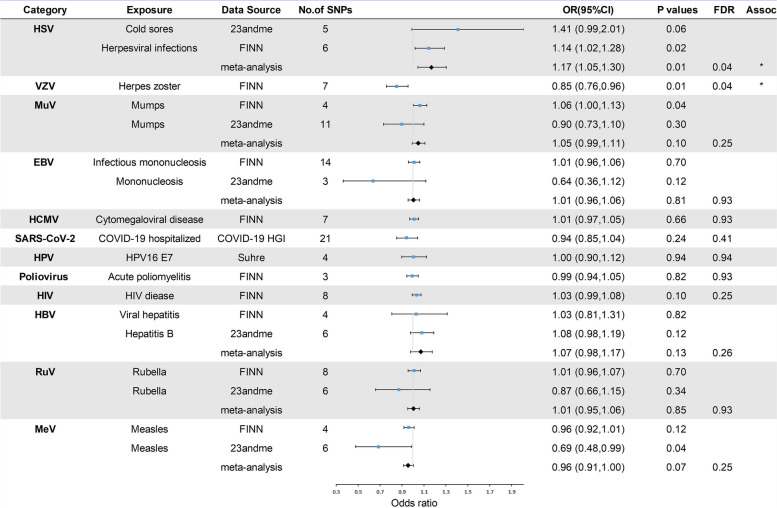
Odds ratios for associations (Assoc.) between genetically predicted viral infection and LGG. *Significant *P* values (FDR *P* < 0.05)

**Fig. 3:**
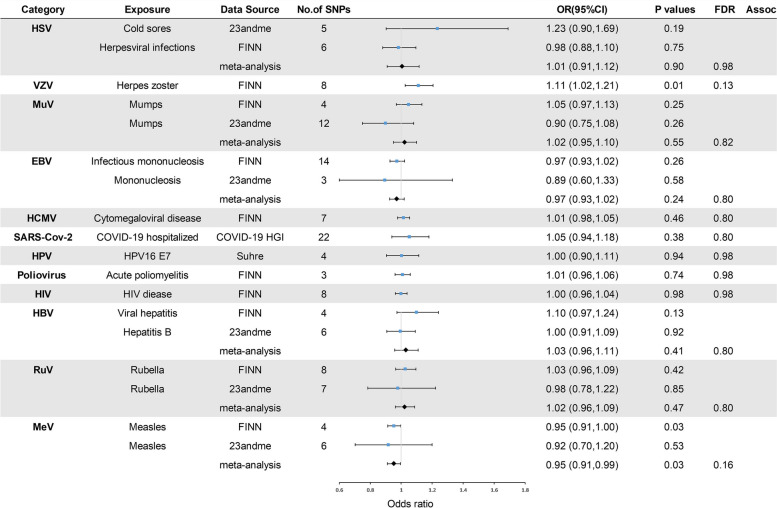
Odds ratios for associations (Assoc.) between genetically predicted viral infection and GBM. *Significant *P* values (FDR *P* < 0.05)

**Fig. 4:**
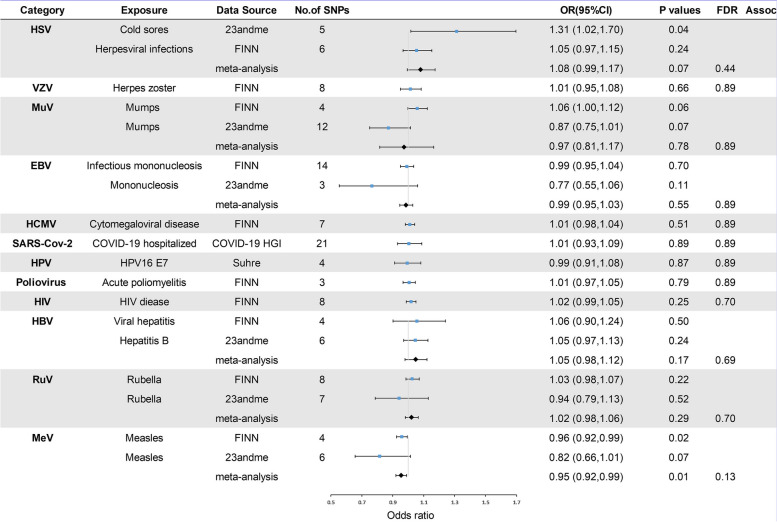
Odds ratios for associations (Assoc.) between genetically predicted viral infection and all-glioma. *Significant *P* values (FDR *P* < 0.05)

In the primary analysis, the number of SNPs used for each pair of viral infections and outcome MR estimates varied from 3 to 22 (Additional file 1). A total of 8 causative relationship features were identified at *P* < 0.05 (Fig. 1B). Figures 2, 3, and 4 and Additional file 3 show the outcome of MR analysis and the sensitivity analysis of the causal association of several kinds of viral infection with glioma.

We found that genetically predicted herpes zoster and HSV infection could change the risk of LGG after FDR control (FDR < 0.05). The association between genetically predicted HSV infection and LGG risk still held true in different cohorts, as shown in Fig. 2 (23andMe and FinnGen). Additionally, genetically predicted symptom caused by MuV (OR = 1.05, *P* = 0.10), EBV (OR = 1.01, *P* = 0.81), HCMV (OR = 1.01, *P* = 0.66), SARS-CoV-2 (OR = 0.94, *P* = 0.24), HPV (OR = 1.00, *P* = 0.94), HBV (OR = 1.07, *P* = 0.13), RuV (OR = 1.01, *P* = 0.85), or MeV (OR = 0.96, *P* = 0.07) infection had a null causal relationship with LGG risk. Interestingly, we found that genetically predicted herpes zoster had a suggestive causal relationship with GBM (FDR > 0.05 and IVW *P* < 0.05) and that genetically predicted measles was nominally associated with GBM and all-gliomas (FDR > 0.05 and IVW *P* < 0.05). The direction of the causality between genetically predicted measles and GBM and all gliomas was consistent in different cohorts, as shown in Figs. 3 and 4 (23andMe and FinnGen). Additionally, no association (*P* > 0.05) was identified between genetically predicted symptom caused by MuV, EBV, HCMV, SARS-CoV-2, HPV, HBV, RuV, and HIV infection and risk of GBM and all-glioma (Figs. 3 and 4).

We also found genetically predicted herpes zoster was associated with a reduced risk of LGG (OR = 0.85, *P* = 0.01, FDR = 0.04) (Fig. 2), while genetically predicted HSV infection was linked to an increased risk of LGG (OR = 1.17, *P* = 0.01, FDR = 0.04). Furthermore, our Mendelian randomization analyses showed suggestive evidence that genetically predicted herpes zoster exhibited an association with GBM risk (OR = 1.11, *P* = 0.01, FDR = 0.13) and genetically predicted measles appeared to confer a protective effect against GBM (OR = 0.95, *P* = 0.03, FDR = 0.16). Moreover, the data suggested that genetically predicted measles could reduce the risk of all-glioma (OR = 0.95, *P* = 0.01, FDR = 0.13). The heterogeneity test indicated no significant heterogeneity among the selected IVs (Q_pval > 0.05) in the causative relationship. No outliers were detected, and the MR estimates were represented by the IVW method. Pleiotropy analyses by MR-Egger analysis suggested no horizontal pleiotropy (Additional file 3). Leave-one-out analysis demonstrated that the causal relationship between herpes zoster and LGG remained robust. However, the causal effect of genetically predicted HSV infection on LGG, herpes zoster on GBM, measles on GBM, and all-glioma relied on particular SNPs, indicating the casualties were not robust (Additional file 4), with casualties no longer existing when excluding such SNPs. The raw results of heterogeneity analysis and pleiotropy analysis are included in Additional file 3 as well as the weighted-median and MR-Egger results. In addition, the direction of estimated causal effect of herpes zoster on LGG risk from GRAPPLE method is the same as IVW method (OR = 0.625), but the *P* value is not significant (*P* = 0.85). The results from MR.RAPS showed that the estimated causal effect is 0.849, with *P*-value of 0.01 (shown in Additional file 5), which accorded with the estimate from IVW method (OR = 0.85, *P* = 0.01). Considering the sample size of glioma, the statistical power for herpes zoster for LGG was 82%; the statistical power for viral infection overall ranged from 5 to 100% (Additional file 3).

### Causal association of glioma with viral infection

**Fig. 5:**
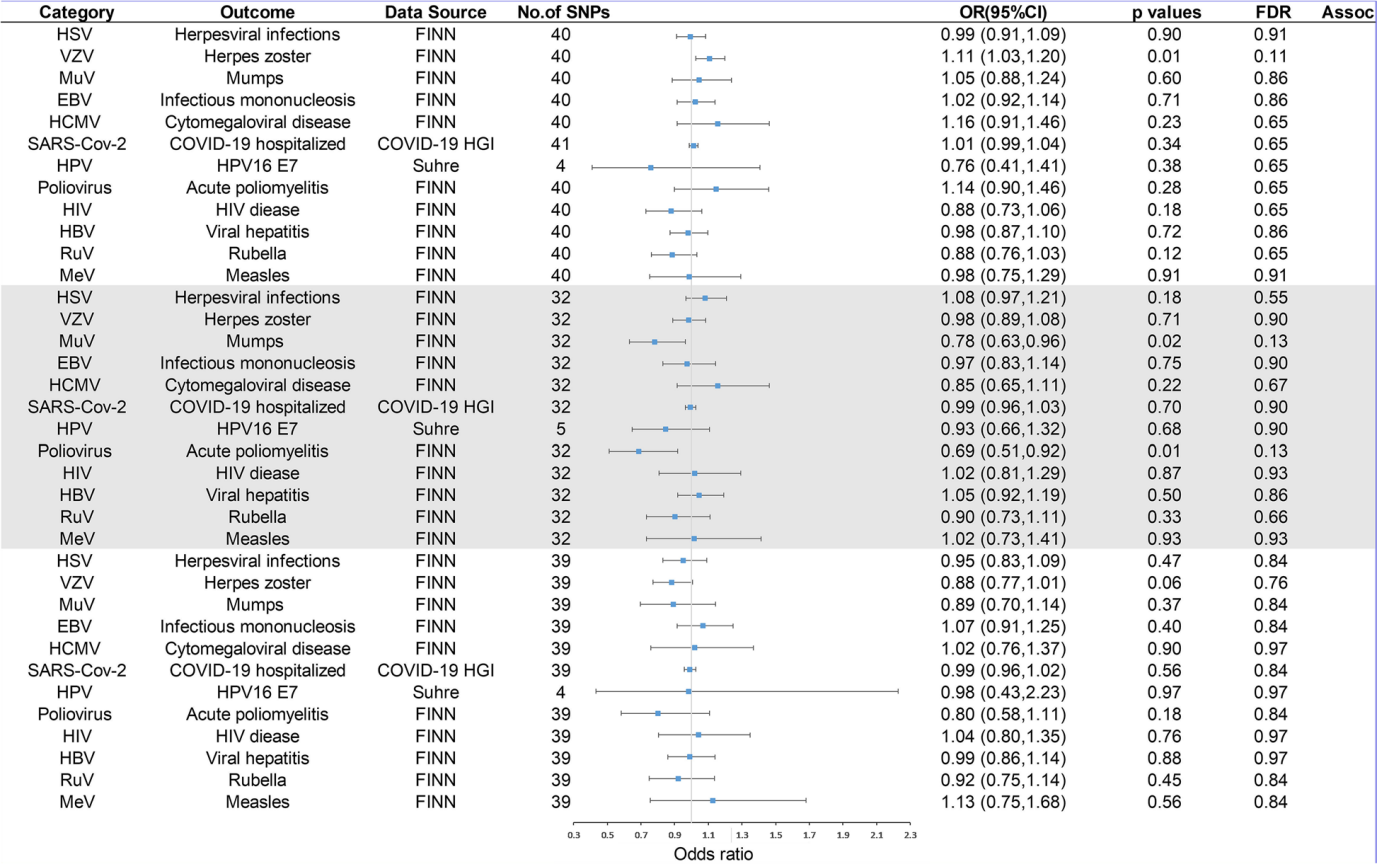
Odds ratios for associations (Assoc.) between genetically predicted glioma and viral infection. *Significant *P* values (FDR *P* < 0.05)

For analysis of the causal effect of glioma on viral infection, we identified SNPs associated with types of glioma as exposure IVs. Nonetheless, these SNPs were ascertained in only 12 viral infection types, and the number of SNPs used for MR estimates ranged from 4 to 41 (Additional file 6). The results of MR analysis and sensitivity analysis of the causal relationship between glioma and viral infection are summarized in Fig. 5 and Additional file 7.

We found that genetically predicted GBM had a suggestive causality with a lower risk of mumps (OR = 0.78, *P* = 0.02, FDR = 0.13) and acute poliomyelitis (OR = 0.69, *P* = 0.01, FDR = 0.13), whereas genetically predicted LGG increased risk of herpes zoster (OR = 1.11, *P* = 0.01, FDR = 0.11), though these relationships were not detected by the weighted median method. In addition, no causal effects (*P* > 0.05) were identified between genetically predicted GBM, LGG, and all gliomas with risk of HSV, EBV, HCMV, SARS-CoV-2, HPV, HBV, RuV, and MeV infection (Fig. 5). Furthermore, Cochran’s *Q* test revealed no significant heterogeneity among the selected IVs (Q_pval > 0.05) in the causative relationship (Additional file 7). Pleiotropy analyses showed no horizontal pleiotropy for our MR results. Nevertheless, leave-one-out analysis suggested that the association of genetically predicted GBM with mumps risk was dependent on specific SNPs (Additional file 8), with the causalities lack of robustness. Considering the sample size of glioma, the statistical power for each pair of relationships ranged from 6 to 100% (Additional file 7).

## Discussion

Gliomas are characterized by a high degree of malignancy, aggressiveness, and morbidity [43]. Researchers have focused on the diagnosis, treatment, and recurrence of gliomas, but less attention has been given to their etiology. Limited by methodological, ethical, and multiple factors, exploring the etiology of gliomas, the most common primary tumors of the central nervous system, is difficult and complex. Viral infection is known to be a risk factor for many cancers, including nasopharyngeal carcinoma and cervical cancer [10]. The results of a previous research indicated that genetic predisposition toward increased seroreactivity to EBV ZEBRA was associated with a decreased overall glioma risk [44]. Despite previous studies, the association between viral infection and glioma incidence remains controversial [15]. In general, establishing causal relationships is difficult due to the limitations of small sample sizes and inherent biases. As a result, very few studies have explored causal relationships between viral infection and glioma. We sought to apply MR, which can overcome the methodological obstacles mentioned above, to elucidate such causality. MR is an analytic research technique using genetic variation as a proxy for exposure. We used MR analysis to investigate a range of viral infection factors and their causation with cancer risk. To date, our work is the most comprehensive MR study to illustrate the causal relationship between viral infection and glioma. Our MR results confirm that genetically predicted herpes zoster is significantly associated with lower risk of LGG after correcting for multiple tests (FDR < 0.05) and sensitivity analysis. Additionally, we found that genetically predicted GBM has suggestive causality with lower risk of acute poliomyelitis (OR = 0.69, *P* = 0.01, FDR = 0.13); genetically predicted LGG also shows suggestive causality with higher risk of herpes zoster (OR = 1.11, *P* = 0.01, FDR = 0.11).

### The association between genetically predicted herpes zoster and glioma

Our MR results confirmed that genetically predicted herpes zoster was significantly associated with lower risk of LGG. Interestingly, the causal associations for GBM and LGG were completely opposite: our MR analysis indicated that herpes zoster increased risk of GBM (OR = 1.11, *P* = 0.01, FDR = 0.13) but decreased that of LGG (OR = 0.85, *P* = 0.01, FDR = 0.04). Based on the results of the leave-one-out method, the MR analysis of herpes zoster and LGG was responsible, and single SNPs did not affect the results. However, this trend with GBM failed to remain consistent in leave-one-out tests, which indicated that it was driven by one SNP in ABCB11, rs75043801. ABCB11, also known as BSEP, encodes bile salt outlet pump (BSEP), which plays an important role in transporting bile acid (BA). Our results suggest that this particular SNP in ABCB11 may dominate the estimation of the causal effect of herpes zoster on GBM, which means the selection of SNPs in the GWAS may influence the soundness of the results.

In previous studies, VZV was the only virus found to be negatively related to glioma. One of the largest studies on the subject to date, International Case–Control Study of Glioma (GICC) collected data for 8704 individuals from several countries and confirmed the negative association between history of chickenpox and glioma [31]. The results showed that varicella history was associated with lower risk of glioma (OR = 0.79, 95% CI: 0.65–0.96) [45]. More specifically, the authors demonstrated that VZV infection provides a strong protective effect against GBM. The same results were validated in other cohorts: Sjostrom et al. investigated three large cohorts consisting of prediagnostic samples and found that anti-VZV IgG levels are related to reduced glioma risk (OR = 0.63, 95% CI: 0.37–1.08) [19]. Additionally, it has been reported that the level of VZV IgG is significantly low (OR = 0.68, 95% CI: 0.41–1.13) in glioma patients [32]. However, without further establishing causality, these studies remain at the stage of simple observation. More bench research focusing on validating the causation between VZV infection and glioma is needed to elucidate the underlying mechanism. Our MR results substantiate the causal relationship between herpes zoster and glioma, providing further reinforcement to the previous perspective.

### Potential mechanism

It is not clear by what biological mechanism VZV infection protects against LGG, but several hypotheses have been proposed [46, 47]. Previous studies have found that immune-related diseases such as allergies and asthma reduce risk of glioma, suggesting that activation of the immune system might play an important protective role in suppressing glioma development [46]. Similarly, we propose that VZV may share epitopes with LGG tumor cells and that its antibodies may be cross-reactive with tumor cells, allowing the immune system to mount protective immune responses against tumor cells. Additionally, some studies have found that VZV infection changes the systemic immune effect, recruits NK cells and T lymphocytes, and increases inflammatory factors [47, 48], which are also known mechanisms of oncolytic viruses [49]. After primary infection, VZV maintains latency in the nervous system. After reactivation, VZV may help to destroy tumor cells and enhance the local immune response against them. All of these anti glioma characteristics suggest that VZV is a better vector for oncolytic virus development than other virus vectors. Henning Leske et al. studied the oncolytic potential of VZV in glioma cell cultures and the tumor-targeting potential of human mesenchymal stem cells infected by VZV. Their results showed that VZV replicates rapidly in all glioma cells studied and dissolves tumors in vitro. Additionally, it was found that human mesenchymal stem cells were able to target varicella-zoster virus to tumor growth sites. Overall, VZV shows great oncolytic capacity in glioma cell cultures and may be an ideal candidate for glioma virus therapy.

### The potential association between other genetically predicted virus infections and gliomas

#### HSV infection

Additionally, we demonstrated by MR that HSV infection increases risk of LGG in genetically susceptible patients. Our MR analysis results indicated a suggestive causal relationship between HSV infection and glioma, though it did not remain robust in the validation test. Specifically, leave-one-out analysis revealed that the relationship was driven by the SNPs rs34264769, rs4885004 (in SNORA68), and rs9289557 (in MRAS). When any one of these SNPs was removed, no suggestive association remained, which means the MR results lack robustness due to the presence of these SNPs. SNORA68 is a small nucleolar RNA (snoRNA) located at p13.1 on chromosome 19 (19p13.1) that is associated with susceptibility to ovarian and breast cancer in individuals with BRCA1 or BRCA2 mutation [50]. Bolton et al. found that two SNPs at 19p13.1, rs8170 and rs2363956, are associated with patient survival in a cohort of 8,951 cases. In addition, high expression of SNORA68 is related to poor prognosis in several cancers, including ovarian cancer and non-small cell lung cancer (NSCLC) [51]. MRAS is similar to classical RAS oncoproteins, with many similar regulatory functions. It plays a vital role in cell differentiation and proliferation as well as cell polarity. However, in stark contrast to RAS, activating mutations in MRAS are rarely found in cancer [52]. Nonetheless, dysregulation of MRAS expression might be a contributing factor to tumorigenesis in some cases [53, 54]. Large-scale GWAS have identified MRAS sites as risk factors for cardiovascular disease [55]. Several observational studies have reached the same conclusion that HSV infection may increase risk of glioma: most GBM patients are serologically positive for HSV antibodies, suggesting that HSV may participate in the pathogenesis of glioma [17, 56]. Regardless, few studies have confirmed any causal relationship. HSV, as part of the *Herpesviridae* family, can remain latent in the nervous system and is well known for its neurovirulence [57]. Previous research proposed a possible mechanism by which miRNA-H16 encoded by HSV-1 induces NOTCH signaling pathway overactivation, which plays crucial roles in glioma cell survival and progression, thereby initiating glioma tumorigenesis [58, 59].

#### MeV infection

We also found suggestive evidence for the association between MeV infection and glioma. MeV infection reduced risk of GBM and all-glioma in patients genetically susceptible to MeV, which contradicts previous epidemiological study results [60]. Possible explanations for this discrepancy include that the underlying link obtained from previous case–control studies might be correlational but not causal and that such studies usually suffer from recall bias. Another possible explanation is the existence of reverse causality. Specifically, patients with glioblastoma may be immunocompromised [61], and more susceptible to measles virus infection, leading to the association between measles virus infection and glioma that we observed. In addition, our reverse MR estimate revealed no causal relationship between glioma and MeV infection. In recent years, oncolytic measles virus has been reported to be a novel treatment for glioma, and Cory Allen has proved that measles virus derivatives have significant antitumor activity against glioma-derived stem cells both in vitro and in vivo [62]. Therefore, further research is needed to examine the association between actual measles virus infection and glioma risk.

#### SARS-CoV-2 infection

As the pandemic progresses, the sequelae of COVID-19, including cardiovascular, pulmonary, and neurological diseases, are raising concerns about the long-term outcomes of SARS-CoV-2 infection, especially in heavily affected areas [63, 64]. Glial cells express SARS-COV-2 receptors such as angiotensin converting enzyme 2 (ACE2) and cathepsin L (CTSL), which may be responsible for making glioma patients more susceptible to SARS-CoV-2 infection and at higher risk for severe COVID-19 [65, 66]. However, the role of SARS-CoV-2 in glioma development remains unclear [67]. Our findings suggest that there is no causal association between SARS-COV-2 infection and glioma risk. Nevertheless, further research is necessary to substantiate that.

### Strengths and limitations

Our study has several strengths. First, this is the first study to draw causal conclusions and eliminate confounding factors and reverse causality using the two-sample MR method to investigate the relationship between viral infection and glioma. Second, by using glioma data derived from the largest GWAS dataset (12,488 cases and 18,169 controls) and viral infection exposure data from credible large-scale GWAS databases (COVID-19 up to 1,887,658 individuals), our current study demonstrates solid validity and generalizability compared with traditional studies. Third, we included novel factors never studied before in our MR analysis, such as mumps and HPV infection. However, some limitations in this research need to be noted, including the lack of stratification of sex or age in the GWAS data and the lack of genetic data, as we were limited to using genome-wide association data of European ancestry only; ideally, we hope to expand the analysis to include all populations when possible. We use a suggestive genome-wide *P*-value threshold at 5 × 10^−6^, which may lead to false positive SNPs. But we additionally calculated *F*-statistics and *R*^2^ to transparently present the strength of our instruments. The *F*-statistics of all the selected SNPs are above the threshold of 10. And the *R*^2^ of the instrumental variables is calculated; SNPs selected as instrumental variables explain around 9.5% for herpes zoster. These results indicate that all the SNPs we selected are qualified and robust enough to carry out rigorous MR analyses. Besides, we do not find the association between herpes zoster and LGG risk by GRAPPLE method. We speculate that this may be related to GRAPPLE’s need for a separate GWAS cohort of exposure for selecting SNPs. Besides, the authors of GRAPPLE package also mentioned that in some areas, it is difficult to obtain multiple high-quality aggregated public GWAS statistics with non-overlapping cohorts in some areas [41].

## Conclusions

For the first time, we show evidence supporting that genetically predicted herpes zoster caused by VZV infection can reduce risk of LGG by using two-sample MR. The findings of our study provide further insight into the etiology of glioma and warrant further research to uncover mechanisms that implicate traits in glioma onset.

### Supplementary Information

**Additional file 1: Table S1.** Single SNP analysis of the association between viral infection and LGG. **Table S2.** Single SNP analysis of the association between viral infection and GBM. **Table S3.** Single SNP analysis of the association between viral infection and all-glioma.

**Additional file 2. **Summary of the 8 glioma GWASs used in the meta-analysis.

**Additional file 3. **Mendelian randomization results of weighted median and MR–Egger methods, sensitivity analysis, for viral infection in glioma. Abbreviations: *P* (heterogeneity): *P* value of Cochrane’s Q value in heterogeneity test; P (pleiotropy): *P* value of MR–Egger intercept.

**Additional file 4. **Leave-one-out plots, forest plots, and scatter plots in primary analysis.

**Additional file 5. **Estimated causal effect between genetically predicted herpes zoster and LGG.

**Additional file 6: Table S1.** Single SNP analysis of the association of LGG on viral infection. **Table S2.** Single SNP analysis of the association of GBM on viral infection. **Table S3.** Single SNP analysis of the association of all-glioma on viral infection.

**Additional file 7. **Mendelian randomization results of weighted median and MR–Egger methods, sensitivity analysis, for GBM, LGG, and all-glioma on viral infection. Abbreviations: *P* (heterogeneity): *P* value of Cochrane’s Q value in heterogeneity test; P (pleiotropy): *P* value of MR–Egger intercept.

**Additional file 8. **Leave-one-out plots, forest plots, and scatter plots in the reverse MR estimate.

**Additional file 9. **Source code.

**Additional file 10. **Retouching certificate.

## Data Availability

All data sources used in this MR study can be acquired from the original genome-wide association studies that are mentioned in the text. GWAS summary statistics for glioma can be downloaded from Melin’s meta-analysis [34]. GWAS summary statistics for virus infection from FinnGen can be downloaded from the FinnGen consortium website (https://fnngen.gitbook.io/documentation/data-download). GWAS summary statistics for virus infection from 23andMe can be downloaded from Tian's GWAS analysis [19]. Any other data generated in the analysis process can be requested from the corresponding author.

## Abbreviations

ABCB11: ATP binding cassette subfamily B member 11
CMV: Cytomegalovirus
COVID-19 HGI: Coronavirus disease 2019 Host Genetics Initiative
EBV: Epstein-Barr virus
HCMV: Human cytomegalovirus
HSV: Herpes simplex virus
HPV: Human papillomavirus
HIV: Human immunodeficiency virus
HBV: Hepatitis B virus
FDR: False discovery rate
GBM: Glioblastoma
GWAS: Genome-wide association study
IV: Instrumental variable
IVW: Inverse-variance weighted
KORA: Cooperative Health Research in the Region of Augsburg
LD: Linkage disequilibrium
LGG: Lower-grade glioma
MuV: Mumps virus
MeV: Measles virus
MRAS: Muscle RAS Oncogene Homolog
MR: Mendelian randomization
MR-PRESSO: Mendelian Randomization Pleiotropy Residual Sum and Outlier
RuV: Rubella virus
SNP: Single-nucleotide polymorphisms
SARS-CoV-2: Severe acute respiratory syndrome coronavirus 2
SNORA: Small nucleolar RNA
VZV: Varicella-zoster virus
