# Supplementary material for: Association between viral infections and glioma risk: a two-sample bidirectional Mendelian randomization analysis
Source: BMC Med. 2023 Dec 5;21:487. doi: 10.1186/s12916-023-03142-9 (PMC10698979; doi:10.1186/s12916-023-03142-9)
Supplement: Supplementary file 1 — Additional file 1: Table S1. Single SNP analysis of the association between viral infection and LGG. Table S2. Single SNP analysis of the association between viral infection and GBM. Table S3. Single SNP analysis of the association between viral infection and all-glioma. [file 12916_2023_3142_MOESM1_ESM.docx]

**Additional file 1: Table S1**. Single SNP analysis of the association between viral infection and LGG

|  |  |  |  |  | **Trait** | | |  | **LGG** | | |  |
| --- | --- | --- | --- | --- | --- | --- | --- | --- | --- | --- | --- | --- |
| Exposure | SNP | Effect allele | Other allele | Effect allele frequency | Beta | Standard error | P-value |  | Beta * | Standard error * | P-value * | F-statistics |
| Cold sores | rs115789906 | G | T | 0.009784 | 0.195 | 0.042 | 2.52E-06 |  | 0.128248 | 0.0887537 | 0.148461 | 21.55612245 |
| Cold sores | rs16974161 | A | G | 0.183506 | 0.1825 | 0.0371 | 1.02E-06 |  | -0.00981743 | 0.0719205 | 0.891423 | 24.19791341 |
| Cold sores | rs17732209 | C | T | 0.176318 | 0.0586 | 0.0126 | 3.79E-06 |  | -0.0334168 | 0.0280966 | 0.234301 | 21.62988158 |
| Cold sores | rs73036068 | C | T | 0.009185 | -0.2055 | 0.0398 | 3.00E-07 |  | 0.0324345 | 0.0718244 | 0.651571 | 26.65983814 |
| Cold sores | rs885950 | A | C | 0.417133 | 0.0782 | 0.0109 | 7.47E-13 |  | -0.0531908 | 0.0243091 | 0.0286619 | 51.47075162 |
| Herpesviral infections | rs12550275 | T | C | 0.1376 | 0.2403 | 0.0526 | 4.84E-06 |  | 0.0202583 | 0.0351234 | 0.564091 | 20.87065376 |
| Herpesviral infections | rs34264769 | A | C | 0.1454 | 0.2507 | 0.0519 | 1.38E-06 |  | 0.0580547 | 0.040446 | 0.151183 | 23.33318112 |
| Herpesviral infections | rs4885004 | G | A | 0.2224 | 0.2019 | 0.0434 | 3.23E-06 |  | 0.0413471 | 0.0283233 | 0.144338 | 21.64179001 |
| Herpesviral infections | rs900978 | T | C | 0.4498 | 0.1648 | 0.036 | 4.62E-06 |  | -0.0183524 | 0.0241997 | 0.448227 | 20.95604938 |
| Herpesviral infections | rs9289557 | T | C | 0.1945 | 0.2158 | 0.0457 | 2.37E-06 |  | 0.0576367 | 0.0304056 | 0.0580136 | 22.29823461 |
| Herpesviral infections | rs9797556 | G | A | 0.3272 | 0.1837 | 0.0381 | 1.41E-06 |  | 0.0237755 | 0.0253966 | 0.349187 | 23.24707738 |
| Herpes zoster | rs10064104 | A | G | 0.3566 | -0.1506 | 0.0329 | 4.76E-06 |  | -0.00642841 | 0.0251195 | 0.798018 | 20.95357582 |
| Herpes zoster | rs10961286 | G | A | 0.1009 | 0.2602 | 0.0532 | 1.02E-06 |  | -0.0563605 | 0.0360427 | 0.117885 | 23.92166035 |
| Herpes zoster | rs11724363 | A | G | 0.3676 | 0.1543 | 0.0326 | 2.25E-06 |  | -0.0101679 | 0.0250792 | 0.68516 | 22.40250856 |
| Herpes zoster | rs1514878 | G | A | 0.2701 | 0.1728 | 0.0355 | 1.16E-06 |  | -0.0485755 | 0.0253973 | 0.0557955 | 23.69358461 |
| Herpes zoster | rs2285414 | G | A | 0.5488 | -0.1498 | 0.0316 | 2.08E-06 |  | 0.0357271 | 0.024352 | 0.142346 | 22.47240026 |
| Herpes zoster | rs2523580 | G | A | 0.3009 | -0.1637 | 0.0345 | 2.05E-06 |  | 0.0258208 | 0.0261865 | 0.324117 | 22.51433732 |
| Herpes zoster | rs6862129 | G | A | 0.03129 | 0.424 | 0.0927 | 4.80E-06 |  | -0.0661652 | 0.0740165 | 0.371363 | 20.92050891 |
| mumps-FINN | rs12881071 | A | G | 0.2841 | 0.3609 | 0.0764 | 2.31E-06 |  | 0.0467402 | 0.0271803 | 0.0854984 | 22.31449419 |
| mumps-FINN | rs1857010 | T | C | 0.5574 | 0.3209 | 0.0685 | 2.84E-06 |  | -0.0056159 | 0.0238966 | 0.814202 | 21.94614737 |
| mumps-FINN | rs192016900 | G | T | 0.04488 | 0.8148 | 0.1763 | 3.81E-06 |  | 0.05123 | 0.0654541 | 0.433812 | 21.35981152 |
| mumps-FINN | rs71488291 | G | A | 0.0153 | 1.5231 | 0.3208 | 2.05E-06 |  | 0.0943941 | 0.060591 | 0.11926 | 22.54177533 |
| mumps-23andMe | rs1061535 | C | T | 0.141174 | -0.0818 | 0.0161 | 3.76E-07 |  | -0.0112541 | 0.030546 | 0.712551 | 25.81397323 |
| mumps-23andMe | rs11160318 | A | G | 0.358227 | 0.0947 | 0.0137 | 4.76E-12 |  | -0.0168758 | 0.0258904 | 0.514519 | 47.78139485 |
| mumps-23andMe | rs111709089 | C | T | 0.026158 | -0.1558 | 0.0323 | 1.41E-06 |  | -0.105534 | 0.056102 | 0.0599568 | 23.26643599 |
| mumps-23andMe | rs13003102 | C | T | 0.013978 | -0.201 | 0.042 | 1.70E-06 |  | 0.0213547 | 0.0700616 | 0.76052 | 22.90306122 |
| mumps-23andMe | rs2139242 | C | T | 0.008387 | 0.3311 | 0.0669 | 7.45E-07 |  | -0.0169287 | 0.113471 | 0.881404 | 24.49436166 |
| mumps-23andMe | rs3862630 | C | T | 0.082468 | 0.1198 | 0.021 | 1.17E-08 |  | -0.000785165 | 0.0438222 | 0.985705 | 32.54430839 |
| mumps-23andMe | rs61960129 | A | G | 0.01238 | -0.1797 | 0.0352 | 3.31E-07 |  | -0.0153333 | 0.0638325 | 0.810167 | 26.06218524 |
| mumps-23andMe | rs72683573 | C | T | 0.037141 | -0.1131 | 0.0243 | 3.25E-06 |  | 0.0327822 | 0.0464364 | 0.480214 | 21.66270386 |
| mumps-23andMe | rs7430852 | G | T | 0.047324 | 0.1014 | 0.0218 | 3.30E-06 |  | 0.021613 | 0.0409028 | 0.597223 | 21.63530006 |
| mumps-23andMe | rs7812934 | A | C | 0.326877 | -0.0894 | 0.0178 | 5.10E-07 |  | 0.0144507 | 0.0335337 | 0.666519 | 25.22522409 |
| mumps-23andMe | rs9461562 | A | G | 0.04253 | -0.2957 | 0.0477 | 5.68E-10 |  | -0.0107349 | 0.0794807 | 0.892562 | 38.42960238 |
| Infectious mononucleosis | rs11265380 | A | G | 0.4638 | -0.1977 | 0.0412 | 1.63E-06 |  | -0.0231672 | 0.0241879 | 0.338163 | 23.02602154 |
| Infectious mononucleosis | rs112824579 | A | C | 0.1364 | -0.2956 | 0.0613 | 1.42E-06 |  | 0.0275496 | 0.0380975 | 0.469599 | 23.25347754 |
| Infectious mononucleosis | rs116856714 | T | C | 0.01357 | 0.9259 | 0.1957 | 2.22E-06 |  | 0.043441 | 0.0855234 | 0.611493 | 22.38445458 |
| Infectious mononucleosis | rs12599876 | T | C | 0.1287 | 0.2945 | 0.062 | 2.02E-06 |  | 0.0352949 | 0.0383739 | 0.357697 | 22.5625 |
| Infectious mononucleosis | rs145215254 | T | C | 0.009257 | 1.1231 | 0.2388 | 2.57E-06 |  | 0.0255901 | 0.0702189 | 0.715534 | 22.11913858 |
| Infectious mononucleosis | rs17160581 | T | C | 0.09341 | 0.3762 | 0.072 | 1.77E-07 |  | 0.0293245 | 0.0416969 | 0.481883 | 27.300625 |
| Infectious mononucleosis | rs1773231 | C | A | 0.8166 | 0.2493 | 0.0535 | 3.17E-06 |  | -0.0277981 | 0.0278641 | 0.318458 | 21.71385798 |
| Infectious mononucleosis | rs2518778 | C | T | 0.9754 | -0.6704 | 0.1402 | 1.75E-06 |  | -0.00211538 | 0.057231 | 0.970515 | 22.86504097 |
| Infectious mononucleosis | rs318491 | G | A | 0.5581 | 0.192 | 0.0419 | 4.56E-06 |  | -0.0376402 | 0.0255943 | 0.141386 | 20.99782981 |
| Infectious mononucleosis | rs34242269 | T | G | 0.4281 | -0.1943 | 0.0414 | 2.76E-06 |  | -0.00211057 | 0.0251786 | 0.933196 | 22.02647086 |
| Infectious mononucleosis | rs60492066 | C | T | 0.2927 | -0.2144 | 0.0455 | 2.48E-06 |  | -0.0266953 | 0.0293603 | 0.363229 | 22.20377249 |
| Infectious mononucleosis | rs73171666 | G | T | 0.1796 | -0.2595 | 0.0543 | 1.79E-06 |  | -0.032679 | 0.0373837 | 0.382036 | 22.83889381 |
| Infectious mononucleosis | rs76330758 | G | A | 0.02938 | 0.6048 | 0.1285 | 2.53E-06 |  | -0.118146 | 0.058575 | 0.0436949 | 22.15222274 |
| Infectious mononucleosis | rs76635736 | A | G | 0.008602 | 1.1693 | 0.2451 | 1.84E-06 |  | 0.0641684 | 0.0708968 | 0.365414 | 22.75963416 |
| Mononucleosis | rs2596465 | C | T | 0.449081 | 0.0729 | 0.0122 | 2.30E-09 |  | -0.0586976 | 0.0244415 | 0.0163257 | 35.70552271 |
| Mononucleosis | rs2612778 | C | T | 0.371605 | -0.0651 | 0.0136 | 1.69E-06 |  | -0.0121397 | 0.0270939 | 0.654111 | 22.91311635 |
| Mononucleosis | rs7487637 | A | G | 0.10643 | 0.0706 | 0.0144 | 9.45E-07 |  | -0.0374576 | 0.0275591 | 0.174091 | 24.03722994 |
| Cytomegaloviral disease | rs11658622 | A | C | 0.1903 | 0.4911 | 0.1035 | 2.10E-06 |  | -0.032423 | 0.0322369 | 0.314524 | 22.51433732 |
| Cytomegaloviral disease | rs12496650 | C | T | 0.0764 | 1.1619 | 0.2509 | 3.63E-06 |  | 0.00780282 | 0.056788 | 0.890712 | 21.44550022 |
| Cytomegaloviral disease | rs35435922 | G | A | 0.00639 | 2.7742 | 0.6058 | 4.66E-06 |  | 0.0869261 | 0.104975 | 0.407635 | 20.9708965 |
| Cytomegaloviral disease | rs66537586 | T | G | 0.008085 | 0.5315 | 0.1139 | 3.08E-06 |  | 0.00836491 | 0.0346917 | 0.809461 | 21.77504642 |
| Cytomegaloviral disease | rs6972880 | T | C | 0.9327 | -0.8905 | 0.186 | 1.69E-06 |  | 0.0351761 | 0.0616348 | 0.56819 | 22.92144323 |
| Cytomegaloviral disease | rs73087989 | G | A | 0.1942 | 0.5247 | 0.1129 | 3.38E-06 |  | 0.0165426 | 0.029481 | 0.574709 | 21.59902984 |
| Cytomegaloviral disease | rs787837 | C | T | 0.7603 | -0.4796 | 0.1021 | 2.65E-06 |  | -0.0109677 | 0.0283521 | 0.698875 | 22.06514901 |
| COVID-19 hospitalized | rs10860891 | A | C | 0.8806 | -0.14498 | 0.028579 | 3.91E-07 |  | -0.00852862 | 0.0385262 | 0.824803 | 25.7348792 |
| COVID-19 hospitalized | rs111837807 | C | T | 0.09058 | 0.1646 | 0.030882 | 9.82E-08 |  | -0.0238808 | 0.04326 | 0.580927 | 28.40853411 |
| COVID-19 hospitalized | rs112159047 | G | A | 0.1511 | 0.13318 | 0.026513 | 5.08E-07 |  | -0.0196499 | 0.0375608 | 0.600871 | 25.23249974 |
| COVID-19 hospitalized | rs113882976 | C | T | 0.01516 | 0.44574 | 0.095347 | 2.94E-06 |  | -0.101869 | 0.0932005 | 0.274391 | 21.8549165 |
| COVID-19 hospitalized | rs11835149 | T | C | 0.2793 | 0.10308 | 0.021887 | 2.48E-06 |  | -0.0196017 | 0.0259085 | 0.449304 | 22.18075598 |
| COVID-19 hospitalized | rs12477217 | C | A | 0.6101 | -0.088134 | 0.018914 | 3.17E-06 |  | 0.0202936 | 0.0246469 | 0.410295 | 21.71301803 |
| COVID-19 hospitalized | rs12519258 | A | G | 0.2337 | -0.10125 | 0.021819 | 3.47E-06 |  | 0.0389392 | 0.0291324 | 0.181344 | 21.53378533 |
| COVID-19 hospitalized | rs12519801 | T | C | 0.2586 | -0.10848 | 0.023079 | 2.60E-06 |  | -0.00760283 | 0.026754 | 0.776276 | 22.09354362 |
| COVID-19 hospitalized | rs13050728 | C | T | 0.6528 | -0.16832 | 0.020183 | 7.44E-17 |  | 0.00859024 | 0.0254577 | 0.735792 | 69.55045965 |
| COVID-19 hospitalized | rs1381109 | T | G | 0.389 | -0.10276 | 0.02123 | 1.30E-06 |  | 0.0239974 | 0.0249124 | 0.335411 | 23.42870062 |
| COVID-19 hospitalized | rs140228296 | C | A | 0.01971 | 0.36582 | 0.079061 | 3.71E-06 |  | -0.00481799 | 0.0922283 | 0.958338 | 21.40968534 |
| COVID-19 hospitalized | rs17056406 | C | T | 0.05164 | 0.20895 | 0.044242 | 2.32E-06 |  | -0.0161891 | 0.0591875 | 0.784452 | 22.30566873 |
| COVID-19 hospitalized | rs17069506 | C | T | 0.038 | 0.22669 | 0.043387 | 1.74E-07 |  | 0.00301364 | 0.0621219 | 0.961308 | 27.29892097 |
| COVID-19 hospitalized | rs2109069 | A | G | 0.3227 | 0.15131 | 0.019906 | 2.94E-14 |  | -0.0120756 | 0.0263081 | 0.646228 | 57.77863307 |
| COVID-19 hospitalized | rs2660 | A | G | 0.6902 | 0.11639 | 0.019406 | 2.00E-09 |  | 0.0373765 | 0.0254676 | 0.14221 | 35.97156081 |
| COVID-19 hospitalized | rs41264915 | G | A | 0.09259 | -0.16219 | 0.030013 | 6.52E-08 |  | 0.0677022 | 0.0376898 | 0.0724469 | 29.20312525 |
| COVID-19 hospitalized | rs505922 | C | T | 0.3501 | 0.11182 | 0.019056 | 4.42E-09 |  | 0.0129687 | 0.0249811 | 0.603663 | 34.43304955 |
| COVID-19 hospitalized | rs622568 | C | A | 0.1512 | 0.15355 | 0.026026 | 3.64E-09 |  | -0.00328903 | 0.0347383 | 0.924569 | 34.80845848 |
| COVID-19 hospitalized | rs6888703 | G | T | 0.127 | -0.13173 | 0.028357 | 3.40E-06 |  | 0.0436066 | 0.037093 | 0.239754 | 21.57986966 |
| COVID-19 hospitalized | rs78544343 | G | A | 0.01745 | 0.30337 | 0.062979 | 1.46E-06 |  | 0.120907 | 0.0916481 | 0.187085 | 23.20351302 |
| COVID-19 hospitalized | rs79204646 | T | C | 0.01433 | 0.51262 | 0.11173 | 4.47E-06 |  | -0.0318775 | 0.0278132 | 0.251742 | 21.04997067 |
| HPV16 E7 | rs10897761 | C | T | 0.279353 | -0.4405 | 0.0944671 | 3.55E-06 |  | 0.00613371 | 0.0503616 | 0.903063 | 21.74356038 |
| HPV16 E7 | rs7850036 | T | C | 0.359824 | 0.2443 | 0.052959 | 4.49E-06 |  | -0.0185217 | 0.0289459 | 0.522255 | 21.27979217 |
| HPV16 E7 | rs8016986 | T | C | 0.151558 | -0.3348 | 0.0721241 | 3.90E-06 |  | 0.0166234 | 0.0377075 | 0.659321 | 21.54815471 |
| HPV16 E7 | rs9808377 | G | A | 0.492812 | -0.2523 | 0.0527163 | 1.96E-06 |  | -0.0376517 | 0.0285783 | 0.187673 | 22.90575754 |
| Hepatitis-B | rs151282715 | C | T | 0.02516 | -0.6367 | 0.1542 | 3.64E-05 |  | 0.15482 | 0.0748744 | 0.0386653 | 17.04908014 |
| Hepatitis-B | rs72792513 | A | G | 0.01797 | -0.3982 | 0.0798 | 6.04E-07 |  | 0.0240934 | 0.0564978 | 0.669782 | 24.89984988 |
| Hepatitis-B | rs7532552 | A | G | 0.463858 | -0.2049 | 0.0433 | 2.22E-06 |  | 0.0282592 | 0.026032 | 0.277675 | 22.39278571 |
| Hepatitis-B | rs7842195 | A | G | 0.273363 | -0.1876 | 0.0396 | 2.17E-06 |  | -0.0135519 | 0.0249034 | 0.586318 | 22.44270993 |
| Hepatitis-B | rs895767 | A | C | 0.214457 | 0.2765 | 0.0503 | 3.86E-08 |  | 0.023734 | 0.034607 | 0.492831 | 30.21720571 |
| Hepatitis-B | rs9268652 | A | G | 0.191893 | 0.2739 | 0.0473 | 7.01E-09 |  | -0.0259083 | 0.0281336 | 0.357102 | 33.53217956 |
| Viral hepatitis | rs10219333 | T | G | 0.2032 | -0.2517 | 0.0522 | 1.41E-06 |  | 0.0714467 | 0.0320245 | 0.0256809 | 23.25013212 |
| Viral hepatitis | rs111313298 | C | T | 0.06278 | 0.3973 | 0.0869 | 4.86E-06 |  | 0.0531636 | 0.0504913 | 0.292375 | 20.90246848 |
| Viral hepatitis | rs114420497 | A | G | 0.01277 | 0.9969 | 0.2039 | 1.01E-06 |  | 0.0156547 | 0.0939887 | 0.867717 | 23.90389874 |
| Viral hepatitis | rs142982771 | C | T | 0.08752 | 0.3443 | 0.0743 | 3.57E-06 |  | 0.149359 | 0.0579918 | 0.0100089 | 21.47318263 |
| HIV disease | rs13296311 | A | G | 0.125 | -0.5443 | 0.1182 | 4.15E-06 |  | -0.0139888 | 0.0487137 | 0.773988 | 21.2051679 |
| HIV disease | rs1373940 | C | T | 0.7064 | -0.4028 | 0.0842 | 1.70E-06 |  | -0.00836888 | 0.0265659 | 0.752744 | 22.88520151 |
| HIV disease | rs300163 | A | C | 0.2952 | 0.3803 | 0.0833 | 4.97E-06 |  | 0.00393521 | 0.026088 | 0.880099 | 20.84311612 |
| HIV disease | rs34628943 | A | G | 0.006551 | 3.1483 | 0.6031 | 1.79E-07 |  | 0.158702 | 0.0912577 | 0.0820264 | 27.25044268 |
| HIV disease | rs56110037 | T | C | 0.4868 | -0.3638 | 0.0768 | 2.19E-06 |  | -0.0411959 | 0.0269894 | 0.126917 | 22.43897163 |
| HIV disease | rs78849743 | T | C | 0.04526 | 0.948 | 0.1959 | 1.31E-06 |  | -0.0804432 | 0.0816599 | 0.324575 | 23.41789221 |
| HIV disease | rs79856406 | G | T | 0.1019 | 0.5923 | 0.1294 | 4.75E-06 |  | 0.0664925 | 0.0474763 | 0.161352 | 20.95148993 |
| HIV disease | rs929671 | C | A | 0.6931 | -0.4044 | 0.0828 | 1.02E-06 |  | 0.0268206 | 0.026315 | 0.308102 | 23.85402226 |
| Meales-23andMe | rs12700593 | C | T | 0.28115 | 0.0659 | 0.0139 | 2.13E-06 |  | 0.016186 | 0.0239485 | 0.499125 | 22.47714922 |
| Meales-23andMe | rs1353279 | C | T | 0.494808 | -0.0697 | 0.0138 | 4.40E-07 |  | -0.000776341 | 0.0238046 | 0.973983 | 25.50981937 |
| Meales-23andMe | rs4568518 | A | G | 0.384585 | 0.064 | 0.014 | 4.84E-06 |  | -0.0449935 | 0.0245016 | 0.0663061 | 20.89795918 |
| Meales-23andMe | rs500141 | C | T | 0.424321 | -0.073 | 0.0149 | 9.62E-07 |  | -0.0231267 | 0.025869 | 0.371326 | 24.00342327 |
| Meales-23andMe | rs67559144 | C | T | 0.358027 | -0.0768 | 0.0153 | 5.18E-07 |  | -0.0732264 | 0.0268339 | 0.00635507 | 25.1964629 |
| Meales-23andMe | rs9879864 | A | G | 0.256789 | 0.0665 | 0.0139 | 1.72E-06 |  | 0.0383275 | 0.0242118 | 0.113419 | 22.88830806 |
| Meales-FINN | rs1490827 | G | A | 0.5628 | 0.5087 | 0.1074 | 2.19E-06 |  | -0.0466291 | 0.0247177 | 0.0592318 | 22.43442343 |
| Meales-FINN | rs2351647 | T | C | 0.5044 | 0.4957 | 0.1068 | 3.45E-06 |  | 0.00065396 | 0.0238248 | 0.978102 | 21.54246185 |
| Meales-FINN | rs6825765 | C | A | 0.5726 | -0.5933 | 0.1088 | 4.99E-08 |  | 0.0298655 | 0.025239 | 0.236687 | 29.73657227 |
| Meales-FINN | rs875031 | G | A | 0.7938 | 0.6518 | 0.1363 | 1.74E-06 |  | 0.00222268 | 0.0360349 | 0.950817 | 22.86846427 |
| Acute poliomyelitis | rs10110503 | A | G | 0.5451 | 0.4506 | 0.0947 | 1.94E-06 |  | -0.0154099 | 0.024093 | 0.522432 | 22.64031249 |
| Acute poliomyelitis | rs116741188 | T | C | 0.05481 | 1.189 | 0.2305 | 2.48E-07 |  | 0.0265779 | 0.0587333 | 0.650896 | 26.60858927 |
| Acute poliomyelitis | rs2097479 | C | A | 0.9759 | -1.5883 | 0.3474 | 4.83E-06 |  | 0.0148328 | 0.0631338 | 0.814254 | 20.90284709 |
| Rubella-23andMe | rs10208323 | G | T | 0.082867 | 0.2079 | 0.0435 | 1.76E-06 |  | 0.0537263 | 0.0692745 | 0.438011 | 22.84180737 |
| Rubella-23andMe | rs11209896 | A | G | 0.408347 | -0.0747 | 0.0156 | 1.68E-06 |  | 0.0196665 | 0.0240873 | 0.414232 | 22.92936391 |
| Rubella-23andMe | rs2601552 | A | G | 0.297724 | -0.0961 | 0.0209 | 4.26E-06 |  | -0.0188983 | 0.031584 | 0.549606 | 21.14239601 |
| Rubella-23andMe | rs2892940 | A | G | 0.341254 | 0.1082 | 0.0227 | 1.87E-06 |  | 0.0425639 | 0.0341855 | 0.2131 | 22.71971123 |
| Rubella-23andMe | rs66878487 | A | G | 0.022564 | -0.1586 | 0.0318 | 6.12E-07 |  | -0.0107041 | 0.0498898 | 0.830115 | 24.87437206 |
| Rubella-23andMe | rs79031264 | A | G | 0.014776 | 0.1837 | 0.039 | 2.47E-06 |  | 0.117358 | 0.0614468 | 0.0561437 | 22.18651545 |
| Rubella-FINN | rs112429055 | G | A | 0.02597 | 0.9557 | 0.2084 | 4.52E-06 |  | -0.038404 | 0.114991 | 0.738399 | 21.03041015 |
| Rubella-FINN | rs116363196 | C | T | 0.08123 | 0.5931 | 0.1159 | 3.11E-07 |  | 0.0589944 | 0.0574257 | 0.304271 | 26.18719464 |
| Rubella-FINN | rs117318756 | C | T | 0.01124 | 1.6083 | 0.3359 | 1.69E-06 |  | -0.0791264 | 0.109071 | 0.468169 | 22.92525189 |
| Rubella-FINN | rs2269790 | T | C | 0.1969 | 0.3829 | 0.0795 | 1.45E-06 |  | -0.00402339 | 0.0325105 | 0.901508 | 23.19724853 |
| Rubella-FINN | rs57810677 | A | G | 0.473 | 0.2862 | 0.0615 | 3.25E-06 |  | 0.038231 | 0.0246334 | 0.120663 | 21.65653778 |
| Rubella-FINN | rs78555786 | A | G | 0.007881 | 1.8824 | 0.4093 | 4.24E-06 |  | 0.0556476 | 0.106617 | 0.601713 | 21.15145945 |
| Rubella-FINN | rs919965 | T | C | 0.4526 | 0.295 | 0.0613 | 1.48E-06 |  | 0.00514461 | 0.0239196 | 0.829706 | 23.15917492 |
| Rubella-FINN | rs941604 | G | A | 0.6551 | 0.2951 | 0.0646 | 4.84E-06 |  | -0.0252766 | 0.0249379 | 0.310783 | 20.86764227 |

**Additional files 1: Table S2.** Single SNP analysis of the association between viral infection and GBM

|  |  |  |  |  | **Trait** | | |  | **GBM** | | |  |
| --- | --- | --- | --- | --- | --- | --- | --- | --- | --- | --- | --- | --- |
| Exposure | SNP | Effect allele | Other allele | Effect allele frequency | Beta | Standard error | P-value |  | Beta * | Standard error * | P-value * | F-statistics |
| Cold sores | rs115789906 | G | T | 0.009784 | 0.195 | 0.042 | 3.44E-06 |  | 0.00356051 | 0.084509 | 0.966394 | 21.55612245 |
| Cold sores | rs16974161 | A | G | 0.183506 | 0.1825 | 0.0371 | 8.69E-07 |  | -0.0873702 | 0.0667734 | 0.190718 | 24.19791341 |
| Cold sores | rs17732209 | C | T | 0.176318 | 0.0586 | 0.0126 | 3.31E-06 |  | -0.0059694 | 0.0261841 | 0.819663 | 21.62988158 |
| Cold sores | rs73036068 | C | T | 0.009185 | -0.2055 | 0.0398 | 2.43E-07 |  | -0.00828909 | 0.0678795 | 0.902808 | 26.65983814 |
| Cold sores | rs885950 | A | C | 0.417133 | 0.0782 | 0.0109 | 7.27E-13 |  | -0.0238357 | 0.0225667 | 0.290862 | 51.47075162 |
| Herpesviral infections | rs146985296 | C | G | 0.01186 | 0.8077 | 0.1764 | 4.67E-06 |  | 0.0454847 | 0.0861358 | 0.597459 | 20.96539283 |
| Herpesviral infections | rs34264769 | A | C | 0.1454 | 0.2507 | 0.0519 | 1.38E-06 |  | -0.00440753 | 0.0380132 | 0.907694 | 23.33318112 |
| Herpesviral infections | rs4885004 | G | A | 0.2224 | 0.2019 | 0.0434 | 3.23E-06 |  | 0.0188191 | 0.0268035 | 0.48261 | 21.64179001 |
| Herpesviral infections | rs900978 | T | C | 0.4498 | 0.1648 | 0.036 | 4.62E-06 |  | -0.0032704 | 0.0225703 | 0.884791 | 20.95604938 |
| Herpesviral infections | rs9289557 | T | C | 0.1945 | 0.2158 | 0.0457 | 2.37E-06 |  | 0.020403 | 0.0283495 | 0.471712 | 22.29823461 |
| Herpesviral infections | rs9797556 | G | A | 0.3272 | 0.1837 | 0.0381 | 1.41E-06 |  | -0.0373374 | 0.0237599 | 0.116079 | 23.24707738 |
| Herpes zoster | rs10064104 | A | G | 0.3566 | -0.1506 | 0.0329 | 4.76E-06 |  | 0.00594988 | 0.0234636 | 0.799821 | 20.95357582 |
| Herpes zoster | rs10961286 | G | A | 0.1009 | 0.2602 | 0.0532 | 1.02E-06 |  | 0.0554711 | 0.0333824 | 0.0965759 | 23.92166035 |
| Herpes zoster | rs11724363 | A | G | 0.3676 | 0.1543 | 0.0326 | 2.25E-06 |  | 0.0147126 | 0.0232871 | 0.527524 | 22.40250856 |
| Herpes zoster | rs1514878 | G | A | 0.2701 | 0.1728 | 0.0355 | 1.16E-06 |  | 0.0239295 | 0.0236409 | 0.311439 | 23.69358461 |
| Herpes zoster | rs2285414 | G | A | 0.5488 | -0.1498 | 0.0316 | 2.08E-06 |  | -0.0313155 | 0.0226885 | 0.167515 | 22.47240026 |
| Herpes zoster | rs2523580 | G | A | 0.3009 | -0.1637 | 0.0345 | 2.05E-06 |  | -0.00169327 | 0.0243329 | 0.944522 | 22.51433732 |
| Herpes zoster | rs6862129 | G | A | 0.03129 | 0.424 | 0.0927 | 4.80E-06 |  | -0.0345109 | 0.069192 | 0.617942 | 20.92050891 |
| Herpes zoster | rs75043801 | T | G | 0.003317 | 1.5508 | 0.3185 | 1.12E-06 |  | 0.19507 | 0.0970409 | 0.0444123 | 23.70787951 |
| mumps-FINN | rs12881071 | A | G | 0.2841 | 0.3609 | 0.0764 | 2.31E-06 |  | -0.0164539 | 0.0254204 | 0.517457 | 22.31449419 |
| mumps-FINN | rs1857010 | T | C | 0.5574 | 0.3209 | 0.0685 | 2.84E-06 |  | -0.0154567 | 0.0222108 | 0.486484 | 21.94614737 |
| mumps-FINN | rs192016900 | G | T | 0.04488 | 0.8148 | 0.1763 | 3.81E-06 |  | 0.0118078 | 0.0601301 | 0.84432 | 21.35981152 |
| mumps-FINN | rs71488291 | G | A | 0.0153 | 1.5231 | 0.3208 | 2.05E-06 |  | 0.164371 | 0.0568979 | 0.00386628 | 22.54177533 |
| mumps-23andMe | rs1061535 | C | T | 0.141174 | -0.0818 | 0.0161 | 3.76E-07 |  | 0.0411384 | 0.0288958 | 0.154539 | 25.81397323 |
| mumps-23andMe | rs11160318 | A | G | 0.358227 | 0.0947 | 0.0137 | 4.76E-12 |  | 0.0115025 | 0.0242556 | 0.635343 | 47.78139485 |
| mumps-23andMe | rs111709089 | C | T | 0.026158 | -0.1558 | 0.0323 | 1.41E-06 |  | -0.0399012 | 0.0518303 | 0.441393 | 23.26643599 |
| mumps-23andMe | rs114565616 | G | T | 0.005791 | 0.3073 | 0.0667 | 4.08E-06 |  | 0.00531156 | 0.111275 | 0.961928 | 21.22625868 |
| mumps-23andMe | rs13003102 | C | T | 0.013978 | -0.201 | 0.042 | 1.70E-06 |  | 0.0129059 | 0.0665199 | 0.846164 | 22.90306122 |
| mumps-23andMe | rs2139242 | C | T | 0.008387 | 0.3311 | 0.0669 | 7.45E-07 |  | 0.0476024 | 0.105633 | 0.652248 | 24.49436166 |
| mumps-23andMe | rs3862630 | C | T | 0.082468 | 0.1198 | 0.021 | 1.17E-08 |  | 0.051985 | 0.0413157 | 0.208306 | 32.54430839 |
| mumps-23andMe | rs61960129 | A | G | 0.01238 | -0.1797 | 0.0352 | 3.31E-07 |  | -0.0389476 | 0.0599591 | 0.515971 | 26.06218524 |
| mumps-23andMe | rs72683573 | C | T | 0.037141 | -0.1131 | 0.0243 | 3.25E-06 |  | 0.0152099 | 0.0434576 | 0.726344 | 21.66270386 |
| mumps-23andMe | rs7430852 | G | T | 0.047324 | 0.1014 | 0.0218 | 3.30E-06 |  | -0.0146318 | 0.0380642 | 0.700683 | 21.63530006 |
| mumps-23andMe | rs7812934 | A | C | 0.326877 | -0.0894 | 0.0178 | 5.10E-07 |  | 0.0146154 | 0.0313465 | 0.641035 | 25.22522409 |
| mumps-23andMe | rs9461562 | A | G | 0.04253 | -0.2957 | 0.0477 | 5.68E-10 |  | -0.024316 | 0.0749934 | 0.745755 | 38.42960238 |
| Infectious mononucleosis | rs11265380 | A | G | 0.4638 | -0.1977 | 0.0412 | 1.63E-06 |  | -0.017916 | 0.0225484 | 0.426871 | 23.02602154 |
| Infectious mononucleosis | rs112824579 | A | C | 0.1364 | -0.2956 | 0.0613 | 1.42E-06 |  | 0.0370629 | 0.0367011 | 0.312563 | 23.25347754 |
| Infectious mononucleosis | rs116856714 | T | C | 0.01357 | 0.9259 | 0.1957 | 2.22E-06 |  | -0.0384573 | 0.080198 | 0.631562 | 22.38445458 |
| Infectious mononucleosis | rs12599876 | T | C | 0.1287 | 0.2945 | 0.062 | 2.02E-06 |  | 0.0256775 | 0.0358033 | 0.473261 | 22.5625 |
| Infectious mononucleosis | rs145215254 | T | C | 0.009257 | 1.1231 | 0.2388 | 2.57E-06 |  | -0.0161273 | 0.0668663 | 0.80941 | 22.11913858 |
| Infectious mononucleosis | rs17160581 | T | C | 0.09341 | 0.3762 | 0.072 | 1.77E-07 |  | 0.000272561 | 0.038271 | 0.994318 | 27.300625 |
| Infectious mononucleosis | rs1773231 | C | A | 0.8166 | 0.2493 | 0.0535 | 3.17E-06 |  | -0.016802 | 0.0259765 | 0.517751 | 21.71385798 |
| Infectious mononucleosis | rs2518778 | C | T | 0.9754 | -0.6704 | 0.1402 | 1.75E-06 |  | 0.0668467 | 0.0533084 | 0.209856 | 22.86504097 |
| Infectious mononucleosis | rs318491 | G | A | 0.5581 | 0.192 | 0.0419 | 4.56E-06 |  | -0.0443481 | 0.0238626 | 0.0631015 | 20.99782981 |
| Infectious mononucleosis | rs34242269 | T | G | 0.4281 | -0.1943 | 0.0414 | 2.76E-06 |  | -0.00640895 | 0.0234392 | 0.784524 | 22.02647086 |
| Infectious mononucleosis | rs60492066 | C | T | 0.2927 | -0.2144 | 0.0455 | 2.48E-06 |  | 0.025559 | 0.027195 | 0.347299 | 22.20377249 |
| Infectious mononucleosis | rs73171666 | G | T | 0.1796 | -0.2595 | 0.0543 | 1.79E-06 |  | -0.000720672 | 0.0345695 | 0.983368 | 22.83889381 |
| Infectious mononucleosis | rs76330758 | G | A | 0.02938 | 0.6048 | 0.1285 | 2.53E-06 |  | -0.0982975 | 0.0549589 | 0.0736851 | 22.15222274 |
| Infectious mononucleosis | rs76635736 | A | G | 0.008602 | 1.1693 | 0.2451 | 1.84E-06 |  | 0.0753117 | 0.0655254 | 0.250411 | 22.75963416 |
| Mononucleosis | rs2596465 | C | T | 0.449081 | 0.0729 | 0.0122 | 2.30E-09 |  | 0.00320705 | 0.0230153 | 0.889178 | 35.70552271 |
| Mononucleosis | rs2612778 | C | T | 0.371605 | -0.0651 | 0.0136 | 1.69E-06 |  | 0.0172125 | 0.0251566 | 0.493841 | 22.91311635 |
| Mononucleosis | rs7487637 | A | G | 0.10643 | 0.0706 | 0.0144 | 9.45E-07 |  | -0.0130539 | 0.0256232 | 0.610431 | 24.03722994 |
| Cytomegaloviral disease | rs11658622 | A | C | 0.1903 | 0.4911 | 0.1035 | 2.10E-06 |  | -0.001091 | 0.0301494 | 0.971134 | 22.51433732 |
| Cytomegaloviral disease | rs12496650 | C | T | 0.0764 | 1.1619 | 0.2509 | 3.63E-06 |  | -0.0188178 | 0.0527903 | 0.721494 | 21.44550022 |
| Cytomegaloviral disease | rs35435922 | G | A | 0.00639 | 2.7742 | 0.6058 | 4.66E-06 |  | 0.0828507 | 0.0985806 | 0.400664 | 20.9708965 |
| Cytomegaloviral disease | rs66537586 | T | G | 0.008085 | 0.5315 | 0.1139 | 3.08E-06 |  | -0.043831 | 0.0323221 | 0.175077 | 21.77504642 |
| Cytomegaloviral disease | rs6972880 | T | C | 0.9327 | -0.8905 | 0.186 | 1.69E-06 |  | 0.00177518 | 0.057154 | 0.975222 | 22.92144323 |
| Cytomegaloviral disease | rs73087989 | G | A | 0.1942 | 0.5247 | 0.1129 | 3.38E-06 |  | 0.0184393 | 0.0275049 | 0.502603 | 21.59902984 |
| Cytomegaloviral disease | rs787837 | C | T | 0.7603 | -0.4796 | 0.1021 | 2.65E-06 |  | -0.0495023 | 0.0263782 | 0.0605673 | 22.06514901 |
| COVID-19 hospitalized | rs10860891 | A | C | 0.8806 | -0.14498 | 0.028579 | 3.91E-07 |  | -0.014228 | 0.0357881 | 0.690953 | 25.7348792 |
| COVID-19 hospitalized | rs111837807 | C | T | 0.09058 | 0.1646 | 0.030882 | 9.82E-08 |  | -0.00196817 | 0.0406162 | 0.961351 | 28.40853411 |
| COVID-19 hospitalized | rs113882976 | C | T | 0.01516 | 0.44574 | 0.095347 | 2.94E-06 |  | -0.203672 | 0.0856962 | 0.0174695 | 21.8549165 |
| COVID-19 hospitalized | rs11835149 | T | C | 0.2793 | 0.10308 | 0.021887 | 2.48E-06 |  | 0.0333918 | 0.024094 | 0.165779 | 22.18075598 |
| COVID-19 hospitalized | rs12477217 | C | A | 0.6101 | -0.088134 | 0.018914 | 3.17E-06 |  | 0.0181746 | 0.0231024 | 0.431459 | 21.71301803 |
| COVID-19 hospitalized | rs12519258 | A | G | 0.2337 | -0.10125 | 0.021819 | 3.47E-06 |  | 0.00290162 | 0.0270913 | 0.914705 | 21.53378533 |
| COVID-19 hospitalized | rs12519801 | T | C | 0.2586 | -0.10848 | 0.023079 | 2.60E-06 |  | 0.0125663 | 0.0249101 | 0.613935 | 22.09354362 |
| COVID-19 hospitalized | rs13050728 | C | T | 0.6528 | -0.16832 | 0.020183 | 7.44E-17 |  | -0.077735 | 0.0235933 | 0.000984951 | 69.55045965 |
| COVID-19 hospitalized | rs1381109 | T | G | 0.389 | -0.10276 | 0.02123 | 1.30E-06 |  | 0.00393403 | 0.0231491 | 0.865055 | 23.42870062 |
| COVID-19 hospitalized | rs140228296 | C | A | 0.01971 | 0.36582 | 0.079061 | 3.71E-06 |  | -0.0641278 | 0.085955 | 0.45563 | 21.40968534 |
| COVID-19 hospitalized | rs17056406 | C | T | 0.05164 | 0.20895 | 0.044242 | 2.32E-06 |  | -0.00450223 | 0.0555433 | 0.935396 | 22.30566873 |
| COVID-19 hospitalized | rs17069506 | C | T | 0.038 | 0.22669 | 0.043387 | 1.74E-07 |  | -0.0636605 | 0.058984 | 0.280461 | 27.29892097 |
| COVID-19 hospitalized | rs2109069 | A | G | 0.3227 | 0.15131 | 0.019906 | 2.94E-14 |  | 0.0264053 | 0.0247453 | 0.285933 | 57.77863307 |
| COVID-19 hospitalized | rs2660 | A | G | 0.6902 | 0.11639 | 0.019406 | 2.00E-09 |  | 0.0196833 | 0.0236875 | 0.405998 | 35.97156081 |
| COVID-19 hospitalized | rs41264915 | G | A | 0.09259 | -0.16219 | 0.030013 | 6.52E-08 |  | -0.0320754 | 0.0357369 | 0.369429 | 29.20312525 |
| COVID-19 hospitalized | rs4310517 | G | C | 0.7553 | -0.10956 | 0.022603 | 1.25E-06 |  | 0.0374999 | 0.025329 | 0.138736 | 23.49480674 |
| COVID-19 hospitalized | rs505922 | C | T | 0.3501 | 0.11182 | 0.019056 | 4.42E-09 |  | -0.0528128 | 0.0234683 | 0.0244245 | 34.43304955 |
| COVID-19 hospitalized | rs622568 | C | A | 0.1512 | 0.15355 | 0.026026 | 3.64E-09 |  | 0.0208365 | 0.0320423 | 0.515512 | 34.80845848 |
| COVID-19 hospitalized | rs6888703 | G | T | 0.127 | -0.13173 | 0.028357 | 3.40E-06 |  | -0.00561536 | 0.0348885 | 0.872131 | 21.57986966 |
| COVID-19 hospitalized | rs78544343 | G | A | 0.01745 | 0.30337 | 0.062979 | 1.46E-06 |  | 0.0150249 | 0.0868299 | 0.862621 | 23.20351302 |
| COVID-19 hospitalized | rs7953236 | C | T | 0.2321 | -0.096631 | 0.021117 | 4.74E-06 |  | -0.0310042 | 0.0259444 | 0.232076 | 20.93960611 |
| COVID-19 hospitalized | rs8096865 | T | C | 0.03442 | 0.24727 | 0.053853 | 4.40E-06 |  | 0.0345903 | 0.0766271 | 0.651694 | 21.08254589 |
| HPV16 E7 | rs10897761 | C | T | 0.279353 | -0.4405 | 0.0944671 | 3.55E-06 |  | -0.0199065 | 0.0471897 | 0.673141 | 21.74356038 |
| HPV16 E7 | rs7850036 | T | C | 0.359824 | 0.2443 | 0.052959 | 4.49E-06 |  | -0.0269183 | 0.0269645 | 0.31814 | 21.27979217 |
| HPV16 E7 | rs8016986 | T | C | 0.151558 | -0.3348 | 0.0721241 | 3.90E-06 |  | -0.0131805 | 0.0352113 | 0.708161 | 21.54815471 |
| HPV16 E7 | rs9808377 | G | A | 0.492812 | -0.2523 | 0.0527163 | 1.96E-06 |  | -0.00793158 | 0.0264923 | 0.764641 | 22.90575754 |
| Hepatitis-B | rs151282715 | C | T | 0.02516 | -0.6367 | 0.1542 | 3.64E-05 |  | 0.0990062 | 0.0703635 | 0.159408 | 17.04908014 |
| Hepatitis-B | rs72792513 | A | G | 0.01797 | -0.3982 | 0.0798 | 6.04E-07 |  | 0.0167696 | 0.0527548 | 0.750577 | 24.89984988 |
| Hepatitis-B | rs7532552 | A | G | 0.463858 | -0.2049 | 0.0433 | 2.22E-06 |  | -0.0156629 | 0.0244195 | 0.521256 | 22.39278571 |
| Hepatitis-B | rs7842195 | A | G | 0.273363 | -0.1876 | 0.0396 | 2.17E-06 |  | -0.00932416 | 0.0232188 | 0.687994 | 22.44270993 |
| Hepatitis-B | rs895767 | A | C | 0.214457 | 0.2765 | 0.0503 | 3.86E-08 |  | 0.00026751 | 0.0324794 | 0.993428 | 30.21720571 |
| Hepatitis-B | rs9268652 | A | G | 0.191893 | 0.2739 | 0.0473 | 7.01E-09 |  | 0.0261901 | 0.0263688 | 0.320602 | 33.53217956 |
| Viral hepatitis | rs10219333 | T | G | 0.2032 | -0.2517 | 0.0522 | 1.41E-06 |  | 0.0253137 | 0.0298957 | 0.397144 | 23.25013212 |
| Viral hepatitis | rs111313298 | C | T | 0.06278 | 0.3973 | 0.0869 | 4.86E-06 |  | 0.0748919 | 0.046728 | 0.108997 | 20.90246848 |
| Viral hepatitis | rs114420497 | A | G | 0.01277 | 0.9969 | 0.2039 | 1.01E-06 |  | 0.11704 | 0.0852374 | 0.16972 | 23.90389874 |
| Viral hepatitis | rs142982771 | C | T | 0.08752 | 0.3443 | 0.0743 | 3.57E-06 |  | 0.0640197 | 0.0543354 | 0.238704 | 21.47318263 |
| HIV disease | rs13296311 | A | G | 0.125 | -0.5443 | 0.1182 | 4.15E-06 |  | 0.000675229 | 0.0453657 | 0.988125 | 21.2051679 |
| HIV disease | rs1373940 | C | T | 0.7064 | -0.4028 | 0.0842 | 1.70E-06 |  | 0.0244349 | 0.0247132 | 0.322792 | 22.88520151 |
| HIV disease | rs300163 | A | C | 0.2952 | 0.3803 | 0.0833 | 4.97E-06 |  | -0.00302865 | 0.0244225 | 0.901307 | 20.84311612 |
| HIV disease | rs34628943 | A | G | 0.006551 | 3.1483 | 0.6031 | 1.79E-07 |  | -0.0379827 | 0.0892472 | 0.670407 | 27.25044268 |
| HIV disease | rs56110037 | T | C | 0.4868 | -0.3638 | 0.0768 | 2.19E-06 |  | -0.041649 | 0.0250119 | 0.0958791 | 22.43897163 |
| HIV disease | rs78849743 | T | C | 0.04526 | 0.948 | 0.1959 | 1.31E-06 |  | 0.0951966 | 0.0768837 | 0.215646 | 23.41789221 |
| HIV disease | rs79856406 | G | T | 0.1019 | 0.5923 | 0.1294 | 4.75E-06 |  | 0.0032266 | 0.0449791 | 0.942812 | 20.95148993 |
| HIV disease | rs929671 | C | A | 0.6931 | -0.4044 | 0.0828 | 1.02E-06 |  | 0.0125823 | 0.0244811 | 0.607279 | 23.85402226 |
| Meales-23andMe | rs12700593 | C | T | 0.28115 | 0.0659 | 0.0139 | 2.13E-06 |  | -0.0160594 | 0.0223351 | 0.472128 | 22.47714922 |
| Meales-23andMe | rs1353279 | C | T | 0.494808 | -0.0697 | 0.0138 | 4.40E-07 |  | -0.0104703 | 0.0221981 | 0.637159 | 25.50981937 |
| Meales-23andMe | rs4568518 | A | G | 0.384585 | 0.064 | 0.014 | 4.84E-06 |  | -0.0135555 | 0.022802 | 0.552187 | 20.89795918 |
| Meales-23andMe | rs500141 | C | T | 0.424321 | -0.073 | 0.0149 | 9.62E-07 |  | -0.00568719 | 0.0241761 | 0.814022 | 24.00342327 |
| Meales-23andMe | rs67559144 | C | T | 0.358027 | -0.0768 | 0.0153 | 5.18E-07 |  | -0.000787251 | 0.0249153 | 0.974793 | 25.1964629 |
| Meales-23andMe | rs9879864 | A | G | 0.256789 | 0.0665 | 0.0139 | 1.72E-06 |  | 0.0114909 | 0.0225747 | 0.610741 | 22.88830806 |
| Meales-FINN | rs1490827 | G | A | 0.5628 | 0.5087 | 0.1074 | 2.19E-06 |  | -0.0470227 | 0.0229653 | 0.0406036 | 22.43442343 |
| Meales-FINN | rs2351647 | T | C | 0.5044 | 0.4957 | 0.1068 | 3.45E-06 |  | -0.03515 | 0.0224664 | 0.117687 | 21.54246185 |
| Meales-FINN | rs6825765 | C | A | 0.5726 | -0.5933 | 0.1088 | 4.99E-08 |  | 0.0184077 | 0.0235742 | 0.434895 | 29.73657227 |
| Meales-FINN | rs875031 | G | A | 0.7938 | 0.6518 | 0.1363 | 1.74E-06 |  | 0.00763319 | 0.033688 | 0.820747 | 22.86846427 |
| Acute poliomyelitis | rs10110503 | A | G | 0.5451 | 0.4506 | 0.0947 | 1.94E-06 |  | -0.0155169 | 0.0224578 | 0.489606 | 22.64031249 |
| Acute poliomyelitis | rs116741188 | T | C | 0.05481 | 1.189 | 0.2305 | 2.48E-07 |  | 0.0626856 | 0.0532036 | 0.238708 | 26.60858927 |
| Acute poliomyelitis | rs2097479 | C | A | 0.9759 | -1.5883 | 0.3474 | 4.83E-06 |  | -0.00217355 | 0.0592034 | 0.970714 | 20.90284709 |
| Rubella-23andMe | rs10208323 | G | T | 0.082867 | 0.2079 | 0.0435 | 1.76E-06 |  | -0.0071646 | 0.0641978 | 0.911139 | 22.84180737 |
| Rubella-23andMe | rs11209896 | A | G | 0.408347 | -0.0747 | 0.0156 | 1.68E-06 |  | 0.0398201 | 0.0224582 | 0.0762152 | 22.92936391 |
| Rubella-23andMe | rs149417607 | A | G | 0.007588 | -0.3591 | 0.0786 | 4.91E-06 |  | -0.0171176 | 0.103031 | 0.868047 | 20.87304062 |
| Rubella-23andMe | rs2601552 | A | G | 0.297724 | -0.0961 | 0.0209 | 4.26E-06 |  | 0.0129562 | 0.0292145 | 0.657415 | 21.14239601 |
| Rubella-23andMe | rs2892940 | A | G | 0.341254 | 0.1082 | 0.0227 | 1.87E-06 |  | 0.0363241 | 0.0317427 | 0.252487 | 22.71971123 |
| Rubella-23andMe | rs66878487 | A | G | 0.022564 | -0.1586 | 0.0318 | 6.12E-07 |  | 0.0179212 | 0.0466478 | 0.700844 | 24.87437206 |
| Rubella-23andMe | rs79031264 | A | G | 0.014776 | 0.1837 | 0.039 | 2.47E-06 |  | -0.00736812 | 0.0579175 | 0.898768 | 22.18651545 |
| Rubella-FINN | rs112429055 | G | A | 0.02597 | 0.9557 | 0.2084 | 4.52E-06 |  | 0.140496 | 0.10843 | 0.195067 | 21.03041015 |
| Rubella-FINN | rs116363196 | C | T | 0.08123 | 0.5931 | 0.1159 | 3.11E-07 |  | 0.120727 | 0.0540123 | 0.0254061 | 26.18719464 |
| Rubella-FINN | rs117318756 | C | T | 0.01124 | 1.6083 | 0.3359 | 1.69E-06 |  | -0.0799649 | 0.104261 | 0.443098 | 22.92525189 |
| Rubella-FINN | rs2269790 | T | C | 0.1969 | 0.3829 | 0.0795 | 1.45E-06 |  | 0.0457205 | 0.0298531 | 0.125641 | 23.19724853 |
| Rubella-FINN | rs57810677 | A | G | 0.473 | 0.2862 | 0.0615 | 3.25E-06 |  | -0.00892882 | 0.0229587 | 0.697344 | 21.65653778 |
| Rubella-FINN | rs78555786 | A | G | 0.007881 | 1.8824 | 0.4093 | 4.24E-06 |  | -0.0412847 | 0.0989346 | 0.676464 | 21.15145945 |
| Rubella-FINN | rs919965 | T | C | 0.4526 | 0.295 | 0.0613 | 1.48E-06 |  | 0.0267357 | 0.0224293 | 0.233262 | 23.15917492 |
| Rubella-FINN | rs941604 | G | A | 0.6551 | 0.2951 | 0.0646 | 4.84E-06 |  | -0.0156762 | 0.0235581 | 0.505777 | 20.86764227 |

**Additional files 1: Table S3.** Single SNP analysis of the association between viral infection and all-glioma

|  |  |  |  |  | **Trait** | | |  | **All-glioma** | | |  |
| --- | --- | --- | --- | --- | --- | --- | --- | --- | --- | --- | --- | --- |
| Exposure | SNP | Effect allele | Other allele | Effect allele frequency | Beta | Standard error | P-value |  | Beta * | Standard error * | P-value * | F-statistics |
| Cold sores | rs115789906 | G | T | 0.009784 | 0.195 | 0.042 | 3.44E-06 |  | 0.0547919 | 0.0681616 | 0.421482 | 21.55612245 |
| Cold sores | rs16974161 | A | G | 0.183506 | 0.1825 | 0.0371 | 8.69E-07 |  | -0.0653761 | 0.054812 | 0.232973 | 24.19791341 |
| Cold sores | rs17732209 | C | T | 0.176318 | 0.0586 | 0.0126 | 3.31E-06 |  | -0.0147146 | 0.0213996 | 0.491696 | 21.62988158 |
| Cold sores | rs73036068 | C | T | 0.009185 | -0.2055 | 0.0398 | 2.43E-07 |  | 0.00818747 | 0.0551737 | 0.882032 | 26.65983814 |
| Cold sores | rs885950 | A | C | 0.417133 | 0.0782 | 0.0109 | 7.27E-13 |  | -0.0365944 | 0.0184719 | 0.0475819 | 51.47075162 |
| Herpesviral infections | rs12550275 | T | C | 0.1376 | 0.2403 | 0.0526 | 4.84E-06 |  | 0.000671006 | 0.026766 | 0.98 | 20.87065376 |
| Herpesviral infections | rs34264769 | A | C | 0.1454 | 0.2507 | 0.0519 | 1.38E-06 |  | 0.0222667 | 0.0308575 | 0.470542 | 23.33318112 |
| Herpesviral infections | rs4885004 | G | A | 0.2224 | 0.2019 | 0.0434 | 3.23E-06 |  | 0.0284901 | 0.0216475 | 0.188144 | 21.64179001 |
| Herpesviral infections | rs900978 | T | C | 0.4498 | 0.1648 | 0.036 | 4.62E-06 |  | -0.00972779 | 0.0183813 | 0.596651 | 20.95604938 |
| Herpesviral infections | rs9289557 | T | C | 0.1945 | 0.2158 | 0.0457 | 2.37E-06 |  | 0.032564 | 0.0231076 | 0.158766 | 22.29823461 |
| Herpesviral infections | rs9797556 | G | A | 0.3272 | 0.1837 | 0.0381 | 1.41E-06 |  | -0.00143814 | 0.0193361 | 0.940711 | 23.24707738 |
| Herpes zoster | rs10064104 | A | G | 0.3566 | -0.1506 | 0.0329 | 4.76E-06 |  | 0.00282243 | 0.0191022 | 0.882537 | 20.95357582 |
| Herpes zoster | rs10961286 | G | A | 0.1009 | 0.2602 | 0.0532 | 1.02E-06 |  | 0.00697672 | 0.0272476 | 0.797913 | 23.92166035 |
| Herpes zoster | rs11724363 | A | G | 0.3676 | 0.1543 | 0.0326 | 2.25E-06 |  | -0.00105135 | 0.0189814 | 0.955829 | 22.40250856 |
| Herpes zoster | rs1514878 | G | A | 0.2701 | 0.1728 | 0.0355 | 1.16E-06 |  | -0.0115289 | 0.0193062 | 0.5504 | 23.69358461 |
| Herpes zoster | rs2285414 | G | A | 0.5488 | -0.1498 | 0.0316 | 2.08E-06 |  | 0.000743559 | 0.018496 | 0.967933 | 22.47240026 |
| Herpes zoster | rs2523580 | G | A | 0.3009 | -0.1637 | 0.0345 | 2.05E-06 |  | 0.0120702 | 0.0199721 | 0.54561 | 22.51433732 |
| Herpes zoster | rs6862129 | G | A | 0.03129 | 0.424 | 0.0927 | 4.80E-06 |  | -0.0458292 | 0.0561987 | 0.414795 | 20.92050891 |
| Herpes zoster | rs75043801 | T | G | 0.003317 | 1.5508 | 0.3185 | 1.12E-06 |  | 0.116441 | 0.0791972 | 0.141488 | 23.70787951 |
| mumps-FINN | rs12881071 | A | G | 0.2841 | 0.3609 | 0.0764 | 2.31E-06 |  | 0.0467402 | 0.0271803 | 0.0854984 | 22.31449419 |
| mumps-FINN | rs1857010 | T | C | 0.5574 | 0.3209 | 0.0685 | 2.84E-06 |  | -0.0056159 | 0.0238966 | 0.814202 | 21.94614737 |
| mumps-FINN | rs192016900 | G | T | 0.04488 | 0.8148 | 0.1763 | 3.81E-06 |  | 0.05123 | 0.0654541 | 0.433812 | 21.35981152 |
| mumps-FINN | rs71488291 | G | A | 0.0153 | 1.5231 | 0.3208 | 2.05E-06 |  | 0.0943941 | 0.060591 | 0.11926 | 22.54177533 |
| mumps-23andMe | rs1061535 | C | T | 0.141174 | -0.0818 | 0.0161 | 3.76E-07 |  | 0.0161853 | 0.0234436 | 0.489948 | 25.81397323 |
| mumps-23andMe | rs11160318 | A | G | 0.358227 | 0.0947 | 0.0137 | 4.76E-12 |  | -0.00737536 | 0.019785 | 0.709315 | 47.78139485 |
| mumps-23andMe | rs111709089 | C | T | 0.026158 | -0.1558 | 0.0323 | 1.41E-06 |  | -0.0668634 | 0.0428107 | 0.118326 | 23.26643599 |
| mumps-23andMe | rs114565616 | G | T | 0.005791 | 0.3073 | 0.0667 | 4.08E-06 |  | 0.0704678 | 0.0886433 | 0.426637 | 21.22625868 |
| mumps-23andMe | rs13003102 | C | T | 0.013978 | -0.201 | 0.042 | 1.70E-06 |  | 0.0144173 | 0.0537128 | 0.78838 | 22.90306122 |
| mumps-23andMe | rs2139242 | C | T | 0.008387 | 0.3311 | 0.0669 | 7.45E-07 |  | 0.0365869 | 0.0860968 | 0.670873 | 24.49436166 |
| mumps-23andMe | rs3862630 | C | T | 0.082468 | 0.1198 | 0.021 | 1.17E-08 |  | 0.0271532 | 0.0338198 | 0.422045 | 32.54430839 |
| mumps-23andMe | rs61960129 | A | G | 0.01238 | -0.1797 | 0.0352 | 3.31E-07 |  | -0.0266551 | 0.048655 | 0.583802 | 26.06218524 |
| mumps-23andMe | rs72683573 | C | T | 0.037141 | -0.1131 | 0.0243 | 3.25E-06 |  | 0.0185038 | 0.0353992 | 0.601171 | 21.66270386 |
| mumps-23andMe | rs7430852 | G | T | 0.047324 | 0.1014 | 0.0218 | 3.30E-06 |  | -0.00390867 | 0.0310741 | 0.899902 | 21.63530006 |
| mumps-23andMe | rs7812934 | A | C | 0.326877 | -0.0894 | 0.0178 | 5.10E-07 |  | 0.00952243 | 0.0255794 | 0.709692 | 25.22522409 |
| mumps-23andMe | rs9461562 | A | G | 0.04253 | -0.2957 | 0.0477 | 5.68E-10 |  | -0.0416847 | 0.0616299 | 0.498805 | 38.42960238 |
| Infectious mononucleosis | rs11265380 | A | G | 0.4638 | -0.1977 | 0.0412 | 1.63E-06 |  | rs11265380 | -0.0248173 | 0.0183644 | 23.02602154 |
| Infectious mononucleosis | rs112824579 | A | C | 0.1364 | -0.2956 | 0.0613 | 1.42E-06 |  | rs112824579 | 0.0330127 | 0.029265 | 23.25347754 |
| Infectious mononucleosis | rs116856714 | T | C | 0.01357 | 0.9259 | 0.1957 | 2.22E-06 |  | rs116856714 | -0.000667347 | 0.0647944 | 22.38445458 |
| Infectious mononucleosis | rs12599876 | T | C | 0.1287 | 0.2945 | 0.062 | 2.02E-06 |  | rs12599876 | 0.0288917 | 0.0292045 | 22.5625 |
| Infectious mononucleosis | rs145215254 | T | C | 0.009257 | 1.1231 | 0.2388 | 2.57E-06 |  | rs145215254 | -0.00129158 | 0.053887 | 22.11913858 |
| Infectious mononucleosis | rs17160581 | T | C | 0.09341 | 0.3762 | 0.072 | 1.77E-07 |  | rs17160581 | 0.0186372 | 0.0313523 | 27.300625 |
| Infectious mononucleosis | rs1773231 | C | A | 0.8166 | 0.2493 | 0.0535 | 3.17E-06 |  | rs1773231 | -0.0162969 | 0.0212176 | 21.71385798 |
| Infectious mononucleosis | rs2518778 | C | T | 0.9754 | -0.6704 | 0.1402 | 1.75E-06 |  | rs2518778 | 0.0405726 | 0.0435456 | 22.86504097 |
| Infectious mononucleosis | rs318491 | G | A | 0.5581 | 0.192 | 0.0419 | 4.56E-06 |  | rs318491 | -0.0375829 | 0.0193794 | 20.99782981 |
| Infectious mononucleosis | rs34242269 | T | G | 0.4281 | -0.1943 | 0.0414 | 2.76E-06 |  | rs34242269 | -0.00151929 | 0.0191505 | 22.02647086 |
| Infectious mononucleosis | rs60492066 | C | T | 0.2927 | -0.2144 | 0.0455 | 2.48E-06 |  | rs60492066 | 0.00518513 | 0.0222835 | 22.20377249 |
| Infectious mononucleosis | rs73171666 | G | T | 0.1796 | -0.2595 | 0.0543 | 1.79E-06 |  | rs73171666 | -0.0232377 | 0.0282923 | 22.83889381 |
| Infectious mononucleosis | rs76330758 | G | A | 0.02938 | 0.6048 | 0.1285 | 2.53E-06 |  | rs76330758 | -0.0947645 | 0.0450226 | 22.15222274 |
| Infectious mononucleosis | rs76635736 | A | G | 0.008602 | 1.1693 | 0.2451 | 1.84E-06 |  | rs76635736 | 0.0569691 | 0.0532484 | 22.75963416 |
| Mononucleosis | rs2596465 | C | T | 0.449081 | 0.0729 | 0.0122 | 2.30E-09 |  | -0.0235578 | 0.018735 | 0.208601 | 35.70552271 |
| Mononucleosis | rs2612778 | C | T | 0.371605 | -0.0651 | 0.0136 | 1.69E-06 |  | 0.00308096 | 0.0206017 | 0.881121 | 22.91311635 |
| Mononucleosis | rs7487637 | A | G | 0.10643 | 0.0706 | 0.0144 | 9.45E-07 |  | -0.027121 | 0.0209707 | 0.195913 | 24.03722994 |
| Cytomegaloviral disease | rs11658622 | A | C | 0.1903 | 0.5247 | 0.1129 | 3.38E-06 |  | -0.0131435 | 0.024591 | 0.593006 | 21.59902984 |
| Cytomegaloviral disease | rs12496650 | C | T | 0.2714 | -0.4796 | 0.1021 | 2.65E-06 |  | -0.0162669 | 0.0431634 | 0.706272 | 22.06514901 |
| Cytomegaloviral disease | rs35435922 | G | A | 0.03762 | 1.1619 | 0.2509 | 3.63E-06 |  | 0.0618089 | 0.0790111 | 0.434049 | 21.44550022 |
| Cytomegaloviral disease | rs66537586 | T | G | 0.008085 | 2.7742 | 0.6058 | 4.66E-06 |  | -0.0241054 | 0.0264122 | 0.361422 | 20.9708965 |
| Cytomegaloviral disease | rs6972880 | T | C | 0.9327 | -0.8905 | 0.186 | 1.69E-06 |  | 0.0159814 | 0.0468744 | 0.733147 | 22.92144323 |
| Cytomegaloviral disease | rs73087989 | G | A | 0.1942 | 0.5315 | 0.1139 | 3.08E-06 |  | 0.0198197 | 0.0224382 | 0.377074 | 21.77504642 |
| Cytomegaloviral disease | rs787837 | C | T | 0.7603 | 0.4911 | 0.1035 | 2.10E-06 |  | -0.0380214 | 0.0214433 | 0.0762095 | 22.51433732 |
| COVID-19 hospitalized | rs111837807 | C | T | 0.09058 | 0.1646 | 0.030882 | 9.82E-08 |  | -0.0101582 | 0.0330725 | 0.75873 | 28.40853411 |
| COVID-19 hospitalized | rs113882976 | C | T | 0.01516 | 0.44574 | 0.095347 | 2.94E-06 |  | -0.179067 | 0.0713446 | 0.012077 | 21.8549165 |
| COVID-19 hospitalized | rs11835149 | T | C | 0.2793 | 0.10308 | 0.021887 | 2.48E-06 |  | 0.00734678 | 0.0196711 | 0.708791 | 22.18075598 |
| COVID-19 hospitalized | rs12477217 | C | A | 0.6101 | -0.088134 | 0.018914 | 3.17E-06 |  | 0.0201582 | 0.0187889 | 0.283326 | 21.71301803 |
| COVID-19 hospitalized | rs12519258 | A | G | 0.2337 | -0.10125 | 0.021819 | 3.47E-06 |  | 0.0100532 | 0.0220967 | 0.649136 | 21.53378533 |
| COVID-19 hospitalized | rs12519801 | T | C | 0.2586 | -0.10848 | 0.023079 | 2.60E-06 |  | -0.000780506 | 0.0203049 | 0.969337 | 22.09354362 |
| COVID-19 hospitalized | rs13050728 | C | T | 0.6528 | -0.16832 | 0.020183 | 7.44E-17 |  | -0.0427084 | 0.0192906 | 0.0268326 | 69.55045965 |
| COVID-19 hospitalized | rs1381109 | T | G | 0.389 | -0.10276 | 0.02123 | 1.30E-06 |  | 0.000752257 | 0.0189274 | 0.968297 | 23.42870062 |
| COVID-19 hospitalized | rs140228296 | C | A | 0.01971 | 0.36582 | 0.079061 | 3.71E-06 |  | -0.0430505 | 0.0699337 | 0.538166 | 21.40968534 |
| COVID-19 hospitalized | rs17056406 | C | T | 0.05164 | 0.20895 | 0.044242 | 2.32E-06 |  | -0.0105667 | 0.0452651 | 0.81542 | 22.30566873 |
| COVID-19 hospitalized | rs17069506 | C | T | 0.038 | 0.22669 | 0.043387 | 1.74E-07 |  | -0.0238996 | 0.0477112 | 0.616426 | 27.29892097 |
| COVID-19 hospitalized | rs2109069 | A | G | 0.3227 | 0.15131 | 0.019906 | 2.94E-14 |  | 2.61798E-05 | 0.0201276 | 0.998962 | 57.77863307 |
| COVID-19 hospitalized | rs2660 | A | G | 0.6902 | 0.11639 | 0.019406 | 2.00E-09 |  | 0.0222897 | 0.0193663 | 0.24975 | 35.97156081 |
| COVID-19 hospitalized | rs35081325 | T | A | 0.08122 | 0.48825 | 0.031508 | 3.68E-54 |  | -0.00492629 | 0.0343417 | 0.885935 | 240.1280147 |
| COVID-19 hospitalized | rs41264915 | G | A | 0.09259 | -0.16219 | 0.030013 | 6.52E-08 |  | 0.0137859 | 0.0288525 | 0.632788 | 29.20312525 |
| COVID-19 hospitalized | rs4310517 | G | C | 0.7553 | -0.10956 | 0.022603 | 1.25E-06 |  | 0.0314454 | 0.0206677 | 0.128141 | 23.49480674 |
| COVID-19 hospitalized | rs505922 | C | T | 0.3501 | 0.11182 | 0.019056 | 4.42E-09 |  | -0.0196352 | 0.0190678 | 0.303123 | 34.43304955 |
| COVID-19 hospitalized | rs622568 | C | A | 0.1512 | 0.15355 | 0.026026 | 3.64E-09 |  | 0.0105664 | 0.0262334 | 0.687108 | 34.80845848 |
| COVID-19 hospitalized | rs6888703 | G | T | 0.127 | -0.13173 | 0.028357 | 3.40E-06 |  | 0.0144887 | 0.0282572 | 0.60813 | 21.57986966 |
| COVID-19 hospitalized | rs78544343 | G | A | 0.01745 | 0.30337 | 0.062979 | 1.46E-06 |  | 0.0390695 | 0.06979 | 0.575606 | 23.20351302 |
| COVID-19 hospitalized | rs7953236 | C | T | 0.2321 | -0.096631 | 0.021117 | 4.74E-06 |  | -0.0349595 | 0.0211607 | 0.0985145 | 20.93960611 |
| HPV16 E7 | rs10897761 | C | T | 0.279353 | -0.4405 | 0.0944671 | 3.55E-06 |  | 0.0103608 | 0.0392286 | 0.791693 | 21.74356038 |
| HPV16 E7 | rs7850036 | T | C | 0.359824 | 0.2443 | 0.052959 | 4.49E-06 |  | -0.0300598 | 0.0220079 | 0.171981 | 21.27979217 |
| HPV16 E7 | rs8016986 | T | C | 0.151558 | -0.3348 | 0.0721241 | 3.90E-06 |  | -0.00939309 | 0.028631 | 0.742855 | 21.54815471 |
| HPV16 E7 | rs9808377 | G | A | 0.492812 | -0.2523 | 0.0527163 | 1.96E-06 |  | -0.0196719 | 0.0216965 | 0.364574 | 22.90575754 |
| Hepatitis-B | rs151282715 | C | T | 0.02516 | -0.6367 | 0.1542 | 3.64E-05 |  | 0.12075 | 0.0564534 | 0.0324411 | 17.04908014 |
| Hepatitis-B | rs72792513 | A | G | 0.01797 | -0.3982 | 0.0798 | 6.04E-07 |  | 0.0192681 | 0.0427515 | 0.652206 | 24.89984988 |
| Hepatitis-B | rs7532552 | A | G | 0.463858 | -0.2049 | 0.0433 | 2.22E-06 |  | 0.00309271 | 0.0198676 | 0.876296 | 22.39278571 |
| Hepatitis-B | rs7842195 | A | G | 0.273363 | -0.1876 | 0.0396 | 2.17E-06 |  | -0.0166835 | 0.0188851 | 0.377007 | 22.44270993 |
| Hepatitis-B | rs895767 | A | C | 0.214457 | 0.2765 | 0.0503 | 3.86E-08 |  | 0.00229315 | 0.0264891 | 0.931014 | 30.21720571 |
| Hepatitis-B | rs9268652 | A | G | 0.191893 | 0.2739 | 0.0473 | 7.01E-09 |  | -0.00289934 | 0.021458 | 0.89252 | 33.53217956 |
| Viral hepatitis | rs10219333 | T | G | 0.2032 | -0.2517 | 0.0522 | 1.41E-06 |  | 0.0415996 | 0.0247376 | 0.0926398 | 23.25013212 |
| Viral hepatitis | rs111313298 | C | T | 0.06278 | 0.3973 | 0.0869 | 4.86E-06 |  | 0.054039 | 0.038148 | 0.156611 | 20.90246848 |
| Viral hepatitis | rs114420497 | A | G | 0.01277 | 0.9969 | 0.2039 | 1.01E-06 |  | 0.0505477 | 0.0702797 | 0.471995 | 23.90389874 |
| Viral hepatitis | rs142982771 | C | T | 0.08752 | 0.3443 | 0.0743 | 3.57E-06 |  | 0.104481 | 0.044123 | 0.0178874 | 21.47318263 |
| HIV disease | rs13296311 | A | G | 0.125 | -0.5443 | 0.1182 | 4.15E-06 |  | -0.00876371 | 0.0368562 | 0.812051 | 21.2051679 |
| HIV disease | rs1373940 | C | T | 0.7064 | -0.4028 | 0.0842 | 1.70E-06 |  | 0.00728019 | 0.0201543 | 0.717934 | 22.88520151 |
| HIV disease | rs300163 | A | C | 0.2952 | 0.3803 | 0.0833 | 4.97E-06 |  | 0.00587568 | 0.0198618 | 0.767361 | 20.84311612 |
| HIV disease | rs34628943 | A | G | 0.006551 | 3.1483 | 0.6031 | 1.79E-07 |  | 0.049168 | 0.0704351 | 0.485139 | 27.25044268 |
| HIV disease | rs56110037 | T | C | 0.4868 | -0.3638 | 0.0768 | 2.19E-06 |  | -0.0455339 | 0.0204541 | 0.0260042 | 22.43897163 |
| HIV disease | rs78849743 | T | C | 0.04526 | 0.948 | 0.1959 | 1.31E-06 |  | 0.0208661 | 0.0617094 | 0.735262 | 23.41789221 |
| HIV disease | rs79856406 | G | T | 0.1019 | 0.5923 | 0.1294 | 4.75E-06 |  | 0.0384782 | 0.0363595 | 0.289932 | 20.95148993 |
| HIV disease | rs929671 | C | A | 0.6931 | -0.4044 | 0.0828 | 1.02E-06 |  | 0.0194141 | 0.0199128 | 0.329583 | 23.85402226 |
| Meales-23andMe | rs12700593 | C | T | 0.28115 | 0.0659 | 0.0139 | 2.13E-06 |  | -0.00621228 | 0.0182262 | 0.733222 | 22.47714922 |
| Meales-23andMe | rs1353279 | C | T | 0.494808 | -0.0697 | 0.0138 | 4.40E-07 |  | -0.00849807 | 0.0181315 | 0.639291 | 25.50981937 |
| Meales-23andMe | rs4568518 | A | G | 0.384585 | 0.064 | 0.014 | 4.84E-06 |  | -0.0221989 | 0.0186233 | 0.233263 | 20.89795918 |
| Meales-23andMe | rs500141 | C | T | 0.424321 | -0.073 | 0.0149 | 9.62E-07 |  | -0.0118085 | 0.0196257 | 0.547383 | 24.00342327 |
| Meales-23andMe | rs67559144 | C | T | 0.358027 | -0.0768 | 0.0153 | 5.18E-07 |  | -0.030339 | 0.0203663 | 0.136311 | 25.1964629 |
| Meales-23andMe | rs9879864 | A | G | 0.256789 | 0.0665 | 0.0139 | 1.72E-06 |  | 0.0257847 | 0.0184133 | 0.161413 | 22.88830806 |
| Meales-FINN | rs1490827 | G | A | 0.5628 | 0.5087 | 0.1074 | 2.19E-06 |  | -0.0439945 | 0.0187171 | 0.0187487 | 22.43442343 |
| Meales-FINN | rs2351647 | T | C | 0.5044 | 0.4957 | 0.1068 | 3.45E-06 |  | -0.0207274 | 0.0182534 | 0.256151 | 21.54246185 |
| Meales-FINN | rs6825765 | C | A | 0.5726 | -0.5933 | 0.1088 | 4.99E-08 |  | 0.0226093 | 0.0191772 | 0.238409 | 29.73657227 |
| Meales-FINN | rs875031 | G | A | 0.7938 | 0.6518 | 0.1363 | 1.74E-06 |  | 0.00768678 | 0.0282101 | 0.785251 | 22.86846427 |
| Acute poliomyelitis | rs10110503 | A | G | 0.5451 | 0.4506 | 0.0947 | 1.94E-06 |  | -0.0125769 | 0.0183148 | 0.492266 | 22.64031249 |
| Acute poliomyelitis | rs116741188 | T | C | 0.05481 | 1.189 | 0.2305 | 2.48E-07 |  | 0.04684 | 0.0437474 | 0.284308 | 26.60858927 |
| Acute poliomyelitis | rs2097479 | C | A | 0.9759 | -1.5883 | 0.3474 | 4.83E-06 |  | -0.00118486 | 0.0483013 | 0.980429 | 20.90284709 |
| Rubella-23andMe | rs10208323 | G | T | 0.082867 | 0.2079 | 0.0435 | 1.76E-06 |  | 0.0136155 | 0.0525399 | 0.795523 | 22.84180737 |
| Rubella-23andMe | rs11209896 | A | G | 0.408347 | -0.0747 | 0.0156 | 1.68E-06 |  | 0.0202402 | 0.0183327 | 0.26957 | 22.92936391 |
| Rubella-23andMe | rs149417607 | A | G | 0.007588 | -0.3591 | 0.0786 | 4.91E-06 |  | -0.016812 | 0.0833893 | 0.840223 | 20.87304062 |
| Rubella-23andMe | rs2601552 | A | G | 0.297724 | -0.0961 | 0.0209 | 4.26E-06 |  | -0.00109684 | 0.0238681 | 0.963347 | 21.14239601 |
| Rubella-23andMe | rs2892940 | A | G | 0.341254 | 0.1082 | 0.0227 | 1.87E-06 |  | 0.0305959 | 0.0258935 | 0.237363 | 22.71971123 |
| Rubella-23andMe | rs66878487 | A | G | 0.022564 | -0.1586 | 0.0318 | 6.12E-07 |  | 0.0138016 | 0.0385995 | 0.720673 | 24.87437206 |
| Rubella-23andMe | rs79031264 | A | G | 0.014776 | 0.1837 | 0.039 | 2.47E-06 |  | 0.0462531 | 0.0468336 | 0.323346 | 22.18651545 |
| Rubella-FINN | rs112429055 | G | A | 0.02597 | 0.9557 | 0.2084 | 4.52E-06 |  | 0.0309358 | 0.0866581 | 0.721102 | 21.03041015 |
| Rubella-FINN | rs116363196 | C | T | 0.08123 | 0.5931 | 0.1159 | 3.11E-07 |  | 0.0773365 | 0.0437204 | 0.0769123 | 26.18719464 |
| Rubella-FINN | rs117318756 | C | T | 0.01124 | 1.6083 | 0.3359 | 1.69E-06 |  | -0.0504031 | 0.0844115 | 0.550433 | 22.92525189 |
| Rubella-FINN | rs2269790 | T | C | 0.1969 | 0.3829 | 0.0795 | 1.45E-06 |  | 0.0253851 | 0.0243034 | 0.296249 | 23.19724853 |
| Rubella-FINN | rs57810677 | A | G | 0.473 | 0.2862 | 0.0615 | 3.25E-06 |  | 0.00934573 | 0.0187321 | 0.617839 | 21.65653778 |
| Rubella-FINN | rs78555786 | A | G | 0.007881 | 1.8824 | 0.4093 | 4.24E-06 |  | 0.0497116 | 0.0809947 | 0.539372 | 21.15145945 |
| Rubella-FINN | rs919965 | T | C | 0.4526 | 0.295 | 0.0613 | 1.48E-06 |  | 0.0216965 | 0.018211 | 0.233498 | 23.15917492 |
| Rubella-FINN | rs941604 | G | A | 0.6551 | 0.2951 | 0.0646 | 4.84E-06 |  | -0.0206059 | 0.0190907 | 0.280422 | 20.86764227 |
