# Supplementary material for: Association between viral infections and glioma risk: a two-sample bidirectional Mendelian randomization analysis
Source: BMC Med. 2023 Dec 5;21:487. doi: 10.1186/s12916-023-03142-9 (PMC10698979; doi:10.1186/s12916-023-03142-9)
Supplement: Supplementary file 2 — Additional file 2. Summary of the 8 glioma GWASs used in the meta-analysis. [file 12916_2023_3142_MOESM2_ESM.docx]

**Additional file 2.** Summary of GWAS Datasets

| **Dataset** | **PUBMED ID** | **LGG cases** | **GBM cases** | **All-glioma cases** | **Number of controls** |
| --- | --- | --- | --- | --- | --- |
| UK-GWAS | 17636416 | 361 | 270 | 631 | 2699 |
| French-GWAS | 21531791 | 993 | 430 | 1423 | 1190 |
| German-GWAS | 26424050 | 415 | 431 | 846 | 1310 |
| MDA-GWAS | 19578367 | 523 | 652 | 1175 | 2236 |
| UCSF- SFAGS | 19578367 | 166 | 511 | 677 | 3940 |
| GliomaScan | 22886559 | 472 | 903 | 1653 | 2725 |
| GICC | 26656478 | 1898 | 2460 | 4564 | 3265 |
| UCSF/Mayo | 19578366 | 992 | 526 | 1519 | 804 |
| **Total** |  | **5820** | **6183** | **12488** | **18169** |
