# Supplementary material for: Association between viral infections and glioma risk: a two-sample bidirectional Mendelian randomization analysis
Source: BMC Med. 2023 Dec 5;21:487. doi: 10.1186/s12916-023-03142-9 (PMC10698979; doi:10.1186/s12916-023-03142-9)
Supplement: Supplementary file 3 — Additional file 3. Mendelian randomization results of weighted median and MR–Egger methods, sensitivity analysis, for viral infection in glioma. Abbreviations: P (heterogeneity): P value of Cochrane’s Q value in heterogeneity test; P (pleiotropy): P value of MR–Egger intercept. [file 12916_2023_3142_MOESM3_ESM.docx]

**Additional file 3** Mendelian randomization results of weighted median and MR‒Egger methods, sensitivity analysis, for viral infection in glioma. Abbreviations: P (heterogeneity): P value of Cochrane’s Q value in heterogeneity test; P (pleiotropy): P value of MR‒Egger intercept.

|  |  |  |  | **Weighted median** | | | |  | **MR Egger** | | | |  | **Heterogeneity test** | | |  |
| --- | --- | --- | --- | --- | --- | --- | --- | --- | --- | --- | --- | --- | --- | --- | --- | --- | --- |
| **Outcome** | **Exposure** | **Data source** | **NSNP** | **Pvalue** | **OR** | **95%LCI** | **95%UCI** |  | **Pvalue** | **OR** | **95%LCI** | **95%UCI** |  | **P(pleiotropy)** | **Q** | **P(heterogeneity)** | **Power** |
| **LGG** | Cold sores | 23andMe | 5 | 0.0204 | 1.7098 | 1.0867 | 2.6904 |  | 0.7384 | 0.8716 | 0.4180 | 1.8175 |  | 0.2447 | 4.4711 | 0.3460 | 0.9600 |
|  | Herpesviral infections | FINN | 6 | 0.0436 | 1.1685 | 1.0045 | 1.3593 |  | 0.1606 | 2.0563 | 0.9041 | 4.6769 |  | 0.2305 | 4.4212 | 0.4905 | 0.9000 |
|  | Herpes zoster | FINN | 7 | 0.0299 | 0.8441 | 0.7244 | 0.9836 |  | 0.3014 | 0.7716 | 0.4963 | 1.1994 |  | 0.6715 | 2.8909 | 0.8224 | 0.8200 |
|  | Mumps | FINN | 4 | 0.0881 | 1.0641 | 0.9908 | 1.1429 |  | 0.3049 | 1.0722 | 0.9702 | 1.1850 |  | 0.8360 | 1.9387 | 0.5852 | 0.8500 |
|  | Mumps | 23andMe | 11 | 0.5743 | 0.9265 | 0.7098 | 1.2093 |  | 0.6000 | 0.8664 | 0.5165 | 1.4532 |  | 0.8865 | 4.1684 | 0.9394 | 0.0900 |
|  | Infectious mononucleosis | FINN | 14 | 0.3172 | 1.0368 | 0.9660 | 1.1127 |  | 0.6593 | 1.0214 | 0.9319 | 1.1193 |  | 0.7807 | 12.6642 | 0.4741 | 0.0800 |
|  | Mononucleosis | 23andme | 3 | 0.0490 | 0.5660 | 0.3211 | 0.9976 |  | 0.2994 | 0.0001 | 0.0000 | 0.9664 |  | 0.3124 | 3.5036 | 0.1735 | 0.9700 |
|  | Cytomegaloviral disease | FINN | 7 | 0.3930 | 1.0218 | 0.9724 | 1.0738 |  | 0.5493 | 1.0268 | 0.9471 | 1.1132 |  | 0.6449 | 2.3742 | 0.8823 | 0.0600 |
|  | COVID-19 hospitalized | COVID-19 HGI | 21 | 0.3432 | 0.9336 | 0.8099 | 1.0761 |  | 0.6534 | 0.9302 | 0.6816 | 1.2694 |  | 0.9394 | 14.9934 | 0.7768 | 0.0800 |
|  | HPV | Suhre | 4 | 0.6421 | 0.9691 | 0.8489 | 1.1063 |  | 0.7271 | 0.8966 | 0.5260 | 1.5282 |  | 0.7112 | 2.3493 | 0.5031 | 0.0500 |
|  | Acute poliomyelitis | FINN | 3 | 0.8137 | 0.9926 | 0.9333 | 1.0557 |  | 0.7952 | 1.0174 | 0.9192 | 1.1262 |  | 0.6894 | 0.6146 | 0.7354 | 0.0600 |
|  | HIV disease | FINN | 8 | 0.1640 | 1.0384 | 0.9847 | 1.0951 |  | 0.2161 | 1.0481 | 0.9806 | 1.1204 |  | 0.6162 | 6.8600 | 0.4436 | 0.4400 |
|  | Viral hepatitis | FINN | 4 | 0.6373 | 1.0405 | 0.8820 | 1.2275 |  | 0.6783 | 1.1632 | 0.6277 | 2.1557 |  | 0.7039 | 12.5225 | 0.0058 | 0.1000 |
|  | Hepatitis B | 23andMe | 6 | 0.1845 | 1.0907 | 0.9594 | 1.2400 |  | 0.4106 | 1.1603 | 0.8447 | 1.5939 |  | 0.6629 | 4.8889 | 0.4296 | 0.4500 |
|  | Rubella | FINN | 8 | 0.6822 | 1.0155 | 0.9435 | 1.0930 |  | 0.9495 | 0.9967 | 0.9038 | 1.0991 |  | 0.7462 | 5.3180 | 0.6212 | 0.0500 |
|  | Rubella | 23andMe | 6 | 0.2067 | 0.8037 | 0.5725 | 1.1283 |  | 0.5508 | 0.7419 | 0.3019 | 1.8236 |  | 0.7260 | 5.8107 | 0.3251 | 1.0000 |
|  | Measles | FINN | 4 | 0.3136 | 0.9713 | 0.9178 | 1.0279 |  | 0.8704 | 1.0477 | 0.6391 | 1.7176 |  | 0.7694 | 2.5335 | 0.4693 | 0.5500 |
|  | Measles | 23andMe | 6 | 0.0636 | 0.6904 | 0.4667 | 1.0211 |  | 0.4413 | 0.0683 | 0.0001 | 32.3767 |  | 0.5027 | 8.0028 | 0.1561 | 0.8700 |
| **GBM** | Cold sores | 23andMe | 5 | 0.4274 | 1.1657 | 0.7983 | 1.7022 |  | 0.6533 | 1.1915 | 0.5972 | 2.3772 |  | 0.9170 | 1.1655 | 0.8837 | 0.6800 |
|  | Herpesviral infections | FINN | 6 | 0.7959 | 0.9815 | 0.8518 | 1.1309 |  | 0.6750 | 1.1939 | 0.5532 | 2.5767 |  | 0.6420 | 3.5376 | 0.6177 | 0.0800 |
|  | Herpes zoster | FINN | 8 | 0.0272 | 1.1313 | 1.0140 | 1.2622 |  | 0.1223 | 1.1282 | 0.9892 | 1.2866 |  | 0.7904 | 3.9273 | 0.7881 | 0.2800 |
|  | Mumps | FINN | 4 | 0.4648 | 1.0310 | 0.9500 | 1.1188 |  | 0.0976 | 1.1520 | 1.0490 | 1.2650 |  | 0.1347 | 6.4713 | 0.0908 | 0.6400 |
|  | Mumps | 23andMe | 12 | 0.4636 | 0.9156 | 0.7231 | 1.1592 |  | 0.5641 | 0.8729 | 0.5583 | 1.3645 |  | 0.8878 | 4.4086 | 0.9564 | 0.5600 |
|  | Infectious mononucleosis | FINN | 14 | 0.6942 | 0.9861 | 0.9198 | 1.0572 |  | 0.9050 | 0.9948 | 0.9149 | 1.0817 |  | 0.5346 | 12.1115 | 0.5185 | 0.2600 |
|  | Mononucleosis | 23andme | 3 | 0.5524 | 0.8696 | 0.5486 | 1.3786 |  | 0.6800 | 10.6570 | 0.0023 | 49067.9218 |  | 0.6670 | 0.4400 | 0.8025 | 0.1600 |
|  | Cytomegaloviral disease | FINN | 7 | 0.6559 | 1.0111 | 0.9633 | 1.0612 |  | 0.8250 | 1.0100 | 0.9292 | 1.0977 |  | 0.9099 | 6.0828 | 0.4140 | 1.0000 |
|  | COVID-19 hospitalized | COVID-19 HGI | 22 | 0.1361 | 1.1081 | 0.9682 | 1.2684 |  | 0.6802 | 0.9289 | 0.6575 | 1.3123 |  | 0.4618 | 30.7727 | 0.0775 | 0.4000 |
|  | HPV | Suhre | 4 | 0.5493 | 1.0364 | 0.9220 | 1.1650 |  | 0.5305 | 1.2024 | 0.7437 | 1.9439 |  | 0.5291 | 1.3994 | 0.7057 | 0.0500 |
|  | Acute poliomyelitis | FINN | 3 | 0.8754 | 1.0048 | 0.9460 | 1.0673 |  | 0.5338 | 1.0447 | 0.9497 | 1.1491 |  | 0.5514 | 1.7554 | 0.4157 | 0.0600 |
|  | HIV disease | FINN | 8 | 0.6533 | 0.9890 | 0.9425 | 1.0379 |  | 0.8221 | 0.9926 | 0.9328 | 1.0562 |  | 0.7916 | 5.7489 | 0.5694 | 0.0500 |
|  | Viral hepatitis | FINN | 4 | 0.0923 | 1.1403 | 0.9787 | 1.3286 |  | 0.2548 | 1.2363 | 0.9503 | 1.6083 |  | 0.4241 | 3.7434 | 0.2905 | 0.5600 |
|  | Hepatitis B | 23andMe | 6 | 0.6006 | 0.9700 | 0.8656 | 1.0871 |  | 0.4424 | 1.1277 | 0.8552 | 1.4870 |  | 0.4008 | 3.6296 | 0.6039 | 0.0500 |
|  | Rubella | FINN | 8 | 0.5218 | 0.9773 | 0.9112 | 1.0483 |  | 0.9615 | 1.0031 | 0.8888 | 1.1321 |  | 0.6636 | 10.7831 | 0.1484 | 0.0600 |
|  | Rubella | 23andMe | 7 | 0.8451 | 1.0291 | 0.7718 | 1.3722 |  | 0.5996 | 1.1642 | 0.6839 | 1.9818 |  | 0.5135 | 4.8184 | 0.5673 | 0.1600 |
|  | Measles | FINN | 4 | 0.0908 | 0.9522 | 0.8996 | 1.0078 |  | 0.3133 | 1.3336 | 0.8741 | 2.0346 |  | 0.2577 | 2.7416 | 0.4332 | 0.7800 |
|  | Measles | 23andMe | 6 | 0.5006 | 0.8978 | 0.6562 | 1.2285 |  | 0.6067 | 3.4180 | 0.0455 | 256.6179 |  | 0.5821 | 1.0118 | 0.9616 | 0.1200 |
| **all-glioma** | Cold sores | 23andMe | 5 | 0.0722 | 1.3577 | 0.9728 | 1.8951 |  | 0.7924 | 1.0860 | 0.6188 | 1.9060 |  | 0.5101 | 2.1236 | 0.7130 | 0.8300 |
|  | Herpesviral infections | FINN | 6 | 0.5095 | 1.0364 | 0.9319 | 1.1526 |  | 0.3361 | 1.4170 | 0.7581 | 2.6488 |  | 0.4026 | 3.1419 | 0.6781 | 0.0800 |
|  | Herpes zoster | FINN | 8 | 0.6825 | 1.0183 | 0.9334 | 1.1110 |  | 0.2345 | 1.0750 | 0.9657 | 1.1966 |  | 0.2289 | 3.4516 | 0.8403 | 0.0500 |
|  | Mumps | FINN | 14 | 0.9762 | 0.9991 | 0.9434 | 1.0581 |  | 0.9905 | 1.0005 | 0.9257 | 1.0813 |  | 0.1896 | 5.3549 | 0.1476 | 0.9600 |
|  | Mumps | 23andMe | 3 | 0.1098 | 0.7228 | 0.4855 | 1.0760 |  | 0.2680 | 0.8045 | 0.5594 | 1.1571 |  | 0.6407 | 2.5907 | 0.9951 | 0.1100 |
|  | Infectious mononucleosis | FINN | 14 | 0.6942 | 0.9861 | 0.9198 | 1.0572 |  | 0.9050 | 0.9948 | 0.9149 | 1.0817 |  | 0.7842 | 15.7743 | 0.2615 | 0.0500 |
|  | Mononucleosis | 23andMe | 3 | 0.5524 | 0.8696 | 0.5486 | 1.3786 |  | 0.5657 | 0.0575 | 0.0001 | 56.6186 |  | 0.5956 | 0.6851 | 0.7099 | 0.1700 |
|  | Cytomegaloviral disease | FINN | 7 | 0.8051 | 1.0053 | 0.9638 | 1.0486 |  | 0.8727 | 1.0055 | 0.9434 | 1.0716 |  | 0.8763 | 5.4875 | 0.4830 | 0.0600 |
|  | COVID-19 hospitalized | COVID-19 HGI | 21 | 0.9977 | 0.9998 | 0.8934 | 1.1189 |  | 0.2985 | 0.8787 | 0.6933 | 1.1138 |  | 0.2531 | 20.1205 | 0.4504 | 0.0600 |
|  | HPV | Suhre | 4 | 0.8996 | 1.0067 | 0.9073 | 1.1170 |  | 0.9152 | 1.0293 | 0.6435 | 1.6464 |  | 0.8917 | 2.8381 | 0.4173 | 0.0500 |
|  | Acute poliomyelitis | FINN | 3 | 0.8984 | 1.0032 | 0.9546 | 1.0543 |  | 0.5540 | 1.0337 | 0.9570 | 1.1166 |  | 0.5607 | 1.5487 | 0.4610 | 0.0600 |
|  | HIV disease | FINN | 8 | 0.4571 | 1.0157 | 0.9748 | 1.0584 |  | 0.5744 | 1.0157 | 0.9649 | 1.0691 |  | 0.9129 | 6.5532 | 0.4768 | 0.2500 |
|  | Viral hepatitis | FINN | 4 | 0.2870 | 1.0688 | 0.9456 | 1.2081 |  | 0.5514 | 1.1555 | 0.7752 | 1.7224 |  | 0.6705 | 9.4975 | 0.0234 | 0.3400 |
|  | Hepatitis B | 23andMe | 6 | 0.8114 | 1.0125 | 0.9146 | 1.1208 |  | 0.3249 | 1.1361 | 0.9090 | 1.4199 |  | 0.4844 | 4.2061 | 0.5201 | 0.2800 |
|  | Rubella | FINN | 8 | 0.3218 | 1.0307 | 0.9709 | 1.0942 |  | 0.6633 | 1.0181 | 0.9428 | 1.0993 |  | 0.8064 | 6.4048 | 0.4934 | 0.0600 |
|  | Rubella | 23andMe | 7 | 0.5977 | 0.9386 | 0.7417 | 1.1877 |  | 0.8369 | 0.9532 | 0.6183 | 1.4696 |  | 0.9549 | 3.4106 | 0.7558 | 0.9200 |
|  | Measles | FINN | 4 | 0.0892 | 0.9609 | 0.9178 | 1.0061 |  | 0.3965 | 1.2093 | 0.8539 | 1.7128 |  | 0.3192 | 3.0210 | 0.3884 | 0.8600 |
|  | Measles | 23andMe | 6 | 0.1045 | 0.7982 | 0.6081 | 1.0478 |  | 0.8605 | 0.7139 | 0.0210 | 24.2435 |  | 0.9443 | 2.9522 | 0.7074 | 0.6500 |
