# Supplementary material for: Association between viral infections and glioma risk: a two-sample bidirectional Mendelian randomization analysis
Source: BMC Med. 2023 Dec 5;21:487. doi: 10.1186/s12916-023-03142-9 (PMC10698979; doi:10.1186/s12916-023-03142-9)
Supplement: Supplementary file 4 — Additional file 4. Leave-one-out plots, forest plots, and scatter plots in primary analysis. [file 12916_2023_3142_MOESM4_ESM.docx]

**Additional file 4. Leave-one-out plots, forest plots, and scatter plots in primary analysis.**

**Figure S1.** The leave-one-out plot, forest plot, and scatter plot for the association of Herpes zoster and LGG in primary analysis. Data from FINN.


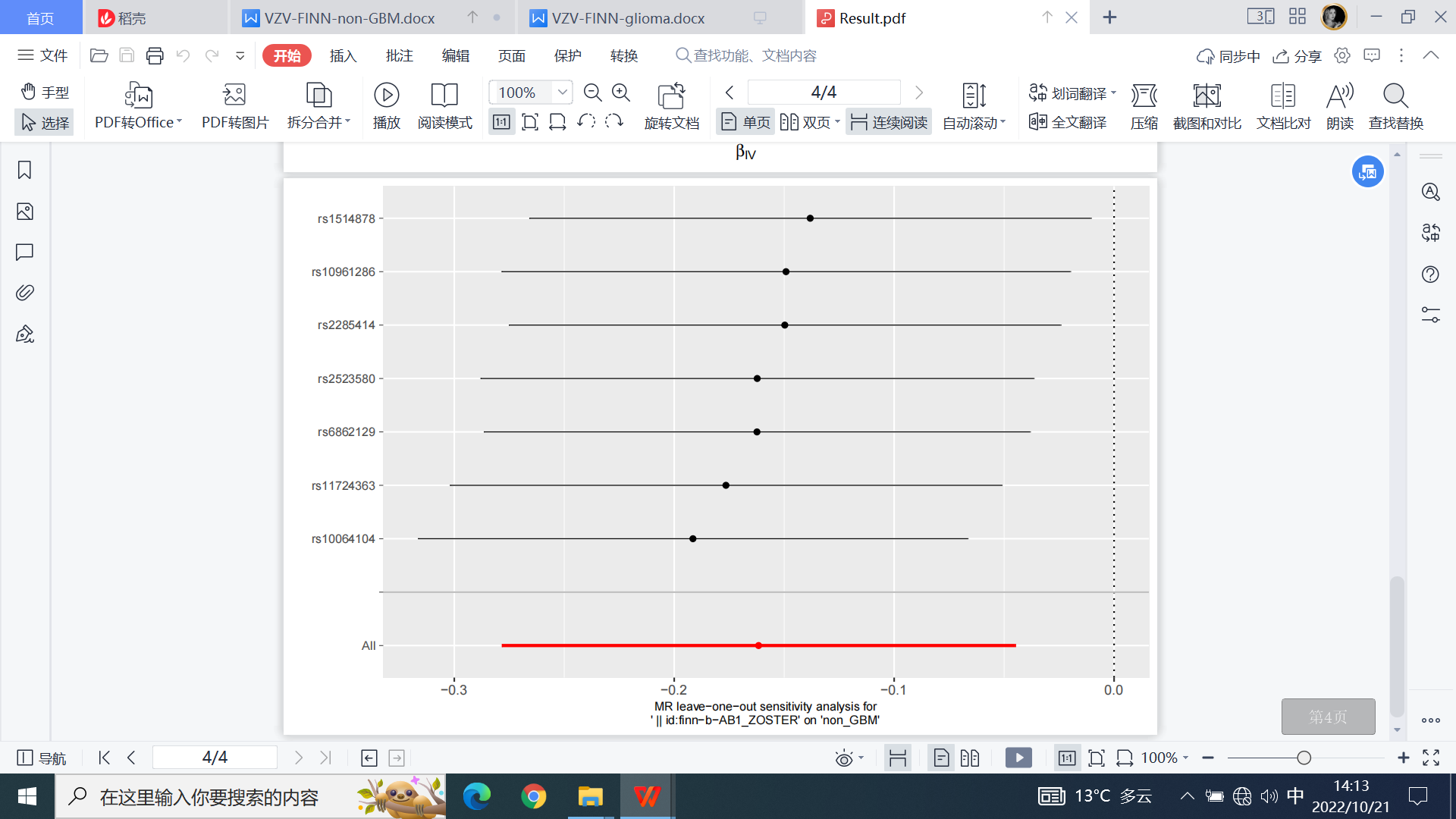


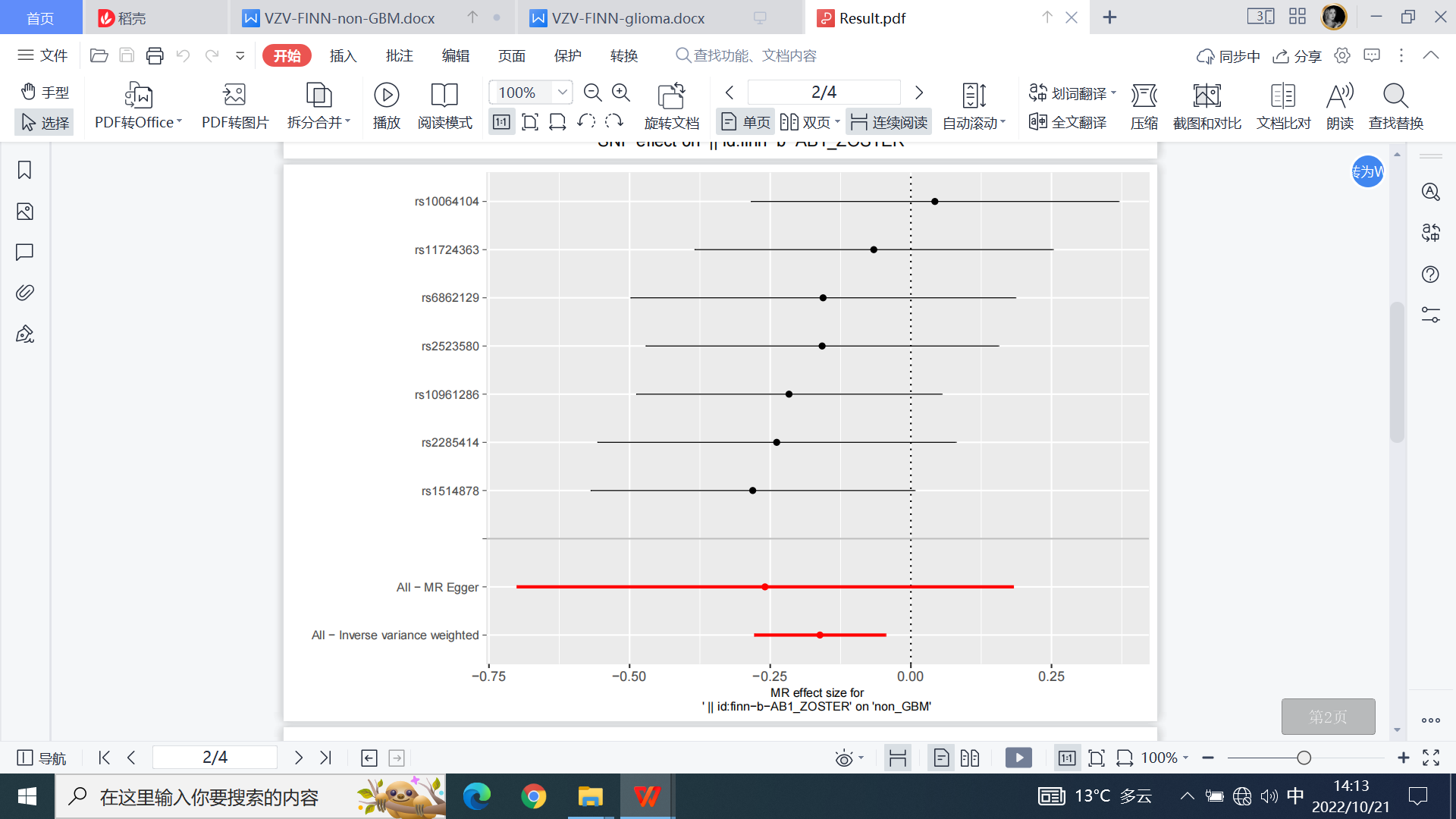


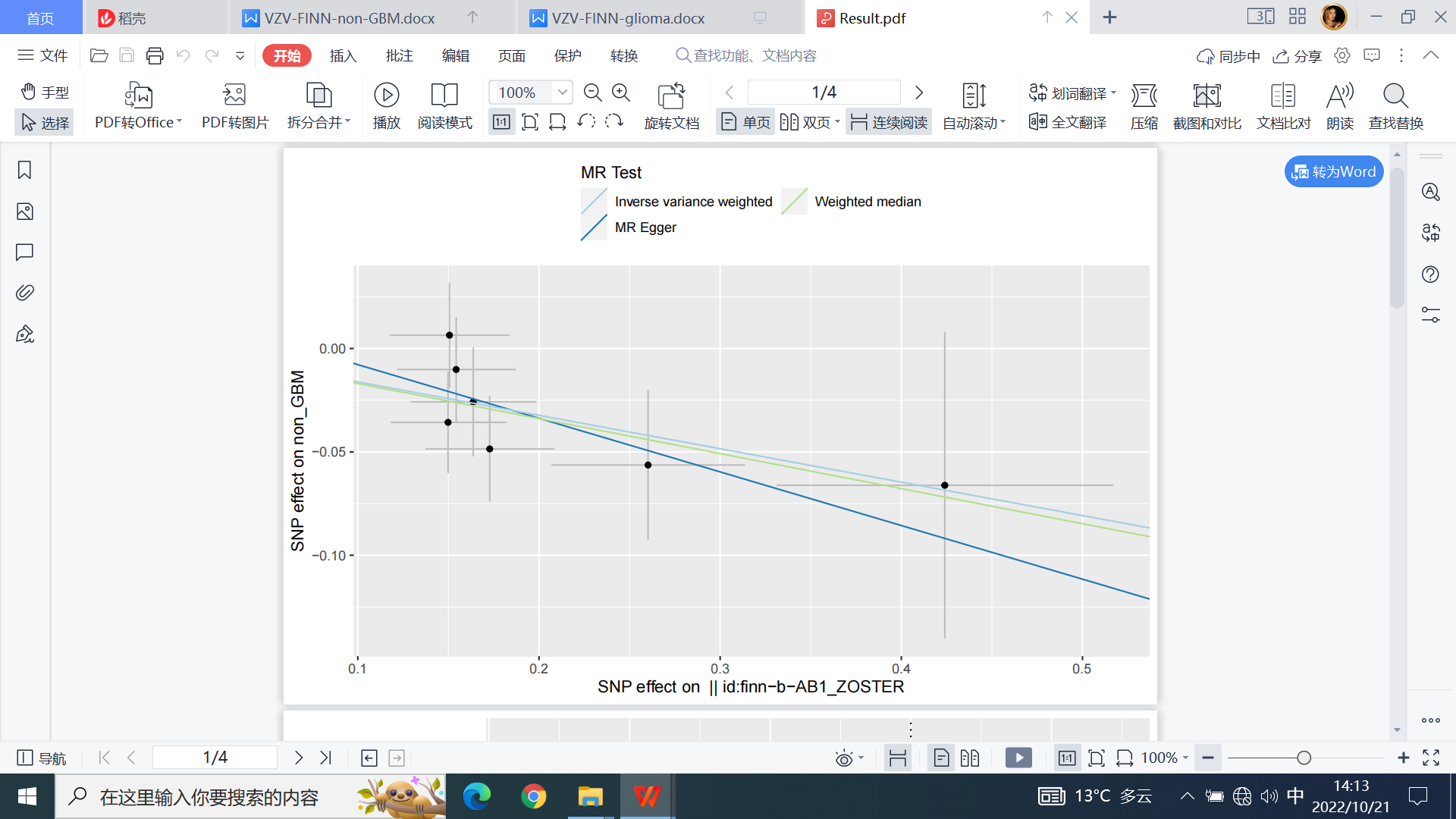


**Figure S2.** The leave-one-out plot, forest plot, and scatter plot for the association of mumps infection and LGG in primary analysis. Data from FINN.


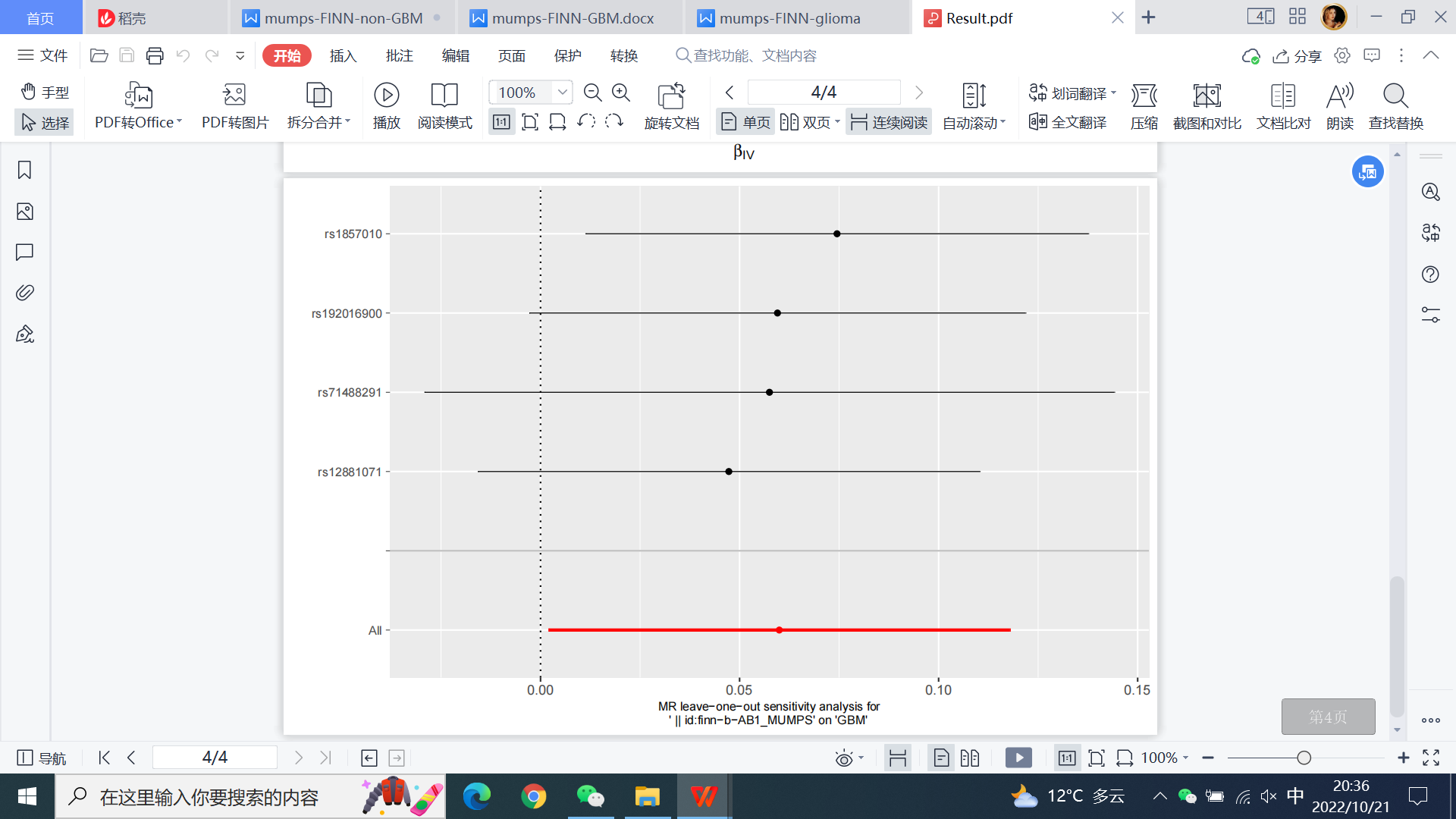


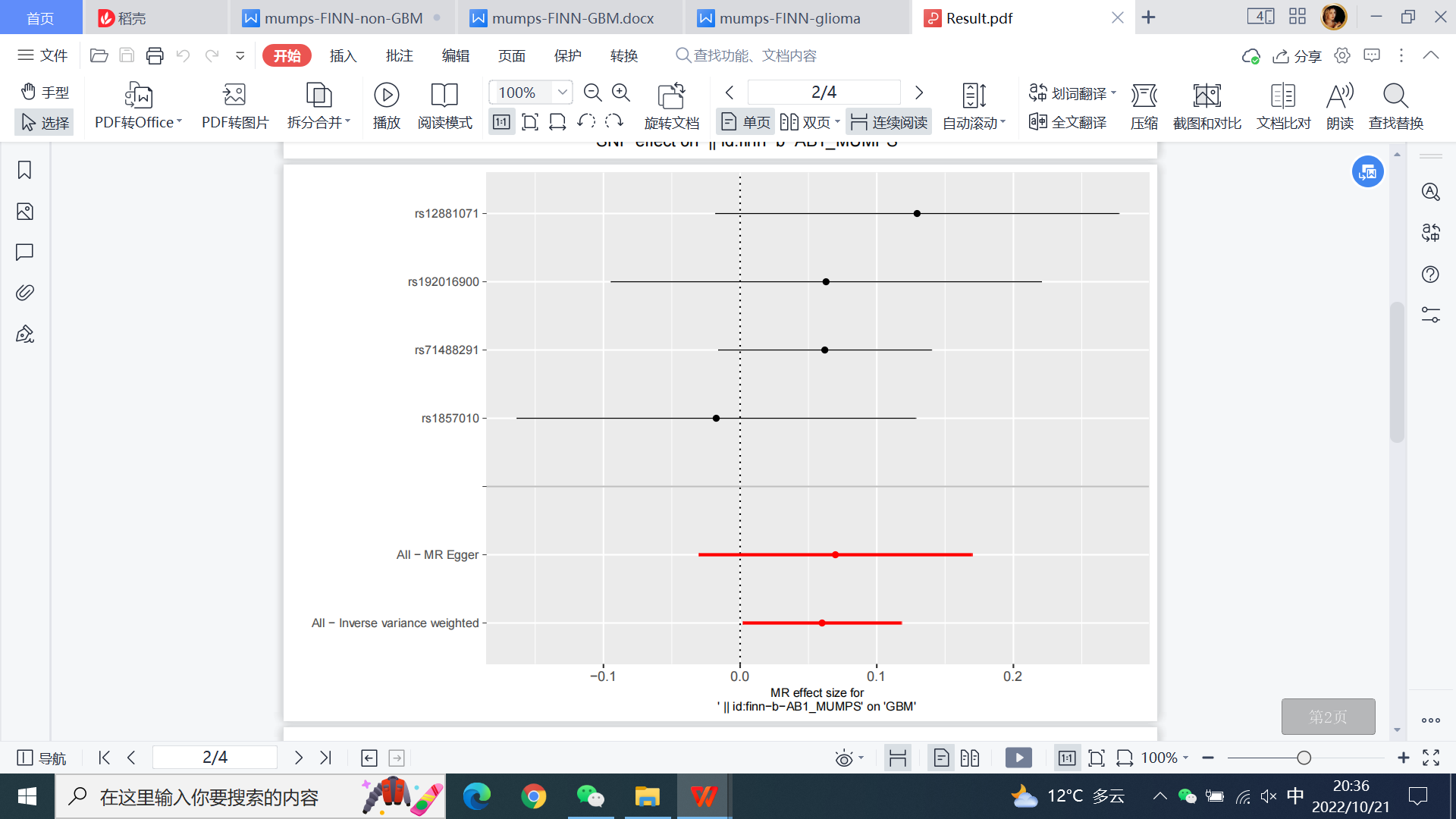

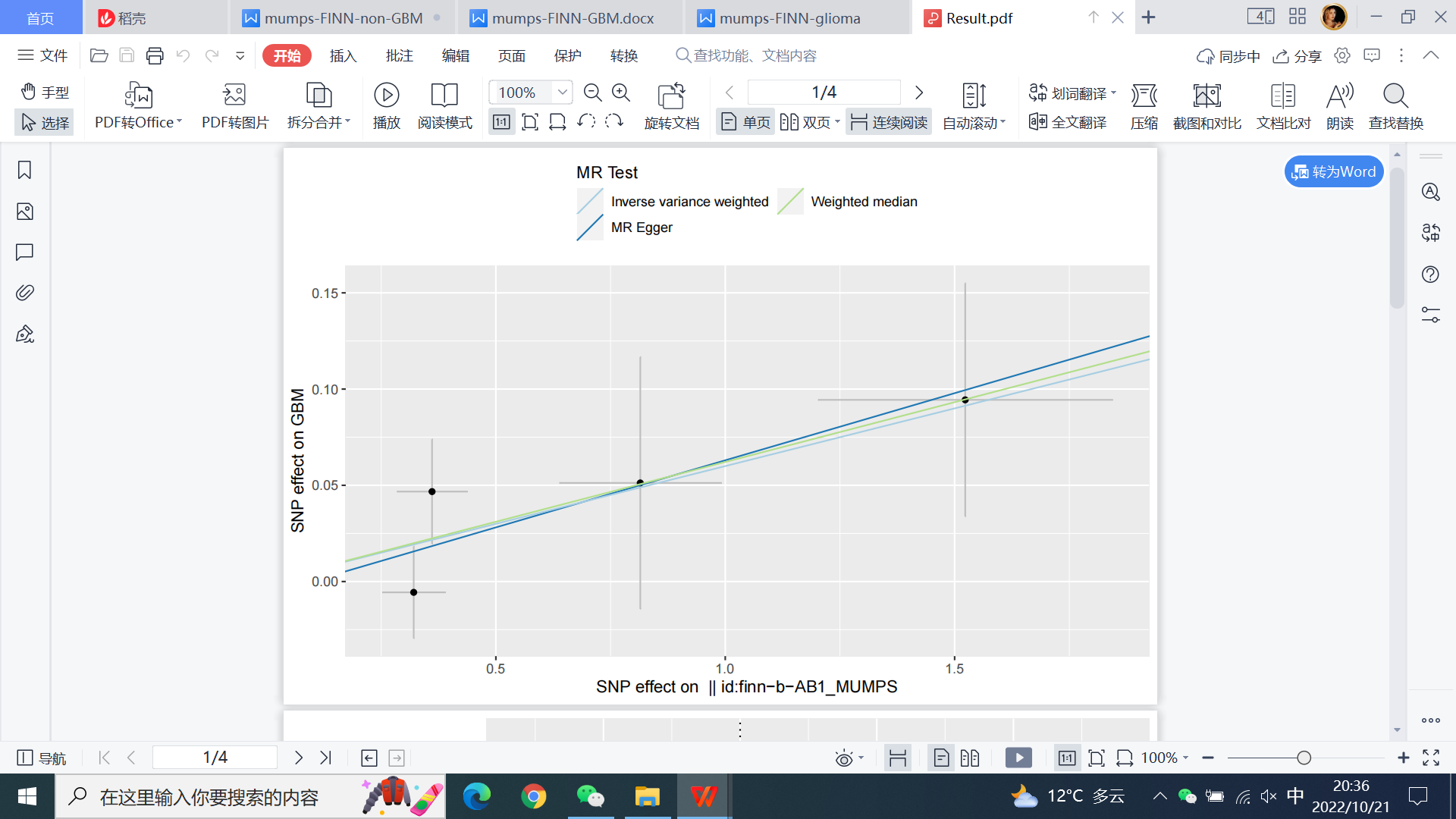


**Figure S3.** The leave-one-out plot, forest plot, and scatter plot for the association of mumps infection and LGG in primary analysis. Data from 23andme.


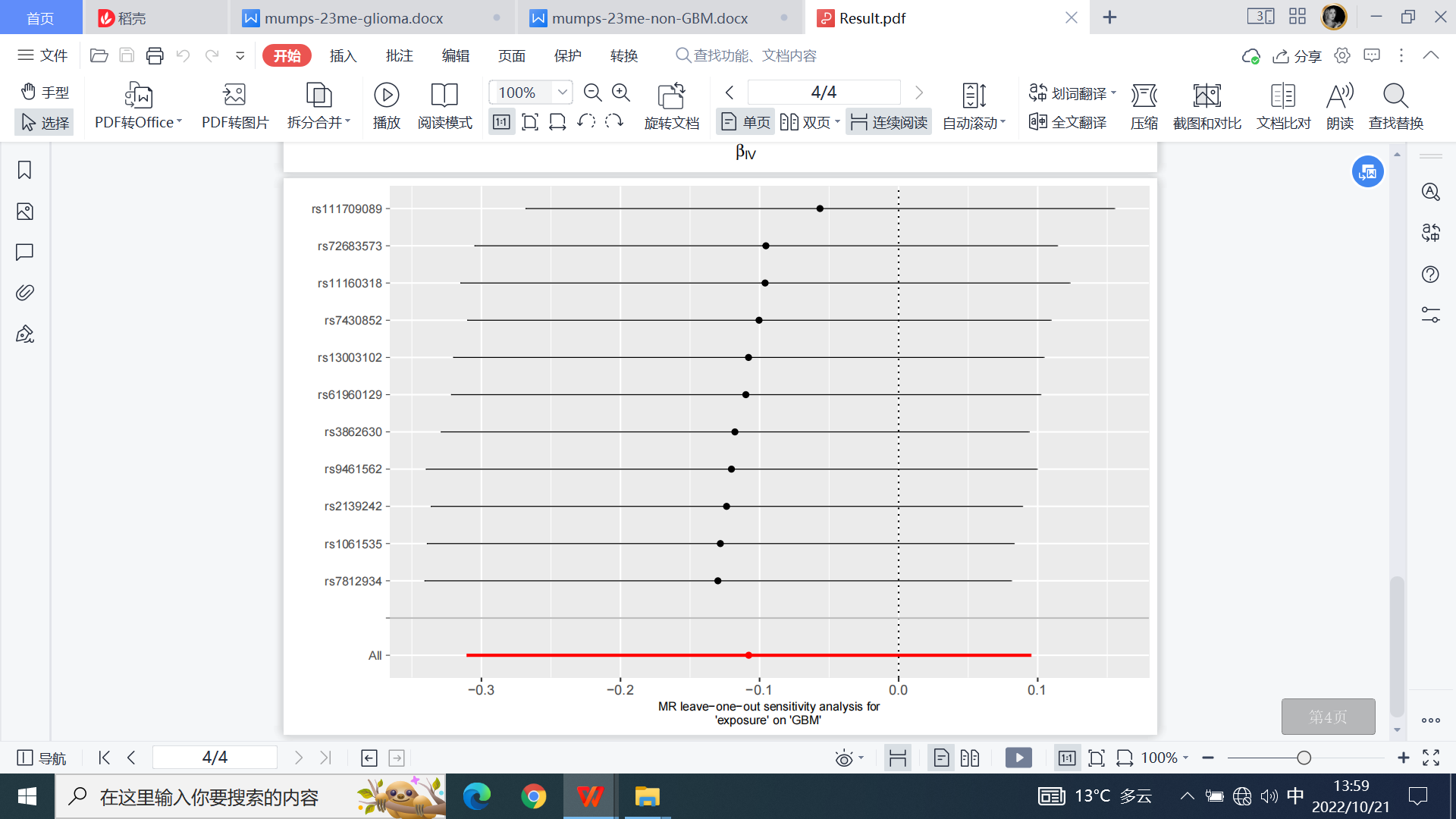


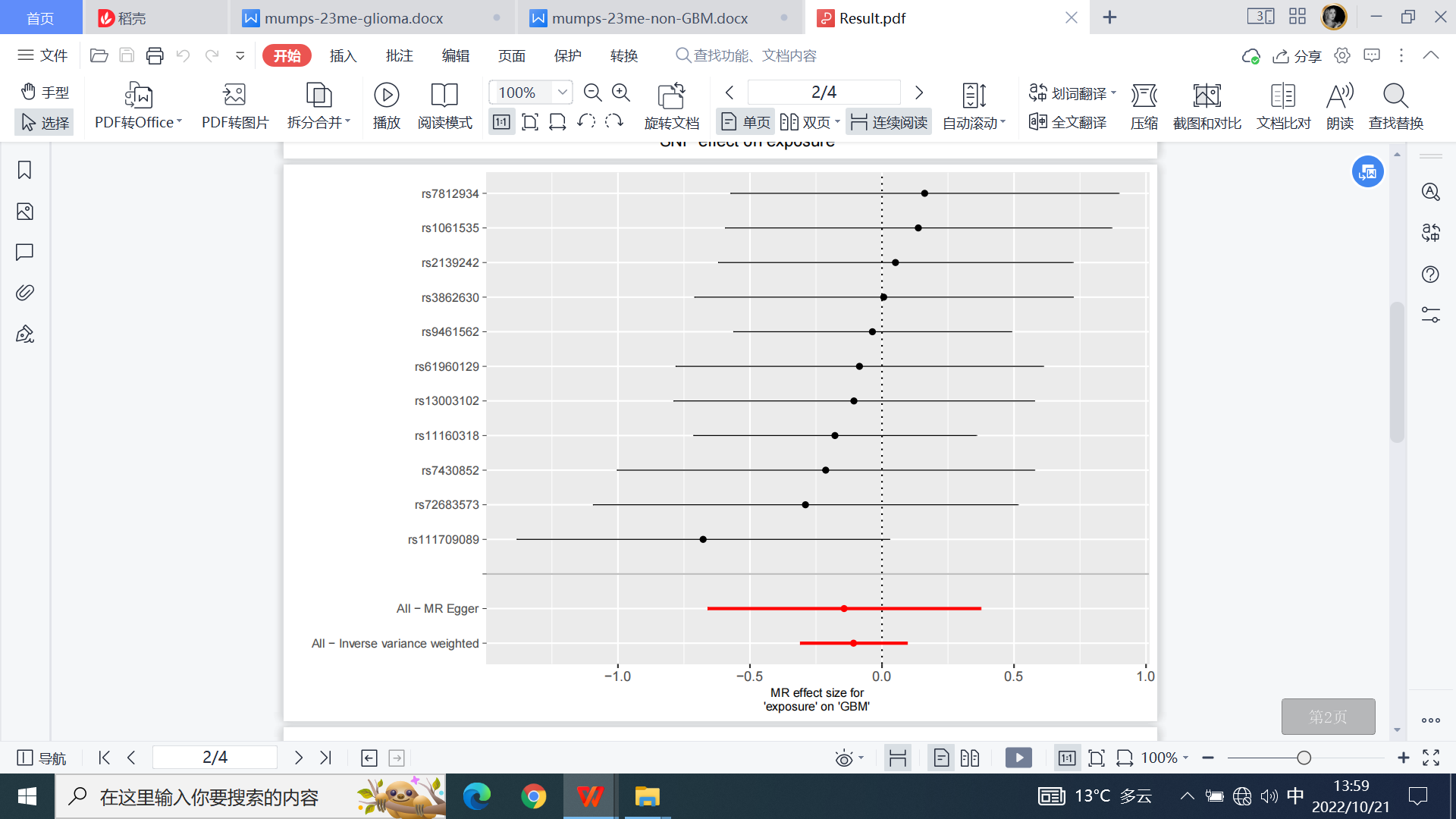


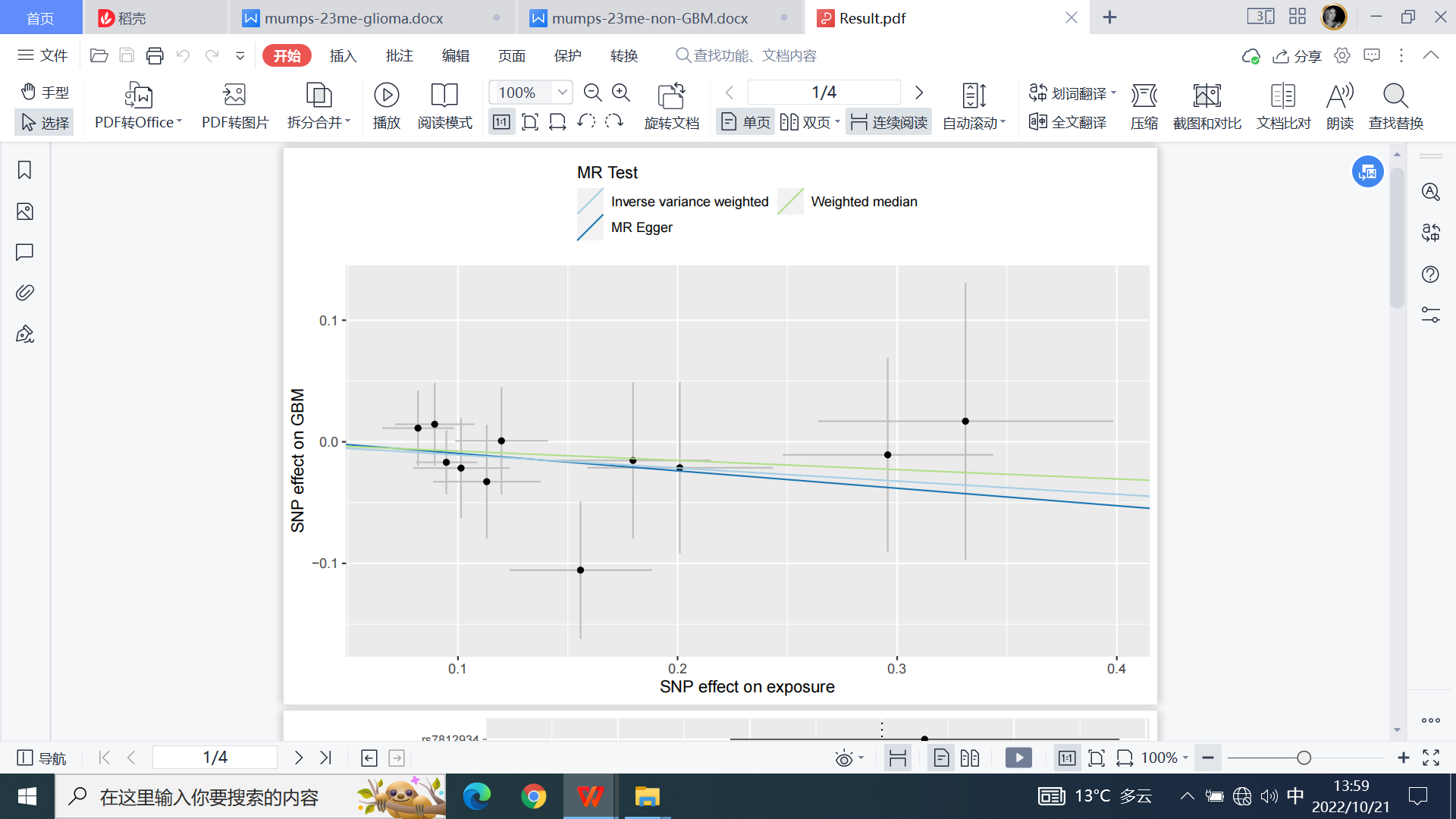


**Figure S4.** The leave-one-out plot, forest plot, and scatter plot for the association of HSV infection and LGG in primary analysis. Data from FINN.


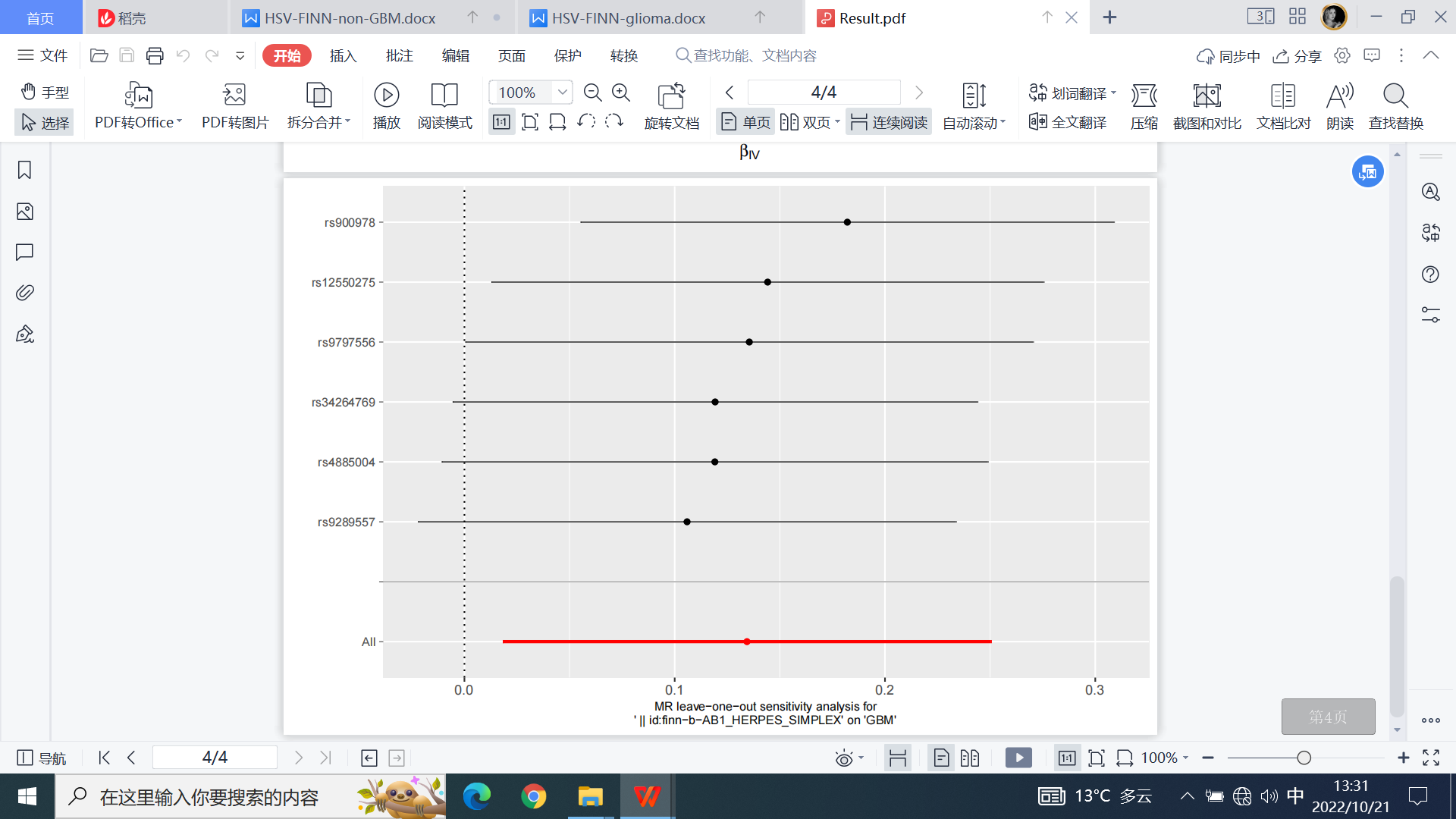


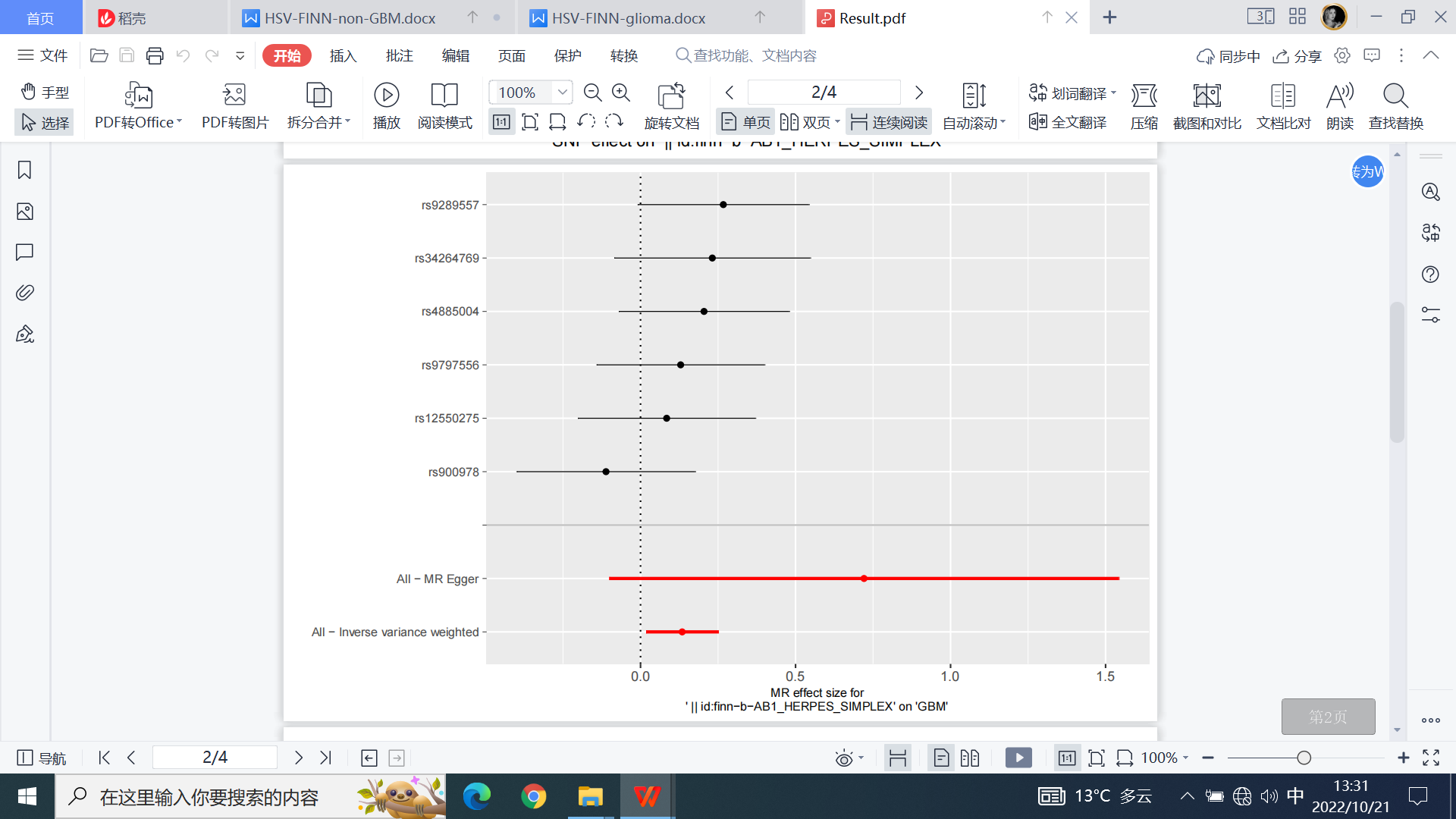


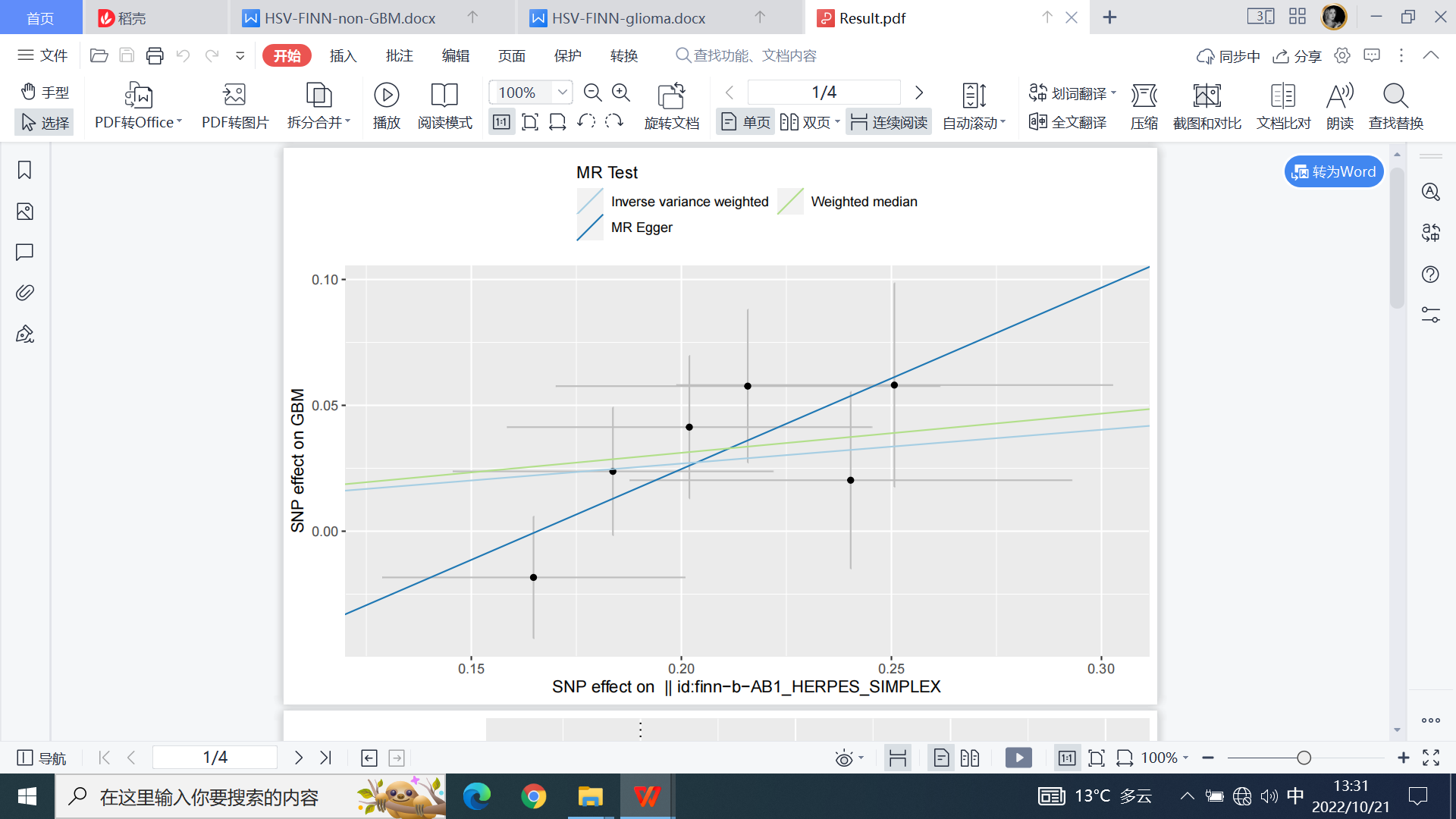


**Figure S5.** The leave-one-out plot, forest plot, and scatter plot for the association of HSV infection and LGG in primary analysis. Data from 23andme.


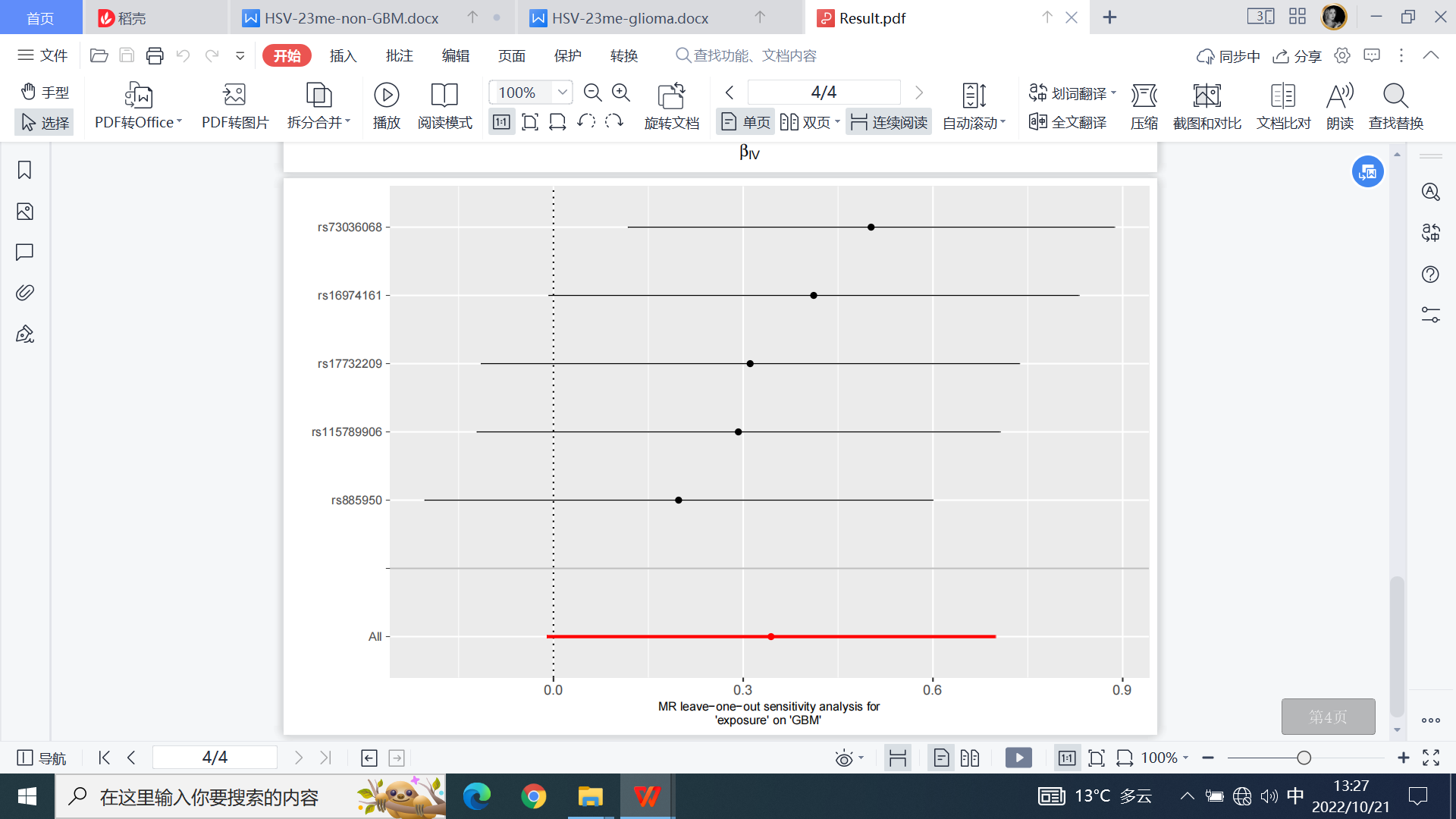


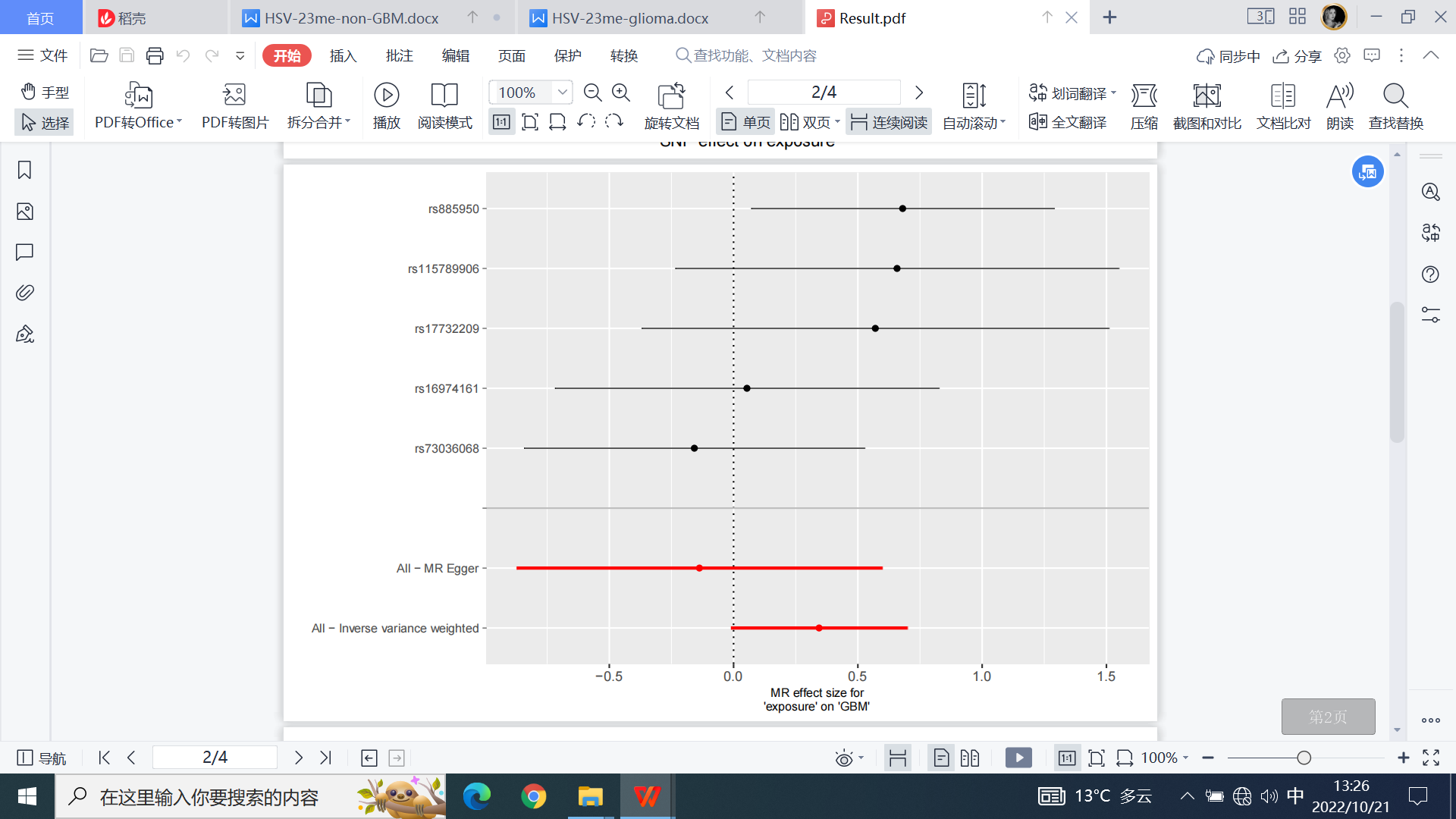


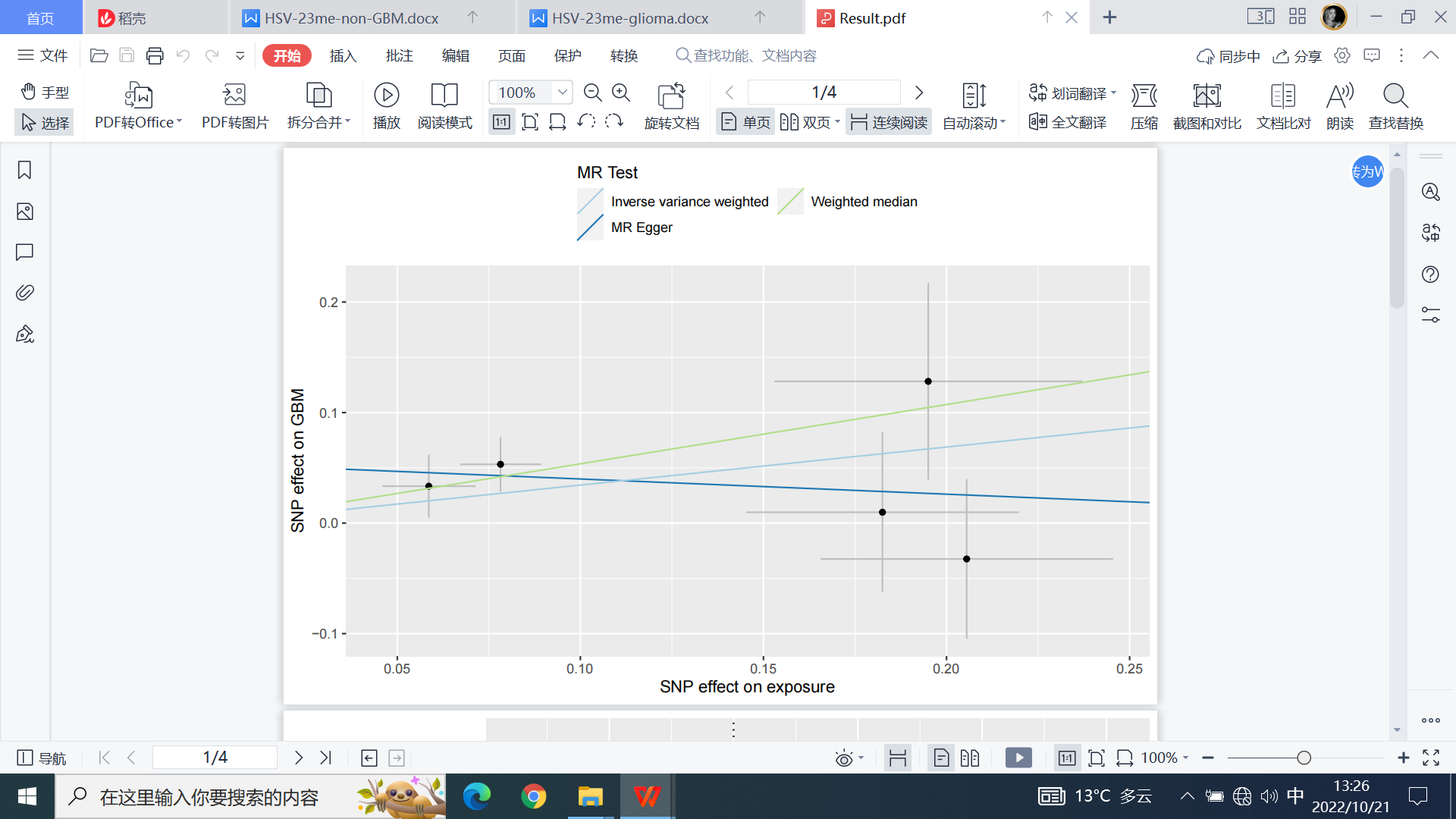


**Figure S6.** The leave-one-out plot, forest plot, and scatter plot for the association of HPV 16E7 infection and LGG in primary analysis. Data from SUHRE.


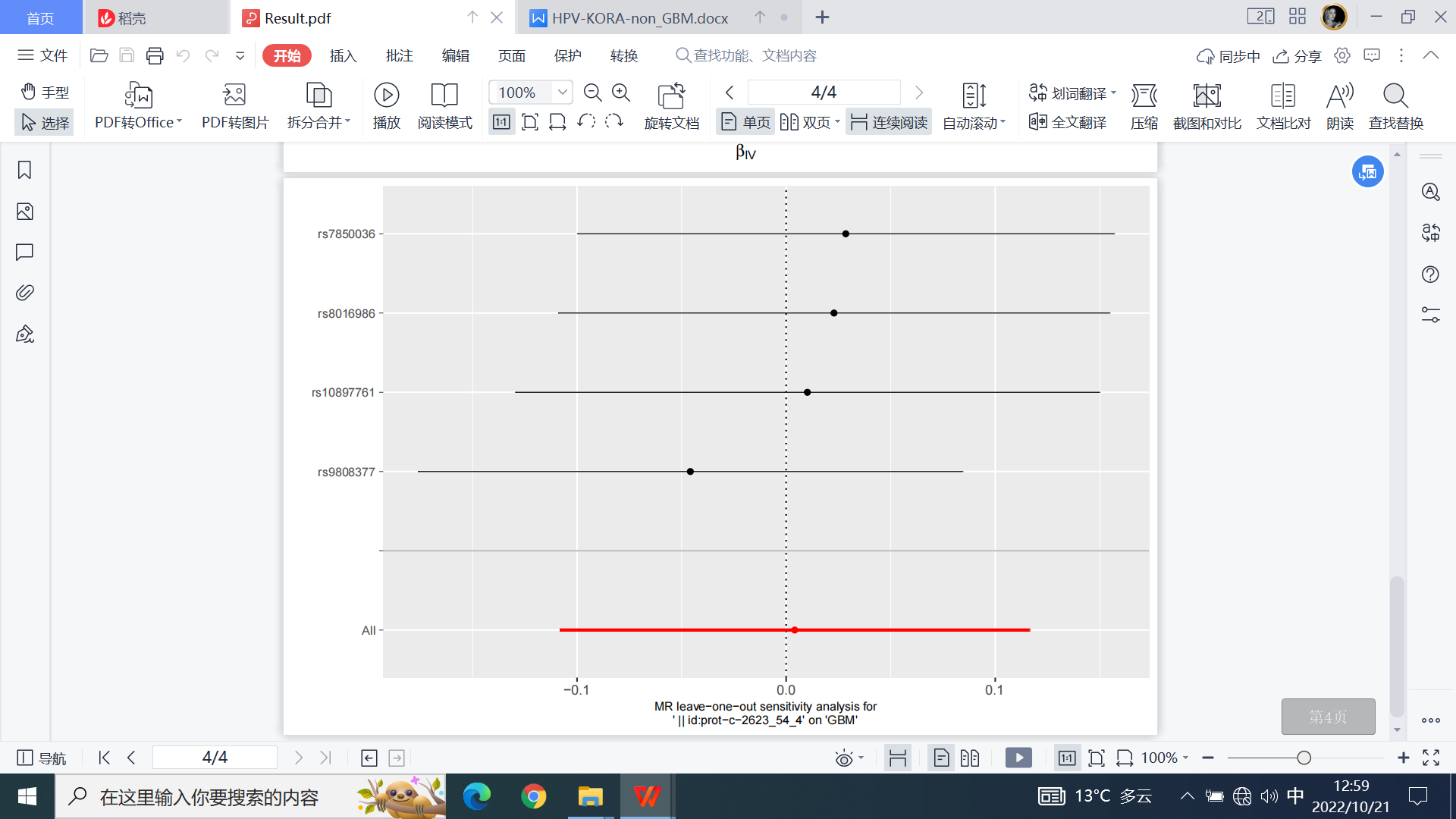


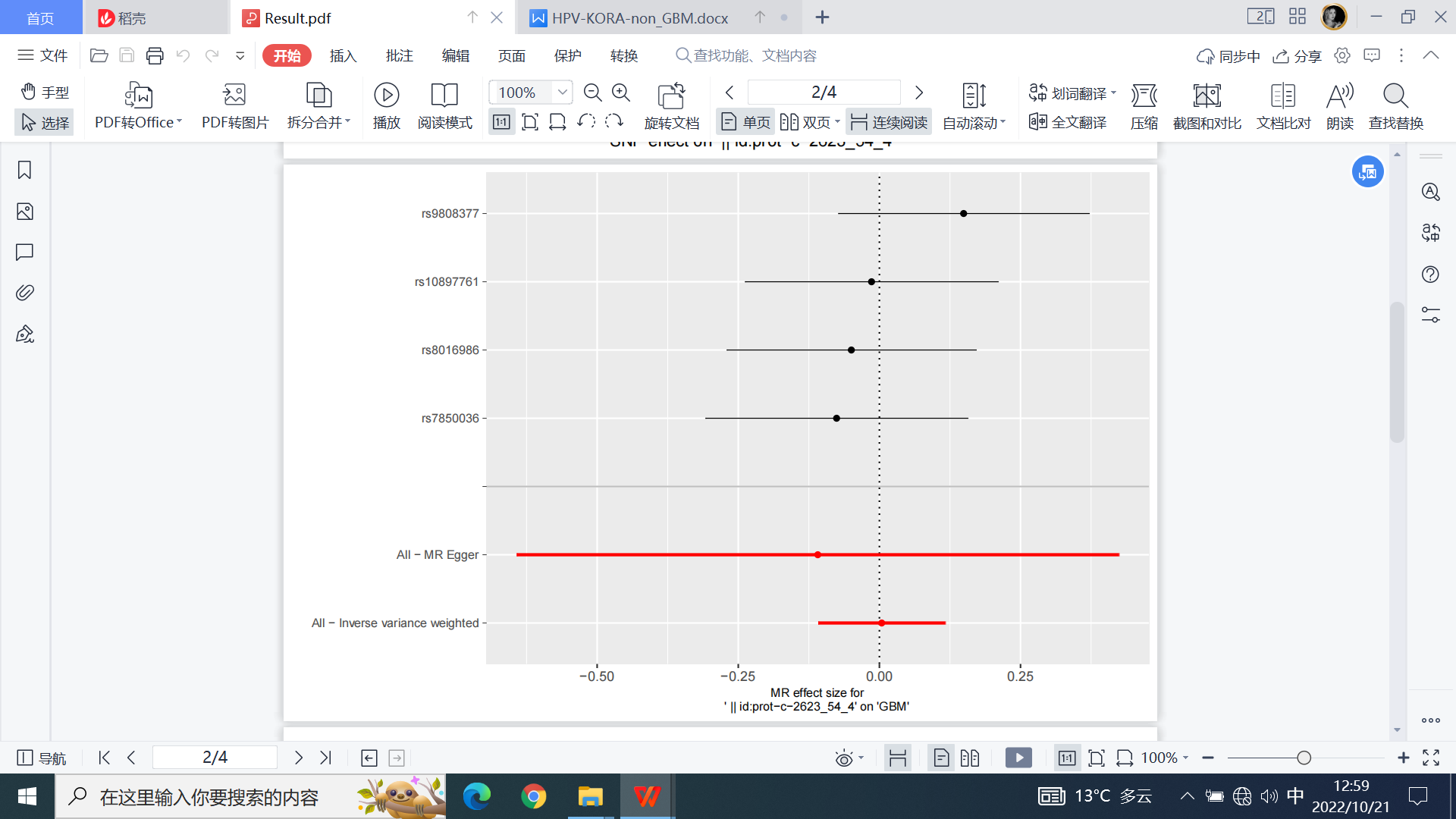


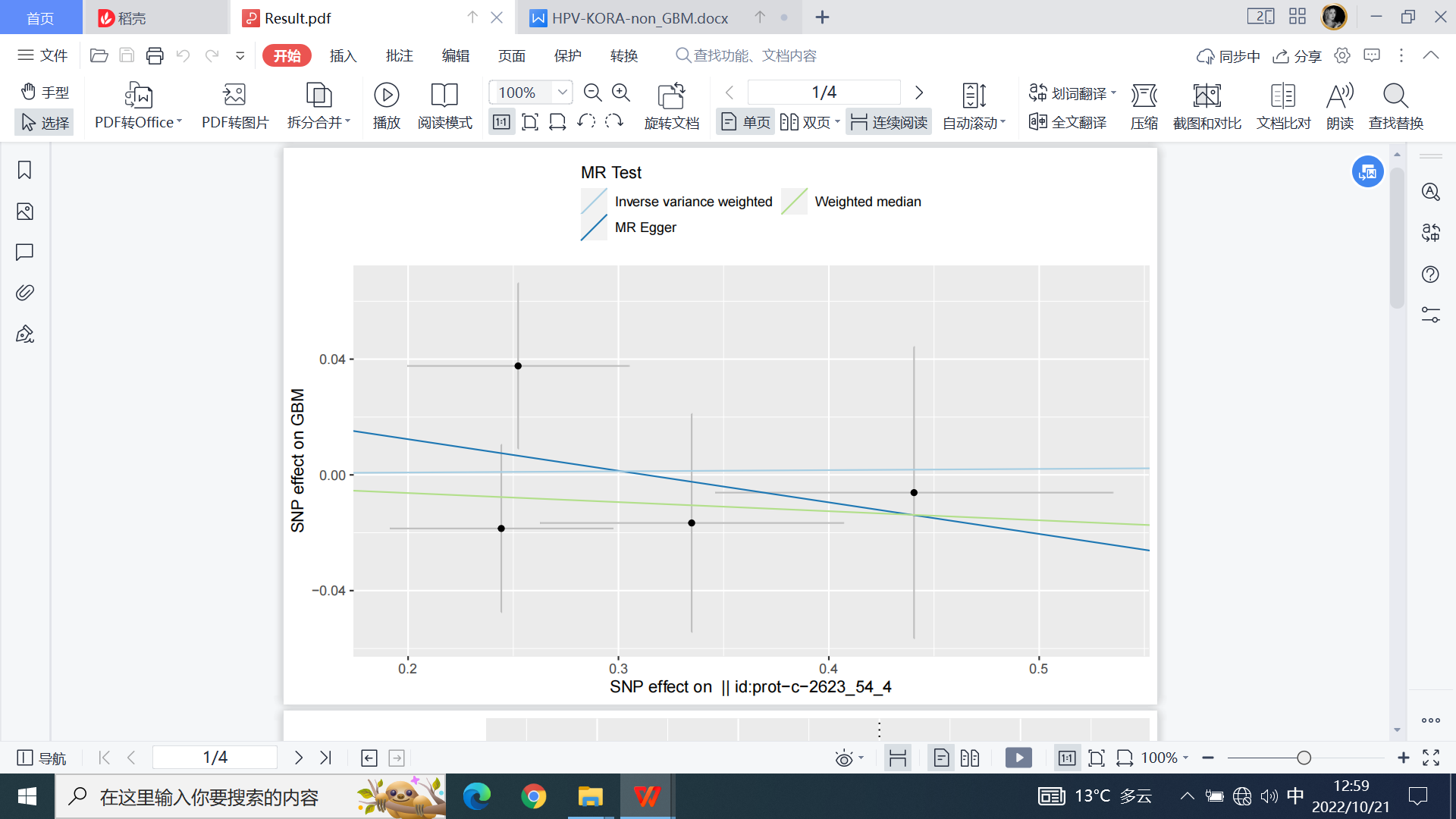


**Figure S7.** The leave-one-out plot, forest plot, and scatter plot for the association of EBV infection and LGG in primary analysis. Data from FINN.


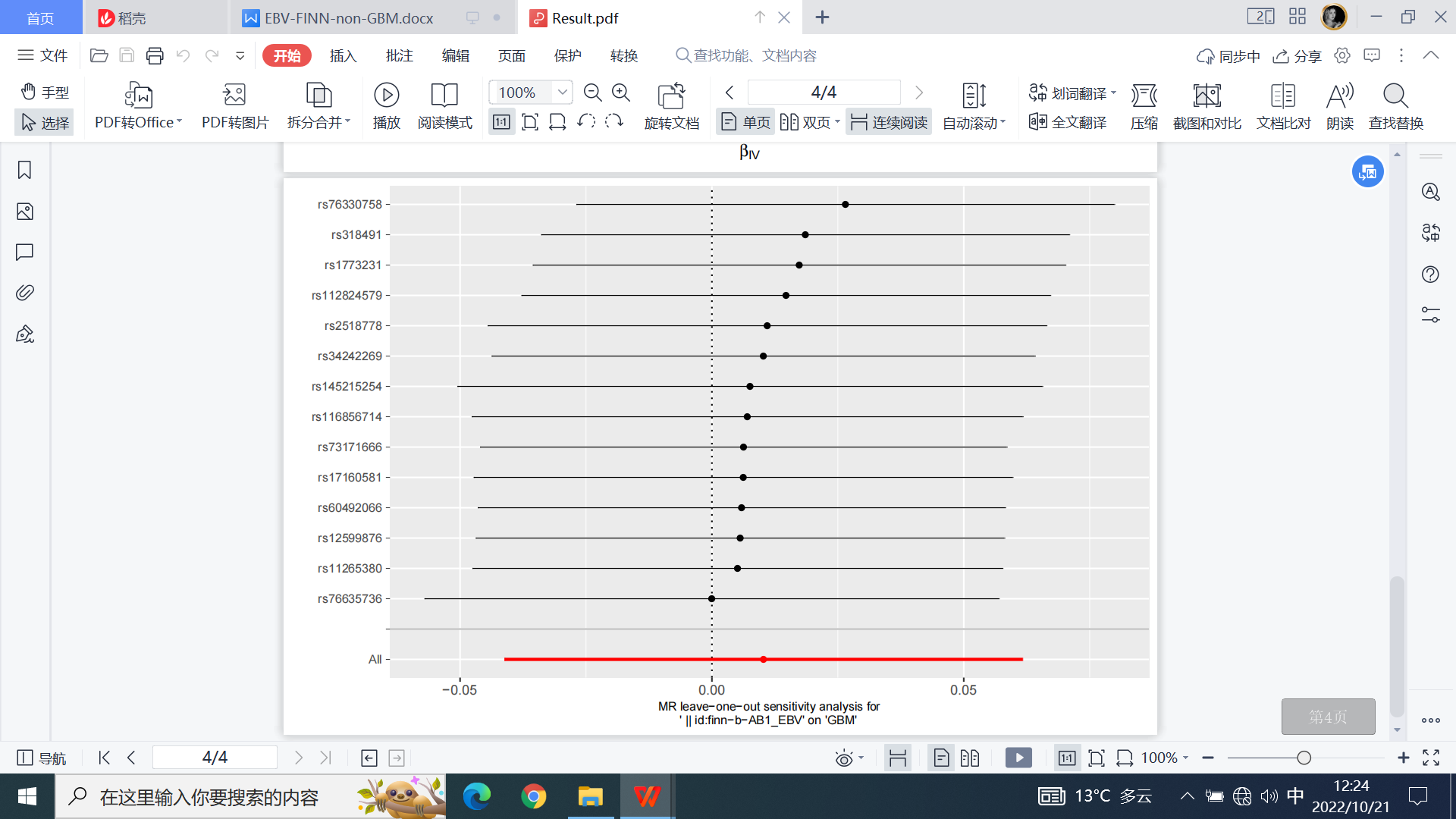


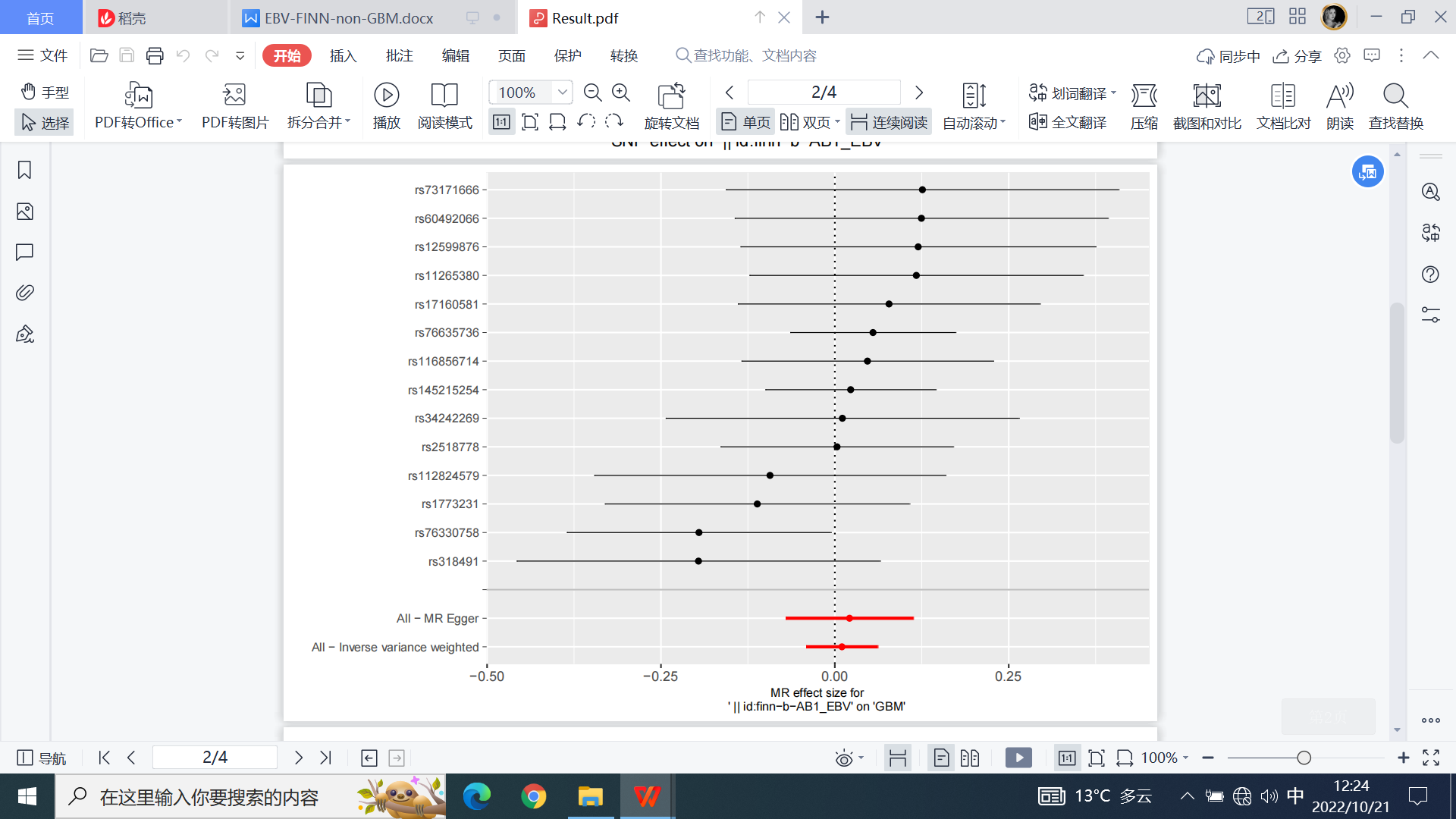


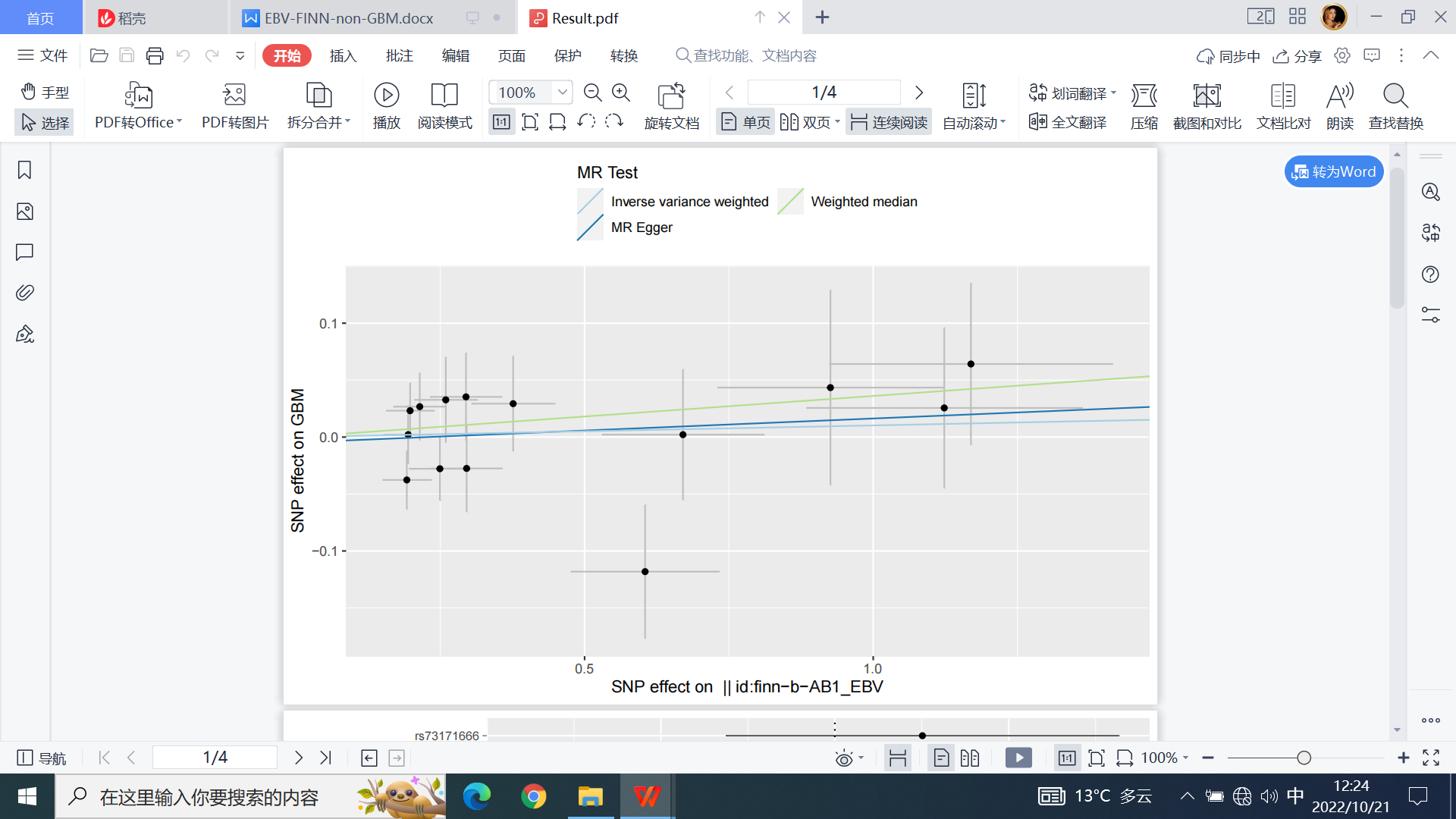


**Figure S8.** The leave-one-out plot, forest plot, and scatter plot for the association of EBV infection and LGG in primary analysis. Data from 23andme.


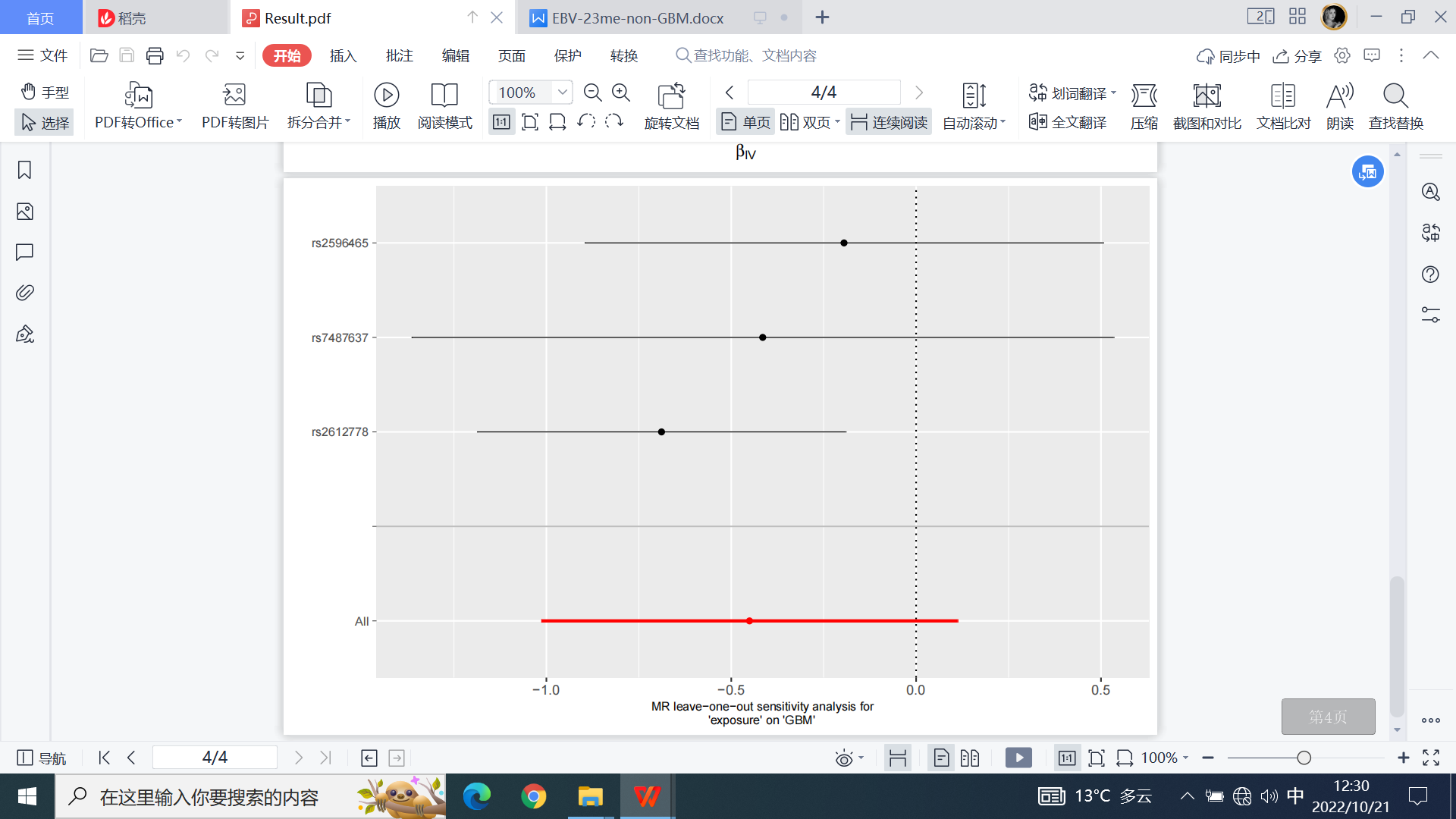


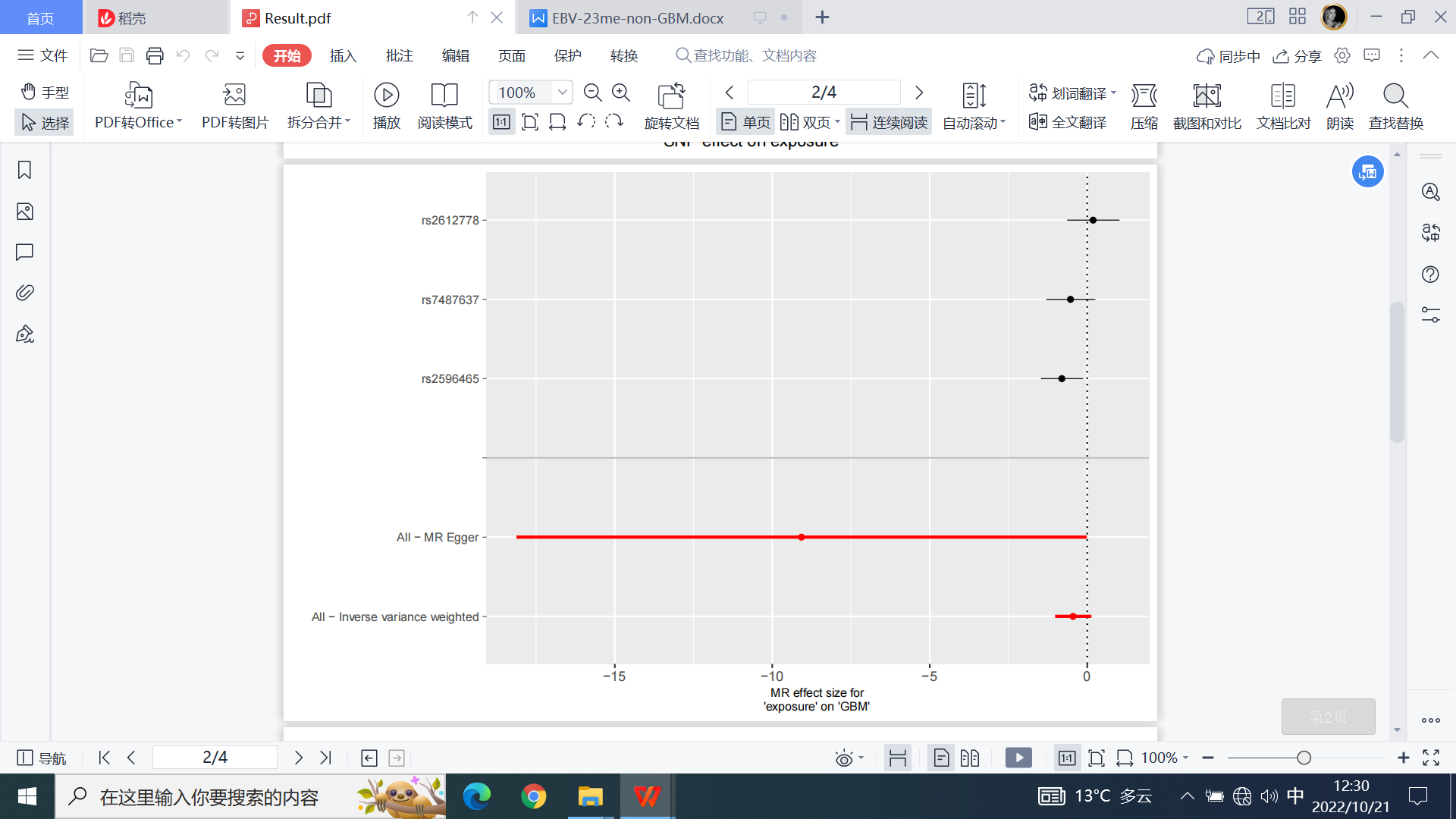


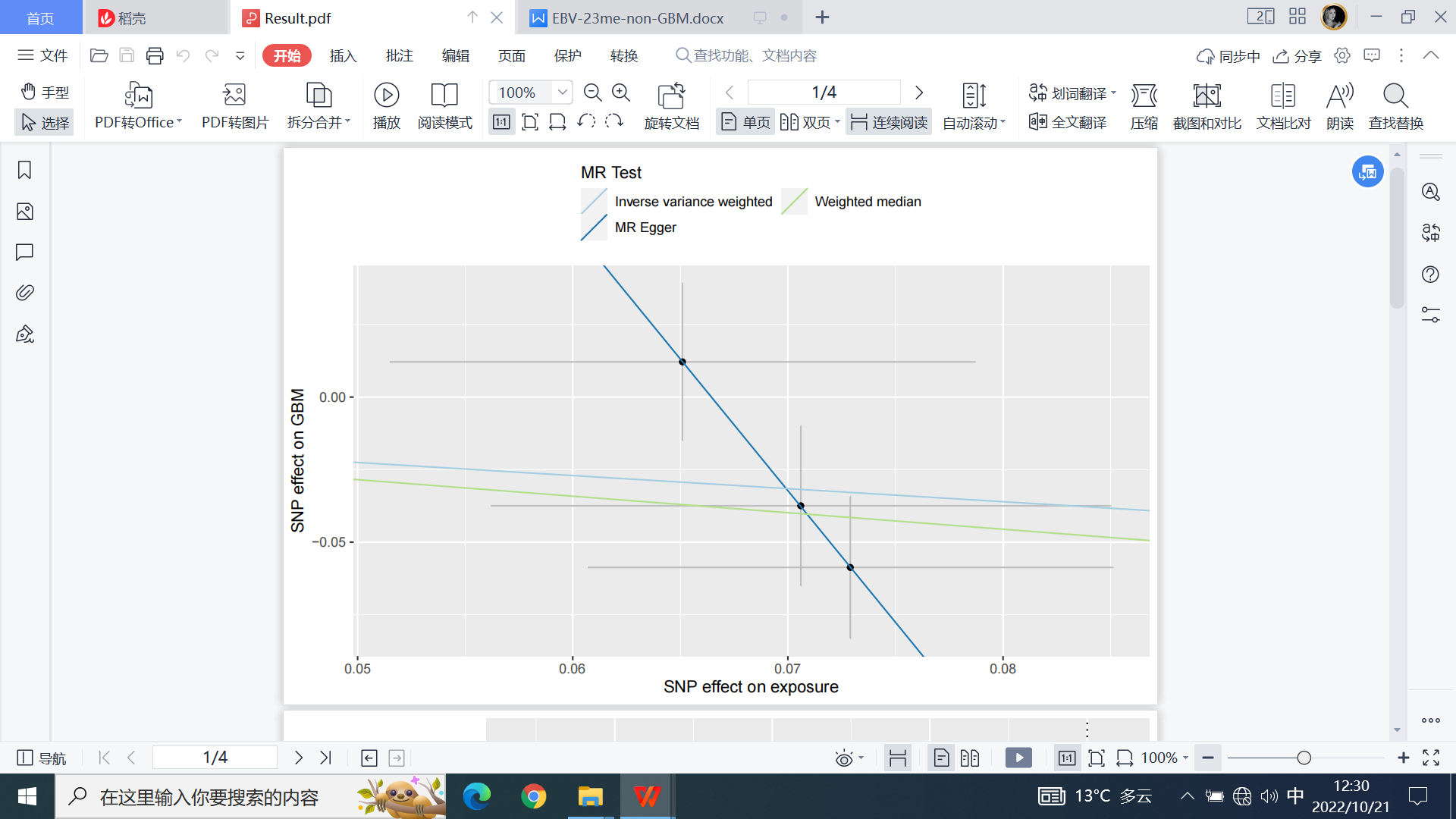


**Figure S9.** The leave-one-out plot, forest plot, and scatter plot for the association of COVID-19 infection and LGG in primary analysis. Data from COVID-19 HGI.


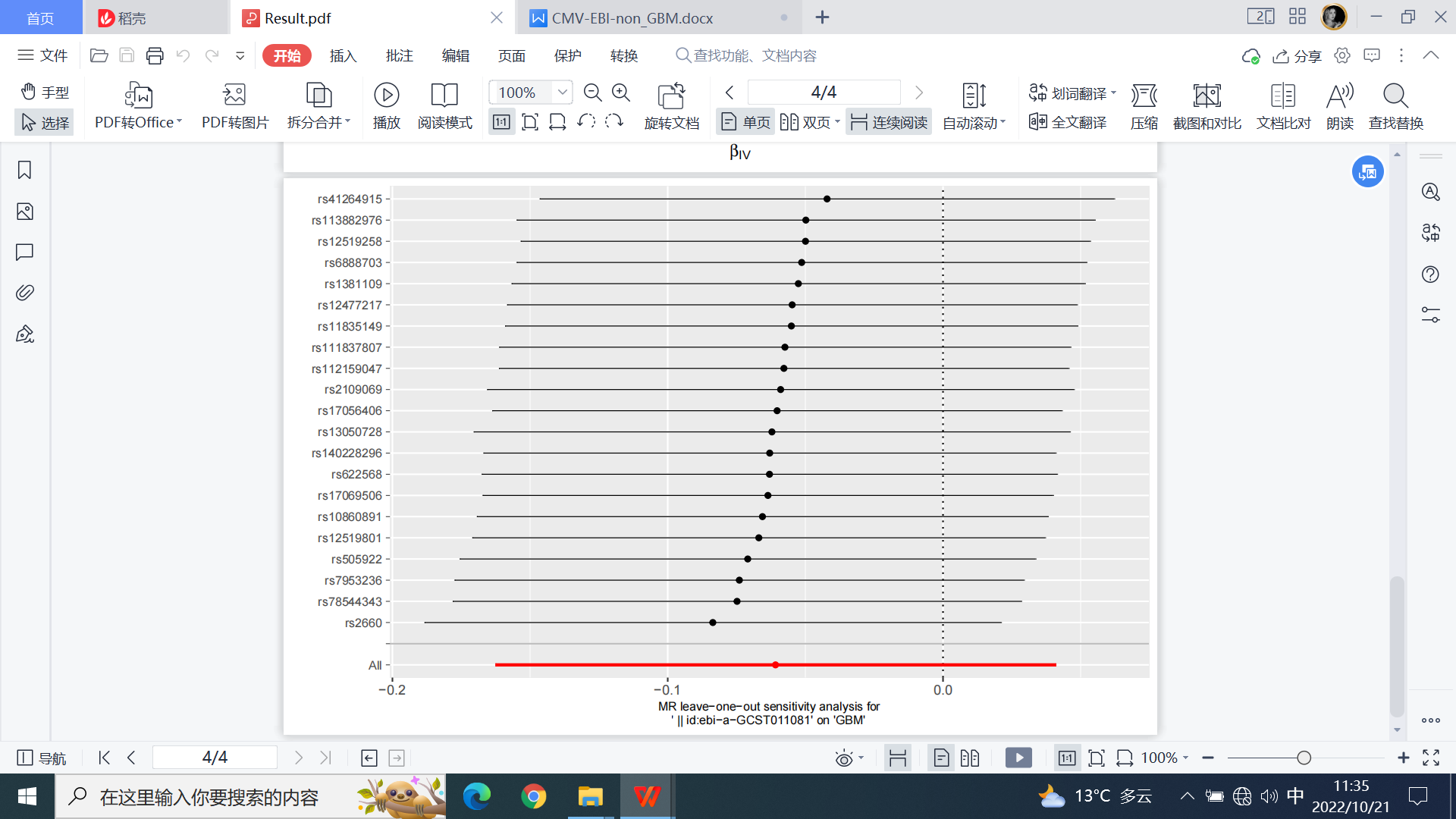


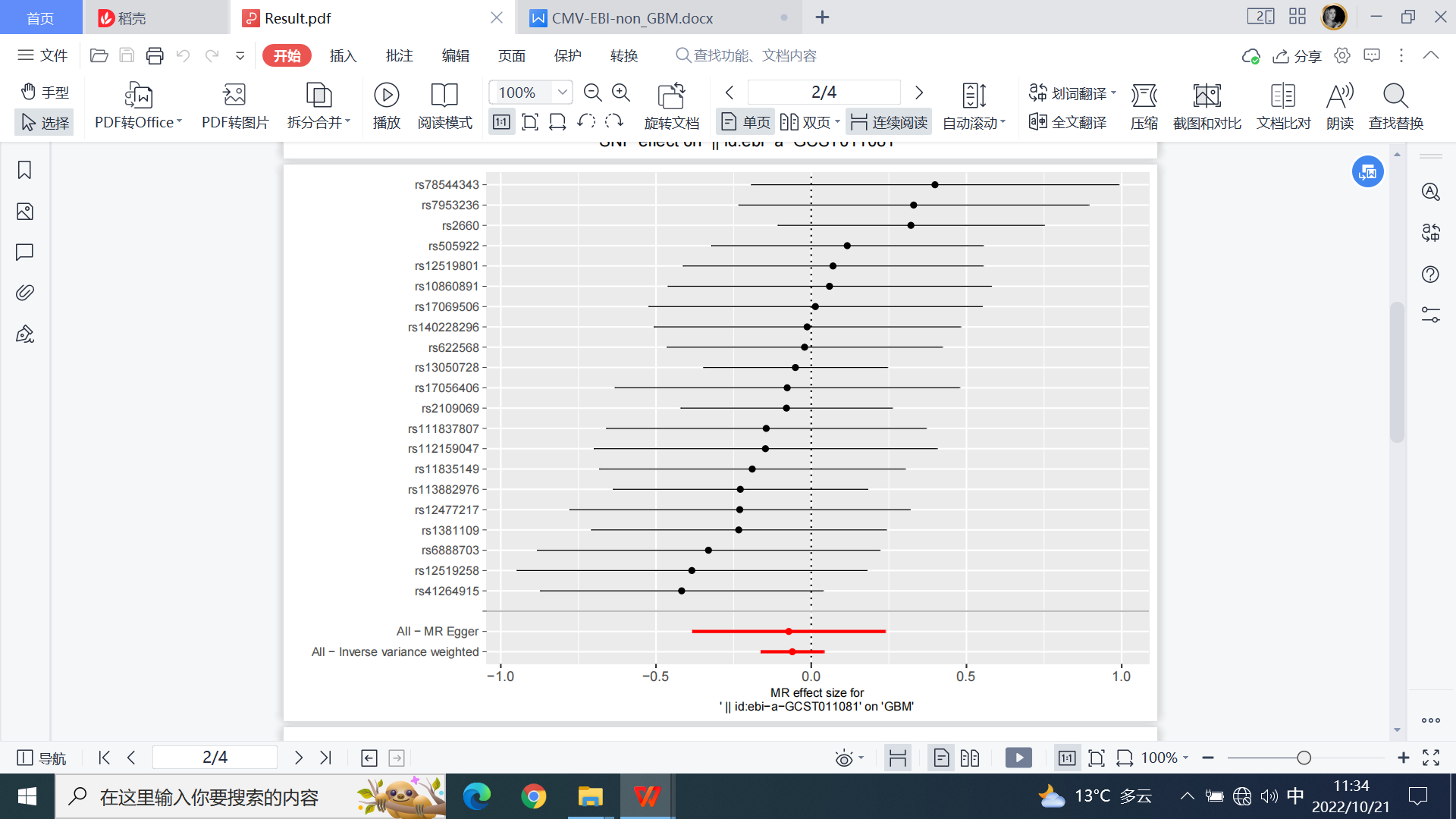


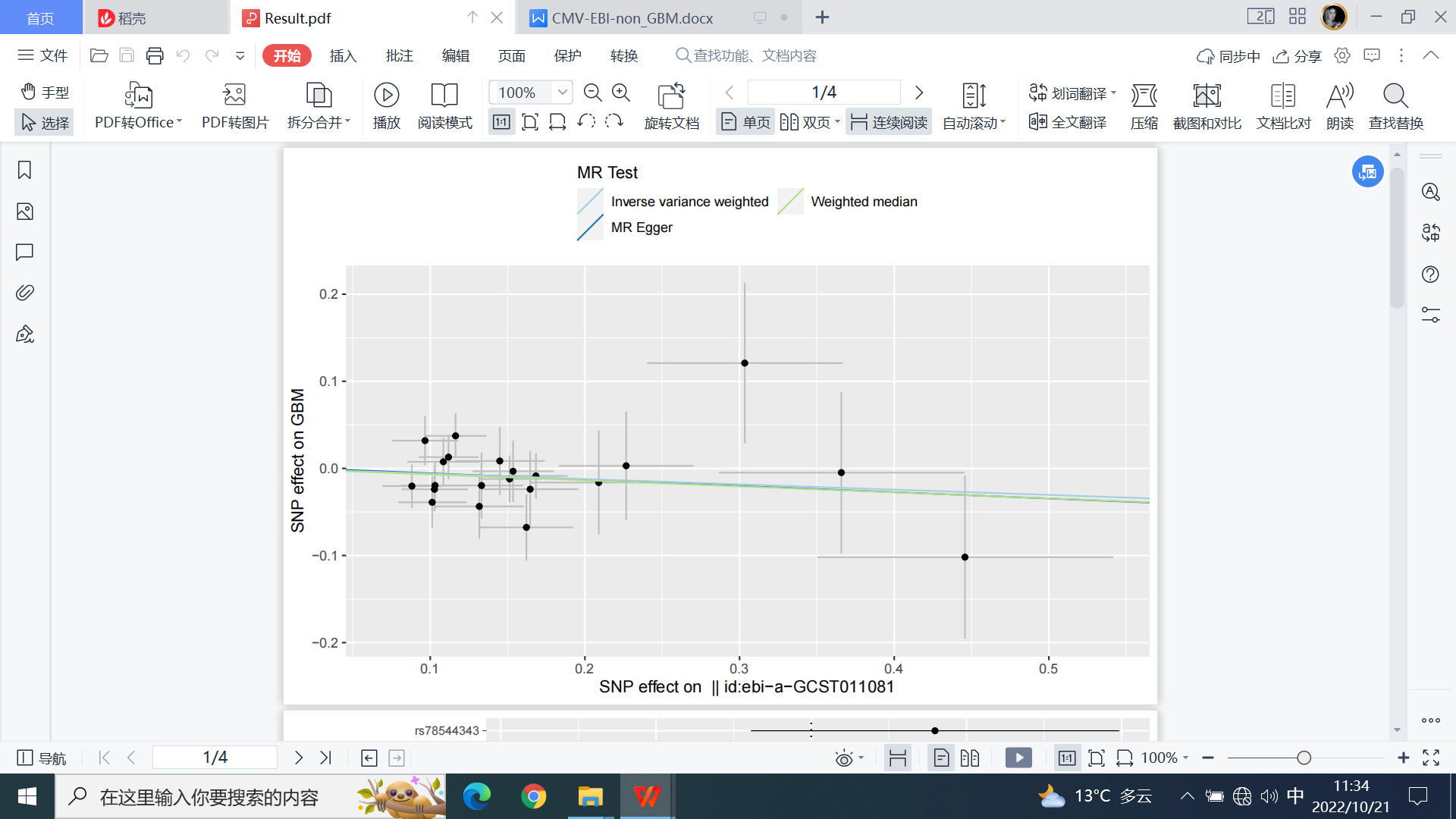


**Figure S10.** The leave-one-out plot, forest plot, and scatter plot for the association of HCMV infection and LGG in primary analysis. Data from FINN.


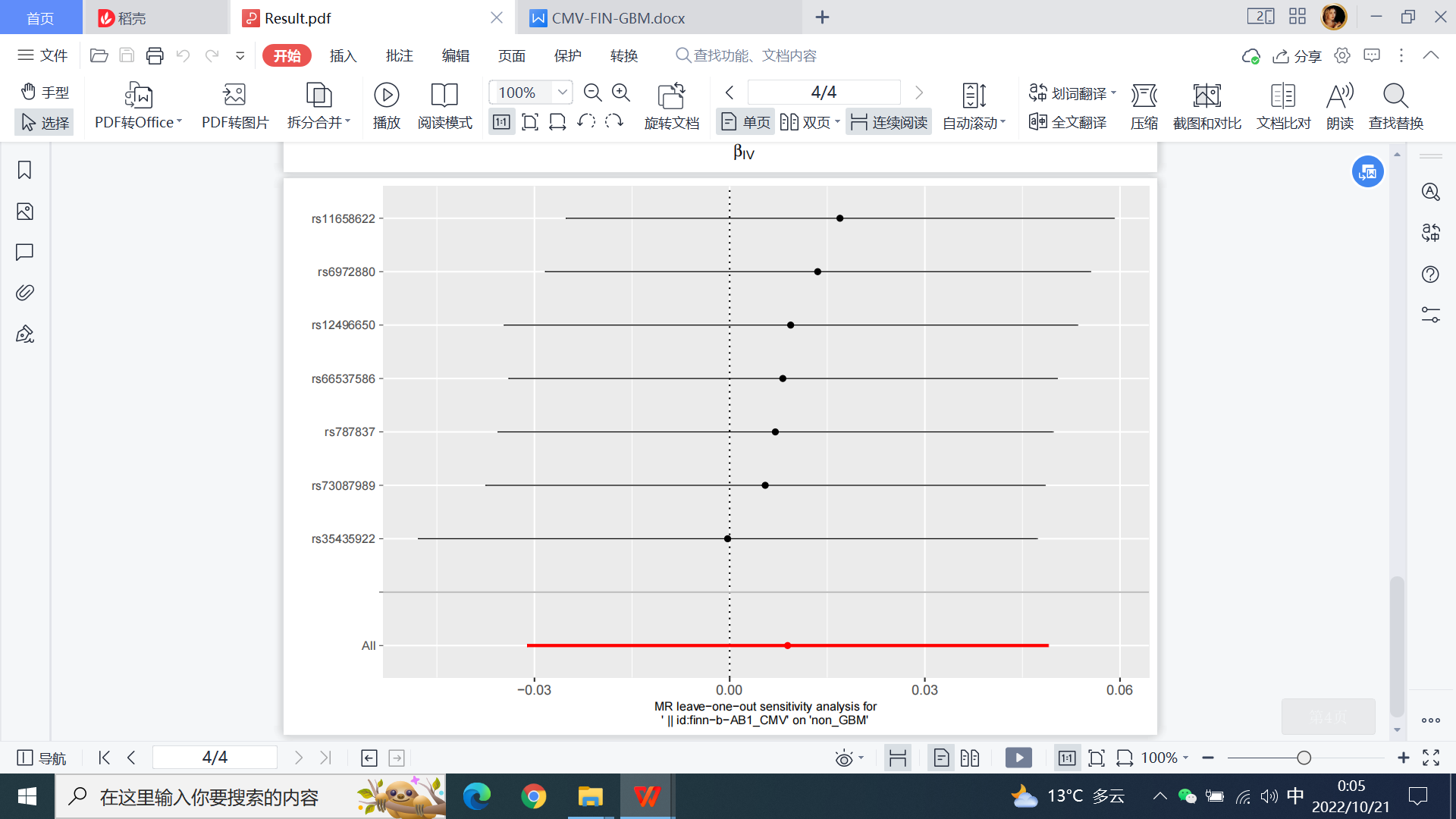


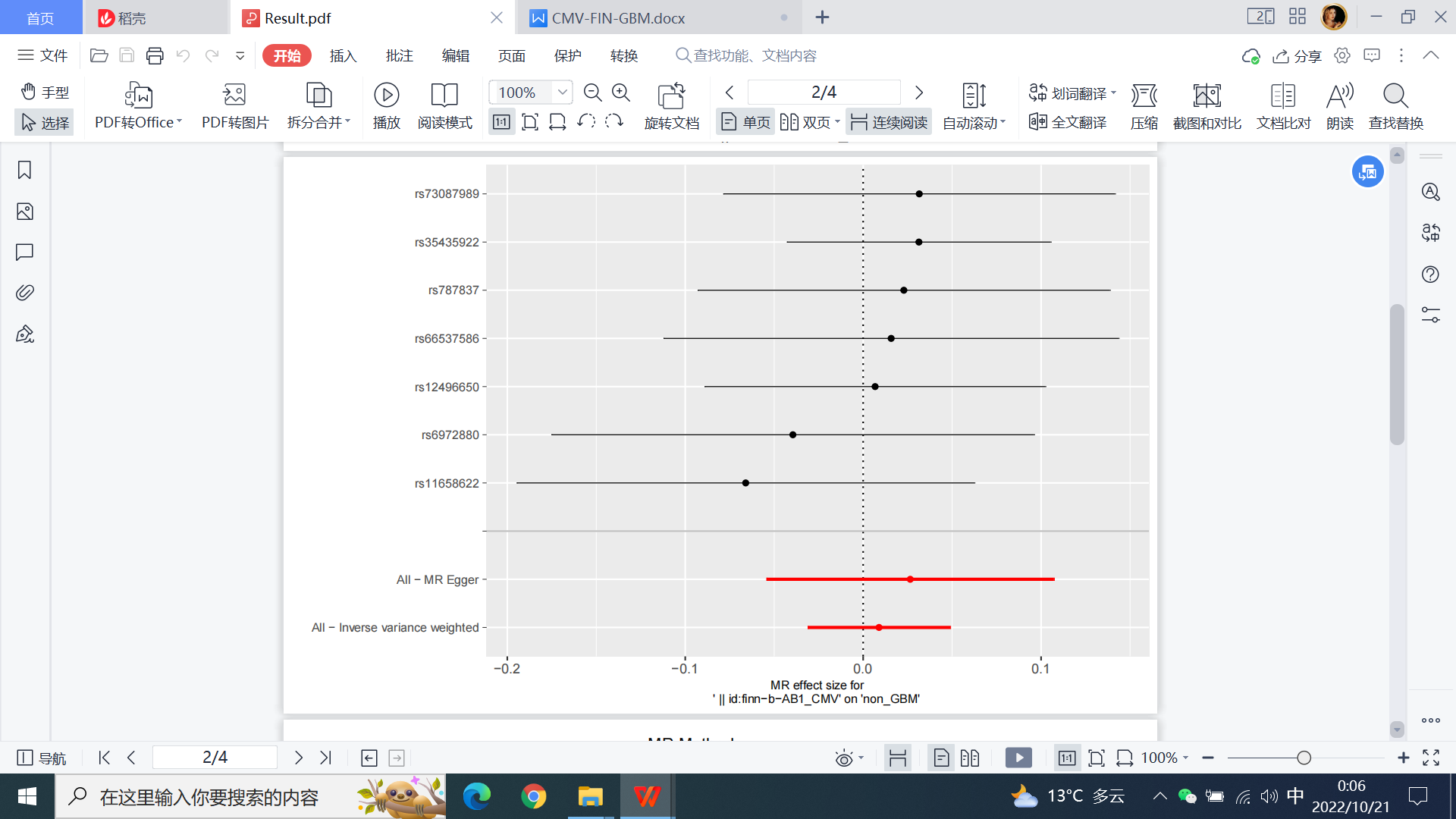


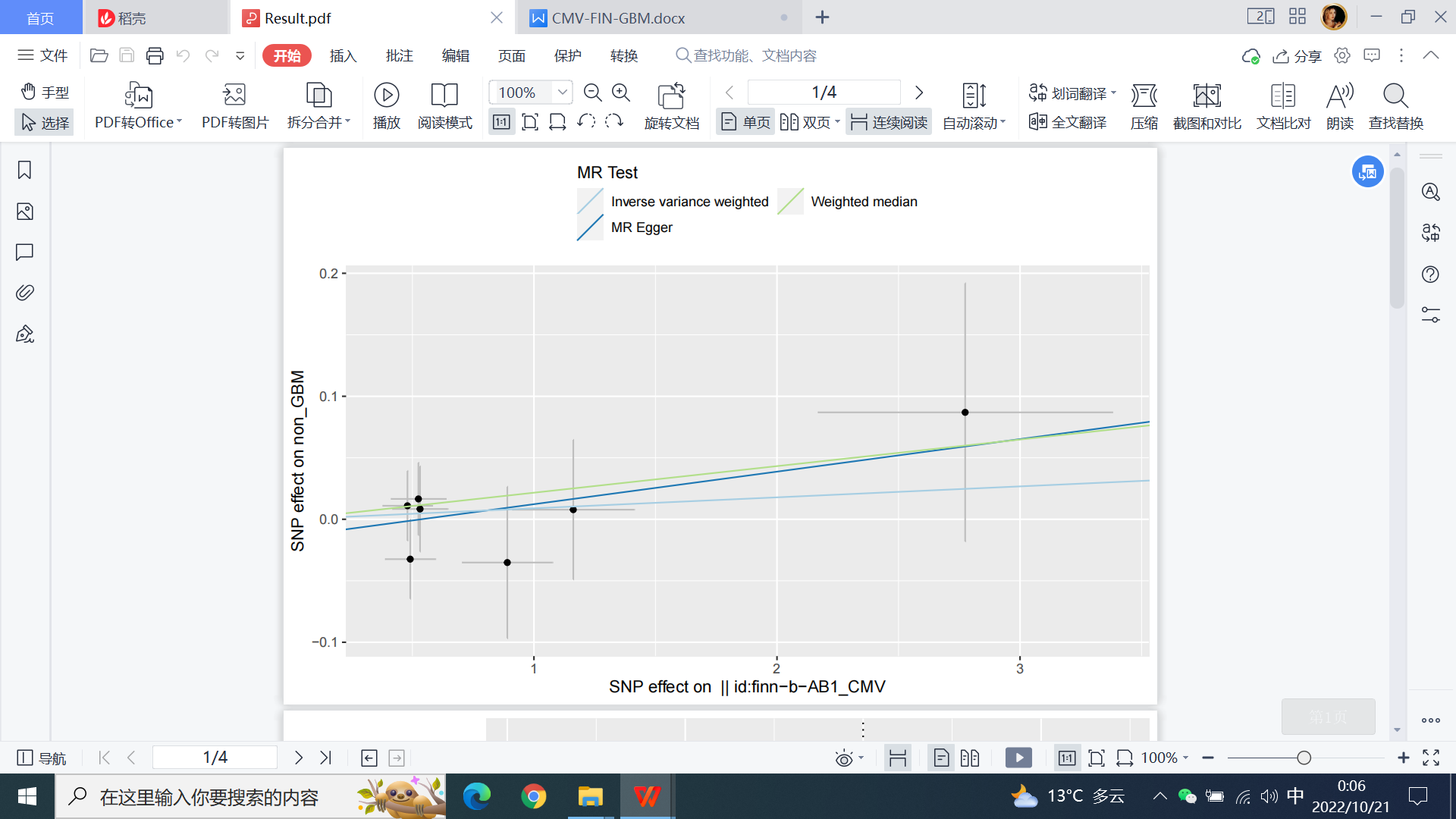


**Figure S11.** The leave-one-out plot, forest plot, and scatter plot for the association of Hepatitis infection and LGG in primary analysis. Data from FINN.


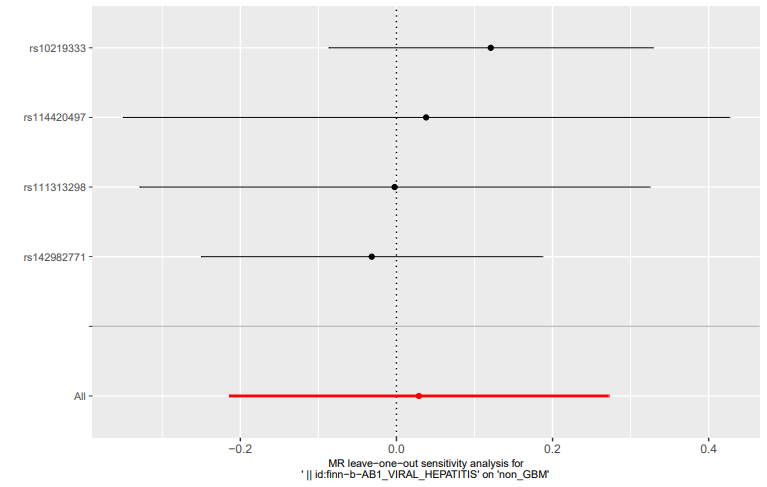


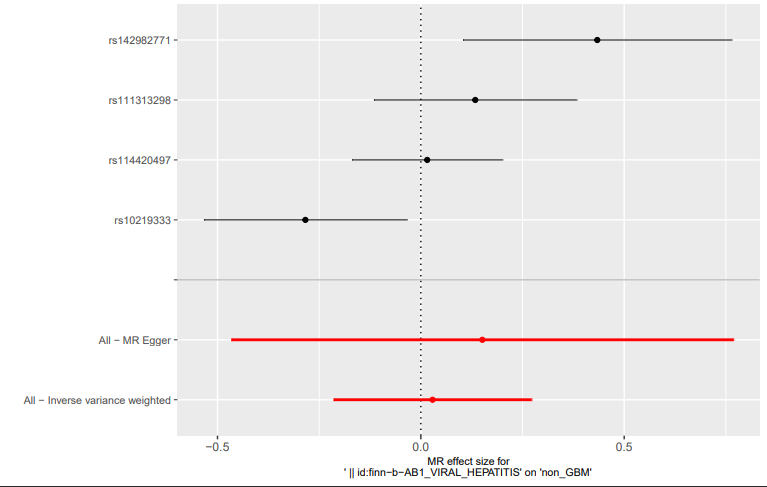


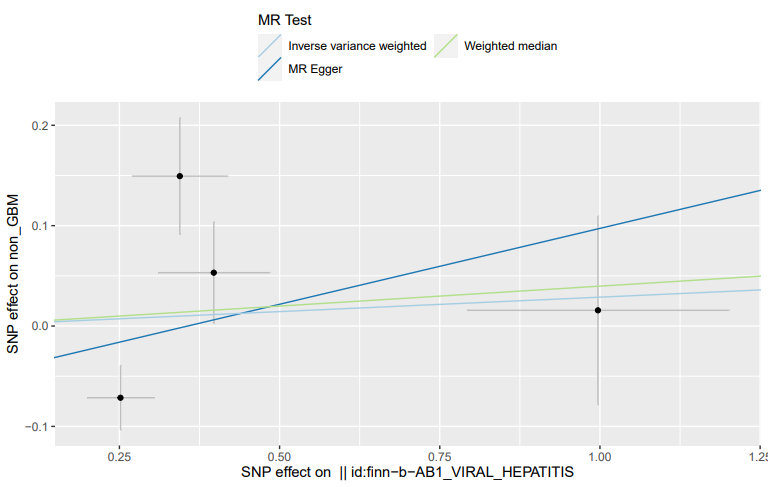


**Figure S12.** The leave-one-out plot, forest plot, and scatter plot for the association of Hepatitis infection and LGG in primary analysis. Data from 23andme.


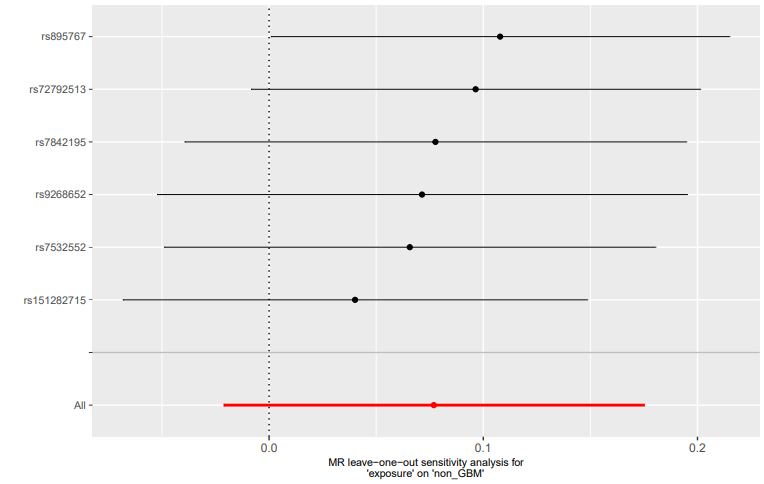


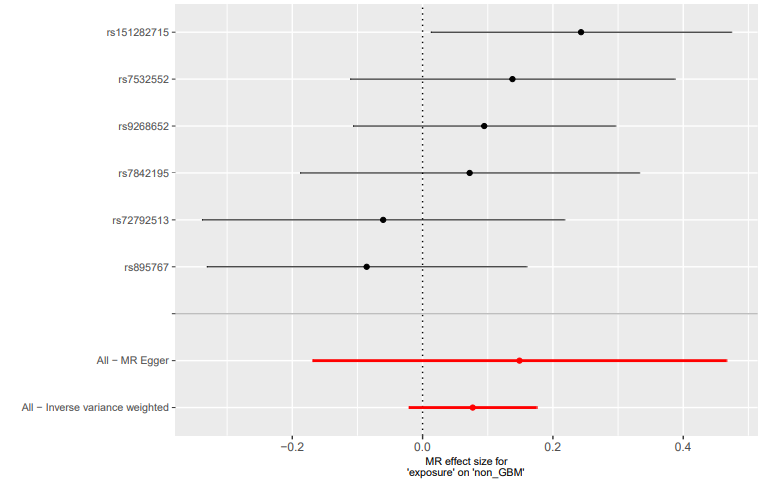


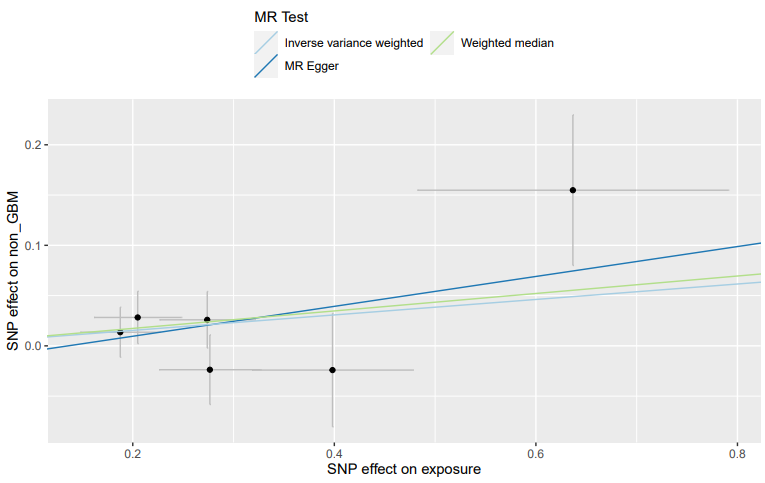


**Figure S13.** The leave-one-out plot, forest plot, and scatter plot for the association of HIV infection and LGG in primary analysis. Data from FINN.


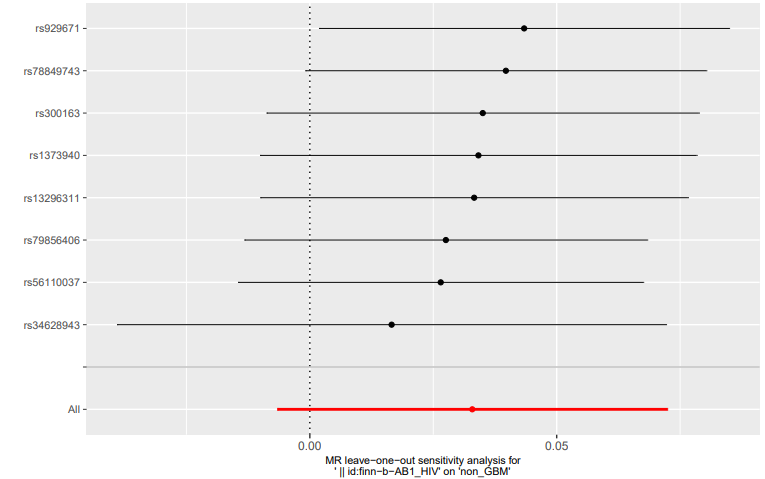


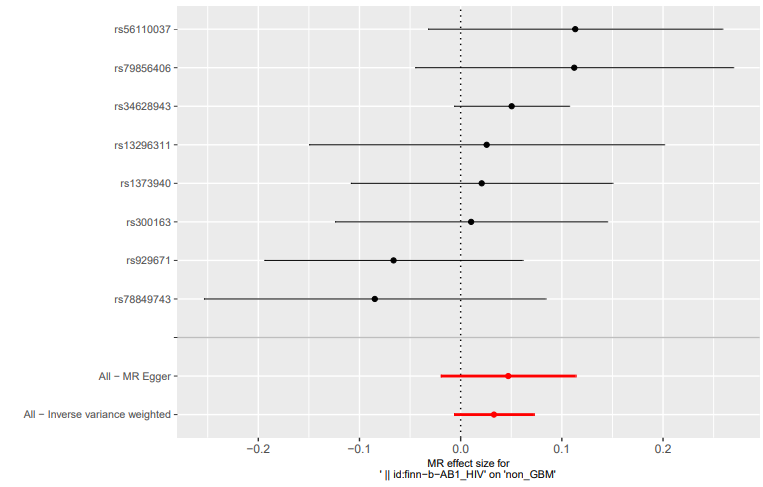


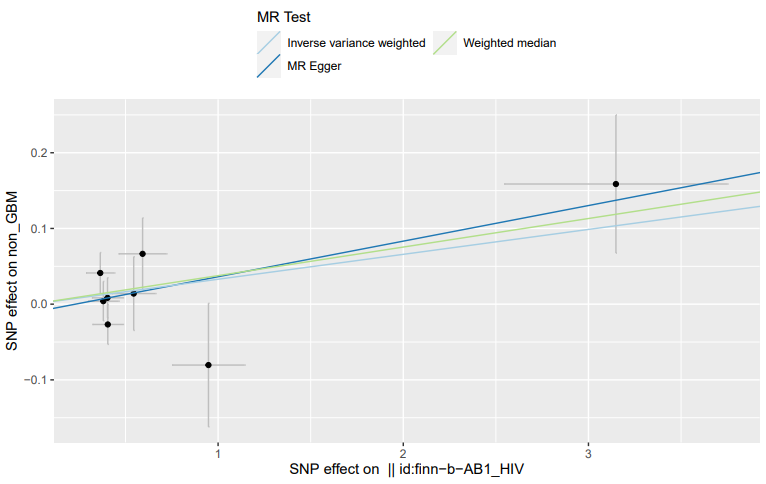


**Figure S14.** The leave-one-out plot, forest plot, and scatter plot for the association of measles infection and LGG in primary analysis. Data from FINN.


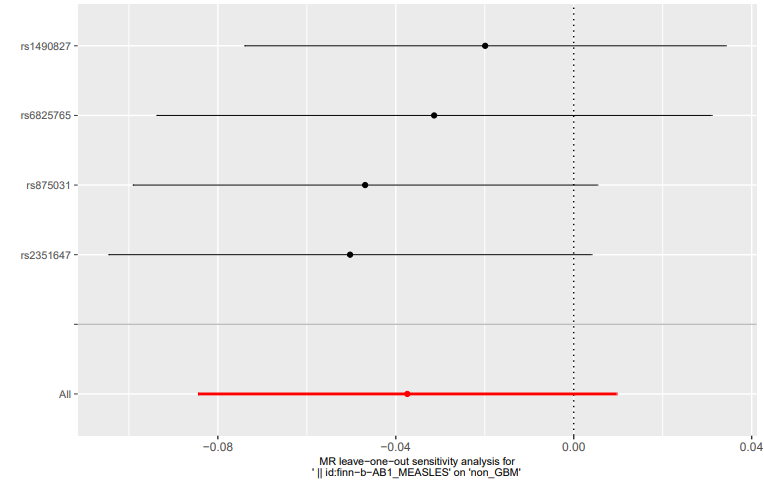


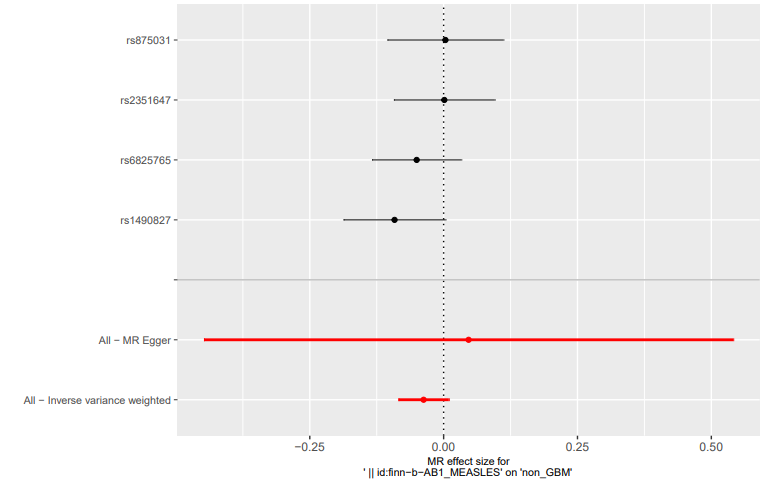


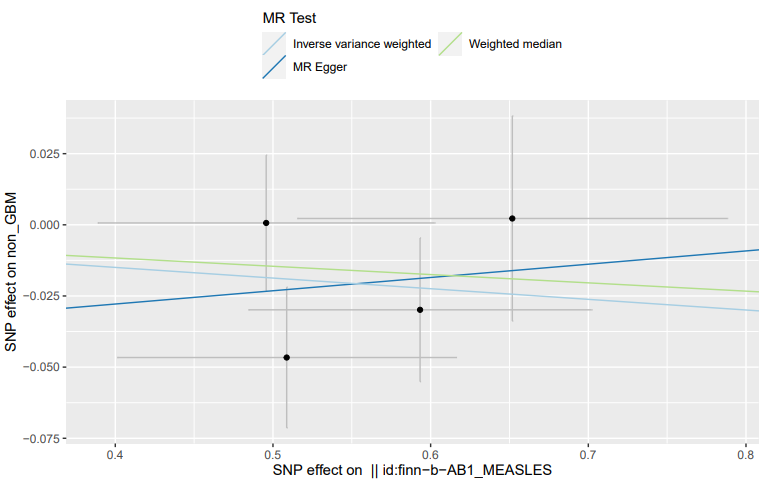


**Figure S15.** The leave-one-out plot, forest plot, and scatter plot for the association of measles infection and LGG in primary analysis. Data from 23andme.


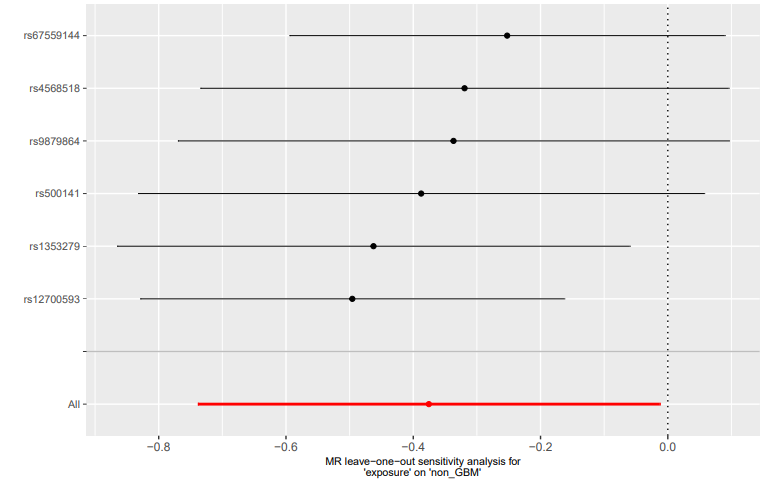


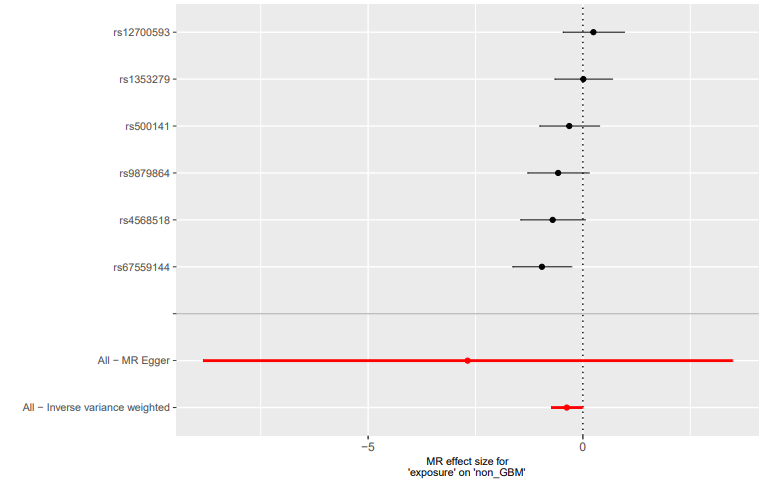


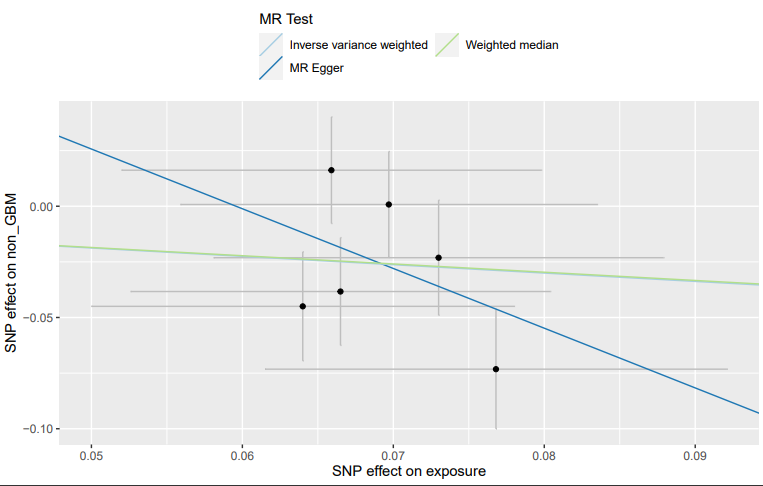


**Figure S16.** The leave-one-out plot, forest plot, and scatter plot for the association of Poliovirus infection and LGG in primary analysis. Data from FINN.


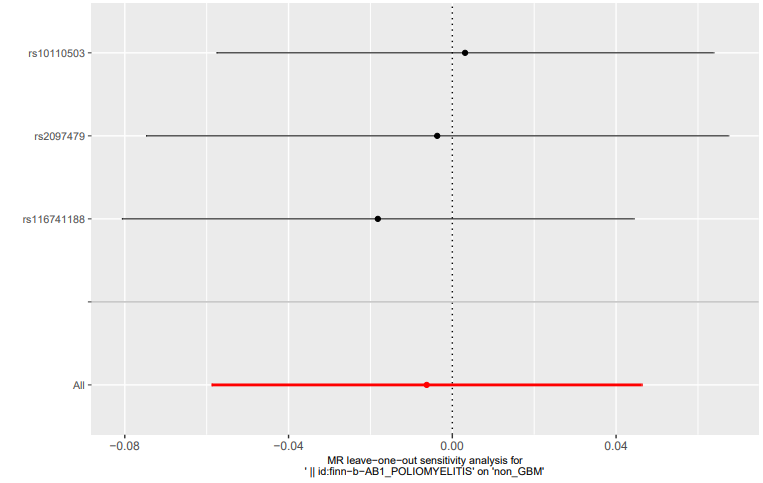


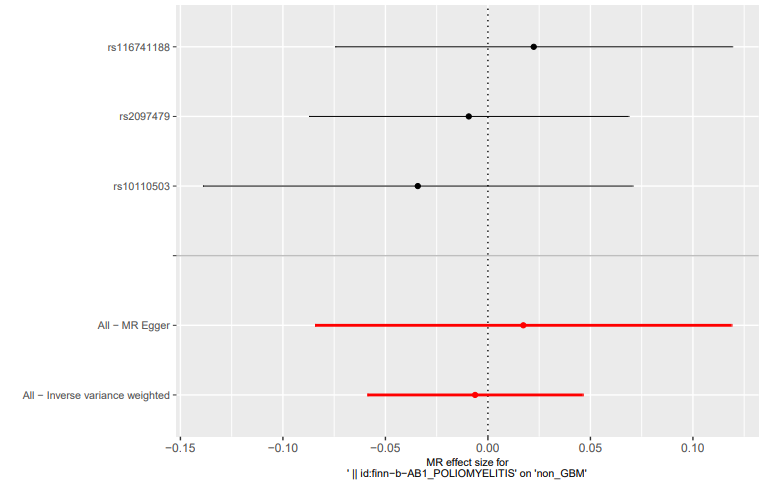


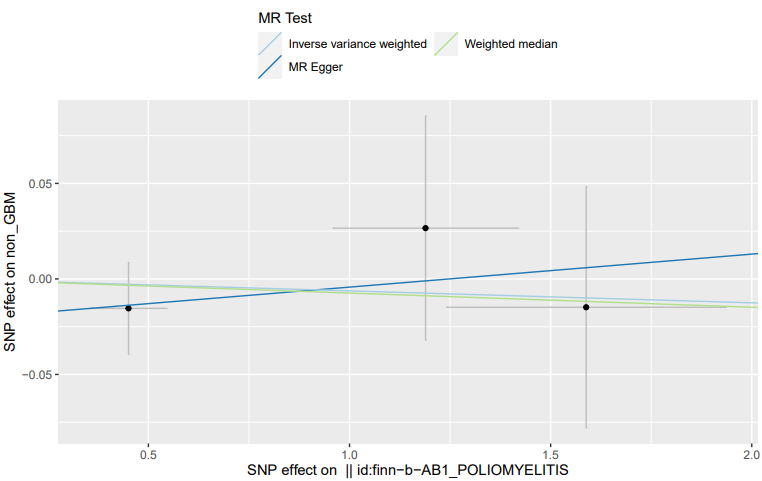


**Figure S17.** The leave-one-out plot, forest plot, and scatter plot for the association of Rubella infection and LGG in primary analysis. Data from FINN.


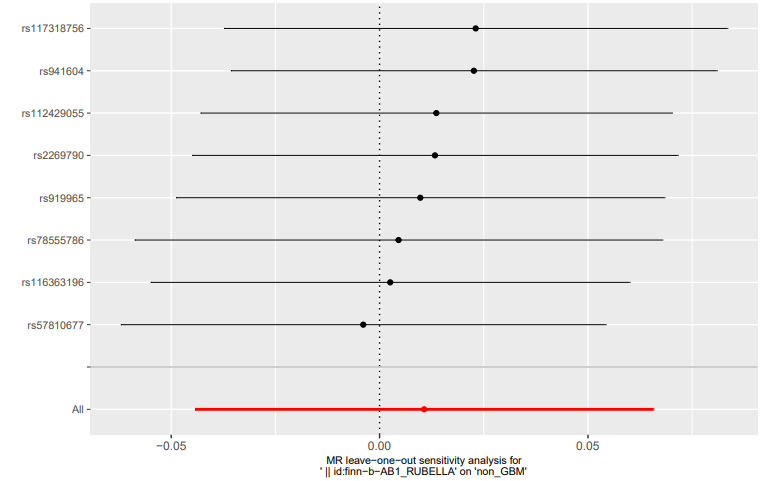


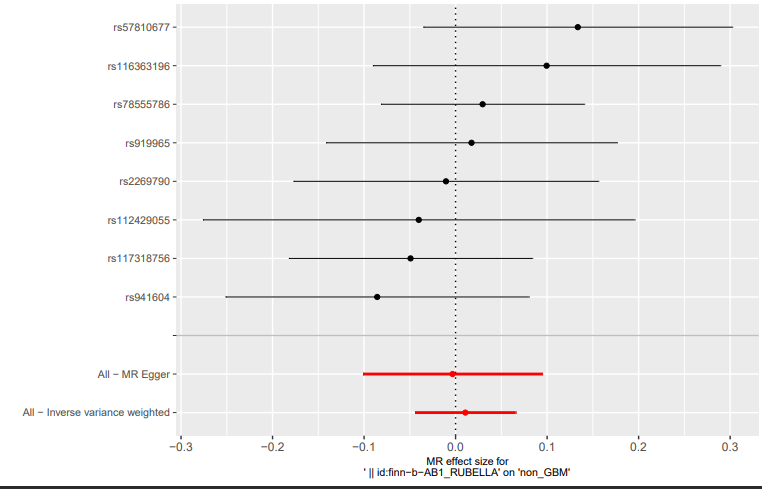


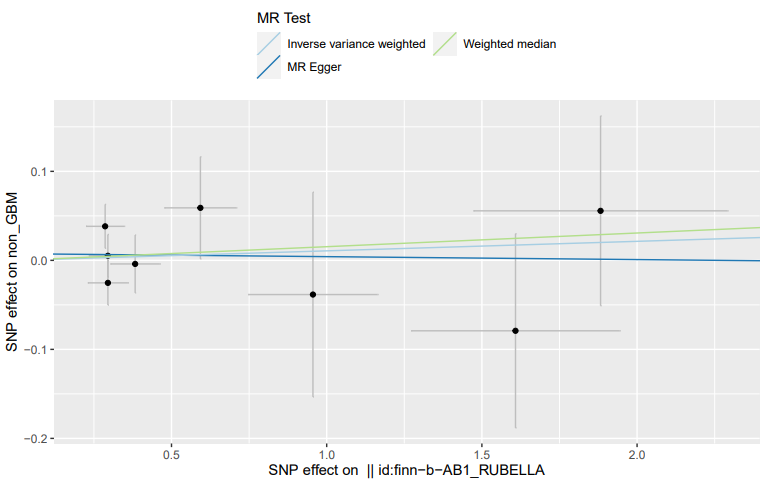


**Figure S18.** The leave-one-out plot, forest plot, and scatter plot for the association of Rubella infection and LGG in primary analysis. Data from 23andme.


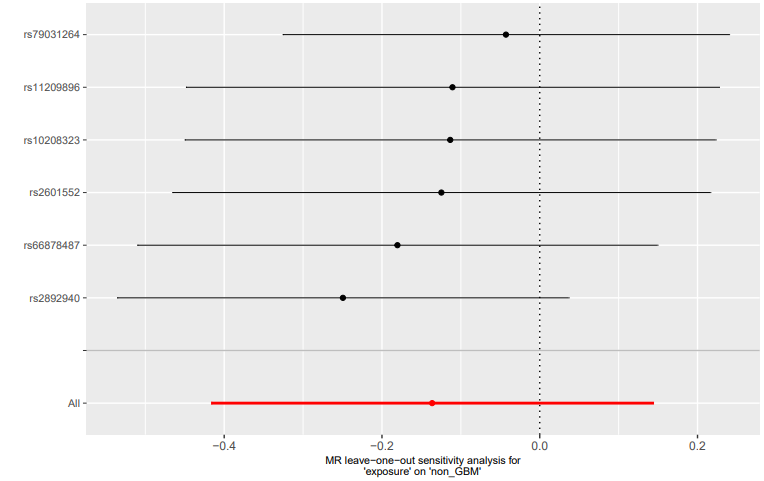


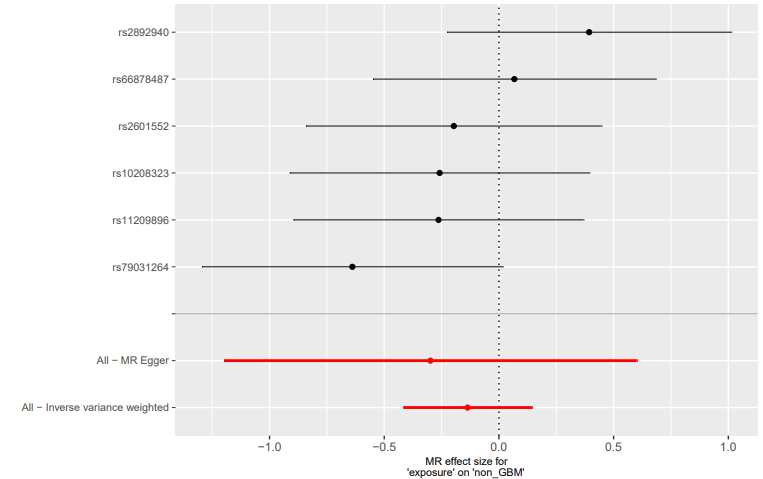


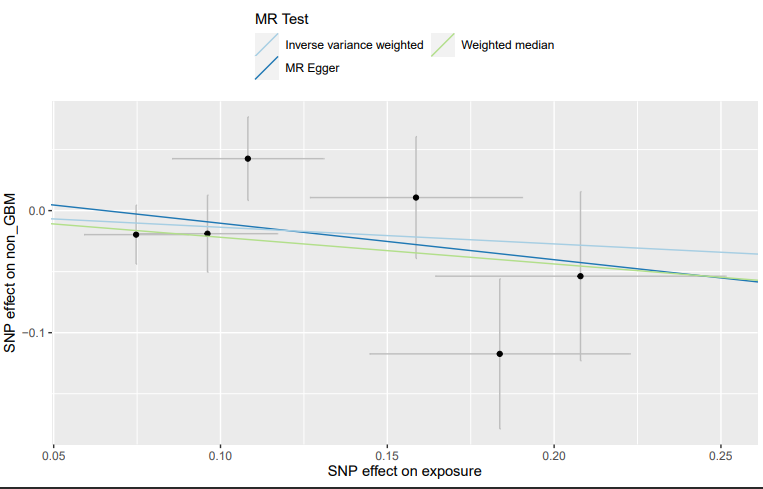


**Figure S19.** The leave-one-out plot, forest plot, and scatter plot for the association of Herpes zoster and GBM in primary analysis. Data from FINN.


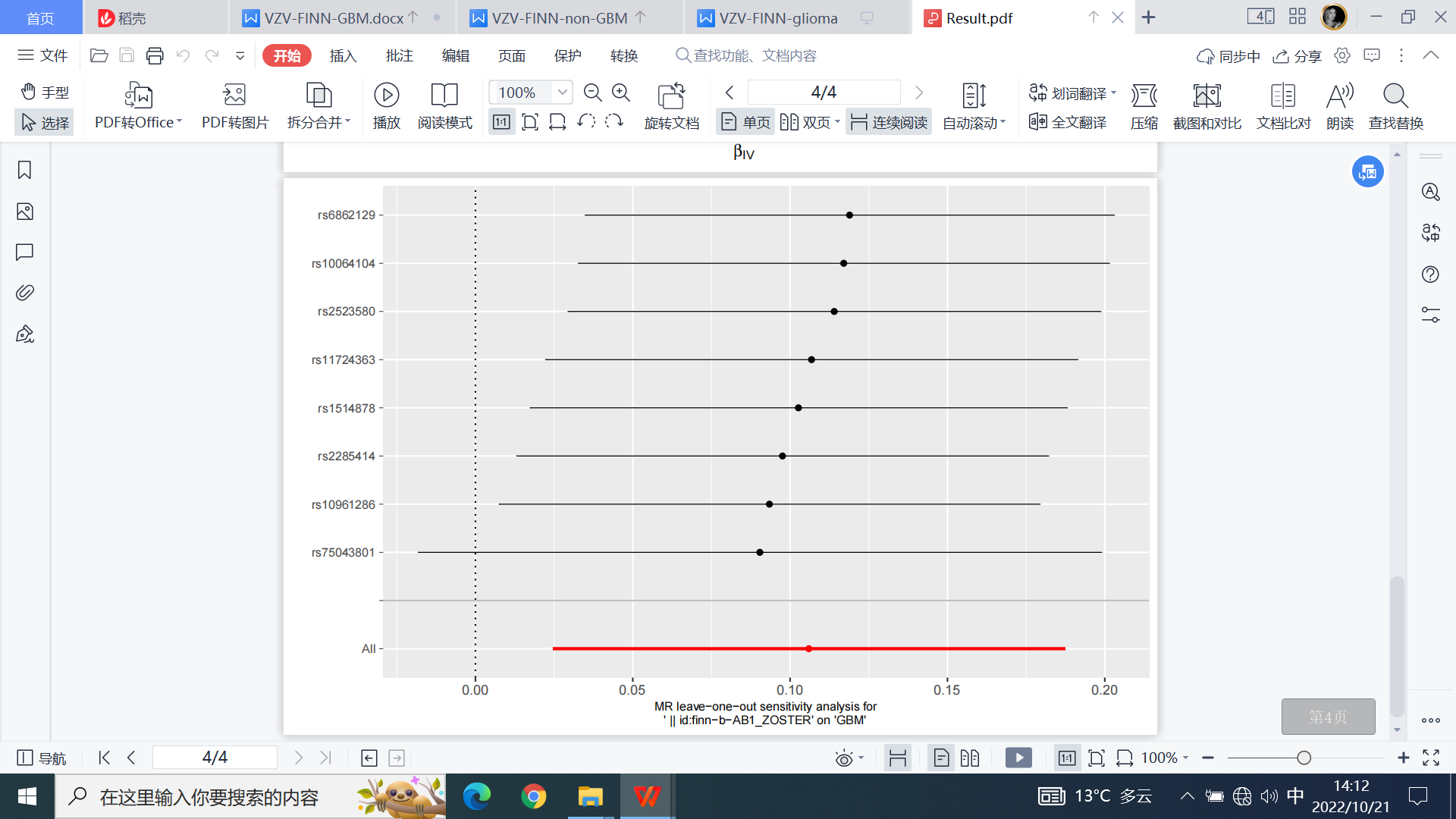


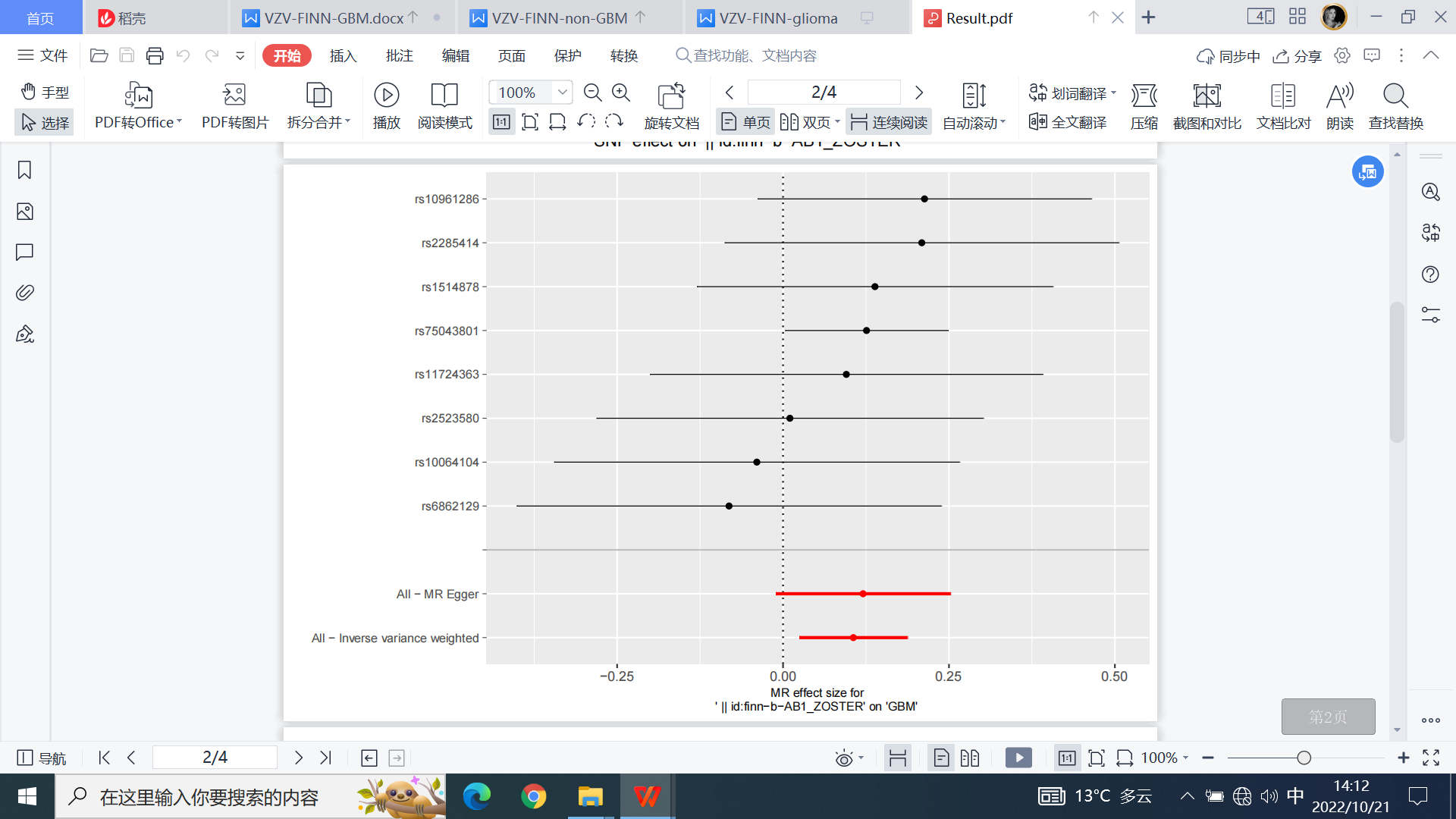


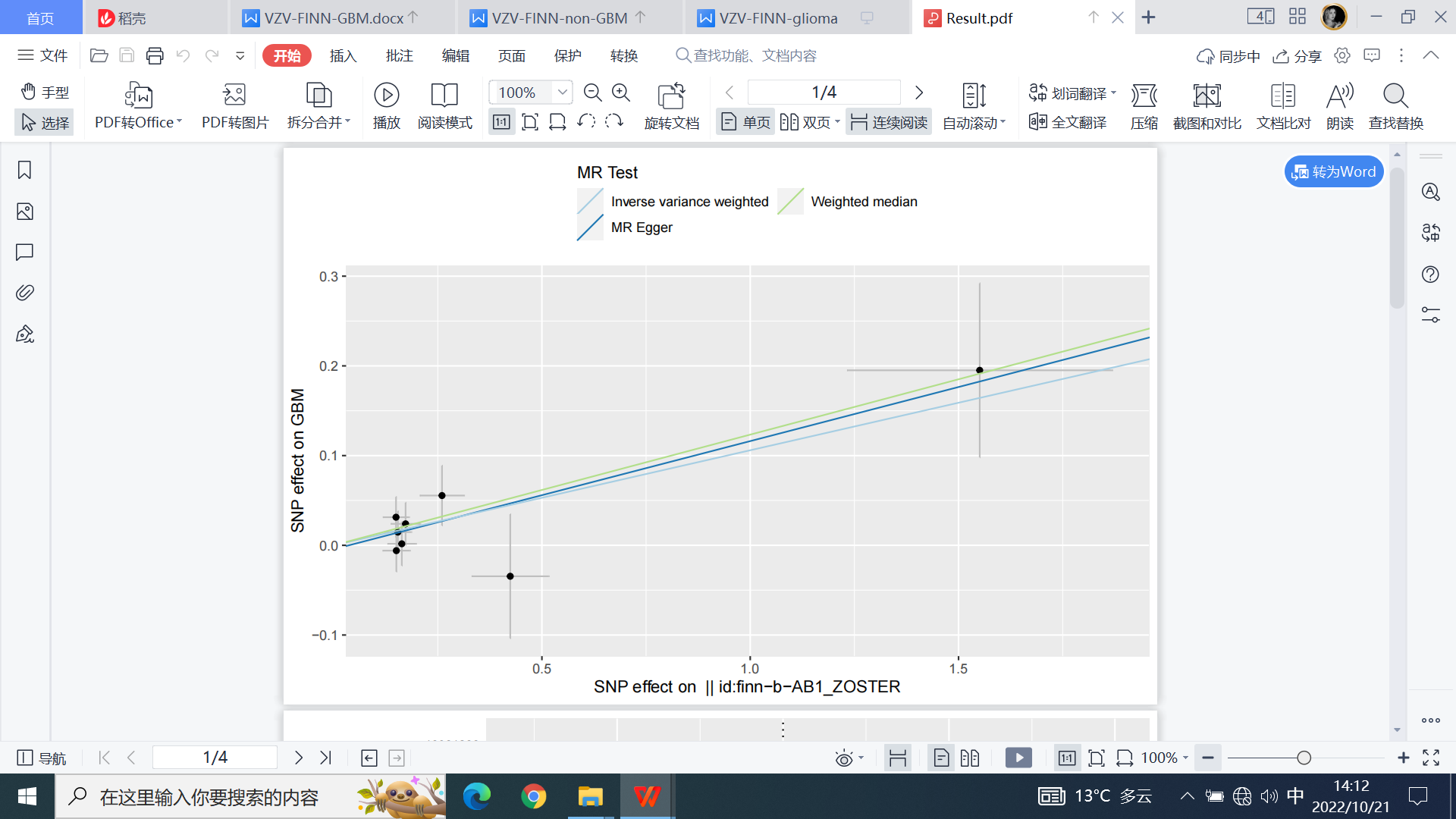


**Figure S20.** The leave-one-out plot, forest plot, and scatter plot for the association of mumps virus infection and GBM in primary analysis. Data from FINN.


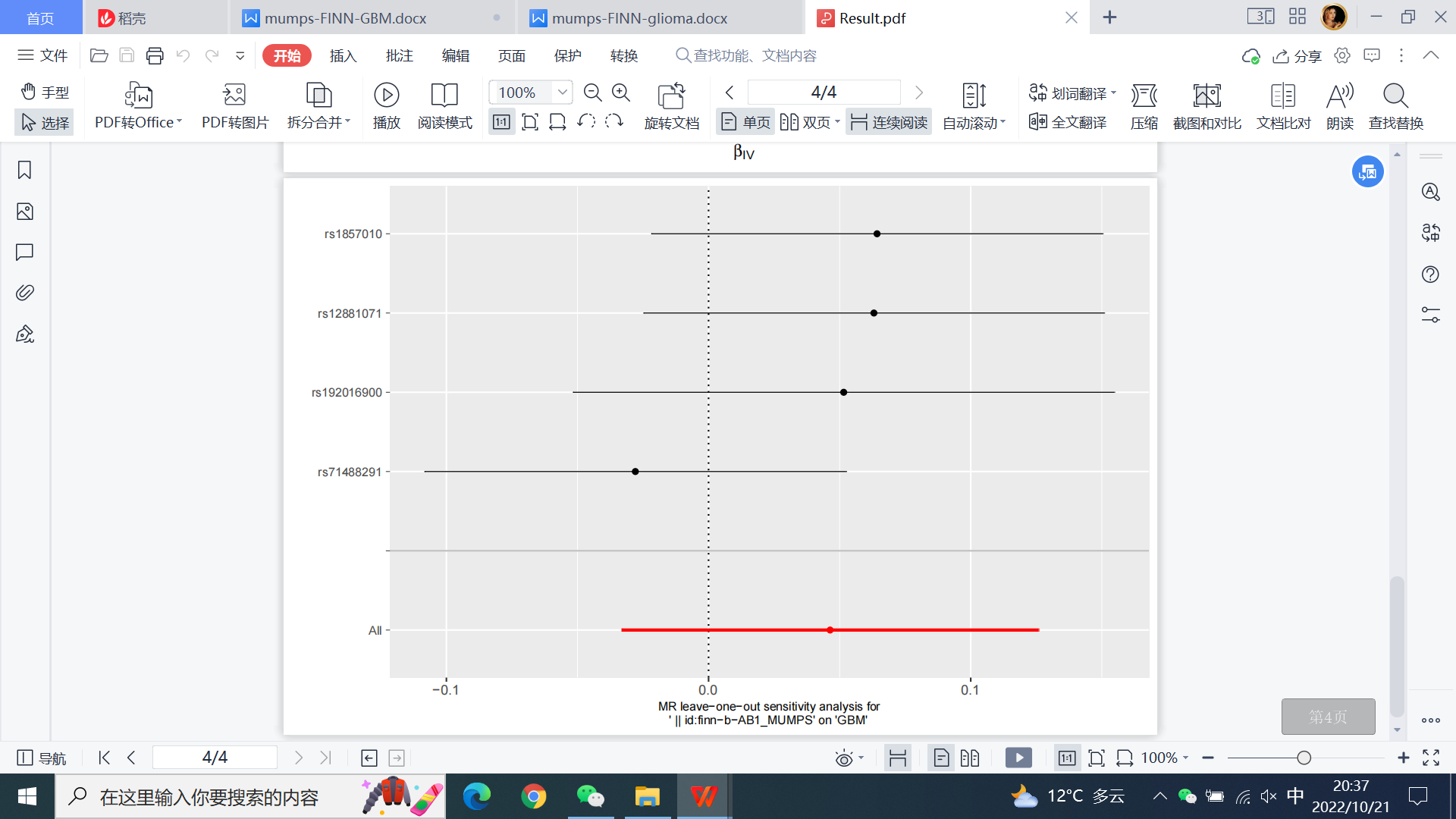


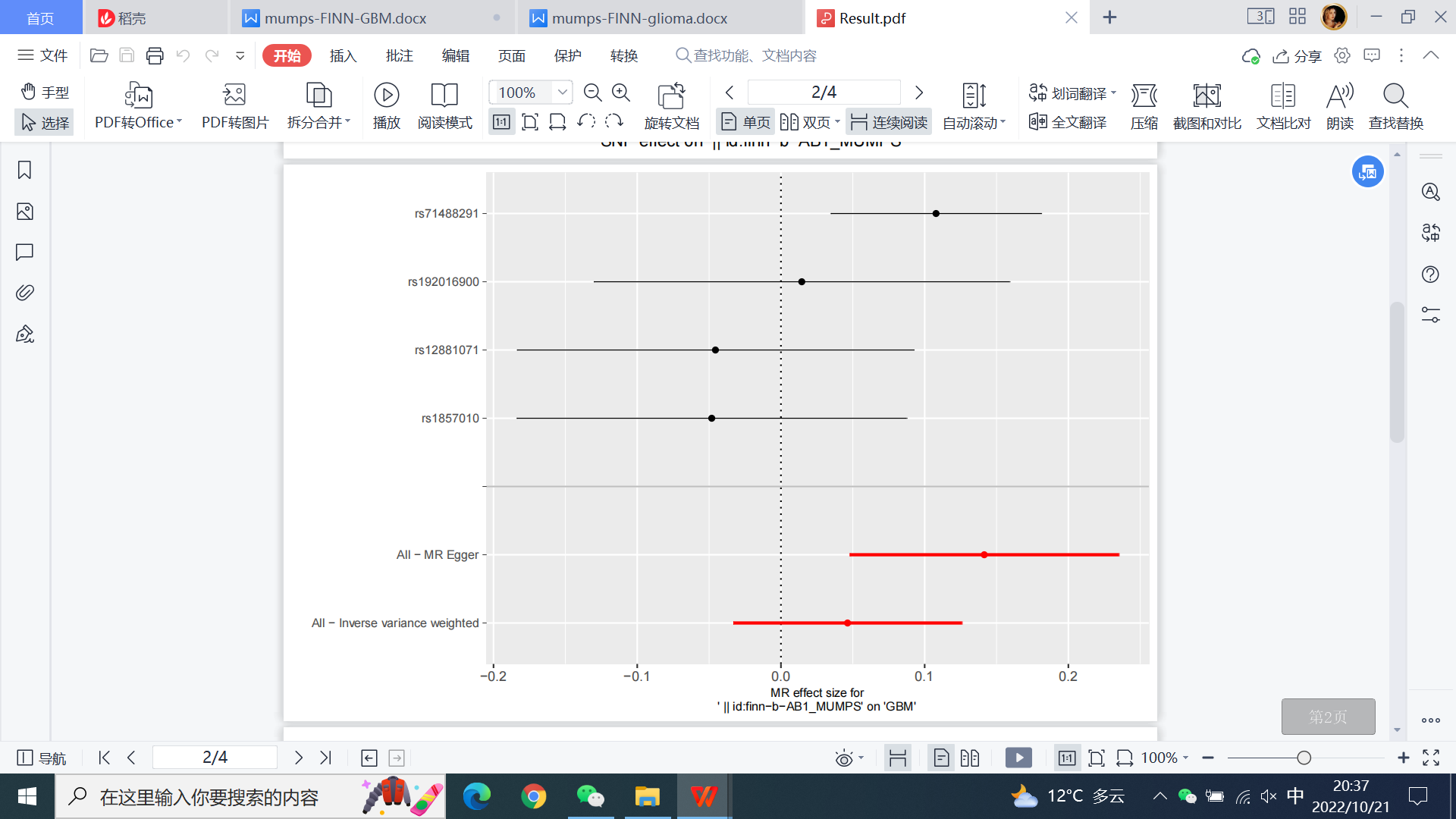


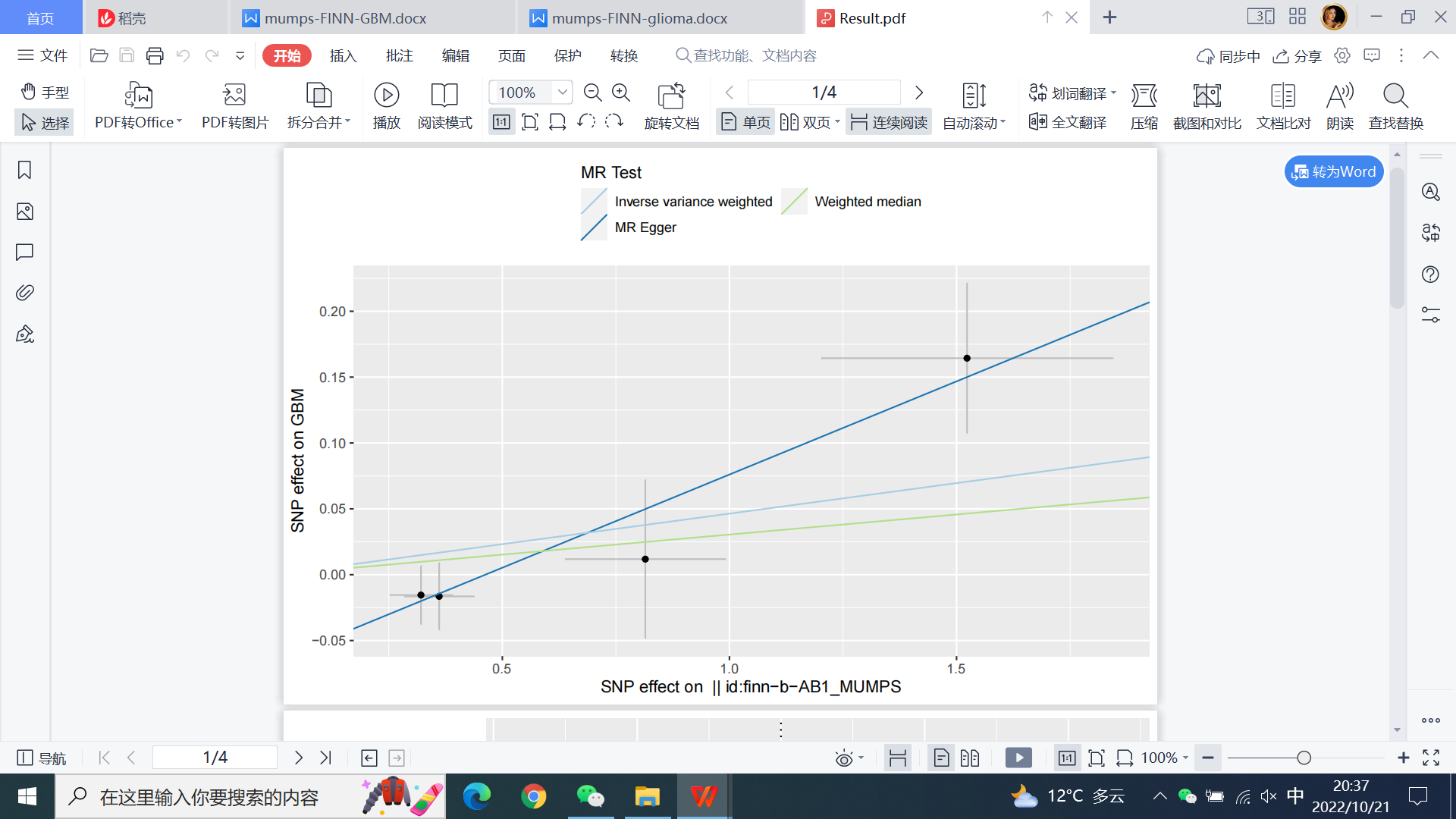


**Figure S21.** The leave-one-out plot, forest plot, and scatter plot for the association of mumps virus infection and GBM in primary analysis. Data from 23andme.


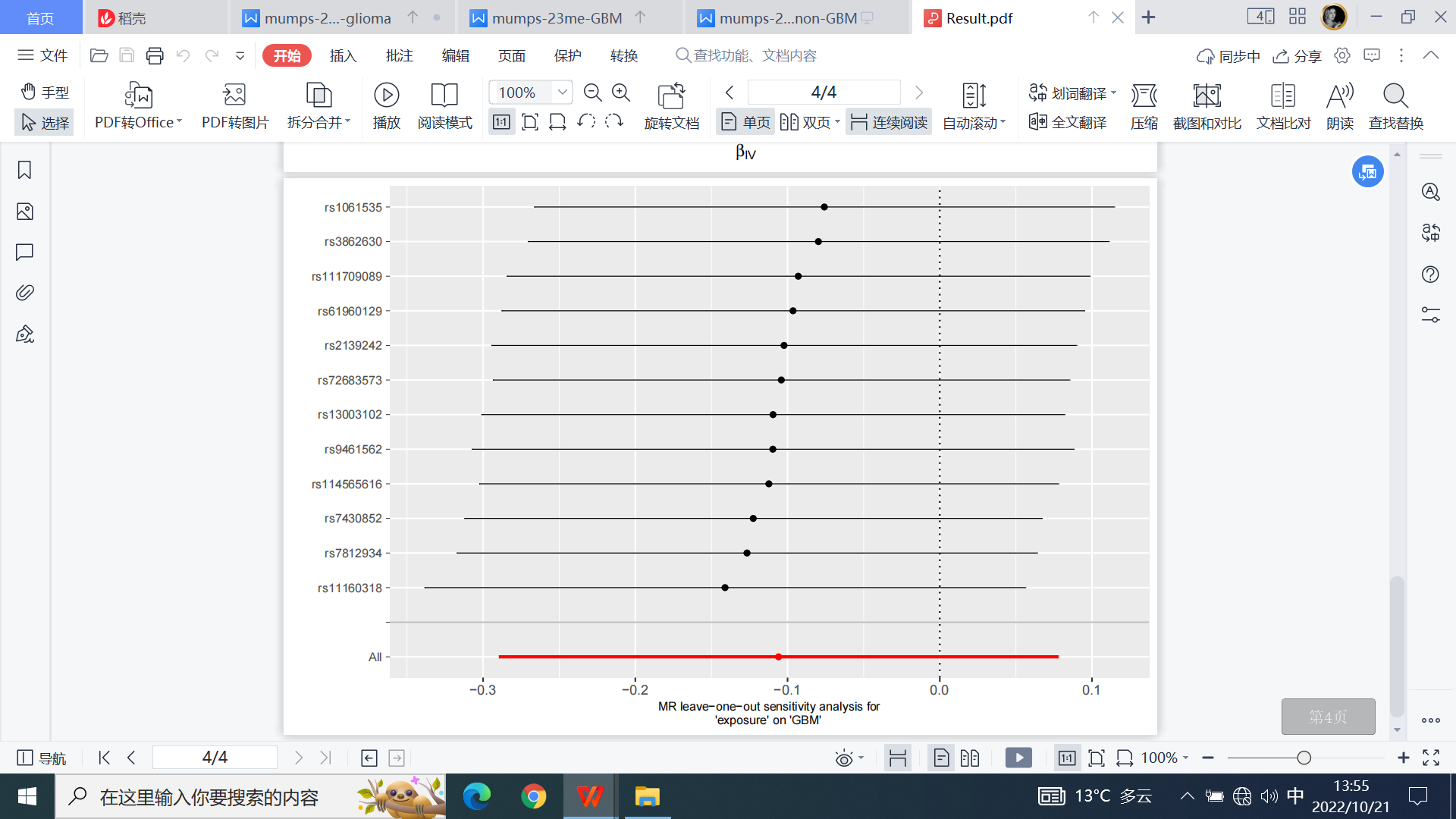


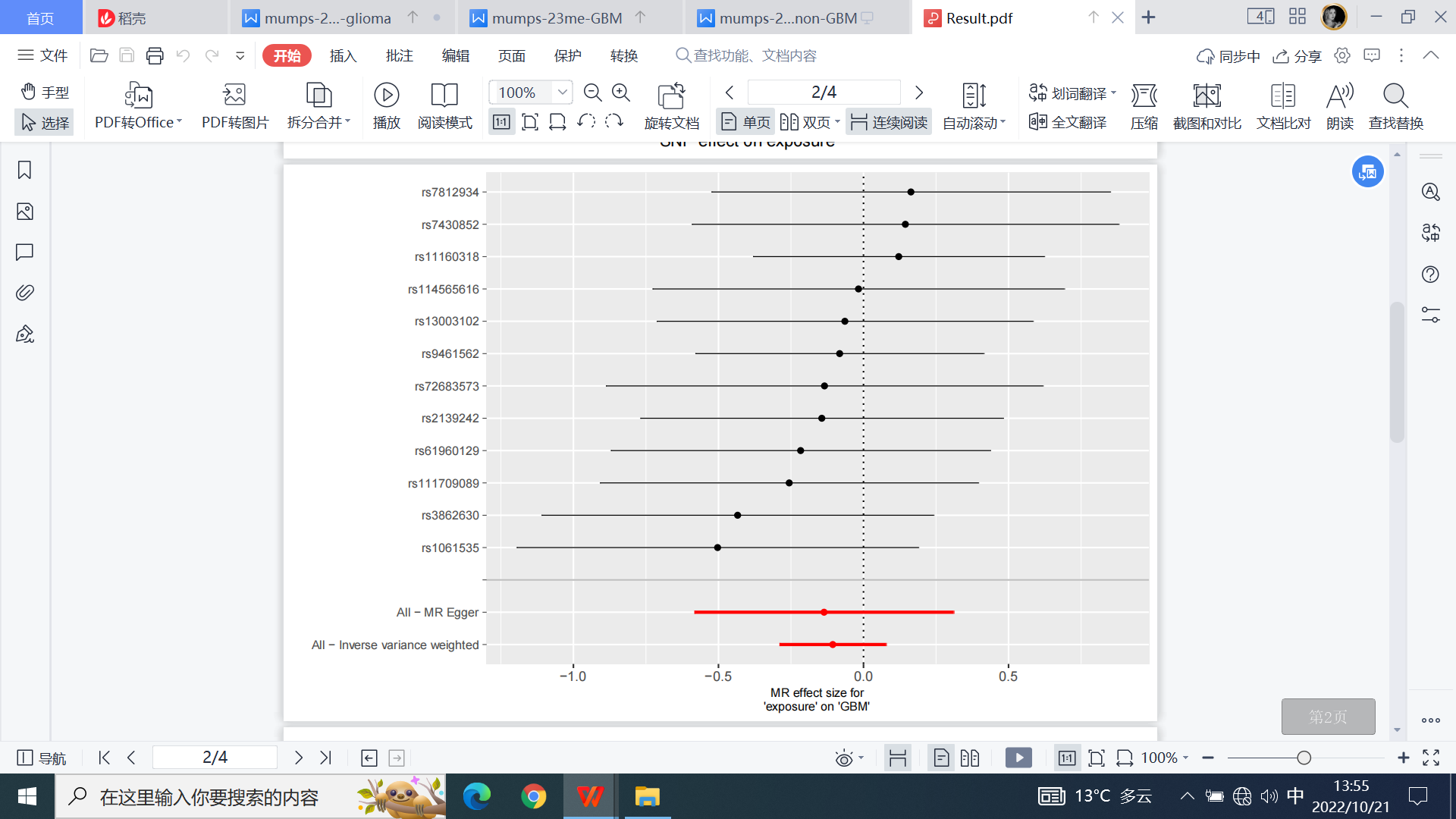


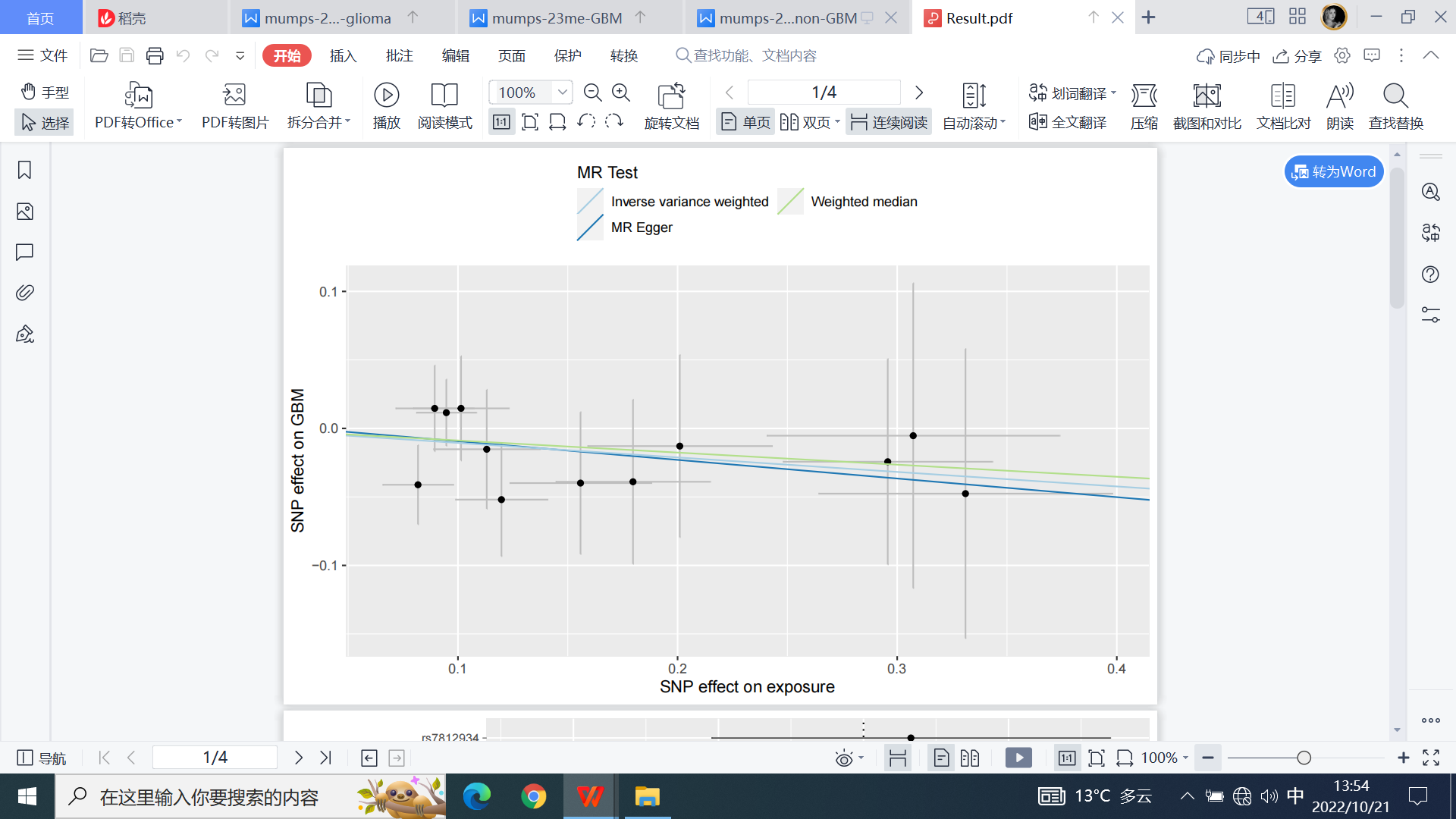


**Figure S22.** The leave-one-out plot, forest plot, and scatter plot for the association of HSV infection and GBM in primary analysis. Data from FINN.


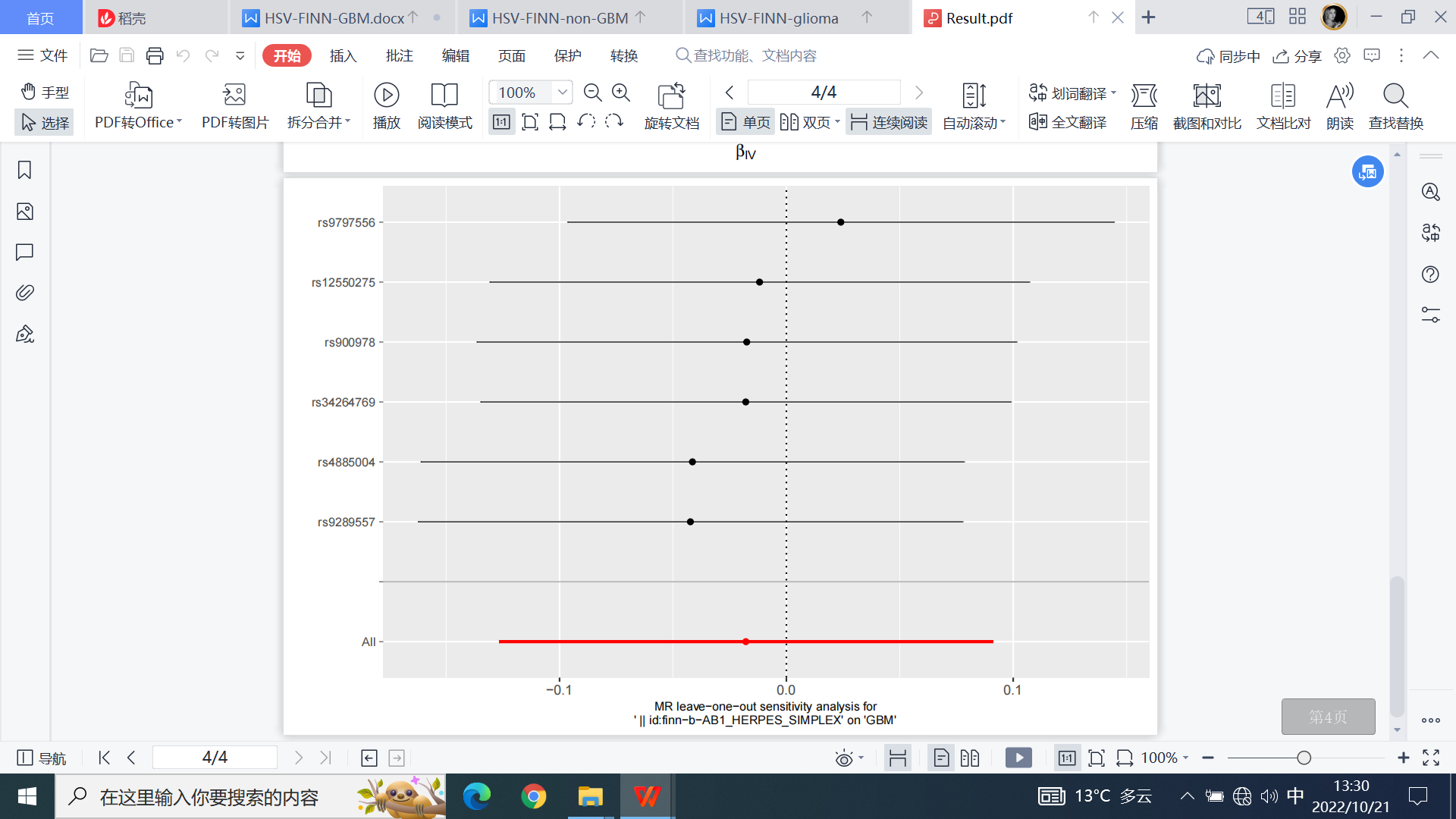


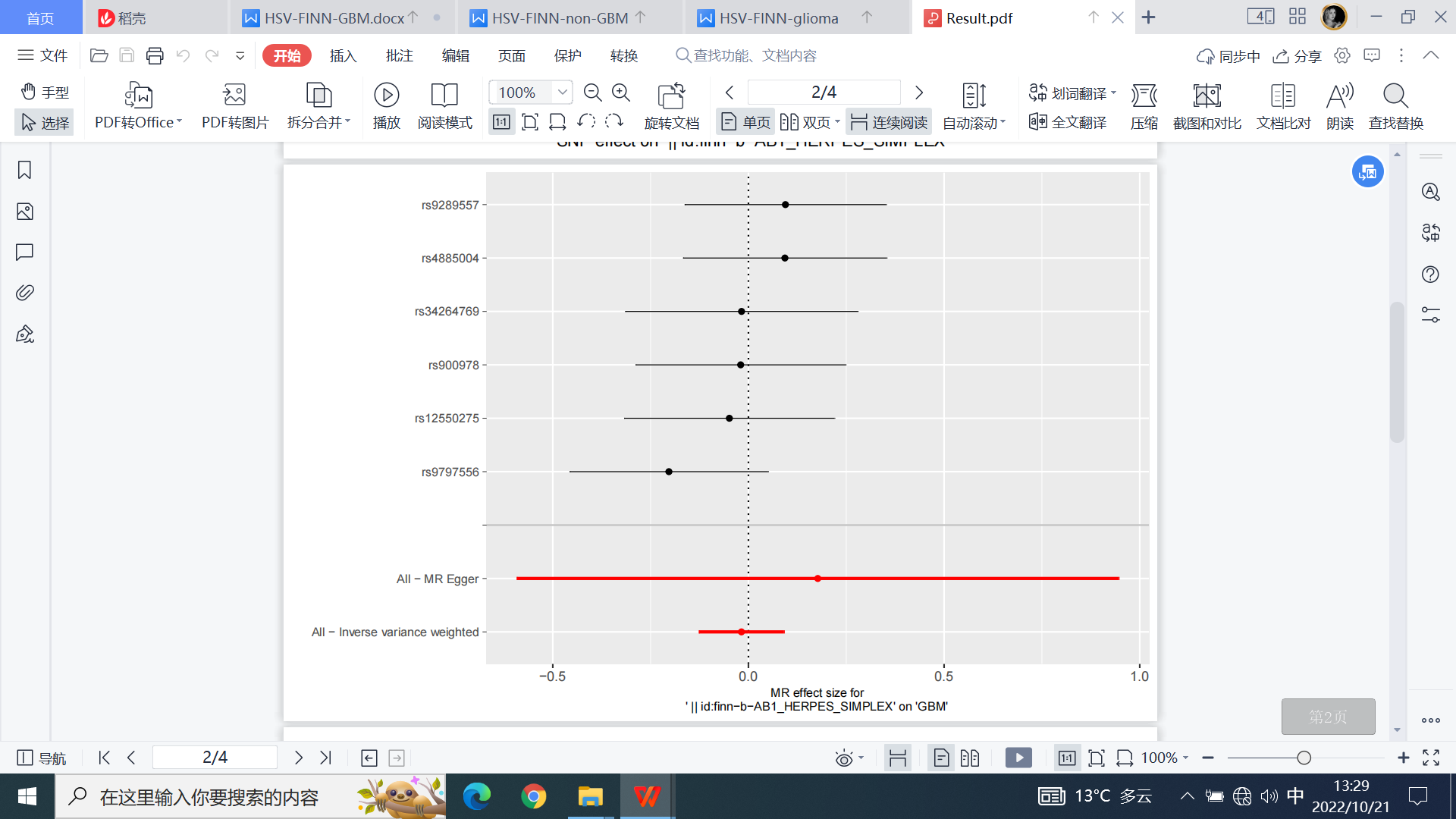

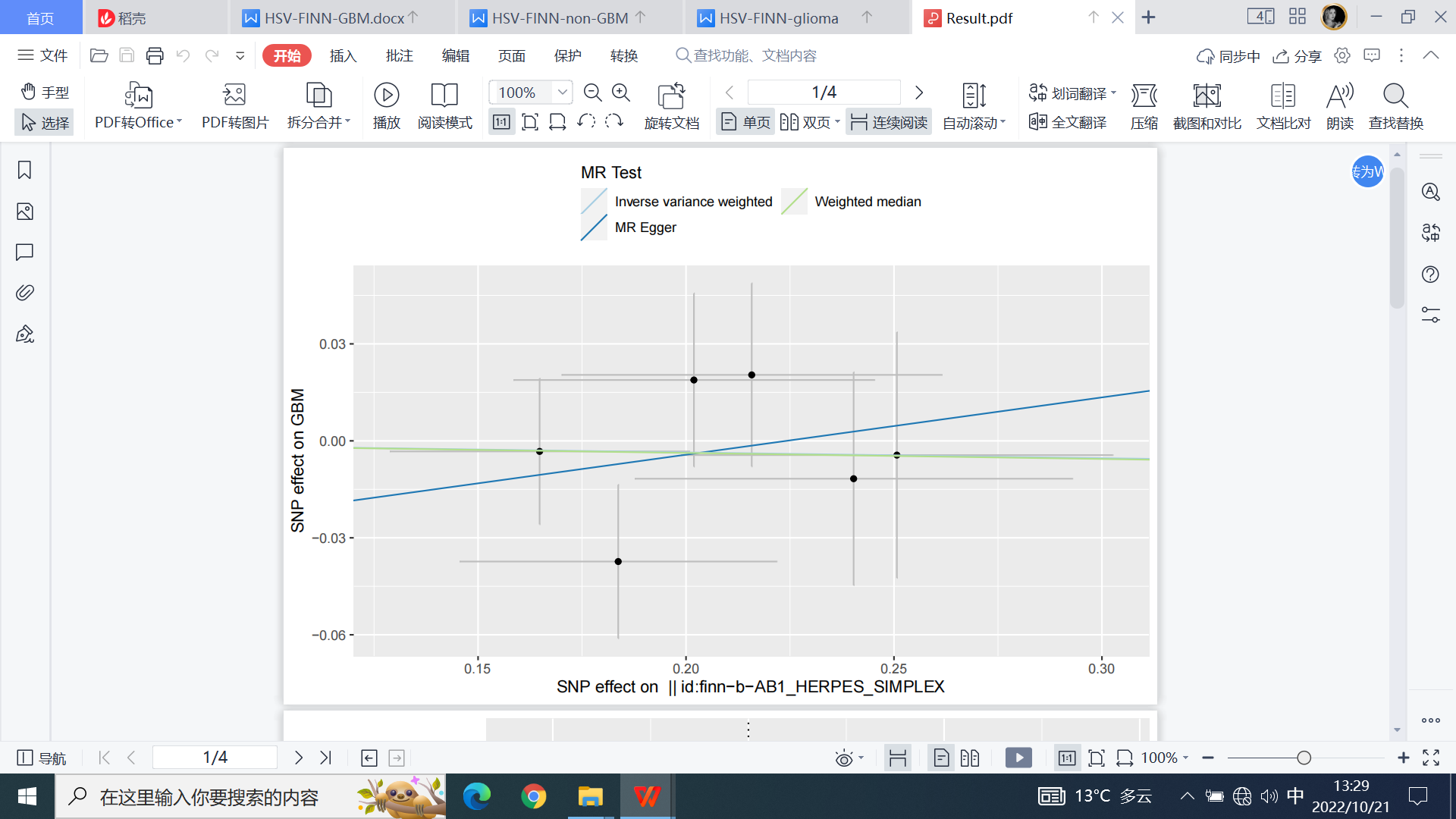


**Figure S23.** The leave-one-out plot, forest plot, and scatter plot for the association of HSV infection and GBM in primary analysis. Data from 23andme.


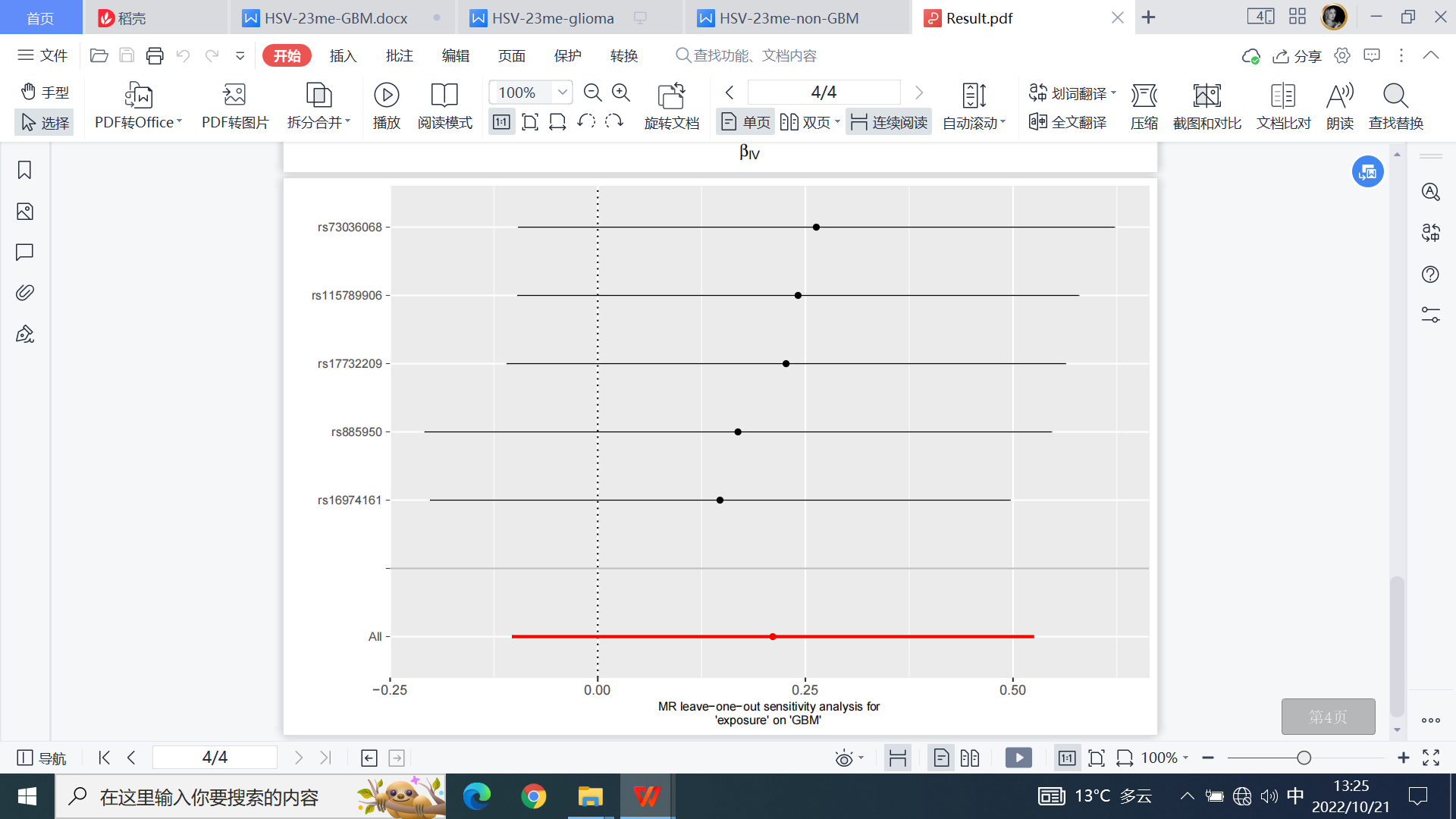


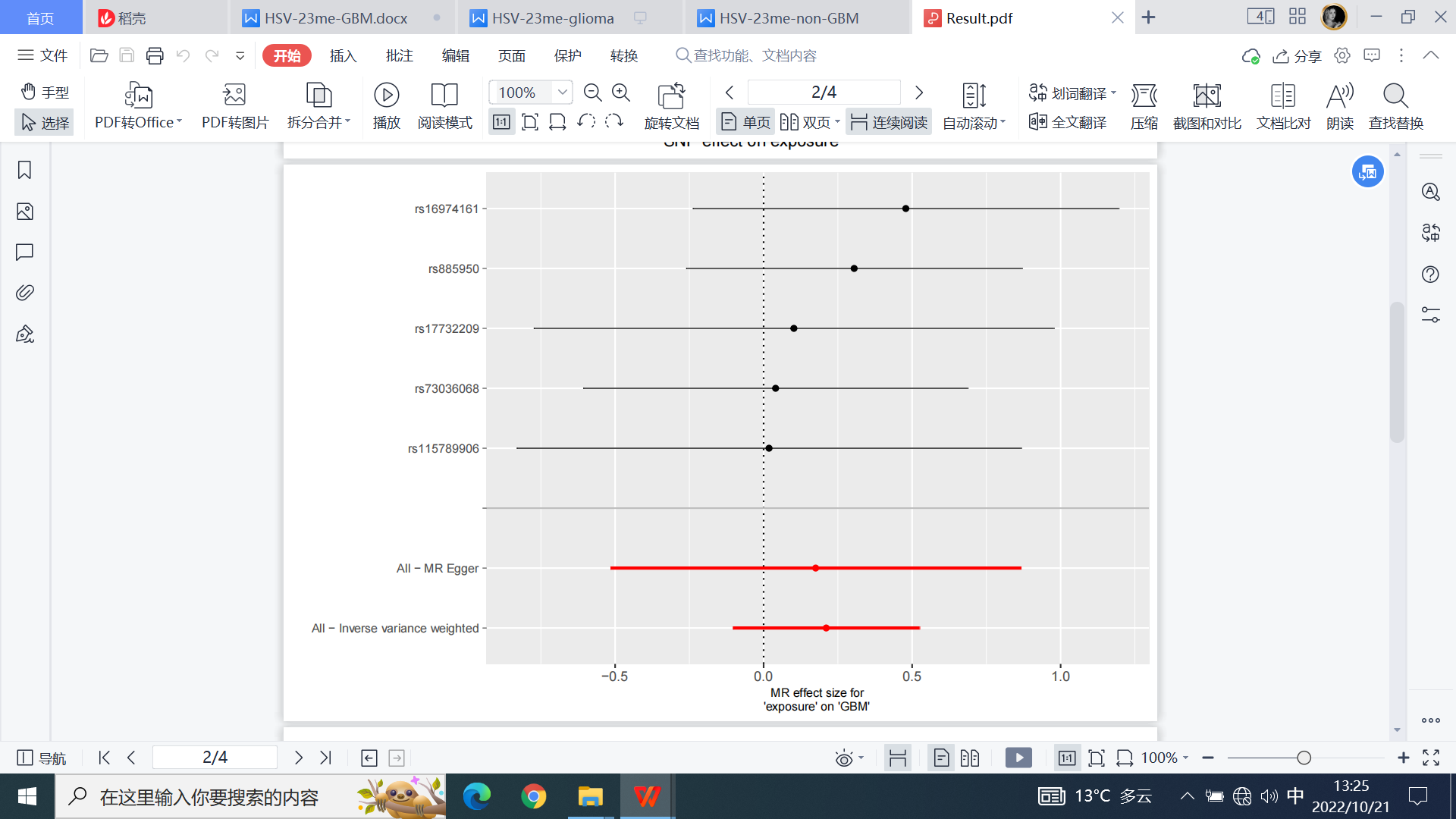


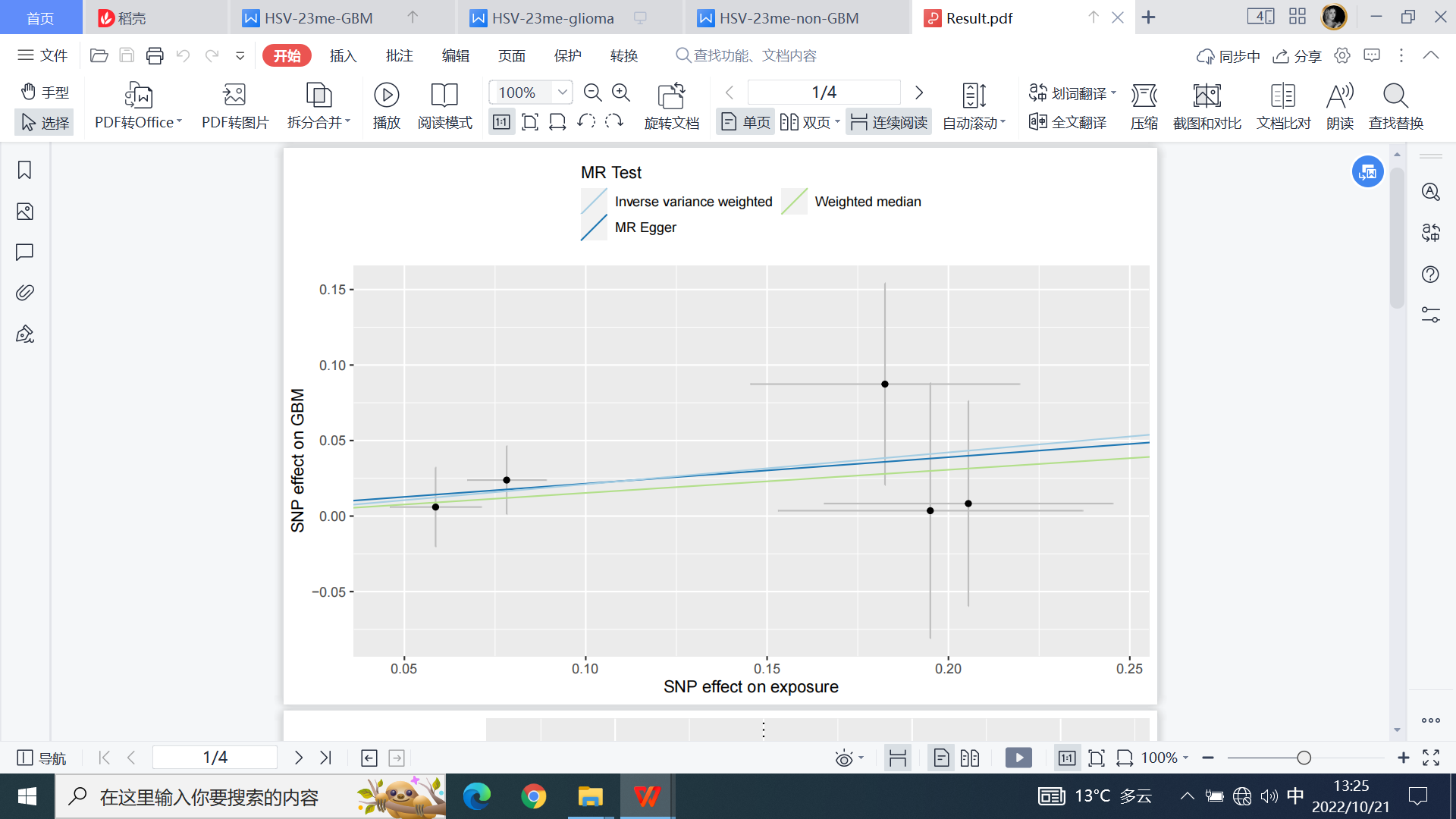


**Figure S24.** The leave-one-out plot, forest plot, and scatter plot for the association of HPV 16E7 and GBM in primary analysis. Data from SUHRE.


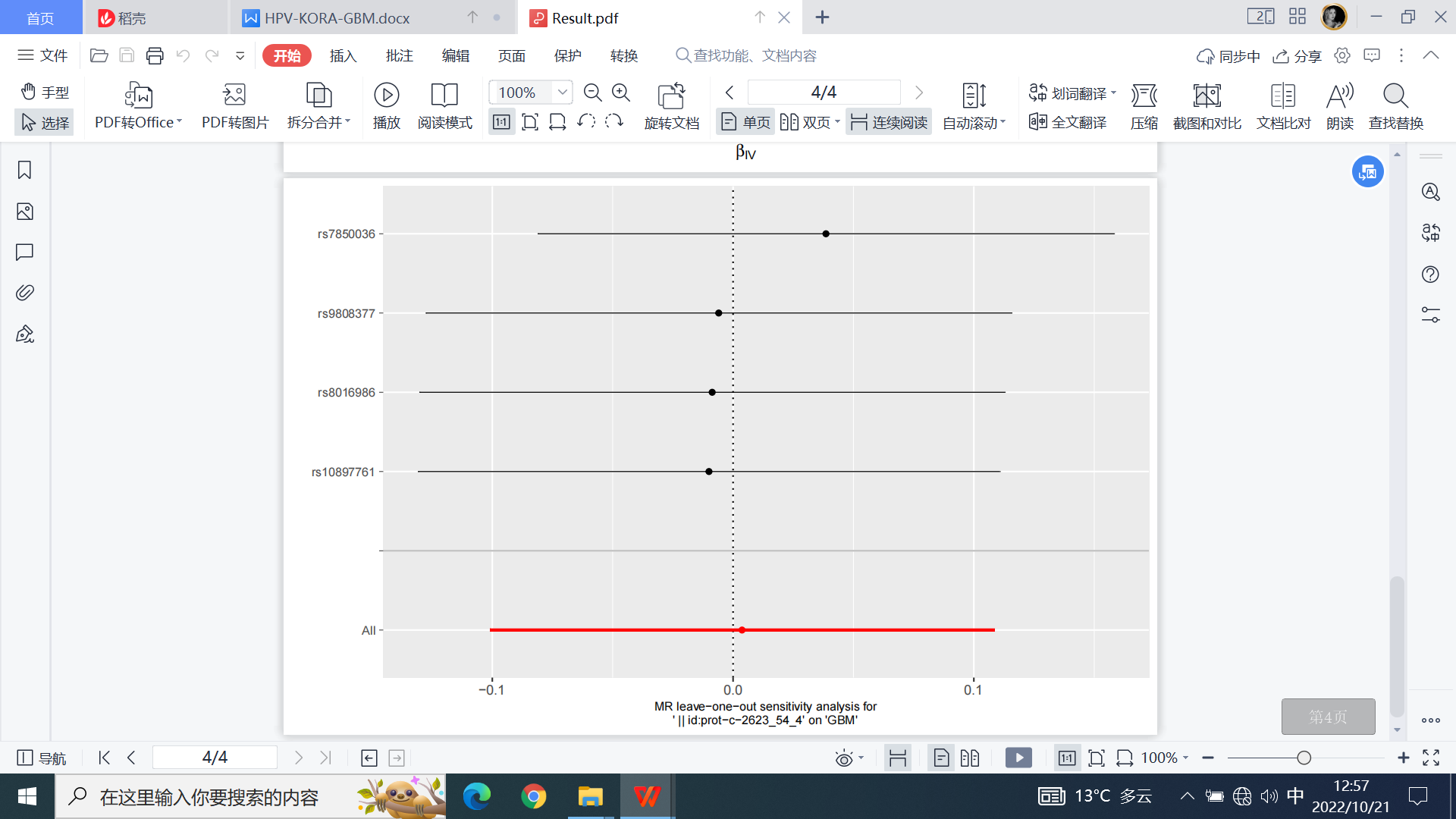


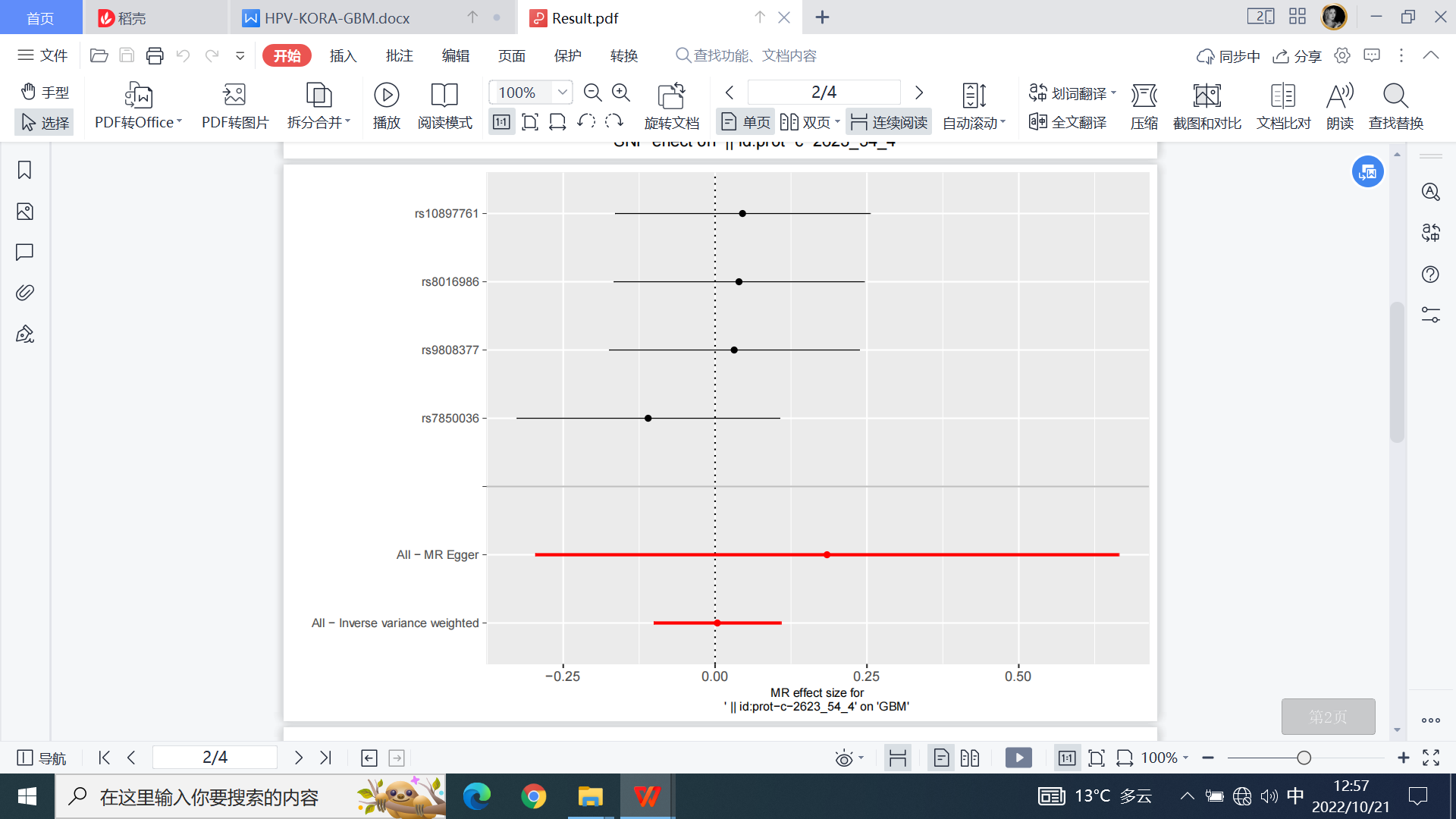


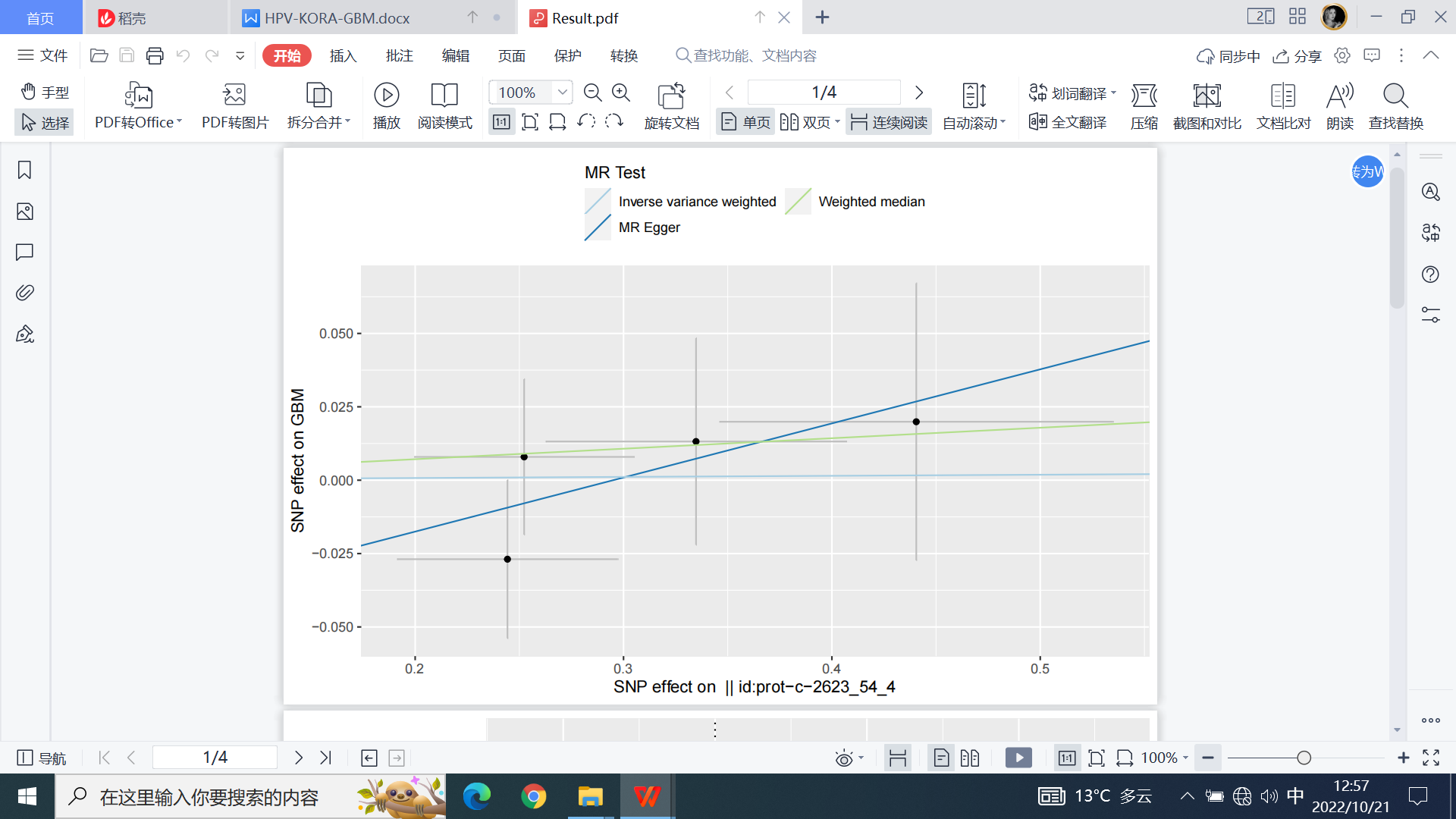


**Figure S25.** The leave-one-out plot, forest plot, and scatter plot for the association of EBV infection and GBM in primary analysis. Data from FINN.


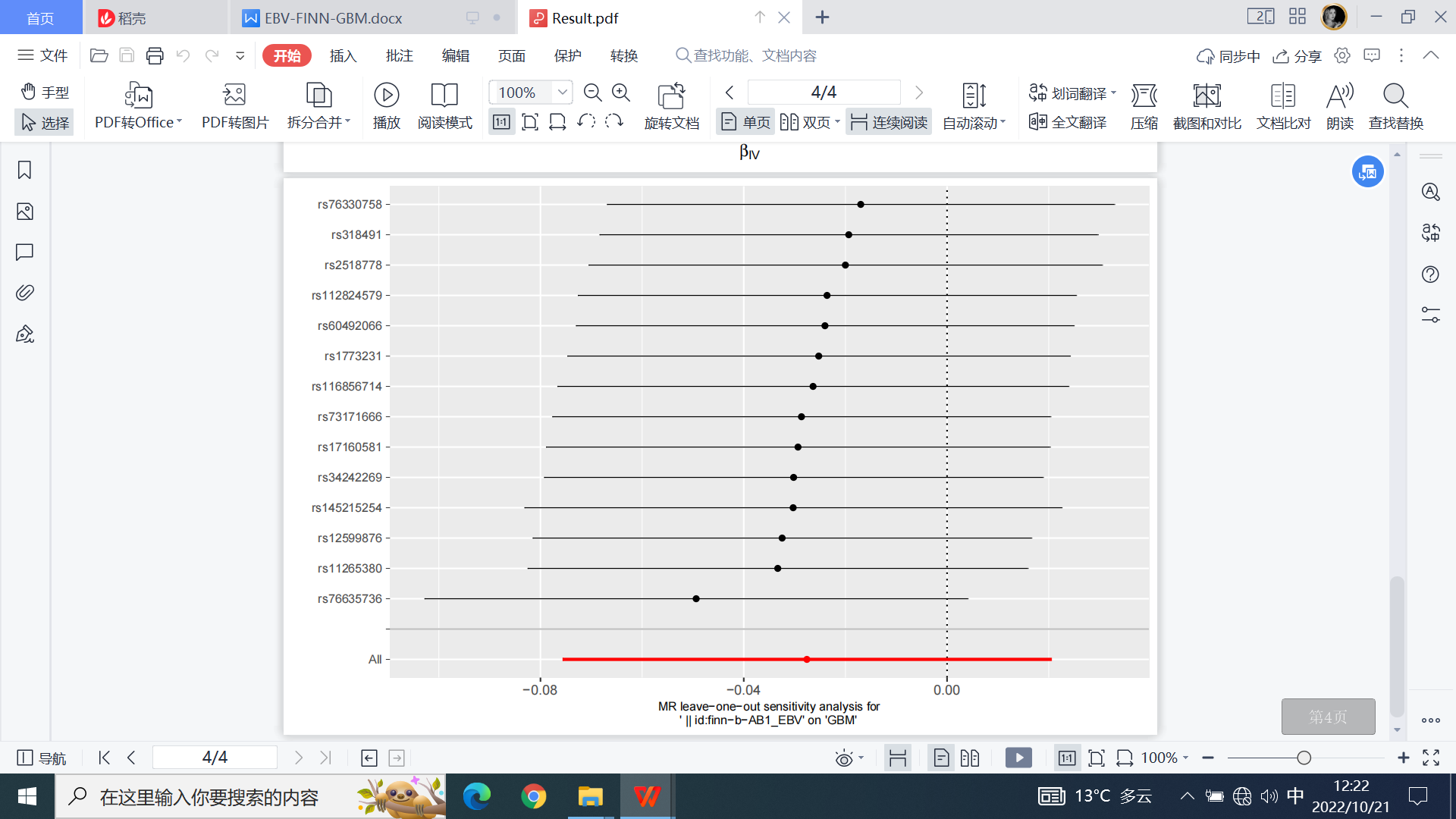


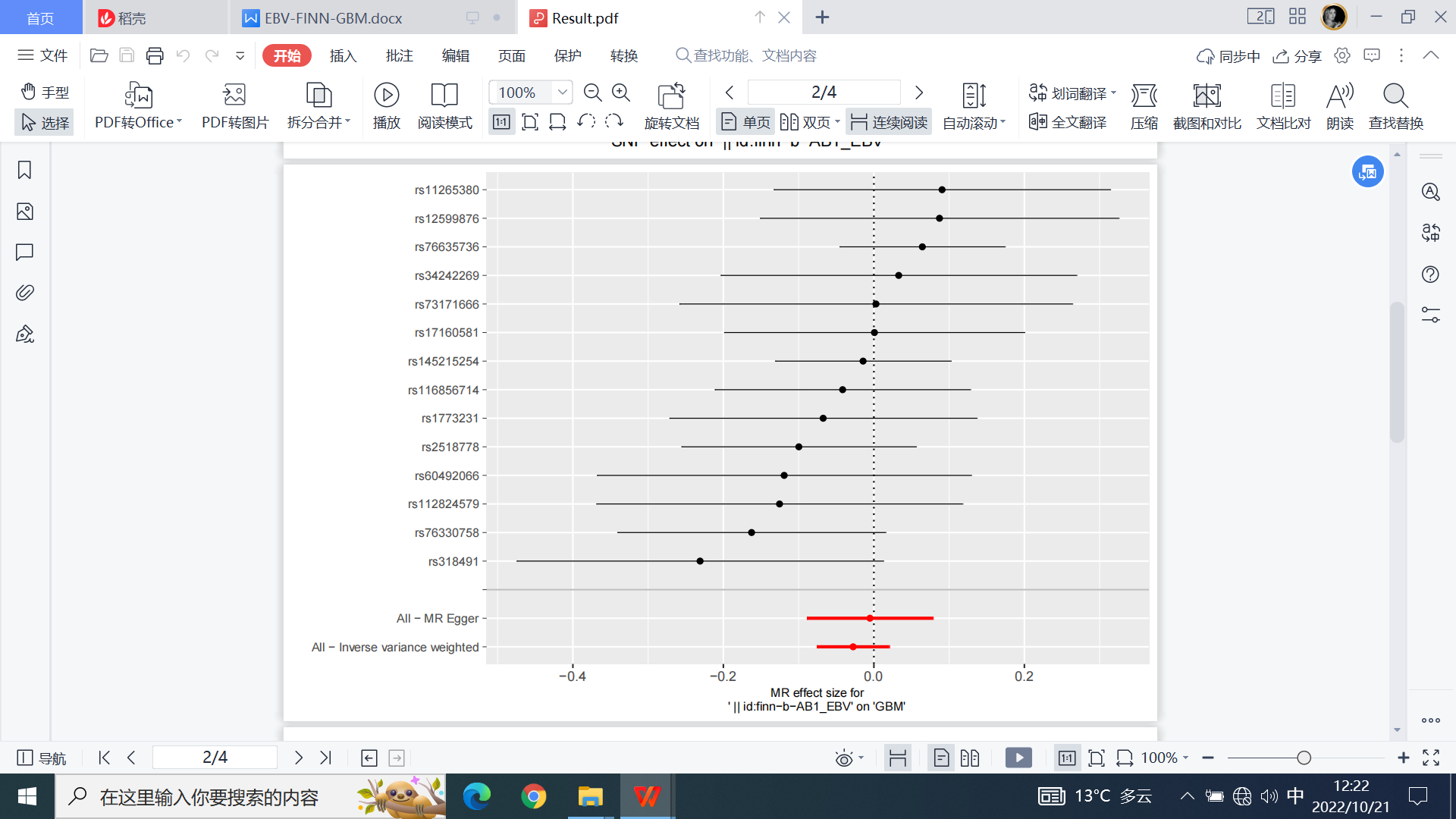


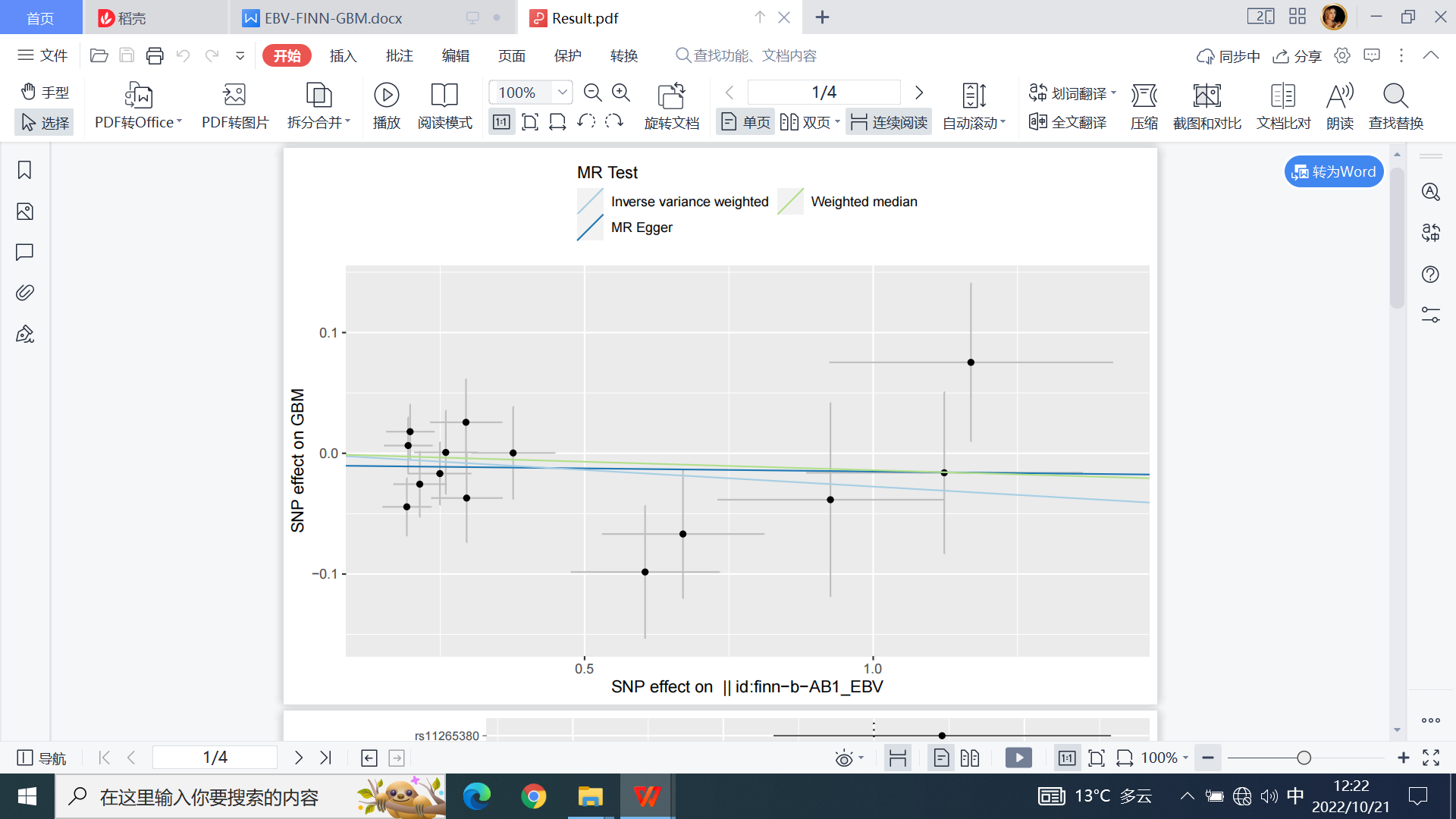


**Figure S26.** The leave-one-out plot, forest plot, and scatter plot for the association of EBV infection and GBM in primary analysis. Data from 23andme.


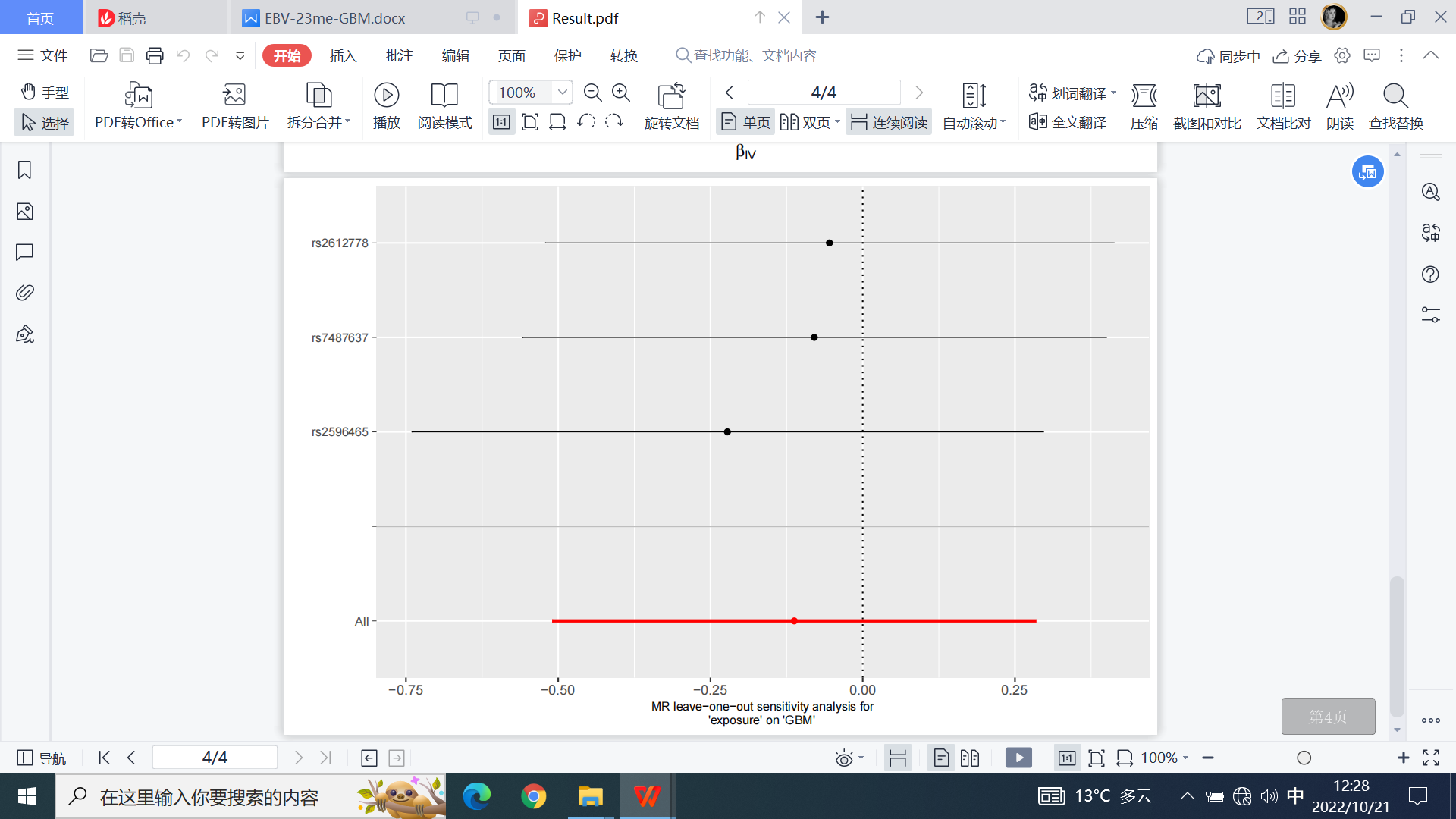


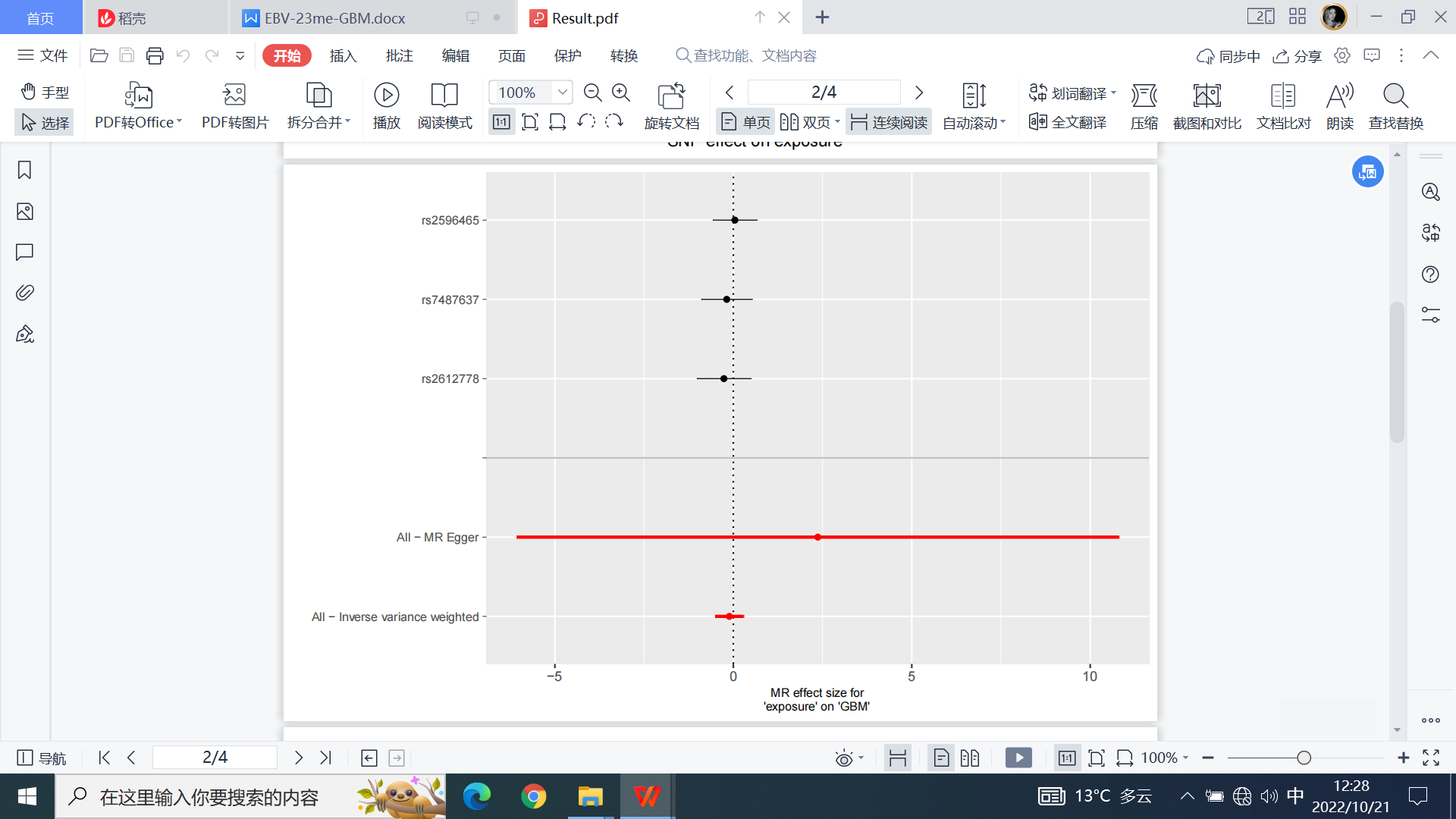


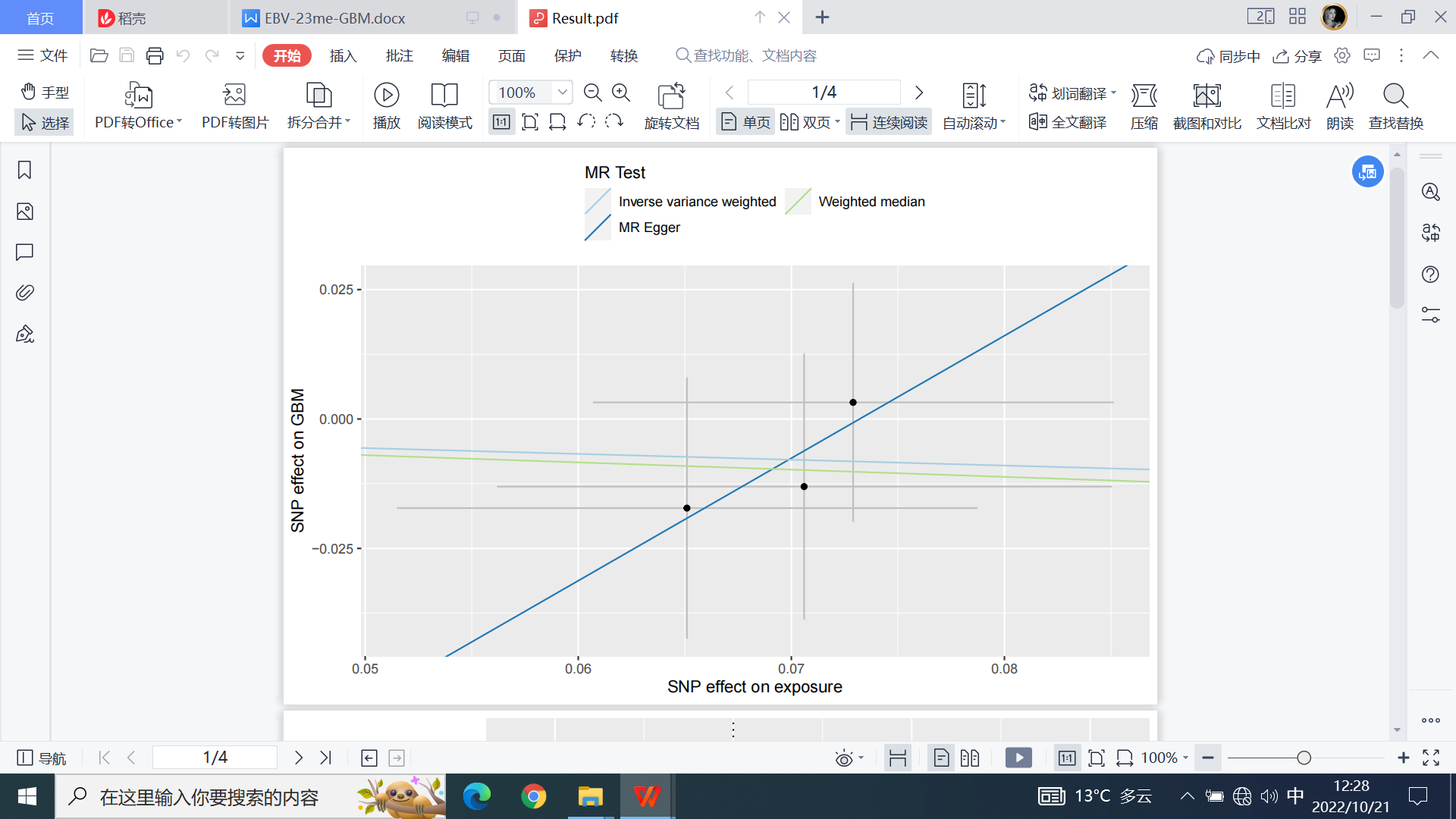


**Figure S27.** The leave-one-out plot, forest plot, and scatter plot for the association of COVID-19 infection and GBM in primary analysis. Data from COVID-19 HGI.


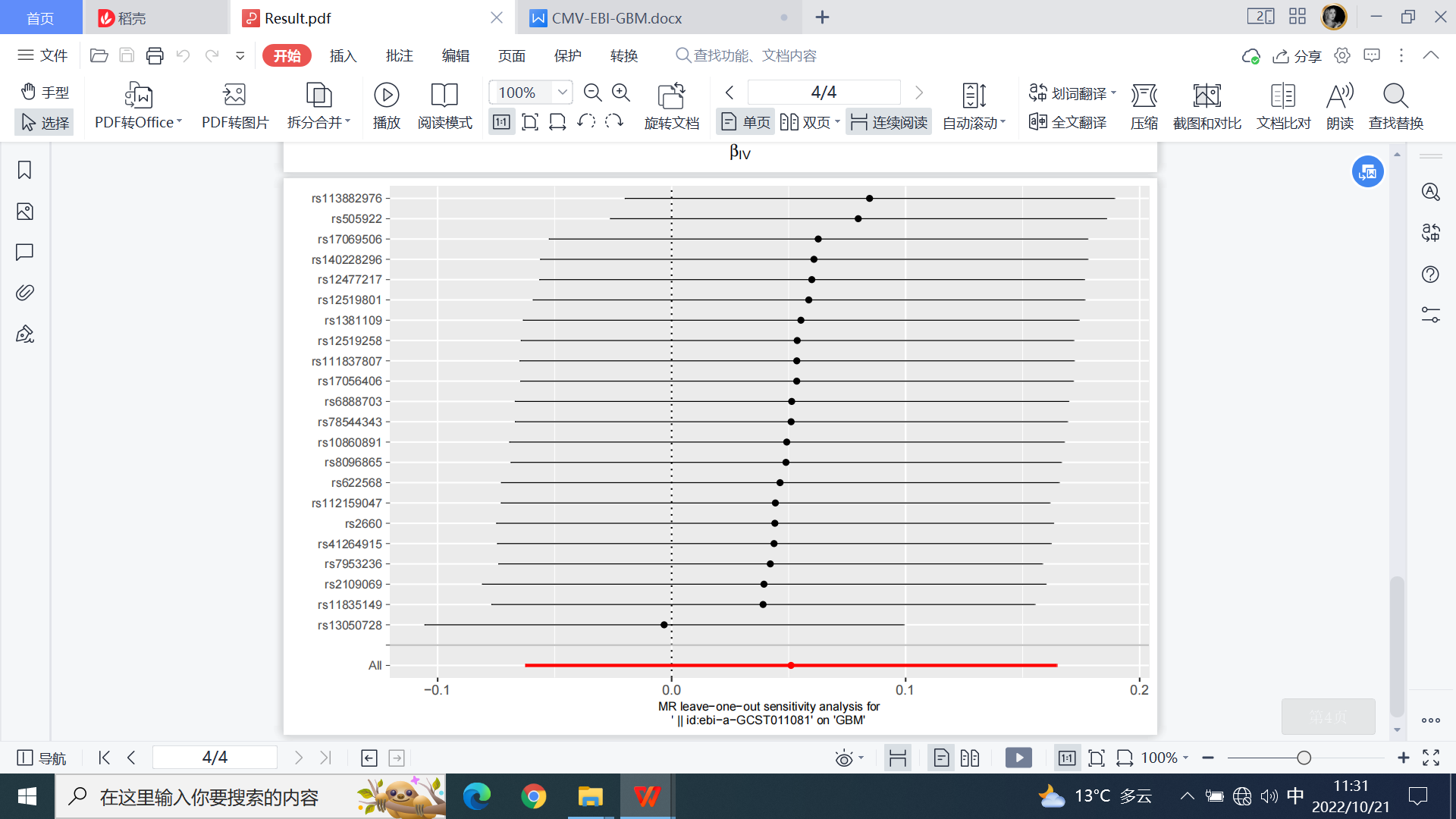


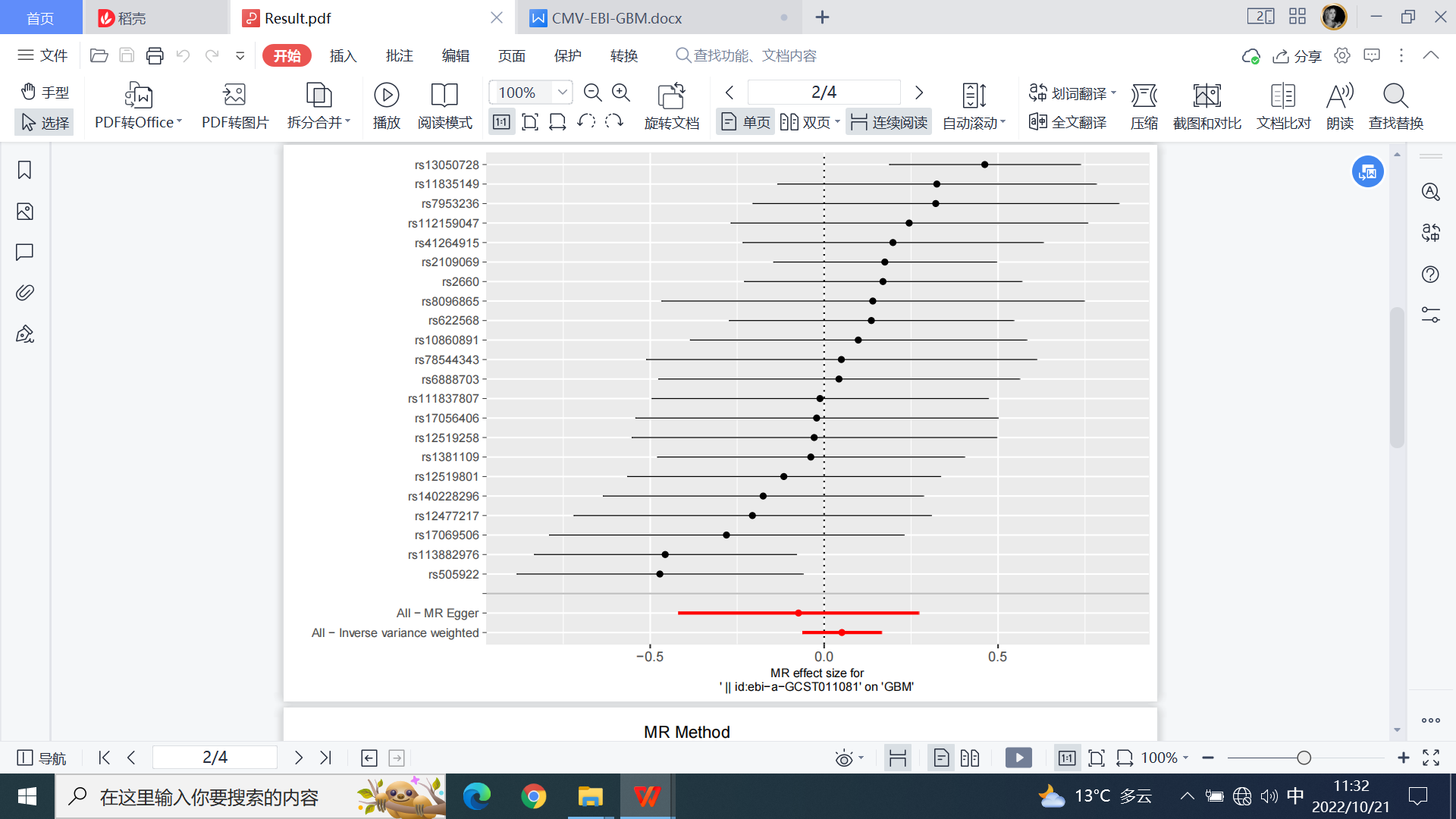


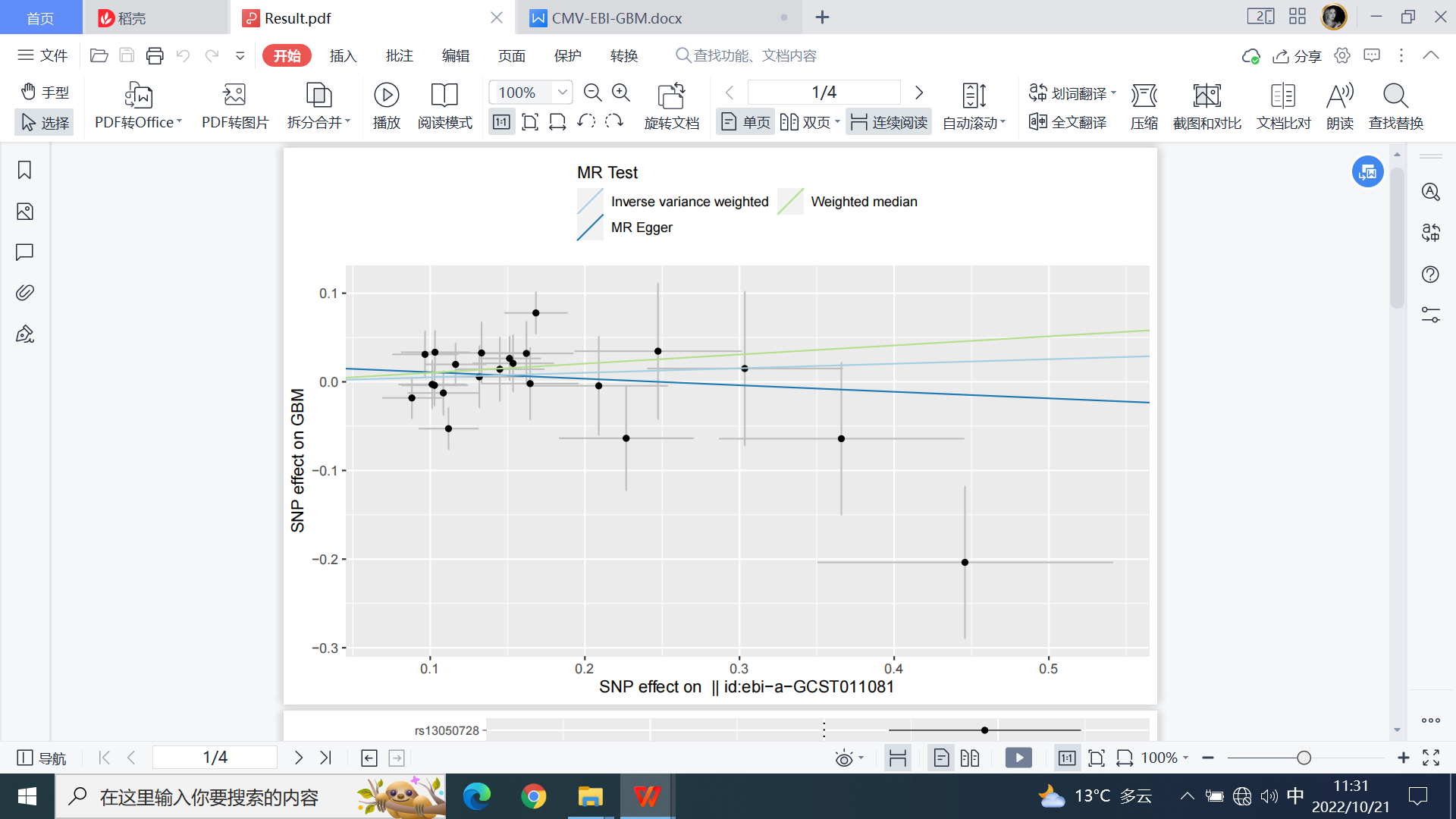


**Figure S28.** The leave-one-out plot, forest plot, and scatter plot for the association of HCMV infection and GBM in primary analysis. Data from FINN.


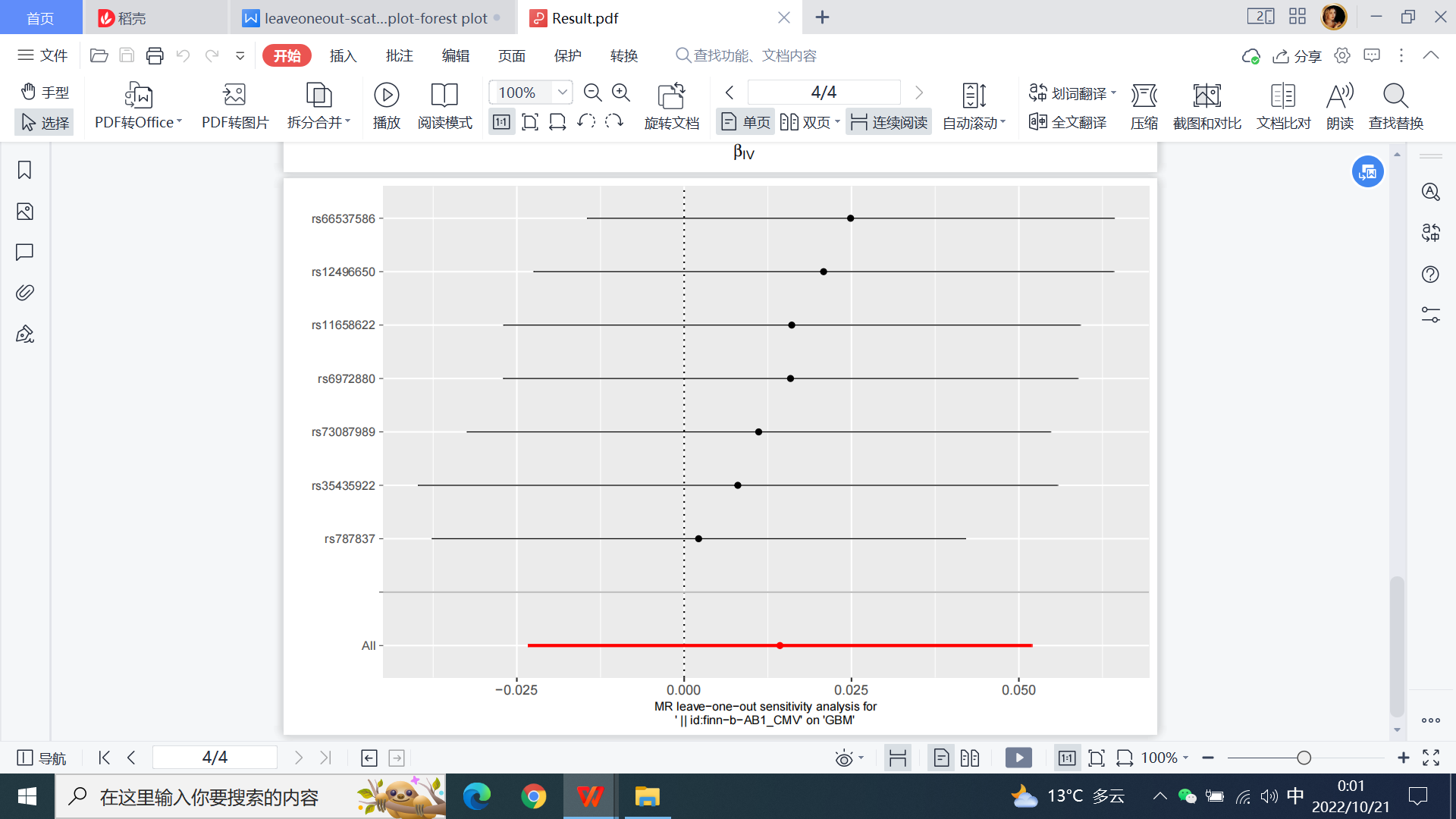


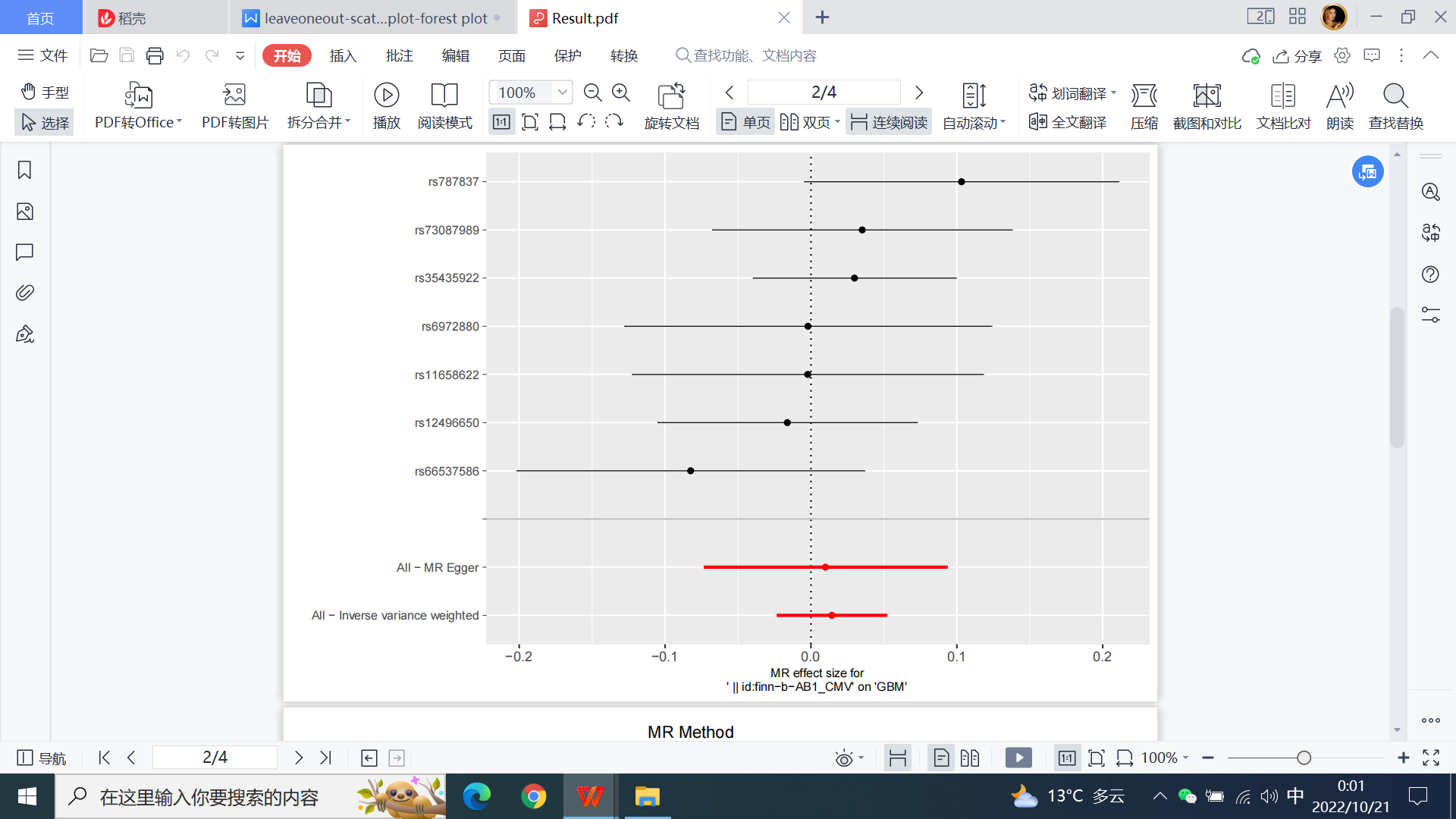


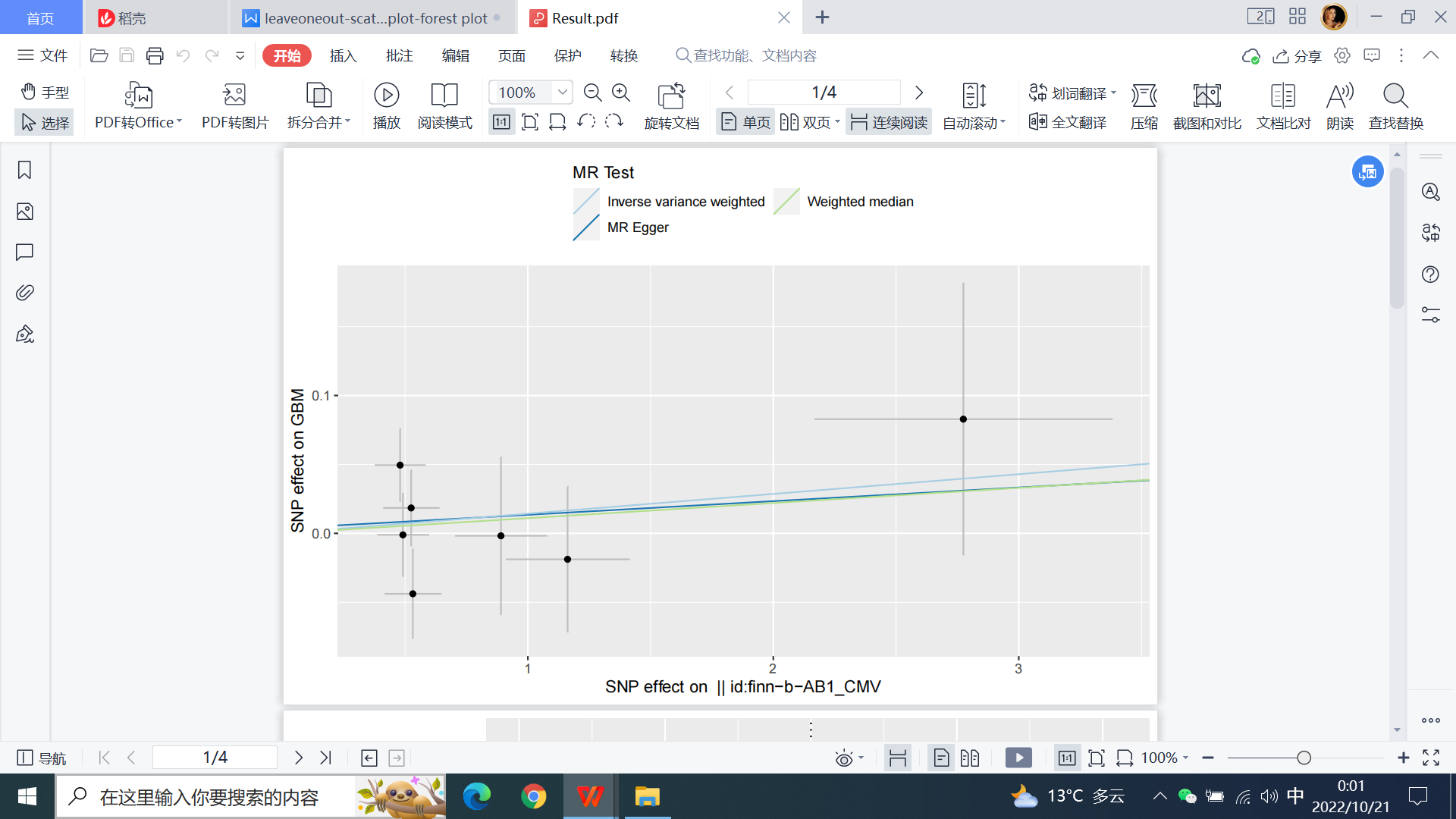


**Figure S29.** The leave-one-out plot, forest plot, and scatter plot for the association of Hepatitis infection and GBM in primary analysis. Data from FINN.


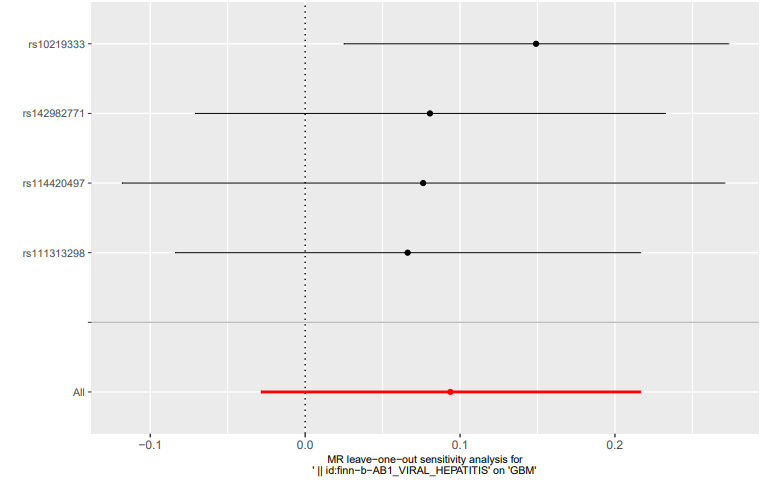


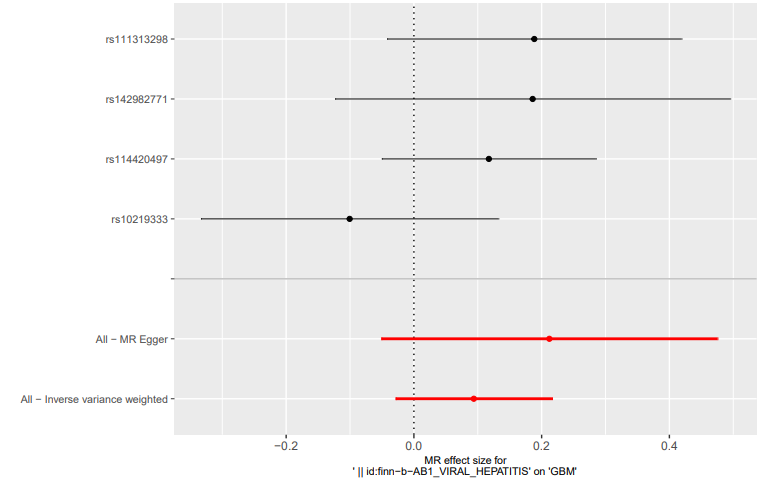


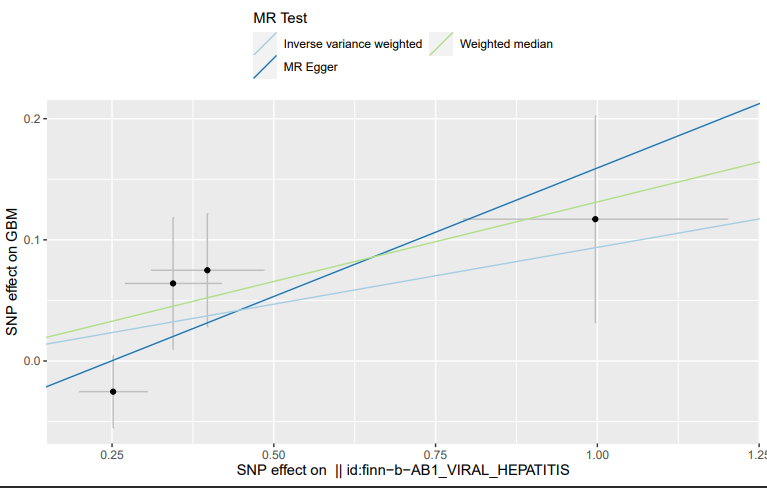


**Figure S30.** The leave-one-out plot, forest plot, and scatter plot for the association of Hepatitis infection and GBM in primary analysis. Data from 23andme.


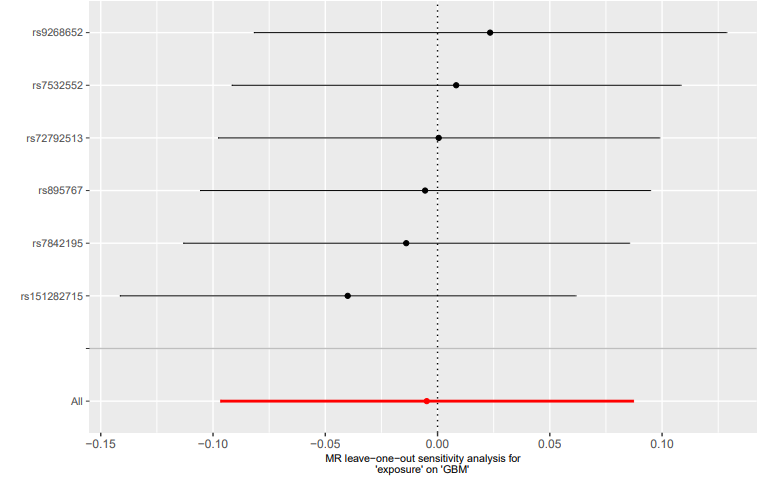


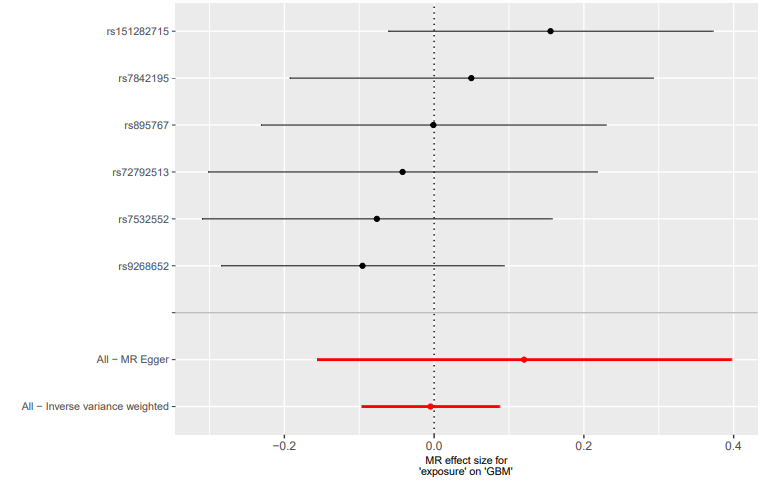


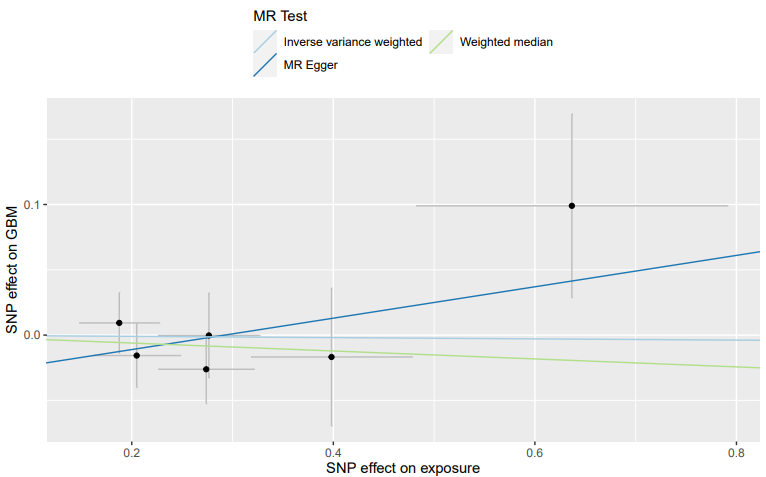


**Figure S31.** The leave-one-out plot, forest plot, and scatter plot for the association of HIV infection and GBM in primary analysis. Data from FINN.


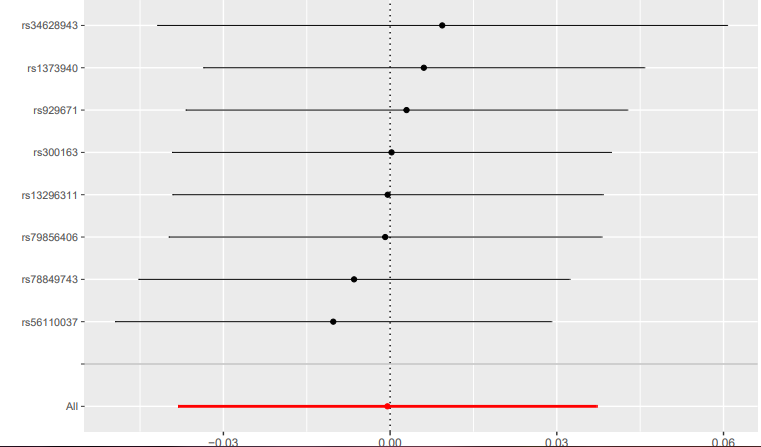


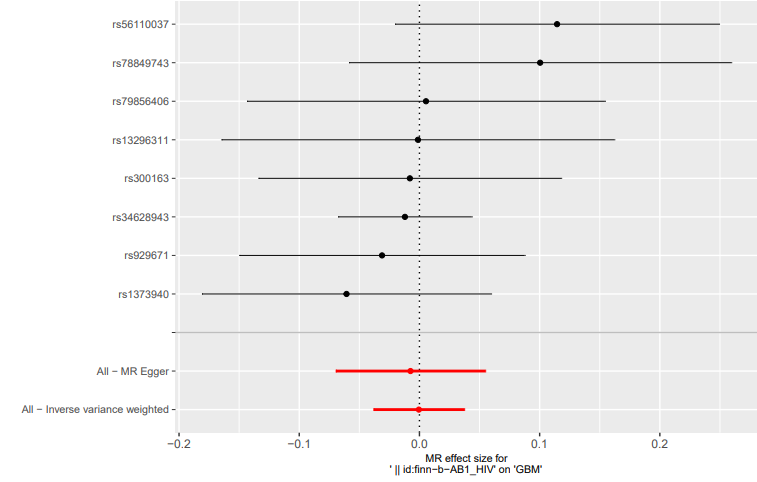


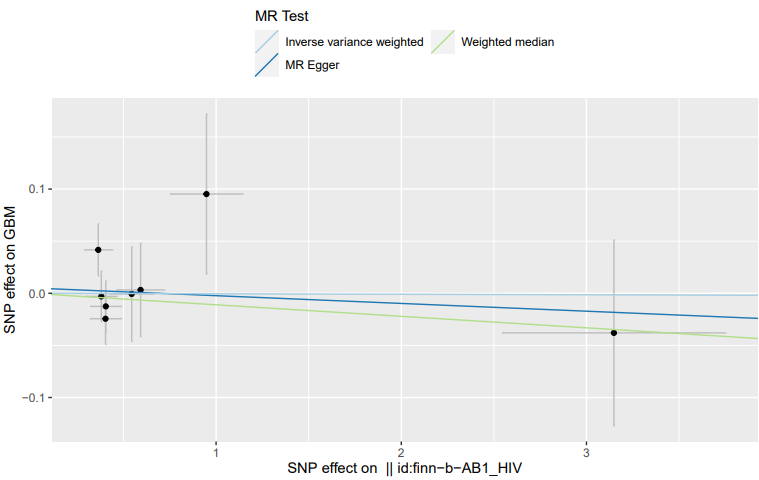


**Figure S32.** The leave-one-out plot, forest plot, and scatter plot for the association of measles infection and GBM in primary analysis. Data from FINN.


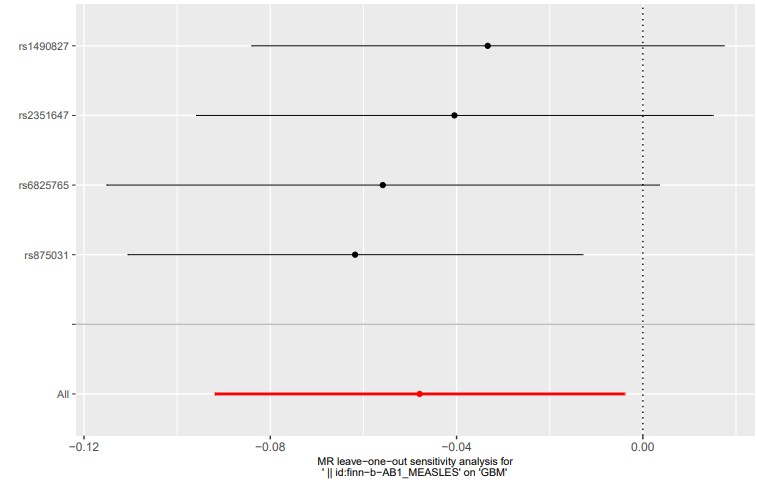


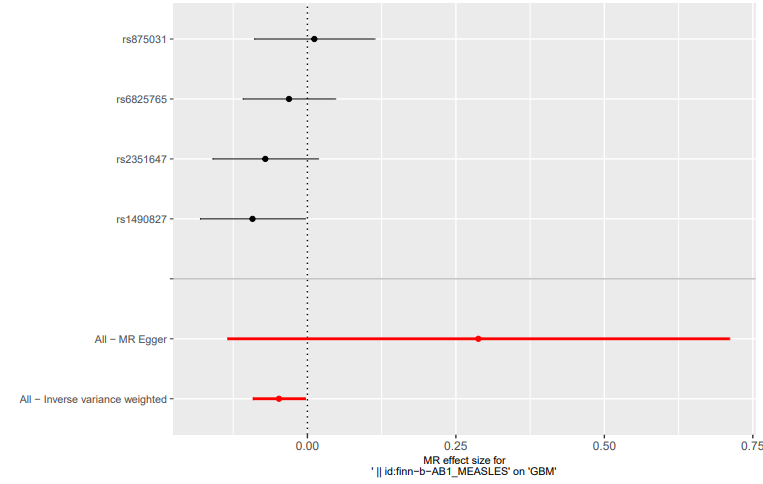


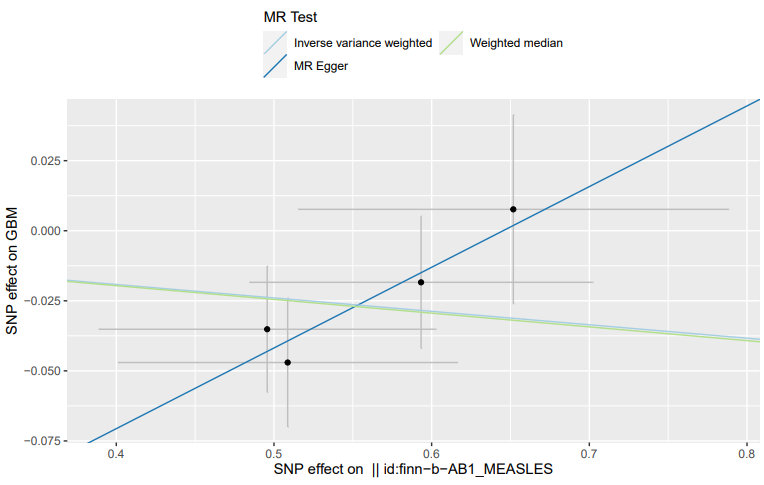


**Figure S33.** The leave-one-out plot, forest plot, and scatter plot for the association of measles infection and GBM in primary analysis. Data from 23andme.


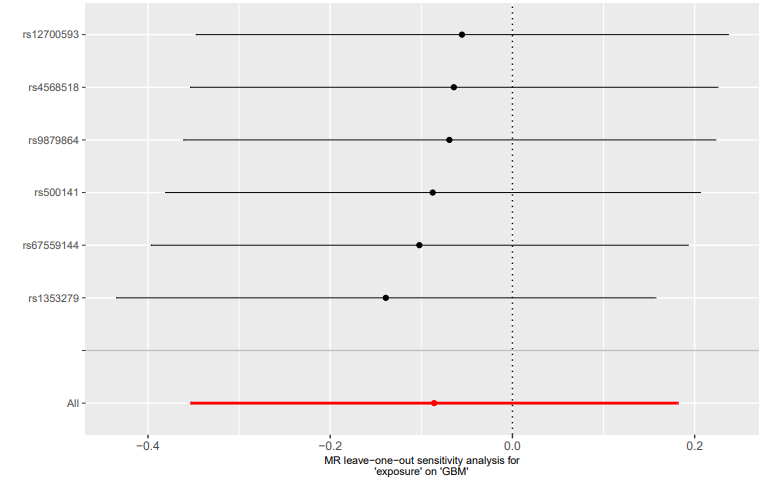


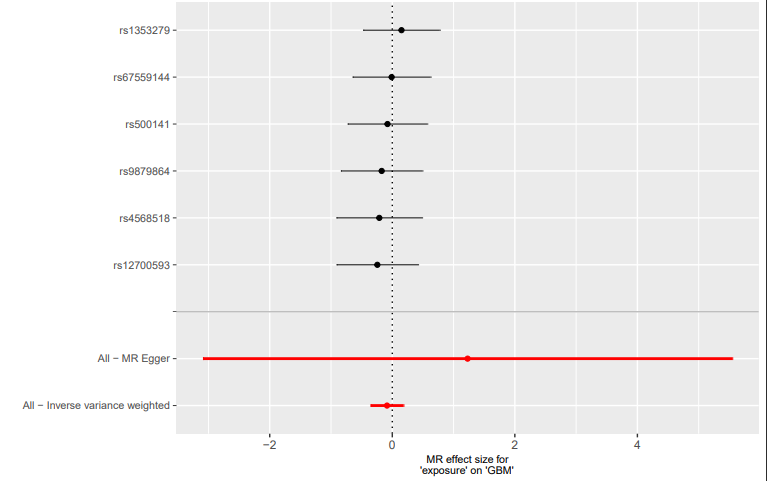


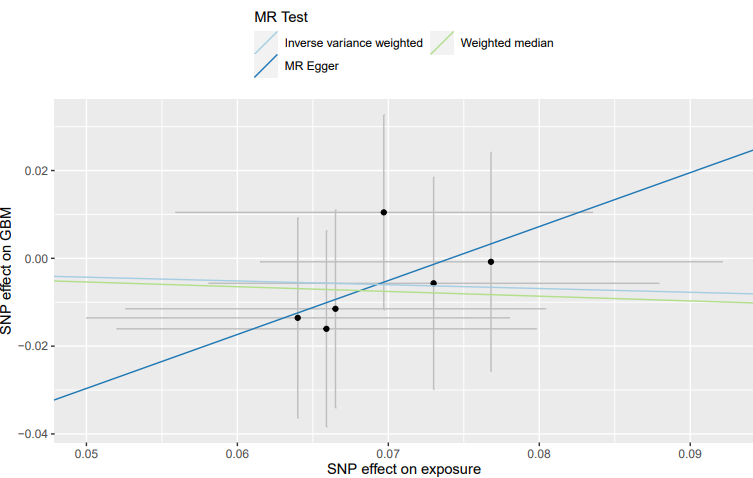


**Figure S34.** The leave-one-out plot, forest plot, and scatter plot for the association of Poliovirus infection and GBM in primary analysis. Data from FINN.


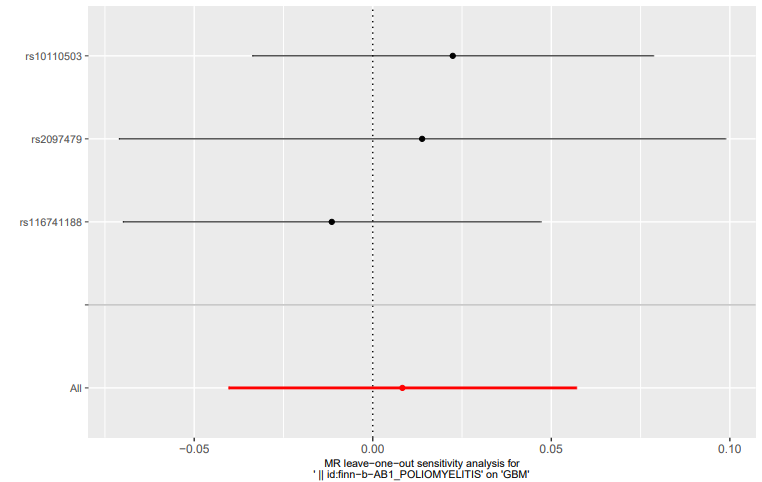


**Figure S35.** The leave-one-out plot, forest plot, and scatter plot for the association of Rubella infection and GBM in primary analysis. Data from FINN.

**Figure S36.** The leave-one-out plot, forest plot, and scatter plot for the association of Rubella infection and GBM in primary analysis. Data from 23andme.

**Figure S37.** The leave-one-out plot, forest plot, and scatter plot for the association of Herpes zoster and glioma in primary analysis. Date from FINN.

**Figure S38.** The leave-one-out plot, forest plot, and scatter plot for the association of mumps virus infection and glioma in primary analysis. Date from FINN.

**Figure S39.** The leave-one-out plot, forest plot, and scatter plot for the association of mumps virus infection and glioma in primary analysis. Data from 23andme.

**Figure S40.** The leave-one-out plot, forest plot, and scatter plot for the association of HSV infection and glioma in primary analysis. Data from FINN.

**Figure S41.** The leave-one-out plot, forest plot, and scatter plot for the association of HSV infection and glioma in primary analysis. Data from 23andme.

**Figure S42.** The leave-one-out plot, forest plot, and scatter plot for the association of HPV infection and glioma in primary analysis. Data from SUHRE.

**Figure S43.** The leave-one-out plot, forest plot, and scatter plot for the association of EBV infection and glioma in primary analysis. Data from FINN.

**Figure S44.** The leave-one-out plot, forest plot, and scatter plot for the association of EBV infection and glioma in primary analysis. Data from 23andme.

**Figure S45.** The leave-one-out plot, forest plot, and scatter plot for the association of COVID-19 infection and glioma in primary analysis. Data from COVID-19 HGI.

**Figure S46.** The leave-one-out plot, forest plot, and scatter plot for the association of HCMV infection and glioma in primary analysis. Data from FINN.

**Figure S47.** The leave-one-out plot, forest plot, and scatter plot for the association of Hepatitis infection and glioma in primary analysis. Data from FINN.

**Figure S48.** The leave-one-out plot, forest plot, and scatter plot for the association of Hepatitis infection and glioma in primary analysis. Data from 23andme.

**Figure S49.** The leave-one-out plot, forest plot, and scatter plot for the association of HIV infection and glioma in primary analysis. Data from FINN.

**Figure S50.** The leave-one-out plot, forest plot, and scatter plot for the association of measles infection and glioma in primary analysis. Data from FINN.

**Figure S51.** The leave-one-out plot, forest plot, and scatter plot for the association of measles infection and glioma in primary analysis. Data from 23andme.

**Figure S52.** The leave-one-out plot, forest plot, and scatter plot for the association of Poliovirus infection and glioma in primary analysis. Data from FINN.

**Figure S53.** The leave-one-out plot, forest plot, and scatter plot for the association of Rubella infection and glioma in primary analysis. Data from FINN.

**Figure S54.** The leave-one-out plot, forest plot, and scatter plot for the association of Rubella infection and glioma in primary analysis. Data from 23andme.
