# Supplementary material for: Association between viral infections and glioma risk: a two-sample bidirectional Mendelian randomization analysis
Source: BMC Med. 2023 Dec 5;21:487. doi: 10.1186/s12916-023-03142-9 (PMC10698979; doi:10.1186/s12916-023-03142-9)

**Additional file 5. Estimated causal effect between genetically predicted herpes zoster and LGG.**


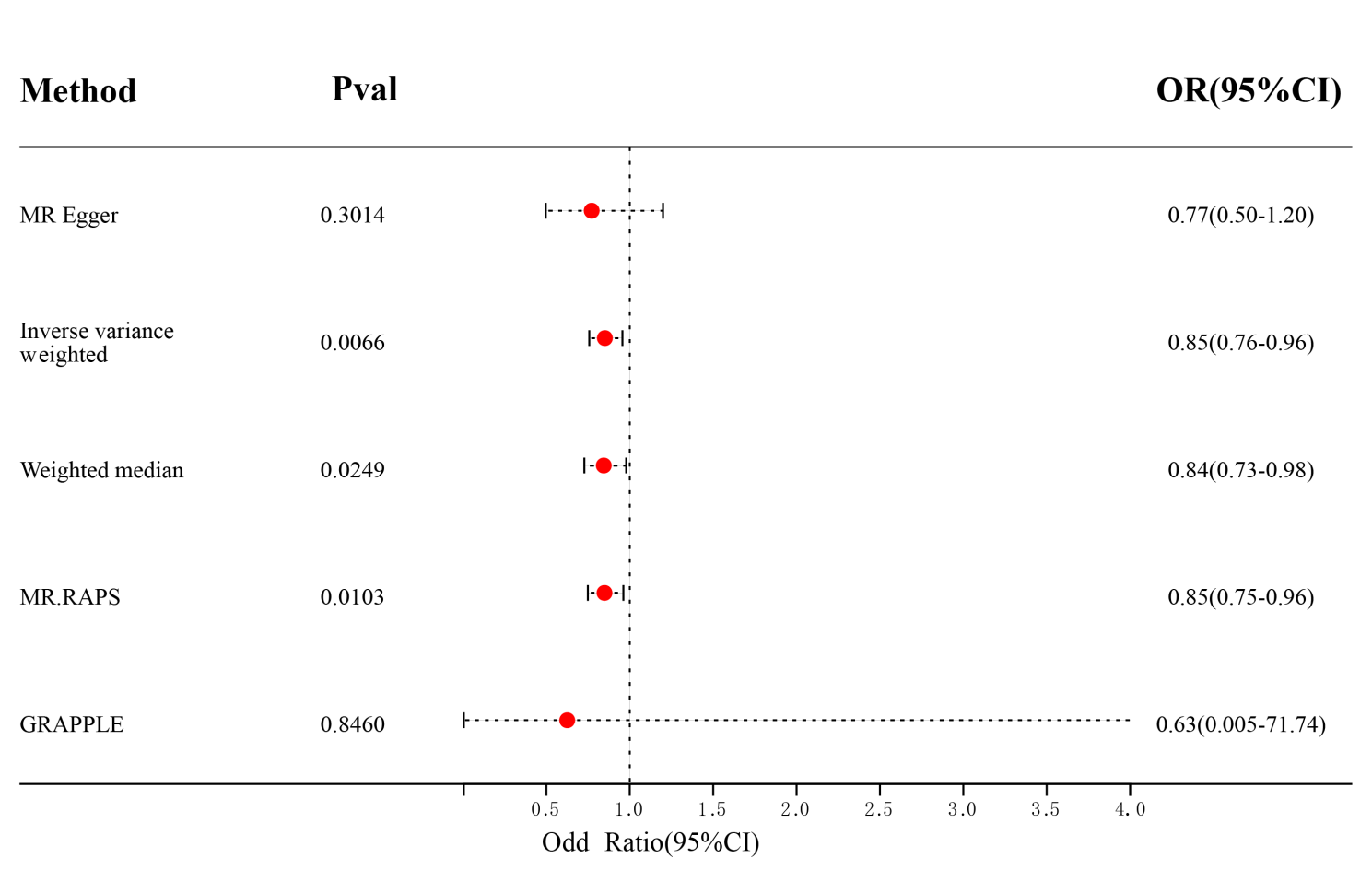

Supplement: Supplementary file 5 — Additional file 5. Estimated causal effect between genetically predicted herpes zoster and LGG. [file 12916_2023_3142_MOESM5_ESM.docx]
