# Supplementary material for: Association between viral infections and glioma risk: a two-sample bidirectional Mendelian randomization analysis
Source: BMC Med. 2023 Dec 5;21:487. doi: 10.1186/s12916-023-03142-9 (PMC10698979; doi:10.1186/s12916-023-03142-9)
Supplement: Supplementary file 6 — Additional file 6: Table S1. Single SNP analysis of the association of LGG on viral infection. Table S2. Single SNP analysis of the association of GBM on viral infection. Table S3. Single SNP analysis of the association of all-glioma on viral infection. [file 12916_2023_3142_MOESM6_ESM.docx]

**Additional file 6: Table S1**. Single SNP analysis of the association of LGG on viral infection

|  |  |  |  |  | **LGG** | | |  | **Virus infection** | | |  |
| --- | --- | --- | --- | --- | --- | --- | --- | --- | --- | --- | --- | --- |
| Outcome | SNP | Effect allele | Other allele | Effect allele frequency | Beta | Standard error | P-value |  | Beta * | Standard error * | P-value * | F-statistics |
| Cytomegaloviral disease | rs10990329 | A | C | 0.08924 | 0.186506 | 0.0372917 | 5.70E-07 |  | 0.0816 | 0.1508 | 0.5883 | 25.01273904 |
| Cytomegaloviral disease | rs11156763 | A | C | 0.818 | -0.194129 | 0.0418207 | 3.45E-06 |  | 0.0315 | 0.1141 | 0.7824 | 21.54756697 |
| Cytomegaloviral disease | rs11180712 | G | T | 0.5809 | 0.112038 | 0.0238794 | 2.71E-06 |  | -0.0474 | 0.0873 | 0.5875 | 22.01323523 |
| Cytomegaloviral disease | rs11576512 | A | G | 0.1714 | -0.158627 | 0.0315456 | 4.94E-07 |  | 0.0698 | 0.1166 | 0.549301 | 25.28579644 |
| Cytomegaloviral disease | rs11599775 | A | G | 0.3693 | -0.149069 | 0.0250404 | 2.63E-09 |  | -0.1329 | 0.0893 | 0.1367 | 35.4398726 |
| Cytomegaloviral disease | rs11642807 | T | C | 0.09835 | 0.2601 | 0.0466597 | 2.48E-08 |  | 0.1894 | 0.146 | 0.1946 | 31.07397558 |
| Cytomegaloviral disease | rs11703757 | T | C | 0.3812 | 0.131146 | 0.0268635 | 1.05E-06 |  | -0.0089 | 0.0892 | 0.9205 | 23.83333991 |
| Cytomegaloviral disease | rs117248307 | T | C | 0.01477 | -0.475794 | 0.0876951 | 5.78E-08 |  | -0.8935 | 0.3606 | 0.01322 | 29.43657427 |
| Cytomegaloviral disease | rs117666374 | G | A | 0.0929 | 0.184402 | 0.0391675 | 2.50E-06 |  | -0.2609 | 0.1481 | 0.0782204 | 22.16560297 |
| Cytomegaloviral disease | rs117679873 | A | G | 0.02838 | 0.279636 | 0.0605221 | 3.83E-06 |  | 0.0192 | 0.2571 | 0.9404 | 21.3480487 |
| Cytomegaloviral disease | rs11867655 | A | G | 0.2469 | 0.117365 | 0.0257044 | 4.97E-06 |  | 0.1204 | 0.0999 | 0.2281 | 20.84789756 |
| Cytomegaloviral disease | rs12548531 | T | C | 0.2006 | -0.146294 | 0.0312939 | 2.94E-06 |  | -0.1026 | 0.1082 | 0.343 | 21.85413635 |
| Cytomegaloviral disease | rs146122326 | A | G | 0.03015 | 0.426881 | 0.0875854 | 1.09E-06 |  | 0.0917 | 0.2558 | 0.719999 | 23.75473638 |
| Cytomegaloviral disease | rs146525470 | T | C | 0.1507 | -0.172127 | 0.0355047 | 1.25E-06 |  | 0.346 | 0.1213 | 0.004345 | 23.50316255 |
| Cytomegaloviral disease | rs17058678 | A | G | 0.1053 | 0.177264 | 0.0343305 | 2.42E-07 |  | -2.00E-04 | 0.1406 | 0.9991 | 26.66126998 |
| Cytomegaloviral disease | rs2076438 | C | T | 0.3621 | 0.114207 | 0.0248548 | 4.33E-06 |  | 0.254 | 0.0899 | 0.00472302 | 21.11372698 |
| Cytomegaloviral disease | rs2676248 | A | G | 0.08127 | 0.293541 | 0.0618352 | 2.06E-06 |  | 0.0503 | 0.1613 | 0.754899 | 22.53543778 |
| Cytomegaloviral disease | rs34018810 | A | G | 0.1003 | 0.181732 | 0.0392026 | 3.56E-06 |  | 0.2154 | 0.1437 | 0.1339 | 21.48983444 |
| Cytomegaloviral disease | rs34181263 | T | C | 0.5186 | -0.116789 | 0.023978 | 1.11E-06 |  | -0.0895 | 0.086 | 0.2979 | 23.72345661 |
| Cytomegaloviral disease | rs34575089 | A | G | 0.1398 | -0.186875 | 0.0324252 | 8.25E-09 |  | -0.0059 | 0.1239 | 0.9623 | 33.21521625 |
| Cytomegaloviral disease | rs3731239 | G | A | 0.4274 | 0.122696 | 0.0244652 | 5.30E-07 |  | -0.0327 | 0.0873 | 0.708101 | 25.15146394 |
| Cytomegaloviral disease | rs4146968 | A | G | 0.8581 | 0.160798 | 0.0347692 | 3.75E-06 |  | 0.0791 | 0.1234 | 0.521601 | 21.38808439 |
| Cytomegaloviral disease | rs536256 | G | A | 0.1883 | -0.18939 | 0.032362 | 4.85E-09 |  | 0.0813 | 0.1109 | 0.4635 | 34.24864398 |
| Cytomegaloviral disease | rs575654 | T | G | 0.4334 | -0.145374 | 0.0255507 | 1.27E-08 |  | 0.0332 | 0.0885 | 0.707301 | 32.37187651 |
| Cytomegaloviral disease | rs6089946 | G | A | 0.1826 | -0.146069 | 0.030473 | 1.64E-06 |  | 0.1846 | 0.1122 | 0.099901 | 22.97659604 |
| Cytomegaloviral disease | rs6106386 | C | T | 0.6779 | -0.117002 | 0.0251639 | 3.33E-06 |  | -0.0336 | 0.0924 | 0.7165 | 21.6187545 |
| Cytomegaloviral disease | rs62191977 | G | T | 0.7282 | 0.185119 | 0.0355407 | 1.90E-07 |  | 7.00E-04 | 0.0989 | 0.9941 | 27.13001517 |
| Cytomegaloviral disease | rs62239496 | G | A | 0.2055 | -0.124096 | 0.0261293 | 2.04E-06 |  | 0.0226 | 0.1067 | 0.8323 | 22.55589248 |
| Cytomegaloviral disease | rs62245373 | T | G | 0.3516 | 0.116167 | 0.024162 | 1.53E-06 |  | 0.1622 | 0.0903 | 0.0724102 | 23.11531349 |
| Cytomegaloviral disease | rs627291 | A | G | 0.7846 | -0.130782 | 0.0281935 | 3.51E-06 |  | 0.0672 | 0.1056 | 0.5248 | 21.51780474 |
| Cytomegaloviral disease | rs66892237 | G | A | 0.07871 | -0.202468 | 0.0410236 | 8.00E-07 |  | 0.0724 | 0.1601 | 0.6513 | 24.35820313 |
| Cytomegaloviral disease | rs67241126 | A | G | 0.1789 | 0.171995 | 0.0358622 | 1.62E-06 |  | 0.0393 | 0.1133 | 0.7286 | 23.00158626 |
| Cytomegaloviral disease | rs7093945 | C | T | 0.7739 | -0.145999 | 0.0272641 | 8.56E-08 |  | -0.1003 | 0.103 | 0.3302 | 28.67592463 |
| Cytomegaloviral disease | rs725076 | T | G | 0.6781 | 0.128304 | 0.0278445 | 4.07E-06 |  | 0.0229 | 0.0924 | 0.8044 | 21.23252015 |
| Cytomegaloviral disease | rs72712509 | C | T | 0.1061 | 0.266864 | 0.0504984 | 1.26E-07 |  | -0.0306 | 0.14 | 0.827 | 27.92702968 |
| Cytomegaloviral disease | rs72835696 | A | G | 0.006801 | 0.402584 | 0.0856012 | 2.56E-06 |  | 0.3458 | 0.5259 | 0.5108 | 22.11838002 |
| Cytomegaloviral disease | rs75061358 | G | T | 0.1055 | 0.24542 | 0.0444095 | 3.27E-08 |  | -0.0878 | 0.1409 | 0.533301 | 30.53993697 |
| Cytomegaloviral disease | rs797974 | C | T | 0.4097 | -0.112245 | 0.0243397 | 4.00E-06 |  | -0.1532 | 0.0877 | 0.0808407 | 21.26686952 |
| Cytomegaloviral disease | rs926771 | T | C | 0.07873 | -0.288211 | 0.0436426 | 4.01E-11 |  | 0.0244 | 0.1604 | 0.8792 | 43.6113844 |
| Cytomegaloviral disease | rs989993 | A | C | 0.04638 | 0.380402 | 0.0812247 | 2.82E-06 |  | 0.0303 | 0.2049 | 0.8826 | 21.93357133 |
| COVID-19 hospitalized | rs10990329 | A | C | 0.1132 | 0.186506 | 0.0372917 | 5.70E-07 |  | -0.006437 | 0.014848 | 0.664599 | 25.01273904 |
| COVID-19 hospitalized | rs11156763 | A | C | 0.8289 | -0.194129 | 0.0418207 | 3.45E-06 |  | 0.018155 | 0.016676 | 0.2763 | 21.54756697 |
| COVID-19 hospitalized | rs11180712 | G | T | 0.558 | 0.112038 | 0.0238794 | 2.71E-06 |  | -0.003655 | 0.0094944 | 0.7003 | 22.01323523 |
| COVID-19 hospitalized | rs11599775 | A | G | 0.3758 | -0.149069 | 0.0250404 | 2.63E-09 |  | -0.0095488 | 0.010153 | 0.347 | 35.4398726 |
| COVID-19 hospitalized | rs11642807 | T | C | 0.08762 | 0.2601 | 0.0466597 | 2.48E-08 |  | -0.020918 | 0.018691 | 0.2631 | 31.07397558 |
| COVID-19 hospitalized | rs11703757 | T | C | 0.3299 | 0.131146 | 0.0268635 | 1.05E-06 |  | 0.0012632 | 0.010981 | 0.9084 | 23.83333991 |
| COVID-19 hospitalized | rs117098244 | C | T | 0.03002 | 0.432979 | 0.0882587 | 9.30E-07 |  | -0.011027 | 0.036697 | 0.763801 | 24.06681467 |
| COVID-19 hospitalized | rs117248307 | T | C | 0.03024 | -0.475794 | 0.0876951 | 5.78E-08 |  | 0.0044196 | 0.032132 | 0.8906 | 29.43657427 |
| COVID-19 hospitalized | rs117666374 | G | A | 0.1045 | 0.184402 | 0.0391675 | 2.50E-06 |  | 0.034184 | 0.015663 | 0.0290797 | 22.16560297 |
| COVID-19 hospitalized | rs117679873 | A | G | 0.04716 | 0.279636 | 0.0605221 | 3.83E-06 |  | -0.0066866 | 0.024036 | 0.780899 | 21.3480487 |
| COVID-19 hospitalized | rs11867655 | A | G | 0.3261 | 0.117365 | 0.0257044 | 4.97E-06 |  | 0.0053435 | 0.01006 | 0.5953 | 20.84789756 |
| COVID-19 hospitalized | rs12548531 | T | C | 0.1766 | -0.146294 | 0.0312939 | 2.94E-06 |  | -0.017382 | 0.012375 | 0.1601 | 21.85413635 |
| COVID-19 hospitalized | rs146122326 | A | G | 0.03316 | 0.426881 | 0.0875854 | 1.09E-06 |  | -0.013993 | 0.035417 | 0.6928 | 23.75473638 |
| COVID-19 hospitalized | rs146525470 | T | C | 0.1561 | -0.172127 | 0.0355047 | 1.25E-06 |  | -0.0010732 | 0.014115 | 0.9394 | 23.50316255 |
| COVID-19 hospitalized | rs17058678 | A | G | 0.1323 | 0.177264 | 0.0343305 | 2.42E-07 |  | -0.010468 | 0.013965 | 0.4535 | 26.66126998 |
| COVID-19 hospitalized | rs192765600 | A | G | 0.02264 | 0.494052 | 0.101915 | 1.25E-06 |  | 0.03204 | 0.044678 | 0.473301 | 23.50006729 |
| COVID-19 hospitalized | rs2076438 | C | T | 0.3623 | 0.114207 | 0.0248548 | 4.33E-06 |  | -0.0064953 | 0.010106 | 0.5204 | 21.11372698 |
| COVID-19 hospitalized | rs2676248 | A | G | 0.05573 | 0.293541 | 0.0618352 | 2.06E-06 |  | 0.057394 | 0.025882 | 0.0265901 | 22.53543778 |
| COVID-19 hospitalized | rs34018810 | A | G | 0.1075 | 0.181732 | 0.0392026 | 3.56E-06 |  | -0.015125 | 0.01556 | 0.331 | 21.48983444 |
| COVID-19 hospitalized | rs34181263 | T | C | 0.5025 | -0.116789 | 0.023978 | 1.11E-06 |  | -0.024391 | 0.0097289 | 0.01217 | 23.72345661 |
| COVID-19 hospitalized | rs34575089 | A | G | 0.159 | -0.186875 | 0.0324252 | 8.25E-09 |  | -0.014058 | 0.012817 | 0.2727 | 33.21521625 |
| COVID-19 hospitalized | rs3731239 | G | A | 0.3874 | 0.122696 | 0.0244652 | 5.30E-07 |  | 0.006681 | 0.010111 | 0.5088 | 25.15146394 |
| COVID-19 hospitalized | rs4146968 | A | G | 0.8436 | 0.160798 | 0.0347692 | 3.75E-06 |  | 0.020386 | 0.014034 | 0.1463 | 21.38808439 |
| COVID-19 hospitalized | rs536256 | G | A | 0.1811 | -0.18939 | 0.032362 | 4.85E-09 |  | -0.00082769 | 0.013193 | 0.95 | 34.24864398 |
| COVID-19 hospitalized | rs575654 | T | G | 0.4573 | -0.145374 | 0.0255507 | 1.27E-08 |  | -0.021564 | 0.010854 | 0.0469602 | 32.37187651 |
| COVID-19 hospitalized | rs6089946 | G | A | 0.21 | -0.146069 | 0.030473 | 1.64E-06 |  | -0.0034748 | 0.012245 | 0.776599 | 22.97659604 |
| COVID-19 hospitalized | rs6106386 | C | T | 0.6557 | -0.117002 | 0.0251639 | 3.33E-06 |  | 0.014491 | 0.010268 | 0.1582 | 21.6187545 |
| COVID-19 hospitalized | rs62191977 | G | T | 0.6499 | 0.185119 | 0.0355407 | 1.90E-07 |  | -0.00977 | 0.011191 | 0.3827 | 27.13001517 |
| COVID-19 hospitalized | rs62239496 | G | A | 0.2785 | -0.124096 | 0.0261293 | 2.04E-06 |  | -0.0050386 | 0.010748 | 0.6392 | 22.55589248 |
| COVID-19 hospitalized | rs62245373 | T | G | 0.4112 | 0.116167 | 0.024162 | 1.53E-06 |  | -0.002336 | 0.0098549 | 0.8126 | 23.11531349 |
| COVID-19 hospitalized | rs627291 | A | G | 0.7478 | -0.130782 | 0.0281935 | 3.51E-06 |  | -0.0030407 | 0.011903 | 0.7984 | 21.51780474 |
| COVID-19 hospitalized | rs66892237 | G | A | 0.09944 | -0.202468 | 0.0410236 | 8.00E-07 |  | 0.012353 | 0.016037 | 0.4411 | 24.35820313 |
| COVID-19 hospitalized | rs67241126 | A | G | 0.1427 | 0.171995 | 0.0358622 | 1.62E-06 |  | -0.0022886 | 0.01462 | 0.8756 | 23.00158626 |
| COVID-19 hospitalized | rs7093945 | C | T | 0.7248 | -0.145999 | 0.0272641 | 8.56E-08 |  | -0.010465 | 0.011382 | 0.3579 | 28.67592463 |
| COVID-19 hospitalized | rs725076 | T | G | 0.7324 | 0.128304 | 0.0278445 | 4.07E-06 |  | -0.0092882 | 0.011405 | 0.4154 | 21.23252015 |
| COVID-19 hospitalized | rs72712509 | C | T | 0.08334 | 0.266864 | 0.0504984 | 1.26E-07 |  | 0.010255 | 0.019971 | 0.6076 | 27.92702968 |
| COVID-19 hospitalized | rs72835696 | A | G | 0.02758 | 0.402584 | 0.0856012 | 2.56E-06 |  | 0.0085837 | 0.033946 | 0.8004 | 22.11838002 |
| COVID-19 hospitalized | rs75061358 | G | T | 0.0902 | 0.24542 | 0.0444095 | 3.27E-08 |  | -0.024528 | 0.017895 | 0.1705 | 30.53993697 |
| COVID-19 hospitalized | rs797974 | C | T | 0.4323 | -0.112245 | 0.0243397 | 4.00E-06 |  | 0.0056393 | 0.010128 | 0.5776 | 21.26686952 |
| COVID-19 hospitalized | rs926771 | T | C | 0.08755 | -0.288211 | 0.0436426 | 4.01E-11 |  | 0.01349 | 0.01736 | 0.4371 | 43.6113844 |
| COVID-19 hospitalized | rs989993 | A | C | 0.03769 | 0.380402 | 0.0812247 | 2.82E-06 |  | 0.04359 | 0.02785 | 0.1175 | 21.93357133 |
| Infectious mononucleosis | rs10990329 | A | C | 0.08926 | 0.186506 | 0.0372917 | 5.70E-07 |  | 0.0447 | 0.0716 | 0.5324 | 25.01273904 |
| Infectious mononucleosis | rs11156763 | A | C | 0.818 | -0.194129 | 0.0418207 | 3.45E-06 |  | -0.0109 | 0.0541 | 0.8408 | 21.54756697 |
| Infectious mononucleosis | rs11180712 | G | T | 0.5809 | 0.112038 | 0.0238794 | 2.71E-06 |  | 0.0226 | 0.0415 | 0.5864 | 22.01323523 |
| Infectious mononucleosis | rs11576512 | A | G | 0.1714 | -0.158627 | 0.0315456 | 4.94E-07 |  | -0.0077 | 0.0552 | 0.8895 | 25.28579644 |
| Infectious mononucleosis | rs11599775 | A | G | 0.3691 | -0.149069 | 0.0250404 | 2.63E-09 |  | -0.1297 | 0.0425 | 0.00228502 | 35.4398726 |
| Infectious mononucleosis | rs11642807 | T | C | 0.0983 | 0.2601 | 0.0466597 | 2.48E-08 |  | -0.0662 | 0.0696 | 0.3412 | 31.07397558 |
| Infectious mononucleosis | rs11703757 | T | C | 0.3811 | 0.131146 | 0.0268635 | 1.05E-06 |  | -0.0187 | 0.0424 | 0.6595 | 23.83333991 |
| Infectious mononucleosis | rs117248307 | T | C | 0.01479 | -0.475794 | 0.0876951 | 5.78E-08 |  | 0.1191 | 0.1706 | 0.4852 | 29.43657427 |
| Infectious mononucleosis | rs117666374 | G | A | 0.09292 | 0.184402 | 0.0391675 | 2.50E-06 |  | -0.0506 | 0.07 | 0.4698 | 22.16560297 |
| Infectious mononucleosis | rs117679873 | A | G | 0.02838 | 0.279636 | 0.0605221 | 3.83E-06 |  | -0.0515 | 0.122 | 0.672699 | 21.3480487 |
| Infectious mononucleosis | rs11867655 | A | G | 0.2469 | 0.117365 | 0.0257044 | 4.97E-06 |  | 0.0254 | 0.0474 | 0.5914 | 20.84789756 |
| Infectious mononucleosis | rs12548531 | T | C | 0.2006 | -0.146294 | 0.0312939 | 2.94E-06 |  | 0.01 | 0.0514 | 0.846 | 21.85413635 |
| Infectious mononucleosis | rs146122326 | A | G | 0.03015 | 0.426881 | 0.0875854 | 1.09E-06 |  | -0.0253 | 0.1209 | 0.834 | 23.75473638 |
| Infectious mononucleosis | rs146525470 | T | C | 0.1506 | -0.172127 | 0.0355047 | 1.25E-06 |  | -0.0813 | 0.0575 | 0.1575 | 23.50316255 |
| Infectious mononucleosis | rs17058678 | A | G | 0.1052 | 0.177264 | 0.0343305 | 2.42E-07 |  | -0.0573 | 0.0665 | 0.3887 | 26.66126998 |
| Infectious mononucleosis | rs2076438 | C | T | 0.362 | 0.114207 | 0.0248548 | 4.33E-06 |  | -0.0238 | 0.0427 | 0.5767 | 21.11372698 |
| Infectious mononucleosis | rs2676248 | A | G | 0.08129 | 0.293541 | 0.0618352 | 2.06E-06 |  | 0.0634 | 0.0766 | 0.4076 | 22.53543778 |
| Infectious mononucleosis | rs34018810 | A | G | 0.1002 | 0.181732 | 0.0392026 | 3.56E-06 |  | -0.0269 | 0.0682 | 0.6937 | 21.48983444 |
| Infectious mononucleosis | rs34181263 | T | C | 0.5185 | -0.116789 | 0.023978 | 1.11E-06 |  | -0.0803 | 0.0408 | 0.04923 | 23.72345661 |
| Infectious mononucleosis | rs34575089 | A | G | 0.1397 | -0.186875 | 0.0324252 | 8.25E-09 |  | -0.0951 | 0.059 | 0.1066 | 33.21521625 |
| Infectious mononucleosis | rs3731239 | G | A | 0.4273 | 0.122696 | 0.0244652 | 5.30E-07 |  | -0.1178 | 0.0415 | 0.00450402 | 25.15146394 |
| Infectious mononucleosis | rs4146968 | A | G | 0.858 | 0.160798 | 0.0347692 | 3.75E-06 |  | -0.0906 | 0.0586 | 0.122 | 21.38808439 |
| Infectious mononucleosis | rs536256 | G | A | 0.1883 | -0.18939 | 0.032362 | 4.85E-09 |  | 0.0225 | 0.0525 | 0.668599 | 34.24864398 |
| Infectious mononucleosis | rs575654 | T | G | 0.4333 | -0.145374 | 0.0255507 | 1.27E-08 |  | -0.0449 | 0.042 | 0.2846 | 32.37187651 |
| Infectious mononucleosis | rs6089946 | G | A | 0.1826 | -0.146069 | 0.030473 | 1.64E-06 |  | 0.0666 | 0.0532 | 0.2105 | 22.97659604 |
| Infectious mononucleosis | rs6106386 | C | T | 0.6779 | -0.117002 | 0.0251639 | 3.33E-06 |  | -0.0029 | 0.0438 | 0.9478 | 21.6187545 |
| Infectious mononucleosis | rs62191977 | G | T | 0.7282 | 0.185119 | 0.0355407 | 1.90E-07 |  | 0.0211 | 0.0468 | 0.6516 | 27.13001517 |
| Infectious mononucleosis | rs62239496 | G | A | 0.2055 | -0.124096 | 0.0261293 | 2.04E-06 |  | 0.0472 | 0.0505 | 0.35 | 22.55589248 |
| Infectious mononucleosis | rs62245373 | T | G | 0.3516 | 0.116167 | 0.024162 | 1.53E-06 |  | -0.0087 | 0.0429 | 0.8384 | 23.11531349 |
| Infectious mononucleosis | rs627291 | A | G | 0.7845 | -0.130782 | 0.0281935 | 3.51E-06 |  | -0.0514 | 0.05 | 0.3041 | 21.51780474 |
| Infectious mononucleosis | rs66892237 | G | A | 0.07868 | -0.202468 | 0.0410236 | 8.00E-07 |  | -0.0812 | 0.0758 | 0.2835 | 24.35820313 |
| Infectious mononucleosis | rs67241126 | A | G | 0.179 | 0.171995 | 0.0358622 | 1.62E-06 |  | 0.0209 | 0.0539 | 0.698601 | 23.00158626 |
| Infectious mononucleosis | rs7093945 | C | T | 0.7739 | -0.145999 | 0.0272641 | 8.56E-08 |  | 0.0118 | 0.0491 | 0.8095 | 28.67592463 |
| Infectious mononucleosis | rs725076 | T | G | 0.678 | 0.128304 | 0.0278445 | 4.07E-06 |  | -0.0337 | 0.0437 | 0.4415 | 21.23252015 |
| Infectious mononucleosis | rs72712509 | C | T | 0.1062 | 0.266864 | 0.0504984 | 1.26E-07 |  | 0.0818 | 0.0666 | 0.2194 | 27.92702968 |
| Infectious mononucleosis | rs72835696 | A | G | 0.006792 | 0.402584 | 0.0856012 | 2.56E-06 |  | -0.1822 | 0.2499 | 0.466 | 22.11838002 |
| Infectious mononucleosis | rs75061358 | G | T | 0.1056 | 0.24542 | 0.0444095 | 3.27E-08 |  | 0.0683 | 0.0666 | 0.3049 | 30.53993697 |
| Infectious mononucleosis | rs797974 | C | T | 0.4097 | -0.112245 | 0.0243397 | 4.00E-06 |  | 0.0095 | 0.0415 | 0.8183 | 21.26686952 |
| Infectious mononucleosis | rs926771 | T | C | 0.07874 | -0.288211 | 0.0436426 | 4.01E-11 |  | 0.0245 | 0.0759 | 0.7472 | 43.6113844 |
| Infectious mononucleosis | rs989993 | A | C | 0.04634 | 0.380402 | 0.0812247 | 2.82E-06 |  | -0.1396 | 0.0982 | 0.1552 | 21.93357133 |
| Viral hepatitis | rs10990329 | A | C | 0.08926 | 0.186506 | 0.0372917 | 5.70E-07 |  | 0.0447 | 0.0716 | 0.5324 | 25.01273904 |
| Viral hepatitis | rs11156763 | A | C | 0.818 | -0.194129 | 0.0418207 | 3.45E-06 |  | -0.0109 | 0.0541 | 0.8408 | 21.54756697 |
| Viral hepatitis | rs11180712 | G | T | 0.5809 | 0.112038 | 0.0238794 | 2.71E-06 |  | 0.0226 | 0.0415 | 0.5864 | 22.01323523 |
| Viral hepatitis | rs11576512 | A | G | 0.1714 | -0.158627 | 0.0315456 | 4.94E-07 |  | -0.0077 | 0.0552 | 0.8895 | 25.28579644 |
| Viral hepatitis | rs11599775 | A | G | 0.3691 | -0.149069 | 0.0250404 | 2.63E-09 |  | -0.1297 | 0.0425 | 0.00228502 | 35.4398726 |
| Viral hepatitis | rs11642807 | T | C | 0.0983 | 0.2601 | 0.0466597 | 2.48E-08 |  | -0.0662 | 0.0696 | 0.3412 | 31.07397558 |
| Viral hepatitis | rs11703757 | T | C | 0.3811 | 0.131146 | 0.0268635 | 1.05E-06 |  | -0.0187 | 0.0424 | 0.6595 | 23.83333991 |
| Viral hepatitis | rs117248307 | T | C | 0.01479 | -0.475794 | 0.0876951 | 5.78E-08 |  | 0.1191 | 0.1706 | 0.4852 | 29.43657427 |
| Viral hepatitis | rs117666374 | G | A | 0.09292 | 0.184402 | 0.0391675 | 2.50E-06 |  | -0.0506 | 0.07 | 0.4698 | 22.16560297 |
| Viral hepatitis | rs117679873 | A | G | 0.02838 | 0.279636 | 0.0605221 | 3.83E-06 |  | -0.0515 | 0.122 | 0.672699 | 21.3480487 |
| Viral hepatitis | rs11867655 | A | G | 0.2469 | 0.117365 | 0.0257044 | 4.97E-06 |  | 0.0254 | 0.0474 | 0.5914 | 20.84789756 |
| Viral hepatitis | rs12548531 | T | C | 0.2006 | -0.146294 | 0.0312939 | 2.94E-06 |  | 0.01 | 0.0514 | 0.846 | 21.85413635 |
| Viral hepatitis | rs146122326 | A | G | 0.03015 | 0.426881 | 0.0875854 | 1.09E-06 |  | -0.0253 | 0.1209 | 0.834 | 23.75473638 |
| Viral hepatitis | rs146525470 | T | C | 0.1506 | -0.172127 | 0.0355047 | 1.25E-06 |  | -0.0813 | 0.0575 | 0.1575 | 23.50316255 |
| Viral hepatitis | rs17058678 | A | G | 0.1052 | 0.177264 | 0.0343305 | 2.42E-07 |  | -0.0573 | 0.0665 | 0.3887 | 26.66126998 |
| Viral hepatitis | rs2076438 | C | T | 0.362 | 0.114207 | 0.0248548 | 4.33E-06 |  | -0.0238 | 0.0427 | 0.5767 | 21.11372698 |
| Viral hepatitis | rs2676248 | A | G | 0.08129 | 0.293541 | 0.0618352 | 2.06E-06 |  | 0.0634 | 0.0766 | 0.4076 | 22.53543778 |
| Viral hepatitis | rs34018810 | A | G | 0.1002 | 0.181732 | 0.0392026 | 3.56E-06 |  | -0.0269 | 0.0682 | 0.6937 | 21.48983444 |
| Viral hepatitis | rs34181263 | T | C | 0.5185 | -0.116789 | 0.023978 | 1.11E-06 |  | -0.0803 | 0.0408 | 0.04923 | 23.72345661 |
| Viral hepatitis | rs34575089 | A | G | 0.1397 | -0.186875 | 0.0324252 | 8.25E-09 |  | -0.0951 | 0.059 | 0.1066 | 33.21521625 |
| Viral hepatitis | rs3731239 | G | A | 0.4273 | 0.122696 | 0.0244652 | 5.30E-07 |  | -0.1178 | 0.0415 | 0.00450402 | 25.15146394 |
| Viral hepatitis | rs4146968 | A | G | 0.858 | 0.160798 | 0.0347692 | 3.75E-06 |  | -0.0906 | 0.0586 | 0.122 | 21.38808439 |
| Viral hepatitis | rs536256 | G | A | 0.1883 | -0.18939 | 0.032362 | 4.85E-09 |  | 0.0225 | 0.0525 | 0.668599 | 34.24864398 |
| Viral hepatitis | rs575654 | T | G | 0.4333 | -0.145374 | 0.0255507 | 1.27E-08 |  | -0.0449 | 0.042 | 0.2846 | 32.37187651 |
| Viral hepatitis | rs6089946 | G | A | 0.1826 | -0.146069 | 0.030473 | 1.64E-06 |  | 0.0666 | 0.0532 | 0.2105 | 22.97659604 |
| Viral hepatitis | rs6106386 | C | T | 0.6779 | -0.117002 | 0.0251639 | 3.33E-06 |  | -0.0029 | 0.0438 | 0.9478 | 21.6187545 |
| Viral hepatitis | rs62191977 | G | T | 0.7282 | 0.185119 | 0.0355407 | 1.90E-07 |  | 0.0211 | 0.0468 | 0.6516 | 27.13001517 |
| Viral hepatitis | rs62239496 | G | A | 0.2055 | -0.124096 | 0.0261293 | 2.04E-06 |  | 0.0472 | 0.0505 | 0.35 | 22.55589248 |
| Viral hepatitis | rs62245373 | T | G | 0.3516 | 0.116167 | 0.024162 | 1.53E-06 |  | -0.0087 | 0.0429 | 0.8384 | 23.11531349 |
| Viral hepatitis | rs627291 | A | G | 0.7845 | -0.130782 | 0.0281935 | 3.51E-06 |  | -0.0514 | 0.05 | 0.3041 | 21.51780474 |
| Viral hepatitis | rs66892237 | G | A | 0.07868 | -0.202468 | 0.0410236 | 8.00E-07 |  | -0.0812 | 0.0758 | 0.2835 | 24.35820313 |
| Viral hepatitis | rs67241126 | A | G | 0.179 | 0.171995 | 0.0358622 | 1.62E-06 |  | 0.0209 | 0.0539 | 0.698601 | 23.00158626 |
| Viral hepatitis | rs7093945 | C | T | 0.7739 | -0.145999 | 0.0272641 | 8.56E-08 |  | 0.0118 | 0.0491 | 0.8095 | 28.67592463 |
| Viral hepatitis | rs725076 | T | G | 0.678 | 0.128304 | 0.0278445 | 4.07E-06 |  | -0.0337 | 0.0437 | 0.4415 | 21.23252015 |
| Viral hepatitis | rs72712509 | C | T | 0.1062 | 0.266864 | 0.0504984 | 1.26E-07 |  | 0.0818 | 0.0666 | 0.2194 | 27.92702968 |
| Viral hepatitis | rs72835696 | A | G | 0.006792 | 0.402584 | 0.0856012 | 2.56E-06 |  | -0.1822 | 0.2499 | 0.466 | 22.11838002 |
| Viral hepatitis | rs75061358 | G | T | 0.1056 | 0.24542 | 0.0444095 | 3.27E-08 |  | 0.0683 | 0.0666 | 0.3049 | 30.53993697 |
| Viral hepatitis | rs797974 | C | T | 0.4097 | -0.112245 | 0.0243397 | 4.00E-06 |  | 0.0095 | 0.0415 | 0.8183 | 21.26686952 |
| Viral hepatitis | rs926771 | T | C | 0.07874 | -0.288211 | 0.0436426 | 4.01E-11 |  | 0.0245 | 0.0759 | 0.7472 | 43.6113844 |
| Viral hepatitis | rs989993 | A | C | 0.04634 | 0.380402 | 0.0812247 | 2.82E-06 |  | -0.1396 | 0.0982 | 0.1552 | 21.93357133 |
| Herpesviral infections | rs10990329 | A | C | 0.08919 | 0.186506 | 0.0372917 | 5.70E-07 |  | -0.0653 | 0.0624 | 0.2955 | 25.01273904 |
| Herpesviral infections | rs11156763 | A | C | 0.818 | -0.194129 | 0.0418207 | 3.45E-06 |  | -0.0206 | 0.0472 | 0.6623 | 21.54756697 |
| Herpesviral infections | rs11180712 | G | T | 0.5808 | 0.112038 | 0.0238794 | 2.71E-06 |  | -0.0091 | 0.0361 | 0.8013 | 22.01323523 |
| Herpesviral infections | rs11576512 | A | G | 0.1714 | -0.158627 | 0.0315456 | 4.94E-07 |  | -0.0894 | 0.0483 | 0.06418 | 25.28579644 |
| Herpesviral infections | rs11599775 | A | G | 0.3692 | -0.149069 | 0.0250404 | 2.63E-09 |  | -0.0254 | 0.037 | 0.4919 | 35.4398726 |
| Herpesviral infections | rs11642807 | T | C | 0.09828 | 0.2601 | 0.0466597 | 2.48E-08 |  | -0.0535 | 0.0608 | 0.3787 | 31.07397558 |
| Herpesviral infections | rs11703757 | T | C | 0.3811 | 0.131146 | 0.0268635 | 1.05E-06 |  | 0.0101 | 0.0368 | 0.784899 | 23.83333991 |
| Herpesviral infections | rs117248307 | T | C | 0.01483 | -0.475794 | 0.0876951 | 5.78E-08 |  | -0.0718 | 0.1481 | 0.6278 | 29.43657427 |
| Herpesviral infections | rs117666374 | G | A | 0.09298 | 0.184402 | 0.0391675 | 2.50E-06 |  | -0.0497 | 0.0616 | 0.4197 | 22.16560297 |
| Herpesviral infections | rs117679873 | A | G | 0.02848 | 0.279636 | 0.0605221 | 3.83E-06 |  | 0.195 | 0.1079 | 0.0706903 | 21.3480487 |
| Herpesviral infections | rs11867655 | A | G | 0.2468 | 0.117365 | 0.0257044 | 4.97E-06 |  | 0.0777 | 0.0412 | 0.0596802 | 20.84789756 |
| Herpesviral infections | rs12548531 | T | C | 0.2006 | -0.146294 | 0.0312939 | 2.94E-06 |  | -9.00E-04 | 0.0446 | 0.9841 | 21.85413635 |
| Herpesviral infections | rs146122326 | A | G | 0.03014 | 0.426881 | 0.0875854 | 1.09E-06 |  | -0.033 | 0.1062 | 0.756 | 23.75473638 |
| Herpesviral infections | rs146525470 | T | C | 0.1506 | -0.172127 | 0.0355047 | 1.25E-06 |  | -0.0173 | 0.0499 | 0.7281 | 23.50316255 |
| Herpesviral infections | rs17058678 | A | G | 0.1054 | 0.177264 | 0.0343305 | 2.42E-07 |  | -0.0164 | 0.0583 | 0.778901 | 26.66126998 |
| Herpesviral infections | rs2076438 | C | T | 0.3619 | 0.114207 | 0.0248548 | 4.33E-06 |  | 0.0323 | 0.0371 | 0.3828 | 21.11372698 |
| Herpesviral infections | rs2676248 | A | G | 0.08128 | 0.293541 | 0.0618352 | 2.06E-06 |  | 0.0025 | 0.0659 | 0.9699 | 22.53543778 |
| Herpesviral infections | rs34018810 | A | G | 0.1002 | 0.181732 | 0.0392026 | 3.56E-06 |  | 0.0541 | 0.0591 | 0.3604 | 21.48983444 |
| Herpesviral infections | rs34181263 | T | C | 0.5185 | -0.116789 | 0.023978 | 1.11E-06 |  | -0.0275 | 0.0355 | 0.4384 | 23.72345661 |
| Herpesviral infections | rs34575089 | A | G | 0.1398 | -0.186875 | 0.0324252 | 8.25E-09 |  | 0.0068 | 0.051 | 0.8944 | 33.21521625 |
| Herpesviral infections | rs3731239 | G | A | 0.4272 | 0.122696 | 0.0244652 | 5.30E-07 |  | -0.009 | 0.0361 | 0.8036 | 25.15146394 |
| Herpesviral infections | rs4146968 | A | G | 0.858 | 0.160798 | 0.0347692 | 3.75E-06 |  | -0.0126 | 0.0509 | 0.8038 | 21.38808439 |
| Herpesviral infections | rs536256 | G | A | 0.1881 | -0.18939 | 0.032362 | 4.85E-09 |  | -0.0562 | 0.0457 | 0.218 | 34.24864398 |
| Herpesviral infections | rs575654 | T | G | 0.4333 | -0.145374 | 0.0255507 | 1.27E-08 |  | -0.079 | 0.0365 | 0.0305197 | 32.37187651 |
| Herpesviral infections | rs6089946 | G | A | 0.1827 | -0.146069 | 0.030473 | 1.64E-06 |  | 0.0364 | 0.0462 | 0.4311 | 22.97659604 |
| Herpesviral infections | rs6106386 | C | T | 0.6781 | -0.117002 | 0.0251639 | 3.33E-06 |  | -0.0045 | 0.0382 | 0.9067 | 21.6187545 |
| Herpesviral infections | rs62191977 | G | T | 0.7281 | 0.185119 | 0.0355407 | 1.90E-07 |  | -0.0168 | 0.041 | 0.6811 | 27.13001517 |
| Herpesviral infections | rs62239496 | G | A | 0.2057 | -0.124096 | 0.0261293 | 2.04E-06 |  | 0.0202 | 0.0442 | 0.6482 | 22.55589248 |
| Herpesviral infections | rs62245373 | T | G | 0.3518 | 0.116167 | 0.024162 | 1.53E-06 |  | -0.054 | 0.0372 | 0.1467 | 23.11531349 |
| Herpesviral infections | rs627291 | A | G | 0.7845 | -0.130782 | 0.0281935 | 3.51E-06 |  | 0.026 | 0.0437 | 0.5526 | 21.51780474 |
| Herpesviral infections | rs66892237 | G | A | 0.07861 | -0.202468 | 0.0410236 | 8.00E-07 |  | -0.0096 | 0.0663 | 0.885 | 24.35820313 |
| Herpesviral infections | rs67241126 | A | G | 0.1789 | 0.171995 | 0.0358622 | 1.62E-06 |  | -0.0625 | 0.0468 | 0.1813 | 23.00158626 |
| Herpesviral infections | rs7093945 | C | T | 0.7741 | -0.145999 | 0.0272641 | 8.56E-08 |  | 0.0234 | 0.0426 | 0.5829 | 28.67592463 |
| Herpesviral infections | rs725076 | T | G | 0.6781 | 0.128304 | 0.0278445 | 4.07E-06 |  | -0.0369 | 0.0382 | 0.3346 | 21.23252015 |
| Herpesviral infections | rs72712509 | C | T | 0.1063 | 0.266864 | 0.0504984 | 1.26E-07 |  | -0.0151 | 0.0579 | 0.7948 | 27.92702968 |
| Herpesviral infections | rs72835696 | A | G | 0.006813 | 0.402584 | 0.0856012 | 2.56E-06 |  | -0.0417 | 0.2176 | 0.8479 | 22.11838002 |
| Herpesviral infections | rs75061358 | G | T | 0.1055 | 0.24542 | 0.0444095 | 3.27E-08 |  | -0.0729 | 0.0581 | 0.2095 | 30.53993697 |
| Herpesviral infections | rs797974 | C | T | 0.4095 | -0.112245 | 0.0243397 | 4.00E-06 |  | 0.0039 | 0.0362 | 0.9153 | 21.26686952 |
| Herpesviral infections | rs926771 | T | C | 0.07868 | -0.288211 | 0.0436426 | 4.01E-11 |  | 0.073 | 0.0664 | 0.272 | 43.6113844 |
| Herpesviral infections | rs989993 | A | C | 0.0463 | 0.380402 | 0.0812247 | 2.82E-06 |  | -0.0337 | 0.085 | 0.6917 | 21.93357133 |
| HIV diease | rs10990329 | A | C | 0.08923 | 0.186506 | 0.0372917 | 5.70E-07 |  | -0.0391 | 0.1333 | 0.769501 | 25.01273904 |
| HIV diease | rs11156763 | A | C | 0.818 | -0.194129 | 0.0418207 | 3.45E-06 |  | 0.0759 | 0.0994 | 0.4455 | 21.54756697 |
| HIV diease | rs11180712 | G | T | 0.5809 | 0.112038 | 0.0238794 | 2.71E-06 |  | -0.0318 | 0.0765 | 0.677599 | 22.01323523 |
| HIV diease | rs11576512 | A | G | 0.1714 | -0.158627 | 0.0315456 | 4.94E-07 |  | 0.0381 | 0.1008 | 0.7052 | 25.28579644 |
| HIV diease | rs11599775 | A | G | 0.3691 | -0.149069 | 0.0250404 | 2.63E-09 |  | 0.0778 | 0.0784 | 0.3211 | 35.4398726 |
| HIV diease | rs11642807 | T | C | 0.09831 | 0.2601 | 0.0466597 | 2.48E-08 |  | -0.1292 | 0.1283 | 0.3141 | 31.07397558 |
| HIV diease | rs11703757 | T | C | 0.3811 | 0.131146 | 0.0268635 | 1.05E-06 |  | -0.1444 | 0.078 | 0.0641505 | 23.83333991 |
| HIV diease | rs117248307 | T | C | 0.0148 | -0.475794 | 0.0876951 | 5.78E-08 |  | 0.0232 | 0.3101 | 0.9403 | 29.43657427 |
| HIV diease | rs117666374 | G | A | 0.09291 | 0.184402 | 0.0391675 | 2.50E-06 |  | -0.2014 | 0.1318 | 0.1264 | 22.16560297 |
| HIV diease | rs117679873 | A | G | 0.02843 | 0.279636 | 0.0605221 | 3.83E-06 |  | -0.1308 | 0.2321 | 0.573099 | 21.3480487 |
| HIV diease | rs11867655 | A | G | 0.2469 | 0.117365 | 0.0257044 | 4.97E-06 |  | 0.2121 | 0.0872 | 0.015 | 20.84789756 |
| HIV diease | rs12548531 | T | C | 0.2005 | -0.146294 | 0.0312939 | 2.94E-06 |  | 0.0466 | 0.0946 | 0.622499 | 21.85413635 |
| HIV diease | rs146122326 | A | G | 0.03015 | 0.426881 | 0.0875854 | 1.09E-06 |  | 0.0166 | 0.2246 | 0.9411 | 23.75473638 |
| HIV diease | rs146525470 | T | C | 0.1507 | -0.172127 | 0.0355047 | 1.25E-06 |  | -0.1148 | 0.1059 | 0.2784 | 23.50316255 |
| HIV diease | rs17058678 | A | G | 0.1052 | 0.177264 | 0.0343305 | 2.42E-07 |  | 0.0084 | 0.1232 | 0.9458 | 26.66126998 |
| HIV diease | rs2076438 | C | T | 0.362 | 0.114207 | 0.0248548 | 4.33E-06 |  | 0.0161 | 0.0789 | 0.838 | 21.11372698 |
| HIV diease | rs2676248 | A | G | 0.08123 | 0.293541 | 0.0618352 | 2.06E-06 |  | 0.0214 | 0.1399 | 0.8784 | 22.53543778 |
| HIV diease | rs34018810 | A | G | 0.1002 | 0.181732 | 0.0392026 | 3.56E-06 |  | -0.1297 | 0.1259 | 0.303 | 21.48983444 |
| HIV diease | rs34181263 | T | C | 0.5184 | -0.116789 | 0.023978 | 1.11E-06 |  | 0.0684 | 0.0752 | 0.363 | 23.72345661 |
| HIV diease | rs34575089 | A | G | 0.1397 | -0.186875 | 0.0324252 | 8.25E-09 |  | -0.2011 | 0.1096 | 0.0665396 | 33.21521625 |
| HIV diease | rs3731239 | G | A | 0.4272 | 0.122696 | 0.0244652 | 5.30E-07 |  | 0.0155 | 0.0764 | 0.8389 | 25.15146394 |
| HIV diease | rs4146968 | A | G | 0.858 | 0.160798 | 0.0347692 | 3.75E-06 |  | -0.1103 | 0.107 | 0.3029 | 21.38808439 |
| HIV diease | rs536256 | G | A | 0.1882 | -0.18939 | 0.032362 | 4.85E-09 |  | 0.0732 | 0.0962 | 0.4467 | 34.24864398 |
| HIV diease | rs575654 | T | G | 0.4333 | -0.145374 | 0.0255507 | 1.27E-08 |  | 0.0303 | 0.0773 | 0.6947 | 32.37187651 |
| HIV diease | rs6089946 | G | A | 0.1826 | -0.146069 | 0.030473 | 1.64E-06 |  | 0.0261 | 0.0974 | 0.7887 | 22.97659604 |
| HIV diease | rs6106386 | C | T | 0.678 | -0.117002 | 0.0251639 | 3.33E-06 |  | -0.0064 | 0.0807 | 0.9369 | 21.6187545 |
| HIV diease | rs62191977 | G | T | 0.7282 | 0.185119 | 0.0355407 | 1.90E-07 |  | -0.0139 | 0.0864 | 0.8721 | 27.13001517 |
| HIV diease | rs62239496 | G | A | 0.2056 | -0.124096 | 0.0261293 | 2.04E-06 |  | 0.1103 | 0.0939 | 0.2399 | 22.55589248 |
| HIV diease | rs62245373 | T | G | 0.3517 | 0.116167 | 0.024162 | 1.53E-06 |  | -0.061 | 0.079 | 0.4399 | 23.11531349 |
| HIV diease | rs627291 | A | G | 0.7846 | -0.130782 | 0.0281935 | 3.51E-06 |  | 0.0793 | 0.0928 | 0.3928 | 21.51780474 |
| HIV diease | rs66892237 | G | A | 0.07865 | -0.202468 | 0.0410236 | 8.00E-07 |  | 0.008 | 0.1413 | 0.9548 | 24.35820313 |
| HIV diease | rs67241126 | A | G | 0.179 | 0.171995 | 0.0358622 | 1.62E-06 |  | -0.0387 | 0.0988 | 0.6955 | 23.00158626 |
| HIV diease | rs7093945 | C | T | 0.774 | -0.145999 | 0.0272641 | 8.56E-08 |  | 0.0631 | 0.09 | 0.483 | 28.67592463 |
| HIV diease | rs725076 | T | G | 0.6781 | 0.128304 | 0.0278445 | 4.07E-06 |  | -0.0125 | 0.0808 | 0.8773 | 21.23252015 |
| HIV diease | rs72712509 | C | T | 0.1062 | 0.266864 | 0.0504984 | 1.26E-07 |  | -0.1969 | 0.1239 | 0.112 | 27.92702968 |
| HIV diease | rs72835696 | A | G | 0.006783 | 0.402584 | 0.0856012 | 2.56E-06 |  | 0.67 | 0.4596 | 0.1449 | 22.11838002 |
| HIV diease | rs75061358 | G | T | 0.1054 | 0.24542 | 0.0444095 | 3.27E-08 |  | -0.0868 | 0.1237 | 0.4831 | 30.53993697 |
| HIV diease | rs797974 | C | T | 0.4097 | -0.112245 | 0.0243397 | 4.00E-06 |  | -0.1149 | 0.0767 | 0.1339 | 21.26686952 |
| HIV diease | rs926771 | T | C | 0.07869 | -0.288211 | 0.0436426 | 4.01E-11 |  | -0.1334 | 0.1392 | 0.3378 | 43.6113844 |
| HIV diease | rs989993 | A | C | 0.04634 | 0.380402 | 0.0812247 | 2.82E-06 |  | 0.2712 | 0.1838 | 0.1401 | 21.93357133 |
| HPV | rs12548531 | T | C | 0.2006 | -0.146294 | 0.0312939 | 2.94E-06 |  | -0.05661 | 0.0599936 | 0.3456 | 21.85413635 |
| HPV | rs3731239 | G | A | 0.4275 | 0.122696 | 0.0244652 | 5.30E-07 |  | -0.1143 | 0.045996 | 0.0131199 | 25.15146394 |
| HPV | rs6106386 | C | T | 0.678 | -0.117002 | 0.0251639 | 3.33E-06 |  | -0.02276 | 0.0489042 | 0.6418 | 21.6187545 |
| HPV | rs725076 | T | G | 0.6781 | 0.128304 | 0.0278445 | 4.07E-06 |  | -0.07479 | 0.0502621 | 0.1372 | 21.23252015 |
| measles | rs10990329 | A | C | 0.08923 | 0.186506 | 0.0372917 | 5.70E-07 |  | 0.0378 | 0.1853 | 0.8384 | 25.01273904 |
| measles | rs11156763 | A | C | 0.818 | -0.194129 | 0.0418207 | 3.45E-06 |  | 0.2845 | 0.1399 | 0.0420097 | 21.54756697 |
| measles | rs11180712 | G | T | 0.5809 | 0.112038 | 0.0238794 | 2.71E-06 |  | 0.0394 | 0.1069 | 0.7126 | 22.01323523 |
| measles | rs11576512 | A | G | 0.1715 | -0.158627 | 0.0315456 | 4.94E-07 |  | -0.1344 | 0.1432 | 0.3479 | 25.28579644 |
| measles | rs11599775 | A | G | 0.3692 | -0.149069 | 0.0250404 | 2.63E-09 |  | -0.1936 | 0.1097 | 0.0775497 | 35.4398726 |
| measles | rs11642807 | T | C | 0.09833 | 0.2601 | 0.0466597 | 2.48E-08 |  | 0.1105 | 0.1802 | 0.539699 | 31.07397558 |
| measles | rs11703757 | T | C | 0.3811 | 0.131146 | 0.0268635 | 1.05E-06 |  | 0.0111 | 0.1092 | 0.9187 | 23.83333991 |
| measles | rs117248307 | T | C | 0.01485 | -0.475794 | 0.0876951 | 5.78E-08 |  | 0.5179 | 0.4402 | 0.2394 | 29.43657427 |
| measles | rs117666374 | G | A | 0.093 | 0.184402 | 0.0391675 | 2.50E-06 |  | -0.2273 | 0.1815 | 0.2106 | 22.16560297 |
| measles | rs117679873 | A | G | 0.02844 | 0.279636 | 0.0605221 | 3.83E-06 |  | -0.0196 | 0.3191 | 0.951 | 21.3480487 |
| measles | rs11867655 | A | G | 0.2467 | 0.117365 | 0.0257044 | 4.97E-06 |  | -0.0831 | 0.1225 | 0.4974 | 20.84789756 |
| measles | rs12548531 | T | C | 0.2005 | -0.146294 | 0.0312939 | 2.94E-06 |  | -0.1371 | 0.1324 | 0.3003 | 21.85413635 |
| measles | rs146122326 | A | G | 0.03015 | 0.426881 | 0.0875854 | 1.09E-06 |  | -0.1734 | 0.3125 | 0.579001 | 23.75473638 |
| measles | rs146525470 | T | C | 0.1507 | -0.172127 | 0.0355047 | 1.25E-06 |  | 0.0587 | 0.1482 | 0.6921 | 23.50316255 |
| measles | rs17058678 | A | G | 0.1054 | 0.177264 | 0.0343305 | 2.42E-07 |  | 0.0691 | 0.173 | 0.6894 | 26.66126998 |
| measles | rs2076438 | C | T | 0.3619 | 0.114207 | 0.0248548 | 4.33E-06 |  | -0.0583 | 0.1099 | 0.5961 | 21.11372698 |
| measles | rs2676248 | A | G | 0.08127 | 0.293541 | 0.0618352 | 2.06E-06 |  | -0.0588 | 0.1959 | 0.7641 | 22.53543778 |
| measles | rs34018810 | A | G | 0.1002 | 0.181732 | 0.0392026 | 3.56E-06 |  | -0.2528 | 0.1756 | 0.15 | 21.48983444 |
| measles | rs34181263 | T | C | 0.5186 | -0.116789 | 0.023978 | 1.11E-06 |  | -0.0534 | 0.1054 | 0.6126 | 23.72345661 |
| measles | rs34575089 | A | G | 0.1397 | -0.186875 | 0.0324252 | 8.25E-09 |  | -0.348 | 0.1517 | 0.02175 | 33.21521625 |
| measles | rs3731239 | G | A | 0.4272 | 0.122696 | 0.0244652 | 5.30E-07 |  | 0.0319 | 0.1071 | 0.7655 | 25.15146394 |
| measles | rs4146968 | A | G | 0.8581 | 0.160798 | 0.0347692 | 3.75E-06 |  | 0.099 | 0.1514 | 0.5132 | 21.38808439 |
| measles | rs536256 | G | A | 0.1882 | -0.18939 | 0.032362 | 4.85E-09 |  | 0.4163 | 0.1361 | 0.002223 | 34.24864398 |
| measles | rs575654 | T | G | 0.4334 | -0.145374 | 0.0255507 | 1.27E-08 |  | -0.0836 | 0.1084 | 0.4407 | 32.37187651 |
| measles | rs6089946 | G | A | 0.1827 | -0.146069 | 0.030473 | 1.64E-06 |  | 0.2495 | 0.1372 | 0.0690701 | 22.97659604 |
| measles | rs6106386 | C | T | 0.6781 | -0.117002 | 0.0251639 | 3.33E-06 |  | -0.0556 | 0.1132 | 0.6233 | 21.6187545 |
| measles | rs62191977 | G | T | 0.7281 | 0.185119 | 0.0355407 | 1.90E-07 |  | 0.0482 | 0.1213 | 0.691 | 27.13001517 |
| measles | rs62239496 | G | A | 0.2056 | -0.124096 | 0.0261293 | 2.04E-06 |  | 0.084 | 0.1309 | 0.521 | 22.55589248 |
| measles | rs62245373 | T | G | 0.3519 | 0.116167 | 0.024162 | 1.53E-06 |  | 0.1128 | 0.1107 | 0.3079 | 23.11531349 |
| measles | rs627291 | A | G | 0.7845 | -0.130782 | 0.0281935 | 3.51E-06 |  | 0.2129 | 0.1295 | 0.1002 | 21.51780474 |
| measles | rs66892237 | G | A | 0.07862 | -0.202468 | 0.0410236 | 8.00E-07 |  | -0.0081 | 0.1964 | 0.9671 | 24.35820313 |
| measles | rs67241126 | A | G | 0.1789 | 0.171995 | 0.0358622 | 1.62E-06 |  | 0.093 | 0.1385 | 0.502 | 23.00158626 |
| measles | rs7093945 | C | T | 0.774 | -0.145999 | 0.0272641 | 8.56E-08 |  | -0.0041 | 0.1265 | 0.9745 | 28.67592463 |
| measles | rs725076 | T | G | 0.6782 | 0.128304 | 0.0278445 | 4.07E-06 |  | 0.09 | 0.1131 | 0.4262 | 21.23252015 |
| measles | rs72712509 | C | T | 0.1063 | 0.266864 | 0.0504984 | 1.26E-07 |  | 0.0698 | 0.1717 | 0.6842 | 27.92702968 |
| measles | rs72835696 | A | G | 0.006814 | 0.402584 | 0.0856012 | 2.56E-06 |  | -0.1831 | 0.65 | 0.7782 | 22.11838002 |
| measles | rs75061358 | G | T | 0.1055 | 0.24542 | 0.0444095 | 3.27E-08 |  | 0.0198 | 0.1726 | 0.9086 | 30.53993697 |
| measles | rs797974 | C | T | 0.4095 | -0.112245 | 0.0243397 | 4.00E-06 |  | -0.0881 | 0.1074 | 0.412 | 21.26686952 |
| measles | rs926771 | T | C | 0.07865 | -0.288211 | 0.0436426 | 4.01E-11 |  | 0.0852 | 0.1963 | 0.6642 | 43.6113844 |
| measles | rs989993 | A | C | 0.04631 | 0.380402 | 0.0812247 | 2.82E-06 |  | 0.0852 | 0.2523 | 0.7356 | 21.93357133 |
| mumps | rs10990329 | A | C | 0.08926 | 0.186506 | 0.0372917 | 5.70E-07 |  | 0.1491 | 0.1192 | 0.2109 | 25.01273904 |
| mumps | rs11156763 | A | C | 0.818 | -0.194129 | 0.0418207 | 3.45E-06 |  | -0.0191 | 0.0899 | 0.8319 | 21.54756697 |
| mumps | rs11180712 | G | T | 0.5808 | 0.112038 | 0.0238794 | 2.71E-06 |  | -0.0597 | 0.0688 | 0.3851 | 22.01323523 |
| mumps | rs11576512 | A | G | 0.1714 | -0.158627 | 0.0315456 | 4.94E-07 |  | 0.039 | 0.0921 | 0.672 | 25.28579644 |
| mumps | rs11599775 | A | G | 0.3693 | -0.149069 | 0.0250404 | 2.63E-09 |  | 0.0816 | 0.0704 | 0.2468 | 35.4398726 |
| mumps | rs11642807 | T | C | 0.09836 | 0.2601 | 0.0466597 | 2.48E-08 |  | 0.147 | 0.1155 | 0.2032 | 31.07397558 |
| mumps | rs11703757 | T | C | 0.3811 | 0.131146 | 0.0268635 | 1.05E-06 |  | -0.0451 | 0.0701 | 0.5197 | 23.83333991 |
| mumps | rs117248307 | T | C | 0.01478 | -0.475794 | 0.0876951 | 5.78E-08 |  | 4.00E-04 | 0.2846 | 0.9988 | 29.43657427 |
| mumps | rs117666374 | G | A | 0.09296 | 0.184402 | 0.0391675 | 2.50E-06 |  | 0.1536 | 0.1167 | 0.1879 | 22.16560297 |
| mumps | rs117679873 | A | G | 0.02839 | 0.279636 | 0.0605221 | 3.83E-06 |  | 0.1173 | 0.2049 | 0.567 | 21.3480487 |
| mumps | rs11867655 | A | G | 0.2469 | 0.117365 | 0.0257044 | 4.97E-06 |  | 0.02 | 0.0787 | 0.7993 | 20.84789756 |
| mumps | rs12548531 | T | C | 0.2006 | -0.146294 | 0.0312939 | 2.94E-06 |  | -0.0814 | 0.0852 | 0.3392 | 21.85413635 |
| mumps | rs146122326 | A | G | 0.03015 | 0.426881 | 0.0875854 | 1.09E-06 |  | -0.0131 | 0.2015 | 0.9483 | 23.75473638 |
| mumps | rs146525470 | T | C | 0.1507 | -0.172127 | 0.0355047 | 1.25E-06 |  | 0.0358 | 0.0953 | 0.706999 | 23.50316255 |
| mumps | rs17058678 | A | G | 0.1053 | 0.177264 | 0.0343305 | 2.42E-07 |  | 0.1616 | 0.1113 | 0.1464 | 26.66126998 |
| mumps | rs2076438 | C | T | 0.3619 | 0.114207 | 0.0248548 | 4.33E-06 |  | -0.135 | 0.0705 | 0.0555405 | 21.11372698 |
| mumps | rs2676248 | A | G | 0.08125 | 0.293541 | 0.0618352 | 2.06E-06 |  | -0.1614 | 0.1261 | 0.2004 | 22.53543778 |
| mumps | rs34018810 | A | G | 0.1003 | 0.181732 | 0.0392026 | 3.56E-06 |  | 0.1222 | 0.1131 | 0.2801 | 21.48983444 |
| mumps | rs34181263 | T | C | 0.5186 | -0.116789 | 0.023978 | 1.11E-06 |  | -0.0211 | 0.0677 | 0.755299 | 23.72345661 |
| mumps | rs34575089 | A | G | 0.1398 | -0.186875 | 0.0324252 | 8.25E-09 |  | 0.1149 | 0.0976 | 0.2391 | 33.21521625 |
| mumps | rs3731239 | G | A | 0.4275 | 0.122696 | 0.0244652 | 5.30E-07 |  | 0.0886 | 0.0688 | 0.1979 | 25.15146394 |
| mumps | rs4146968 | A | G | 0.8581 | 0.160798 | 0.0347692 | 3.75E-06 |  | -0.09 | 0.0973 | 0.3552 | 21.38808439 |
| mumps | rs536256 | G | A | 0.1882 | -0.18939 | 0.032362 | 4.85E-09 |  | -0.0211 | 0.087 | 0.8082 | 34.24864398 |
| mumps | rs575654 | T | G | 0.4334 | -0.145374 | 0.0255507 | 1.27E-08 |  | -0.0464 | 0.0696 | 0.5055 | 32.37187651 |
| mumps | rs6089946 | G | A | 0.1825 | -0.146069 | 0.030473 | 1.64E-06 |  | -0.0227 | 0.0883 | 0.7975 | 22.97659604 |
| mumps | rs6106386 | C | T | 0.678 | -0.117002 | 0.0251639 | 3.33E-06 |  | 0.0719 | 0.0727 | 0.3223 | 21.6187545 |
| mumps | rs62191977 | G | T | 0.7282 | 0.185119 | 0.0355407 | 1.90E-07 |  | 0.0413 | 0.078 | 0.5961 | 27.13001517 |
| mumps | rs62239496 | G | A | 0.2055 | -0.124096 | 0.0261293 | 2.04E-06 |  | 0.001 | 0.0842 | 0.9903 | 22.55589248 |
| mumps | rs62245373 | T | G | 0.3516 | 0.116167 | 0.024162 | 1.53E-06 |  | 0.0217 | 0.0711 | 0.759899 | 23.11531349 |
| mumps | rs627291 | A | G | 0.7846 | -0.130782 | 0.0281935 | 3.51E-06 |  | 0.0368 | 0.0834 | 0.658801 | 21.51780474 |
| mumps | rs66892237 | G | A | 0.07869 | -0.202468 | 0.0410236 | 8.00E-07 |  | -0.1066 | 0.1262 | 0.3983 | 24.35820313 |
| mumps | rs67241126 | A | G | 0.1789 | 0.171995 | 0.0358622 | 1.62E-06 |  | -0.0827 | 0.089 | 0.3529 | 23.00158626 |
| mumps | rs7093945 | C | T | 0.7739 | -0.145999 | 0.0272641 | 8.56E-08 |  | -0.0222 | 0.0813 | 0.7843 | 28.67592463 |
| mumps | rs725076 | T | G | 0.6781 | 0.128304 | 0.0278445 | 4.07E-06 |  | 0.1149 | 0.0727 | 0.1142 | 21.23252015 |
| mumps | rs72712509 | C | T | 0.1062 | 0.266864 | 0.0504984 | 1.26E-07 |  | 0.0638 | 0.1106 | 0.564 | 27.92702968 |
| mumps | rs72835696 | A | G | 0.006789 | 0.402584 | 0.0856012 | 2.56E-06 |  | -0.6749 | 0.4192 | 0.1074 | 22.11838002 |
| mumps | rs75061358 | G | T | 0.1055 | 0.24542 | 0.0444095 | 3.27E-08 |  | 0.0313 | 0.1108 | 0.7777 | 30.53993697 |
| mumps | rs797974 | C | T | 0.4097 | -0.112245 | 0.0243397 | 4.00E-06 |  | -0.0954 | 0.069 | 0.167 | 21.26686952 |
| mumps | rs926771 | T | C | 0.07874 | -0.288211 | 0.0436426 | 4.01E-11 |  | 0.0471 | 0.1257 | 0.707499 | 43.6113844 |
| mumps | rs989993 | A | C | 0.04637 | 0.380402 | 0.0812247 | 2.82E-06 |  | -0.0641 | 0.1626 | 0.693201 | 21.93357133 |
| poliovirus | rs10990329 | A | C | 0.08925 | 0.186506 | 0.0372917 | 5.70E-07 |  | -0.2638 | 0.1645 | 0.1088 | 25.01273904 |
| poliovirus | rs11156763 | A | C | 0.818 | -0.194129 | 0.0418207 | 3.45E-06 |  | -0.1191 | 0.1241 | 0.337 | 21.54756697 |
| poliovirus | rs11180712 | G | T | 0.5809 | 0.112038 | 0.0238794 | 2.71E-06 |  | 0.1308 | 0.0948 | 0.1674 | 22.01323523 |
| poliovirus | rs11576512 | A | G | 0.1714 | -0.158627 | 0.0315456 | 4.94E-07 |  | -0.0175 | 0.1271 | 0.8907 | 25.28579644 |
| poliovirus | rs11599775 | A | G | 0.3691 | -0.149069 | 0.0250404 | 2.63E-09 |  | -0.0132 | 0.0972 | 0.8918 | 35.4398726 |
| poliovirus | rs11642807 | T | C | 0.09833 | 0.2601 | 0.0466597 | 2.48E-08 |  | -0.2225 | 0.1604 | 0.1655 | 31.07397558 |
| poliovirus | rs11703757 | T | C | 0.3811 | 0.131146 | 0.0268635 | 1.05E-06 |  | -0.0391 | 0.0966 | 0.685499 | 23.83333991 |
| poliovirus | rs117248307 | T | C | 0.01481 | -0.475794 | 0.0876951 | 5.78E-08 |  | 0.7318 | 0.3882 | 0.0593895 | 29.43657427 |
| poliovirus | rs117666374 | G | A | 0.0929 | 0.184402 | 0.0391675 | 2.50E-06 |  | 0.128 | 0.1627 | 0.4313 | 22.16560297 |
| poliovirus | rs117679873 | A | G | 0.02845 | 0.279636 | 0.0605221 | 3.83E-06 |  | 0.1243 | 0.2882 | 0.6663 | 21.3480487 |
| poliovirus | rs11867655 | A | G | 0.2468 | 0.117365 | 0.0257044 | 4.97E-06 |  | 0.2146 | 0.1084 | 0.0476497 | 20.84789756 |
| poliovirus | rs12548531 | T | C | 0.2005 | -0.146294 | 0.0312939 | 2.94E-06 |  | 0.0272 | 0.1172 | 0.8167 | 21.85413635 |
| poliovirus | rs146122326 | A | G | 0.03015 | 0.426881 | 0.0875854 | 1.09E-06 |  | -0.2041 | 0.2804 | 0.4666 | 23.75473638 |
| poliovirus | rs146525470 | T | C | 0.1507 | -0.172127 | 0.0355047 | 1.25E-06 |  | -0.2514 | 0.1309 | 0.0547999 | 23.50316255 |
| poliovirus | rs17058678 | A | G | 0.1052 | 0.177264 | 0.0343305 | 2.42E-07 |  | 0.3377 | 0.1539 | 0.0281702 | 26.66126998 |
| poliovirus | rs2076438 | C | T | 0.362 | 0.114207 | 0.0248548 | 4.33E-06 |  | 0.1154 | 0.0972 | 0.2352 | 21.11372698 |
| poliovirus | rs2676248 | A | G | 0.08125 | 0.293541 | 0.0618352 | 2.06E-06 |  | 0.0934 | 0.1719 | 0.5869 | 22.53543778 |
| poliovirus | rs34018810 | A | G | 0.1002 | 0.181732 | 0.0392026 | 3.56E-06 |  | -0.0595 | 0.1551 | 0.7013 | 21.48983444 |
| poliovirus | rs34181263 | T | C | 0.5184 | -0.116789 | 0.023978 | 1.11E-06 |  | 0.0827 | 0.0934 | 0.3756 | 23.72345661 |
| poliovirus | rs34575089 | A | G | 0.1397 | -0.186875 | 0.0324252 | 8.25E-09 |  | -0.0258 | 0.134 | 0.8476 | 33.21521625 |
| poliovirus | rs3731239 | G | A | 0.4272 | 0.122696 | 0.0244652 | 5.30E-07 |  | -0.0643 | 0.0948 | 0.4975 | 25.15146394 |
| poliovirus | rs4146968 | A | G | 0.858 | 0.160798 | 0.0347692 | 3.75E-06 |  | 0.0978 | 0.1337 | 0.4645 | 21.38808439 |
| poliovirus | rs536256 | G | A | 0.1882 | -0.18939 | 0.032362 | 4.85E-09 |  | 0.0555 | 0.1195 | 0.6421 | 34.24864398 |
| poliovirus | rs575654 | T | G | 0.4333 | -0.145374 | 0.0255507 | 1.27E-08 |  | 0.0032 | 0.096 | 0.9737 | 32.37187651 |
| poliovirus | rs6089946 | G | A | 0.1826 | -0.146069 | 0.030473 | 1.64E-06 |  | -0.0381 | 0.1213 | 0.753401 | 22.97659604 |
| poliovirus | rs6106386 | C | T | 0.678 | -0.117002 | 0.0251639 | 3.33E-06 |  | -0.1135 | 0.1002 | 0.2571 | 21.6187545 |
| poliovirus | rs62191977 | G | T | 0.7281 | 0.185119 | 0.0355407 | 1.90E-07 |  | 0.0535 | 0.1079 | 0.6204 | 27.13001517 |
| poliovirus | rs62239496 | G | A | 0.2056 | -0.124096 | 0.0261293 | 2.04E-06 |  | -0.1882 | 0.1163 | 0.1055 | 22.55589248 |
| poliovirus | rs62245373 | T | G | 0.3517 | 0.116167 | 0.024162 | 1.53E-06 |  | -0.0957 | 0.0979 | 0.3282 | 23.11531349 |
| poliovirus | rs627291 | A | G | 0.7846 | -0.130782 | 0.0281935 | 3.51E-06 |  | -0.057 | 0.1151 | 0.6205 | 21.51780474 |
| poliovirus | rs66892237 | G | A | 0.07863 | -0.202468 | 0.0410236 | 8.00E-07 |  | -0.1261 | 0.1743 | 0.4695 | 24.35820313 |
| poliovirus | rs67241126 | A | G | 0.179 | 0.171995 | 0.0358622 | 1.62E-06 |  | -0.0489 | 0.1224 | 0.6898 | 23.00158626 |
| poliovirus | rs7093945 | C | T | 0.774 | -0.145999 | 0.0272641 | 8.56E-08 |  | -0.1298 | 0.1121 | 0.2467 | 28.67592463 |
| poliovirus | rs725076 | T | G | 0.6781 | 0.128304 | 0.0278445 | 4.07E-06 |  | -0.0437 | 0.1005 | 0.6633 | 21.23252015 |
| poliovirus | rs72712509 | C | T | 0.1063 | 0.266864 | 0.0504984 | 1.26E-07 |  | 0.1378 | 0.1523 | 0.3657 | 27.92702968 |
| poliovirus | rs72835696 | A | G | 0.006782 | 0.402584 | 0.0856012 | 2.56E-06 |  | 0.9749 | 0.5786 | 0.0920111 | 22.11838002 |
| poliovirus | rs75061358 | G | T | 0.1054 | 0.24542 | 0.0444095 | 3.27E-08 |  | 0.0018 | 0.153 | 0.9907 | 30.53993697 |
| poliovirus | rs797974 | C | T | 0.4097 | -0.112245 | 0.0243397 | 4.00E-06 |  | 0.1186 | 0.095 | 0.2118 | 21.26686952 |
| poliovirus | rs926771 | T | C | 0.0787 | -0.288211 | 0.0436426 | 4.01E-11 |  | 0.2336 | 0.1737 | 0.1786 | 43.6113844 |
| poliovirus | rs989993 | A | C | 0.04633 | 0.380402 | 0.0812247 | 2.82E-06 |  | 0.2365 | 0.2237 | 0.2905 | 21.93357133 |
| Rubella | rs10990329 | A | C | 0.08926 | 0.186506 | 0.0372917 | 5.70E-07 |  | 0.1716 | 0.1073 | 0.1098 | 25.01273904 |
| Rubella | rs11156763 | A | C | 0.818 | -0.194129 | 0.0418207 | 3.45E-06 |  | -0.0224 | 0.0801 | 0.7798 | 21.54756697 |
| Rubella | rs11180712 | G | T | 0.5808 | 0.112038 | 0.0238794 | 2.71E-06 |  | -0.0673 | 0.0618 | 0.2765 | 22.01323523 |
| Rubella | rs11576512 | A | G | 0.1715 | -0.158627 | 0.0315456 | 4.94E-07 |  | 0.0183 | 0.0829 | 0.8254 | 25.28579644 |
| Rubella | rs11599775 | A | G | 0.3693 | -0.149069 | 0.0250404 | 2.63E-09 |  | 0.0917 | 0.0633 | 0.1477 | 35.4398726 |
| Rubella | rs11642807 | T | C | 0.09831 | 0.2601 | 0.0466597 | 2.48E-08 |  | -0.0767 | 0.1033 | 0.457999 | 31.07397558 |
| Rubella | rs11703757 | T | C | 0.3811 | 0.131146 | 0.0268635 | 1.05E-06 |  | -0.0034 | 0.0629 | 0.9572 | 23.83333991 |
| Rubella | rs117248307 | T | C | 0.01482 | -0.475794 | 0.0876951 | 5.78E-08 |  | -0.3962 | 0.2561 | 0.1219 | 29.43657427 |
| Rubella | rs117666374 | G | A | 0.09303 | 0.184402 | 0.0391675 | 2.50E-06 |  | 0.0514 | 0.1052 | 0.625299 | 22.16560297 |
| Rubella | rs117679873 | A | G | 0.02843 | 0.279636 | 0.0605221 | 3.83E-06 |  | -0.1681 | 0.1828 | 0.3577 | 21.3480487 |
| Rubella | rs11867655 | A | G | 0.2467 | 0.117365 | 0.0257044 | 4.97E-06 |  | 0.0263 | 0.0706 | 0.7092 | 20.84789756 |
| Rubella | rs12548531 | T | C | 0.2005 | -0.146294 | 0.0312939 | 2.94E-06 |  | -0.0697 | 0.0761 | 0.3602 | 21.85413635 |
| Rubella | rs146122326 | A | G | 0.03015 | 0.426881 | 0.0875854 | 1.09E-06 |  | -0.0384 | 0.1819 | 0.833 | 23.75473638 |
| Rubella | rs146525470 | T | C | 0.1507 | -0.172127 | 0.0355047 | 1.25E-06 |  | 0.0123 | 0.0853 | 0.8852 | 23.50316255 |
| Rubella | rs17058678 | A | G | 0.1053 | 0.177264 | 0.0343305 | 2.42E-07 |  | -0.0986 | 0.1 | 0.3239 | 26.66126998 |
| Rubella | rs2076438 | C | T | 0.3619 | 0.114207 | 0.0248548 | 4.33E-06 |  | -4.00E-04 | 0.0633 | 0.9948 | 21.11372698 |
| Rubella | rs2676248 | A | G | 0.08125 | 0.293541 | 0.0618352 | 2.06E-06 |  | -0.1299 | 0.1129 | 0.2498 | 22.53543778 |
| Rubella | rs34018810 | A | G | 0.1001 | 0.181732 | 0.0392026 | 3.56E-06 |  | -0.1872 | 0.1016 | 0.0654696 | 21.48983444 |
| Rubella | rs34181263 | T | C | 0.5185 | -0.116789 | 0.023978 | 1.11E-06 |  | -0.075 | 0.0606 | 0.2158 | 23.72345661 |
| Rubella | rs34575089 | A | G | 0.1398 | -0.186875 | 0.0324252 | 8.25E-09 |  | 0.0077 | 0.0875 | 0.93 | 33.21521625 |
| Rubella | rs3731239 | G | A | 0.4272 | 0.122696 | 0.0244652 | 5.30E-07 |  | 0.0831 | 0.0617 | 0.1784 | 25.15146394 |
| Rubella | rs4146968 | A | G | 0.8581 | 0.160798 | 0.0347692 | 3.75E-06 |  | 0.0257 | 0.0871 | 0.7674 | 21.38808439 |
| Rubella | rs536256 | G | A | 0.1882 | -0.18939 | 0.032362 | 4.85E-09 |  | 0.1508 | 0.0776 | 0.05199 | 34.24864398 |
| Rubella | rs575654 | T | G | 0.4335 | -0.145374 | 0.0255507 | 1.27E-08 |  | 0.0557 | 0.0623 | 0.372 | 32.37187651 |
| Rubella | rs6089946 | G | A | 0.1827 | -0.146069 | 0.030473 | 1.64E-06 |  | 0.0026 | 0.0791 | 0.9736 | 22.97659604 |
| Rubella | rs6106386 | C | T | 0.6781 | -0.117002 | 0.0251639 | 3.33E-06 |  | 0.0338 | 0.065 | 0.6035 | 21.6187545 |
| Rubella | rs62191977 | G | T | 0.7281 | 0.185119 | 0.0355407 | 1.90E-07 |  | -0.015 | 0.07 | 0.8301 | 27.13001517 |
| Rubella | rs62239496 | G | A | 0.2056 | -0.124096 | 0.0261293 | 2.04E-06 |  | -0.0205 | 0.0758 | 0.7866 | 22.55589248 |
| Rubella | rs62245373 | T | G | 0.3519 | 0.116167 | 0.024162 | 1.53E-06 |  | -0.0492 | 0.0637 | 0.4404 | 23.11531349 |
| Rubella | rs627291 | A | G | 0.7846 | -0.130782 | 0.0281935 | 3.51E-06 |  | 0.1494 | 0.0751 | 0.0468296 | 21.51780474 |
| Rubella | rs66892237 | G | A | 0.07866 | -0.202468 | 0.0410236 | 8.00E-07 |  | 0.2675 | 0.1135 | 0.0184901 | 24.35820313 |
| Rubella | rs67241126 | A | G | 0.1789 | 0.171995 | 0.0358622 | 1.62E-06 |  | 0.02 | 0.0796 | 0.8017 | 23.00158626 |
| Rubella | rs7093945 | C | T | 0.774 | -0.145999 | 0.0272641 | 8.56E-08 |  | -0.102 | 0.0727 | 0.161 | 28.67592463 |
| Rubella | rs725076 | T | G | 0.6782 | 0.128304 | 0.0278445 | 4.07E-06 |  | 0.0274 | 0.0654 | 0.675601 | 21.23252015 |
| Rubella | rs72712509 | C | T | 0.1063 | 0.266864 | 0.0504984 | 1.26E-07 |  | -0.1431 | 0.0999 | 0.1523 | 27.92702968 |
| Rubella | rs72835696 | A | G | 0.006807 | 0.402584 | 0.0856012 | 2.56E-06 |  | -0.5027 | 0.373 | 0.1777 | 22.11838002 |
| Rubella | rs75061358 | G | T | 0.1055 | 0.24542 | 0.0444095 | 3.27E-08 |  | 0.013 | 0.0992 | 0.8955 | 30.53993697 |
| Rubella | rs797974 | C | T | 0.4095 | -0.112245 | 0.0243397 | 4.00E-06 |  | 0.0234 | 0.0619 | 0.706 | 21.26686952 |
| Rubella | rs926771 | T | C | 0.07862 | -0.288211 | 0.0436426 | 4.01E-11 |  | -0.1161 | 0.1141 | 0.3089 | 43.6113844 |
| Rubella | rs989993 | A | C | 0.0463 | 0.380402 | 0.0812247 | 2.82E-06 |  | -0.0821 | 0.1462 | 0.5743 | 21.93357133 |
| Herpes zoster | rs10990329 | A | C | 0.08927 | 0.186506 | 0.0372917 | 5.70E-07 |  | 0.0558 | 0.055 | 0.3097 | 25.01273904 |
| Herpes zoster | rs11156763 | A | C | 0.818 | -0.194129 | 0.0418207 | 3.45E-06 |  | -0.0365 | 0.0415 | 0.3801 | 21.54756697 |
| Herpes zoster | rs11180712 | G | T | 0.5808 | 0.112038 | 0.0238794 | 2.71E-06 |  | -0.0065 | 0.0318 | 0.8376 | 22.01323523 |
| Herpes zoster | rs11576512 | A | G | 0.1715 | -0.158627 | 0.0315456 | 4.94E-07 |  | -0.031 | 0.0426 | 0.4662 | 25.28579644 |
| Herpes zoster | rs11599775 | A | G | 0.3691 | -0.149069 | 0.0250404 | 2.63E-09 |  | -0.0775 | 0.0325 | 0.01725 | 35.4398726 |
| Herpes zoster | rs11642807 | T | C | 0.09836 | 0.2601 | 0.0466597 | 2.48E-08 |  | 0.0452 | 0.0535 | 0.3984 | 31.07397558 |
| Herpes zoster | rs11703757 | T | C | 0.3811 | 0.131146 | 0.0268635 | 1.05E-06 |  | 0.0251 | 0.0324 | 0.4393 | 23.83333991 |
| Herpes zoster | rs117248307 | T | C | 0.0148 | -0.475794 | 0.0876951 | 5.78E-08 |  | -0.3195 | 0.1304 | 0.0143001 | 29.43657427 |
| Herpes zoster | rs117666374 | G | A | 0.09302 | 0.184402 | 0.0391675 | 2.50E-06 |  | 0.007 | 0.0544 | 0.8981 | 22.16560297 |
| Herpes zoster | rs117679873 | A | G | 0.02841 | 0.279636 | 0.0605221 | 3.83E-06 |  | -0.0985 | 0.0947 | 0.2984 | 21.3480487 |
| Herpes zoster | rs11867655 | A | G | 0.2468 | 0.117365 | 0.0257044 | 4.97E-06 |  | 0.0158 | 0.0364 | 0.664401 | 20.84789756 |
| Herpes zoster | rs12548531 | T | C | 0.2005 | -0.146294 | 0.0312939 | 2.94E-06 |  | -0.0231 | 0.0393 | 0.5567 | 21.85413635 |
| Herpes zoster | rs146122326 | A | G | 0.03017 | 0.426881 | 0.0875854 | 1.09E-06 |  | 0.0874 | 0.0938 | 0.3513 | 23.75473638 |
| Herpes zoster | rs146525470 | T | C | 0.1506 | -0.172127 | 0.0355047 | 1.25E-06 |  | -0.006 | 0.0439 | 0.8911 | 23.50316255 |
| Herpes zoster | rs17058678 | A | G | 0.1053 | 0.177264 | 0.0343305 | 2.42E-07 |  | -0.0367 | 0.0513 | 0.4742 | 26.66126998 |
| Herpes zoster | rs2076438 | C | T | 0.3619 | 0.114207 | 0.0248548 | 4.33E-06 |  | 0.019 | 0.0326 | 0.5593 | 21.11372698 |
| Herpes zoster | rs2676248 | A | G | 0.08127 | 0.293541 | 0.0618352 | 2.06E-06 |  | -0.0113 | 0.0582 | 0.8459 | 22.53543778 |
| Herpes zoster | rs34018810 | A | G | 0.1003 | 0.181732 | 0.0392026 | 3.56E-06 |  | 0.1413 | 0.0521 | 0.00664293 | 21.48983444 |
| Herpes zoster | rs34181263 | T | C | 0.5185 | -0.116789 | 0.023978 | 1.11E-06 |  | -0.0324 | 0.0313 | 0.3007 | 23.72345661 |
| Herpes zoster | rs34575089 | A | G | 0.1397 | -0.186875 | 0.0324252 | 8.25E-09 |  | -0.0628 | 0.0449 | 0.1613 | 33.21521625 |
| Herpes zoster | rs3731239 | G | A | 0.4272 | 0.122696 | 0.0244652 | 5.30E-07 |  | 0.0043 | 0.0317 | 0.8932 | 25.15146394 |
| Herpes zoster | rs4146968 | A | G | 0.8581 | 0.160798 | 0.0347692 | 3.75E-06 |  | 0.018 | 0.0448 | 0.687999 | 21.38808439 |
| Herpes zoster | rs536256 | G | A | 0.1882 | -0.18939 | 0.032362 | 4.85E-09 |  | -0.0074 | 0.0402 | 0.8539 | 34.24864398 |
| Herpes zoster | rs575654 | T | G | 0.4334 | -0.145374 | 0.0255507 | 1.27E-08 |  | -0.0158 | 0.0322 | 0.6243 | 32.37187651 |
| Herpes zoster | rs6089946 | G | A | 0.1827 | -0.146069 | 0.030473 | 1.64E-06 |  | -6.00E-04 | 0.0408 | 0.9889 | 22.97659604 |
| Herpes zoster | rs6106386 | C | T | 0.6781 | -0.117002 | 0.0251639 | 3.33E-06 |  | -0.0335 | 0.0336 | 0.3179 | 21.6187545 |
| Herpes zoster | rs62191977 | G | T | 0.7281 | 0.185119 | 0.0355407 | 1.90E-07 |  | 0.0197 | 0.0361 | 0.585901 | 27.13001517 |
| Herpes zoster | rs62239496 | G | A | 0.2056 | -0.124096 | 0.0261293 | 2.04E-06 |  | -0.0206 | 0.0389 | 0.5968 | 22.55589248 |
| Herpes zoster | rs62245373 | T | G | 0.3518 | 0.116167 | 0.024162 | 1.53E-06 |  | -0.0256 | 0.0328 | 0.4347 | 23.11531349 |
| Herpes zoster | rs627291 | A | G | 0.7846 | -0.130782 | 0.0281935 | 3.51E-06 |  | 0.0382 | 0.0385 | 0.3212 | 21.51780474 |
| Herpes zoster | rs66892237 | G | A | 0.07859 | -0.202468 | 0.0410236 | 8.00E-07 |  | -0.0426 | 0.0583 | 0.465 | 24.35820313 |
| Herpes zoster | rs67241126 | A | G | 0.179 | 0.171995 | 0.0358622 | 1.62E-06 |  | 0.0645 | 0.0411 | 0.1168 | 23.00158626 |
| Herpes zoster | rs7093945 | C | T | 0.774 | -0.145999 | 0.0272641 | 8.56E-08 |  | -0.0013 | 0.0375 | 0.9734 | 28.67592463 |
| Herpes zoster | rs725076 | T | G | 0.6781 | 0.128304 | 0.0278445 | 4.07E-06 |  | -0.0233 | 0.0337 | 0.489199 | 21.23252015 |
| Herpes zoster | rs72712509 | C | T | 0.1063 | 0.266864 | 0.0504984 | 1.26E-07 |  | -0.0206 | 0.051 | 0.6857 | 27.92702968 |
| Herpes zoster | rs72835696 | A | G | 0.006802 | 0.402584 | 0.0856012 | 2.56E-06 |  | -0.2061 | 0.1917 | 0.2823 | 22.11838002 |
| Herpes zoster | rs75061358 | G | T | 0.1055 | 0.24542 | 0.0444095 | 3.27E-08 |  | -0.0267 | 0.0512 | 0.6026 | 30.53993697 |
| Herpes zoster | rs797974 | C | T | 0.4096 | -0.112245 | 0.0243397 | 4.00E-06 |  | 0.048 | 0.0319 | 0.1327 | 21.26686952 |
| Herpes zoster | rs926771 | T | C | 0.07865 | -0.288211 | 0.0436426 | 4.01E-11 |  | 0.027 | 0.0587 | 0.645 | 43.6113844 |
| Herpes zoster | rs989993 | A | C | 0.04635 | 0.380402 | 0.0812247 | 2.82E-06 |  | 0.0928 | 0.0746 | 0.2136 | 21.93357133 |

**Additional file 6: Table S2**. Single SNP analysis of the association of GBM on viral infection

|  |  |  |  |  | **GBM** | | |  | **Virus infection** | | |  |
| --- | --- | --- | --- | --- | --- | --- | --- | --- | --- | --- | --- | --- |
| **Outcome** | **SNP** | **Effect allele** | **Other allele** | **Effect allele frequency** | **Beta** | **Standard error** | **P-value** |  | **Beta *** | **Standard error *** | **P-value *** | **F-statistics** |
| Cytomegaloviral disease | rs10889496 | G | A | 0.4695 | -0.11761 | 0.0242264 | 1.21E-06 |  | 0.0122 | 0.087 | 0.8881 | 23.56734898 |
| Cytomegaloviral disease | rs1122530 | G | A | 0.1236 | 0.147371 | 0.0316843 | 3.30E-06 |  | 0.0862 | 0.1312 | 0.5114 | 21.63395029 |
| Cytomegaloviral disease | rs113089273 | T | C | 0.04232 | 0.25776 | 0.0551957 | 3.01E-06 |  | -0.2709 | 0.2144 | 0.2064 | 21.80823685 |
| Cytomegaloviral disease | rs114531477 | A | G | 0.03825 | 0.324092 | 0.069409 | 3.02E-06 |  | -0.0671 | 0.228 | 0.768599 | 21.80243734 |
| Cytomegaloviral disease | rs11551684 | T | C | 0.1706 | -0.147887 | 0.0301716 | 9.51E-07 |  | 0.0982 | 0.1158 | 0.3965 | 24.02499551 |
| Cytomegaloviral disease | rs117157710 | G | A | 0.04296 | 0.271495 | 0.0585753 | 3.57E-06 |  | 0.1386 | 0.2139 | 0.517101 | 21.48298516 |
| Cytomegaloviral disease | rs117265989 | C | T | 0.0214 | -0.467117 | 0.0629887 | 1.21E-13 |  | 0.3851 | 0.2981 | 0.1964 | 54.99536081 |
| Cytomegaloviral disease | rs12332680 | G | A | 0.3322 | -0.114082 | 0.0242868 | 2.64E-06 |  | -0.0376 | 0.0923 | 0.6838 | 22.06447812 |
| Cytomegaloviral disease | rs12449010 | T | C | 0.08774 | -0.207737 | 0.043937 | 2.27E-06 |  | -0.112 | 0.1516 | 0.4598 | 22.35460039 |
| Cytomegaloviral disease | rs12482260 | G | A | 0.01528 | 0.446356 | 0.085082 | 1.55E-07 |  | -0.1483 | 0.3523 | 0.673701 | 27.52246789 |
| Cytomegaloviral disease | rs146122326 | A | G | 0.03015 | 0.446578 | 0.0820452 | 5.24E-08 |  | 0.0917 | 0.2558 | 0.719999 | 29.62704197 |
| Cytomegaloviral disease | rs2110922 | A | C | 0.4572 | 0.106743 | 0.0230252 | 3.55E-06 |  | -0.047 | 0.0868 | 0.587899 | 21.49176021 |
| Cytomegaloviral disease | rs2288369 | A | G | 0.6775 | 0.127081 | 0.0257983 | 8.40E-07 |  | -0.0275 | 0.0925 | 0.7665 | 24.26493151 |
| Cytomegaloviral disease | rs2330761 | C | A | 0.4654 | 0.108058 | 0.0223539 | 1.34E-06 |  | -0.181 | 0.0865 | 0.0364704 | 23.3672305 |
| Cytomegaloviral disease | rs30356 | C | T | 0.2756 | -0.12131 | 0.0236614 | 2.95E-07 |  | 0.0405 | 0.097 | 0.6765 | 26.28526323 |
| Cytomegaloviral disease | rs35312516 | T | C | 0.04686 | 0.206088 | 0.0449243 | 4.49E-06 |  | -0.1744 | 0.2035 | 0.3916 | 21.04470158 |
| Cytomegaloviral disease | rs3761121 | C | T | 0.09086 | 0.24109 | 0.0345589 | 3.03E-12 |  | 0.102 | 0.1501 | 0.4966 | 48.66744792 |
| Cytomegaloviral disease | rs3844166 | A | G | 0.06318 | -0.234127 | 0.0512792 | 4.98E-06 |  | -0.0192 | 0.1761 | 0.9134 | 20.84589367 |
| Cytomegaloviral disease | rs4149909 | G | A | 0.03154 | 0.363112 | 0.0689955 | 1.42E-07 |  | -0.5091 | 0.2468 | 0.0391598 | 27.69744257 |
| Cytomegaloviral disease | rs4150221 | C | T | 0.3194 | -0.123268 | 0.0253274 | 1.13E-06 |  | -0.0436 | 0.0923 | 0.6363 | 23.68751382 |
| Cytomegaloviral disease | rs4984741 | A | G | 0.1707 | 0.118358 | 0.0257405 | 4.26E-06 |  | -0.1854 | 0.1148 | 0.1062 | 21.14274045 |
| Cytomegaloviral disease | rs56064154 | A | C | 0.2181 | -0.156413 | 0.0323743 | 1.36E-06 |  | 0.064 | 0.104 | 0.538 | 23.34236835 |
| Cytomegaloviral disease | rs56168929 | A | G | 0.2823 | 0.125714 | 0.0261174 | 1.48E-06 |  | 0.0456 | 0.0961 | 0.6349 | 23.16900638 |
| Cytomegaloviral disease | rs5995517 | C | T | 0.4195 | 0.106013 | 0.0226246 | 2.79E-06 |  | -0.0806 | 0.0874 | 0.3562 | 21.95616216 |
| Cytomegaloviral disease | rs6006421 | A | G | 0.1323 | -0.138601 | 0.0295874 | 2.81E-06 |  | 0.2297 | 0.1277 | 0.0721506 | 21.94416809 |
| Cytomegaloviral disease | rs6082722 | A | G | 0.008488 | 0.417034 | 0.0913098 | 4.94E-06 |  | 0.3007 | 0.4739 | 0.5257 | 20.85970413 |
| Cytomegaloviral disease | rs62556523 | A | G | 0.04691 | 0.253426 | 0.0521249 | 1.16E-06 |  | 0.4367 | 0.2126 | 0.0399402 | 23.63806204 |
| Cytomegaloviral disease | rs74631778 | G | T | 0.1344 | -0.171312 | 0.0365695 | 2.81E-06 |  | 0.0115 | 0.126 | 0.9275 | 21.94509806 |
| Cytomegaloviral disease | rs75675564 | A | G | 0.05483 | 0.190796 | 0.0414533 | 4.17E-06 |  | -0.1582 | 0.1892 | 0.403 | 21.18460215 |
| Cytomegaloviral disease | rs7718658 | T | C | 0.2761 | 0.11229 | 0.0243882 | 4.14E-06 |  | 0.0933 | 0.0964 | 0.3333 | 21.199356 |
| Cytomegaloviral disease | rs7789987 | C | T | 0.6645 | 0.111305 | 0.0240291 | 3.62E-06 |  | 0.0083 | 0.0913 | 0.9276 | 21.45627557 |
| Cytomegaloviral disease | rs9514622 | A | G | 0.3124 | 0.109803 | 0.023249 | 2.32E-06 |  | 0.0935 | 0.0936 | 0.3183 | 22.30590544 |
| COVID-19 hospitalized | rs10889496 | G | A | 0.3638 | -0.11761 | 0.0242264 | 1.21E-06 |  | 0.025603 | 0.010633 | 0.0160398 | 23.56734898 |
| COVID-19 hospitalized | rs1122530 | G | A | 0.1456 | 0.147371 | 0.0316843 | 3.30E-06 |  | -0.010759 | 0.01341 | 0.4224 | 21.63395029 |
| COVID-19 hospitalized | rs113089273 | T | C | 0.05164 | 0.25776 | 0.0551957 | 3.01E-06 |  | 0.0044276 | 0.023036 | 0.8476 | 21.80823685 |
| COVID-19 hospitalized | rs114531477 | A | G | 0.03934 | 0.324092 | 0.069409 | 3.02E-06 |  | -0.01043 | 0.029955 | 0.727699 | 21.80243734 |
| COVID-19 hospitalized | rs11551684 | T | C | 0.1901 | -0.147887 | 0.0301716 | 9.51E-07 |  | -0.0020604 | 0.012769 | 0.8718 | 24.02499551 |
| COVID-19 hospitalized | rs117157710 | G | A | 0.04304 | 0.271495 | 0.0585753 | 3.57E-06 |  | -0.0048971 | 0.024909 | 0.8441 | 21.48298516 |
| COVID-19 hospitalized | rs117265989 | C | T | 0.04365 | -0.467117 | 0.0629887 | 1.21E-13 |  | -0.021996 | 0.026766 | 0.4112 | 54.99536081 |
| COVID-19 hospitalized | rs12332680 | G | A | 0.3093 | -0.114082 | 0.0242868 | 2.64E-06 |  | 0.018918 | 0.01102 | 0.08603 | 22.06447812 |
| COVID-19 hospitalized | rs12449010 | T | C | 0.08424 | -0.207737 | 0.043937 | 2.27E-06 |  | 0.0018507 | 0.018499 | 0.9203 | 22.35460039 |
| COVID-19 hospitalized | rs12482260 | G | A | 0.02858 | 0.446356 | 0.085082 | 1.55E-07 |  | 0.027763 | 0.033703 | 0.4101 | 27.52246789 |
| COVID-19 hospitalized | rs146122326 | A | G | 0.03316 | 0.446578 | 0.0820452 | 5.24E-08 |  | -0.013993 | 0.035417 | 0.6928 | 29.62704197 |
| COVID-19 hospitalized | rs2110922 | A | C | 0.3894 | 0.106743 | 0.0230252 | 3.55E-06 |  | 0.0082776 | 0.0097429 | 0.3955 | 21.49176021 |
| COVID-19 hospitalized | rs2288369 | A | G | 0.7269 | 0.127081 | 0.0257983 | 8.40E-07 |  | -0.00019604 | 0.010839 | 0.9856 | 24.26493151 |
| COVID-19 hospitalized | rs2330761 | C | A | 0.491 | 0.108058 | 0.0223539 | 1.34E-06 |  | -0.029563 | 0.0096794 | 0.00225699 | 23.3672305 |
| COVID-19 hospitalized | rs30356 | C | T | 0.3216 | -0.12131 | 0.0236614 | 2.95E-07 |  | -0.0027445 | 0.010016 | 0.784099 | 26.28526323 |
| COVID-19 hospitalized | rs35312516 | T | C | 0.06821 | 0.206088 | 0.0449243 | 4.49E-06 |  | 0.019658 | 0.019118 | 0.3039 | 21.04470158 |
| COVID-19 hospitalized | rs3761121 | C | T | 0.1197 | 0.24109 | 0.0345589 | 3.03E-12 |  | -0.0018649 | 0.014831 | 0.8999 | 48.66744792 |
| COVID-19 hospitalized | rs3844166 | A | G | 0.05827 | -0.234127 | 0.0512792 | 4.98E-06 |  | -0.040554 | 0.021232 | 0.0561306 | 20.84589367 |
| COVID-19 hospitalized | rs4149909 | G | A | 0.03957 | 0.363112 | 0.0689955 | 1.42E-07 |  | 0.0035869 | 0.027459 | 0.8961 | 27.69744257 |
| COVID-19 hospitalized | rs4150221 | C | T | 0.2813 | -0.123268 | 0.0253274 | 1.13E-06 |  | -0.0070457 | 0.010631 | 0.5075 | 23.68751382 |
| COVID-19 hospitalized | rs4984741 | A | G | 0.2398 | 0.118358 | 0.0257405 | 4.26E-06 |  | 0.0074804 | 0.010848 | 0.4905 | 21.14274045 |
| COVID-19 hospitalized | rs56064154 | A | C | 0.2111 | -0.156413 | 0.0323743 | 1.36E-06 |  | 0.0002806 | 0.011765 | 0.981 | 23.34236835 |
| COVID-19 hospitalized | rs56168929 | A | G | 0.2572 | 0.125714 | 0.0261174 | 1.48E-06 |  | -0.0010352 | 0.011092 | 0.9256 | 23.16900638 |
| COVID-19 hospitalized | rs5995517 | C | T | 0.3949 | 0.106013 | 0.0226246 | 2.79E-06 |  | -0.0028977 | 0.0096788 | 0.764599 | 21.95616216 |
| COVID-19 hospitalized | rs6006421 | A | G | 0.1625 | -0.138601 | 0.0295874 | 2.81E-06 |  | 0.011073 | 0.012842 | 0.3885 | 21.94416809 |
| COVID-19 hospitalized | rs6082722 | A | G | 0.02481 | 0.417034 | 0.0913098 | 4.94E-06 |  | -0.079391 | 0.032839 | 0.01562 | 20.85970413 |
| COVID-19 hospitalized | rs62556523 | A | G | 0.05471 | 0.253426 | 0.0521249 | 1.16E-06 |  | -0.0025997 | 0.026024 | 0.9204 | 23.63806204 |
| COVID-19 hospitalized | rs74631778 | G | T | 0.1265 | -0.171312 | 0.0365695 | 2.81E-06 |  | -0.0022338 | 0.014798 | 0.88 | 21.94509806 |
| COVID-19 hospitalized | rs75675564 | A | G | 0.08176 | 0.190796 | 0.0414533 | 4.17E-06 |  | 0.0052564 | 0.017301 | 0.7613 | 21.18460215 |
| COVID-19 hospitalized | rs7718658 | T | C | 0.3043 | 0.11229 | 0.0243882 | 4.14E-06 |  | 0.0073585 | 0.010532 | 0.4848 | 21.199356 |
| COVID-19 hospitalized | rs7789987 | C | T | 0.6699 | 0.111305 | 0.0240291 | 3.62E-06 |  | 0.0021106 | 0.010434 | 0.8397 | 21.45627557 |
| COVID-19 hospitalized | rs9514622 | A | G | 0.3466 | 0.109803 | 0.023249 | 2.32E-06 |  | 0.013387 | 0.010307 | 0.194 | 22.30590544 |
| Infectious mononucleosis | rs10889496 | G | A | 0.4694 | -0.11761 | 0.0242264 | 1.21E-06 |  | -0.1004 | 0.0413 | 0.0150501 | 23.56734898 |
| Infectious mononucleosis | rs1122530 | G | A | 0.1236 | 0.147371 | 0.0316843 | 3.30E-06 |  | -0.06 | 0.0622 | 0.335 | 21.63395029 |
| Infectious mononucleosis | rs113089273 | T | C | 0.04232 | 0.25776 | 0.0551957 | 3.01E-06 |  | -0.0984 | 0.1009 | 0.3296 | 21.80823685 |
| Infectious mononucleosis | rs114531477 | A | G | 0.0382 | 0.324092 | 0.069409 | 3.02E-06 |  | -0.2307 | 0.1087 | 0.0337404 | 21.80243734 |
| Infectious mononucleosis | rs11551684 | T | C | 0.1705 | -0.147887 | 0.0301716 | 9.51E-07 |  | -0.0958 | 0.055 | 0.08127 | 24.02499551 |
| Infectious mononucleosis | rs117157710 | G | A | 0.04291 | 0.271495 | 0.0585753 | 3.57E-06 |  | -0.2071 | 0.1018 | 0.0418302 | 21.48298516 |
| Infectious mononucleosis | rs117265989 | C | T | 0.02143 | -0.467117 | 0.0629887 | 1.21E-13 |  | 0.3831 | 0.1423 | 0.00711295 | 54.99536081 |
| Infectious mononucleosis | rs12332680 | G | A | 0.3322 | -0.114082 | 0.0242868 | 2.64E-06 |  | -0.0141 | 0.0437 | 0.7472 | 22.06447812 |
| Infectious mononucleosis | rs12449010 | T | C | 0.08777 | -0.207737 | 0.043937 | 2.27E-06 |  | 0.0333 | 0.0722 | 0.6447 | 22.35460039 |
| Infectious mononucleosis | rs12482260 | G | A | 0.01528 | 0.446356 | 0.085082 | 1.55E-07 |  | 0.0047 | 0.1666 | 0.9774 | 27.52246789 |
| Infectious mononucleosis | rs146122326 | A | G | 0.03015 | 0.446578 | 0.0820452 | 5.24E-08 |  | -0.0253 | 0.1209 | 0.834 | 29.62704197 |
| Infectious mononucleosis | rs2110922 | A | C | 0.4572 | 0.106743 | 0.0230252 | 3.55E-06 |  | -0.0294 | 0.0412 | 0.4757 | 21.49176021 |
| Infectious mononucleosis | rs2288369 | A | G | 0.6776 | 0.127081 | 0.0257983 | 8.40E-07 |  | 0.0078 | 0.0439 | 0.8597 | 24.26493151 |
| Infectious mononucleosis | rs2330761 | C | A | 0.4656 | 0.108058 | 0.0223539 | 1.34E-06 |  | 0.0878 | 0.0411 | 0.0327499 | 23.3672305 |
| Infectious mononucleosis | rs30356 | C | T | 0.2756 | -0.12131 | 0.0236614 | 2.95E-07 |  | -0.0241 | 0.0461 | 0.600999 | 26.28526323 |
| Infectious mononucleosis | rs35312516 | T | C | 0.04687 | 0.206088 | 0.0449243 | 4.49E-06 |  | 0.0241 | 0.0968 | 0.8032 | 21.04470158 |
| Infectious mononucleosis | rs3761121 | C | T | 0.09086 | 0.24109 | 0.0345589 | 3.03E-12 |  | -0.0035 | 0.0712 | 0.961 | 48.66744792 |
| Infectious mononucleosis | rs3844166 | A | G | 0.06313 | -0.234127 | 0.0512792 | 4.98E-06 |  | -0.1793 | 0.0837 | 0.0321603 | 20.84589367 |
| Infectious mononucleosis | rs4149909 | G | A | 0.03156 | 0.363112 | 0.0689955 | 1.42E-07 |  | 0.0446 | 0.1188 | 0.707499 | 27.69744257 |
| Infectious mononucleosis | rs4150221 | C | T | 0.3194 | -0.123268 | 0.0253274 | 1.13E-06 |  | -0.004 | 0.0438 | 0.9265 | 23.68751382 |
| Infectious mononucleosis | rs4984741 | A | G | 0.1708 | 0.118358 | 0.0257405 | 4.26E-06 |  | 0.0053 | 0.0544 | 0.9217 | 21.14274045 |
| Infectious mononucleosis | rs56064154 | A | C | 0.218 | -0.156413 | 0.0323743 | 1.36E-06 |  | 0.0048 | 0.0494 | 0.923 | 23.34236835 |
| Infectious mononucleosis | rs56168929 | A | G | 0.2824 | 0.125714 | 0.0261174 | 1.48E-06 |  | 0.0939 | 0.0456 | 0.0393704 | 23.16900638 |
| Infectious mononucleosis | rs5995517 | C | T | 0.4195 | 0.106013 | 0.0226246 | 2.79E-06 |  | -0.0098 | 0.0415 | 0.8135 | 21.95616216 |
| Infectious mononucleosis | rs6006421 | A | G | 0.1323 | -0.138601 | 0.0295874 | 2.81E-06 |  | 0.0395 | 0.0607 | 0.515101 | 21.94416809 |
| Infectious mononucleosis | rs6082722 | A | G | 0.008502 | 0.417034 | 0.0913098 | 4.94E-06 |  | 0.3266 | 0.2229 | 0.1429 | 20.85970413 |
| Infectious mononucleosis | rs62556523 | A | G | 0.04687 | 0.253426 | 0.0521249 | 1.16E-06 |  | -0.1389 | 0.1 | 0.1648 | 23.63806204 |
| Infectious mononucleosis | rs74631778 | G | T | 0.1344 | -0.171312 | 0.0365695 | 2.81E-06 |  | 0.0345 | 0.0602 | 0.5667 | 21.94509806 |
| Infectious mononucleosis | rs75675564 | A | G | 0.05482 | 0.190796 | 0.0414533 | 4.17E-06 |  | -0.0968 | 0.0903 | 0.2839 | 21.18460215 |
| Infectious mononucleosis | rs7718658 | T | C | 0.276 | 0.11229 | 0.0243882 | 4.14E-06 |  | -0.0535 | 0.0458 | 0.2433 | 21.199356 |
| Infectious mononucleosis | rs7789987 | C | T | 0.6645 | 0.111305 | 0.0240291 | 3.62E-06 |  | 0.0052 | 0.0433 | 0.9048 | 21.45627557 |
| Infectious mononucleosis | rs9514622 | A | G | 0.3124 | 0.109803 | 0.023249 | 2.32E-06 |  | -0.0169 | 0.0444 | 0.7035 | 22.30590544 |
| Viral hepatitis | rs10889496 | G | A | 0.4694 | -0.11761 | 0.0242264 | 1.21E-06 |  | -0.0181 | 0.0415 | 0.663399 | 23.56734898 |
| Viral hepatitis | rs1122530 | G | A | 0.1235 | 0.147371 | 0.0316843 | 3.30E-06 |  | -0.0031 | 0.0632 | 0.9604 | 21.63395029 |
| Viral hepatitis | rs113089273 | T | C | 0.04231 | 0.25776 | 0.0551957 | 3.01E-06 |  | 0.0134 | 0.1028 | 0.8967 | 21.80823685 |
| Viral hepatitis | rs114531477 | A | G | 0.03815 | 0.324092 | 0.069409 | 3.02E-06 |  | 0.2423 | 0.1082 | 0.0251403 | 21.80243734 |
| Viral hepatitis | rs11551684 | T | C | 0.1705 | -0.147887 | 0.0301716 | 9.51E-07 |  | -0.0594 | 0.0554 | 0.2839 | 24.02499551 |
| Viral hepatitis | rs117157710 | G | A | 0.04292 | 0.271495 | 0.0585753 | 3.57E-06 |  | 0.1196 | 0.1011 | 0.2369 | 21.48298516 |
| Viral hepatitis | rs117265989 | C | T | 0.02143 | -0.467117 | 0.0629887 | 1.21E-13 |  | 0.0785 | 0.1463 | 0.5916 | 54.99536081 |
| Viral hepatitis | rs12332680 | G | A | 0.3323 | -0.114082 | 0.0242868 | 2.64E-06 |  | -0.0653 | 0.0439 | 0.1367 | 22.06447812 |
| Viral hepatitis | rs12449010 | T | C | 0.08771 | -0.207737 | 0.043937 | 2.27E-06 |  | 0.0399 | 0.073 | 0.5847 | 22.35460039 |
| Viral hepatitis | rs12482260 | G | A | 0.01529 | 0.446356 | 0.085082 | 1.55E-07 |  | 0.0255 | 0.169 | 0.8802 | 27.52246789 |
| Viral hepatitis | rs146122326 | A | G | 0.03015 | 0.446578 | 0.0820452 | 5.24E-08 |  | 0.045 | 0.124 | 0.7166 | 29.62704197 |
| Viral hepatitis | rs2110922 | A | C | 0.4574 | 0.106743 | 0.0230252 | 3.55E-06 |  | -0.035 | 0.0415 | 0.3989 | 21.49176021 |
| Viral hepatitis | rs2288369 | A | G | 0.6777 | 0.127081 | 0.0257983 | 8.40E-07 |  | -0.0176 | 0.0441 | 0.6909 | 24.26493151 |
| Viral hepatitis | rs2330761 | C | A | 0.4653 | 0.108058 | 0.0223539 | 1.34E-06 |  | -0.0212 | 0.0413 | 0.6072 | 23.3672305 |
| Viral hepatitis | rs30356 | C | T | 0.2755 | -0.12131 | 0.0236614 | 2.95E-07 |  | 0.0308 | 0.0462 | 0.5055 | 26.28526323 |
| Viral hepatitis | rs35312516 | T | C | 0.04687 | 0.206088 | 0.0449243 | 4.49E-06 |  | 0.237 | 0.0968 | 0.01439 | 21.04470158 |
| Viral hepatitis | rs3761121 | C | T | 0.09095 | 0.24109 | 0.0345589 | 3.03E-12 |  | 0.0566 | 0.0723 | 0.434 | 48.66744792 |
| Viral hepatitis | rs3844166 | A | G | 0.06307 | -0.234127 | 0.0512792 | 4.98E-06 |  | -0.0357 | 0.0852 | 0.674899 | 20.84589367 |
| Viral hepatitis | rs4149909 | G | A | 0.03155 | 0.363112 | 0.0689955 | 1.42E-07 |  | -0.2148 | 0.1184 | 0.0696899 | 27.69744257 |
| Viral hepatitis | rs4150221 | C | T | 0.3194 | -0.123268 | 0.0253274 | 1.13E-06 |  | -0.0031 | 0.0441 | 0.9432 | 23.68751382 |
| Viral hepatitis | rs4984741 | A | G | 0.1707 | 0.118358 | 0.0257405 | 4.26E-06 |  | 0.0167 | 0.0553 | 0.762801 | 21.14274045 |
| Viral hepatitis | rs56064154 | A | C | 0.2181 | -0.156413 | 0.0323743 | 1.36E-06 |  | -0.0509 | 0.0498 | 0.3074 | 23.34236835 |
| Viral hepatitis | rs56168929 | A | G | 0.2824 | 0.125714 | 0.0261174 | 1.48E-06 |  | -0.038 | 0.0459 | 0.4076 | 23.16900638 |
| Viral hepatitis | rs5995517 | C | T | 0.4196 | 0.106013 | 0.0226246 | 2.79E-06 |  | 0.0031 | 0.0418 | 0.94 | 21.95616216 |
| Viral hepatitis | rs6006421 | A | G | 0.1322 | -0.138601 | 0.0295874 | 2.81E-06 |  | 0.0598 | 0.0614 | 0.3302 | 21.94416809 |
| Viral hepatitis | rs6082722 | A | G | 0.008506 | 0.417034 | 0.0913098 | 4.94E-06 |  | -0.2611 | 0.2268 | 0.2495 | 20.85970413 |
| Viral hepatitis | rs62556523 | A | G | 0.04683 | 0.253426 | 0.0521249 | 1.16E-06 |  | -0.1344 | 0.1024 | 0.1897 | 23.63806204 |
| Viral hepatitis | rs74631778 | G | T | 0.1344 | -0.171312 | 0.0365695 | 2.81E-06 |  | 0.0011 | 0.0606 | 0.9852 | 21.94509806 |
| Viral hepatitis | rs75675564 | A | G | 0.05486 | 0.190796 | 0.0414533 | 4.17E-06 |  | 0.0414 | 0.0912 | 0.6495 | 21.18460215 |
| Viral hepatitis | rs7718658 | T | C | 0.2761 | 0.11229 | 0.0243882 | 4.14E-06 |  | 0.0459 | 0.0463 | 0.3216 | 21.199356 |
| Viral hepatitis | rs7789987 | C | T | 0.6645 | 0.111305 | 0.0240291 | 3.62E-06 |  | -0.06 | 0.0436 | 0.1688 | 21.45627557 |
| Viral hepatitis | rs9514622 | A | G | 0.3123 | 0.109803 | 0.023249 | 2.32E-06 |  | 0.0482 | 0.0446 | 0.2795 | 22.30590544 |
| Herpesviral infections | rs10889496 | G | A | 0.4693 | -0.11761 | 0.0242264 | 1.21E-06 |  | -0.0423 | 0.0359 | 0.2389 | 23.56734898 |
| Herpesviral infections | rs1122530 | G | A | 0.1235 | 0.147371 | 0.0316843 | 3.30E-06 |  | 0.0176 | 0.0542 | 0.744699 | 21.63395029 |
| Herpesviral infections | rs113089273 | T | C | 0.04234 | 0.25776 | 0.0551957 | 3.01E-06 |  | -0.0798 | 0.0885 | 0.3673 | 21.80823685 |
| Herpesviral infections | rs114531477 | A | G | 0.03815 | 0.324092 | 0.069409 | 3.02E-06 |  | 0.0234 | 0.0931 | 0.8012 | 21.80243734 |
| Herpesviral infections | rs11551684 | T | C | 0.1706 | -0.147887 | 0.0301716 | 9.51E-07 |  | -0.0158 | 0.0477 | 0.739799 | 24.02499551 |
| Herpesviral infections | rs117157710 | G | A | 0.04296 | 0.271495 | 0.0585753 | 3.57E-06 |  | -0.0856 | 0.0878 | 0.3298 | 21.48298516 |
| Herpesviral infections | rs117265989 | C | T | 0.02139 | -0.467117 | 0.0629887 | 1.21E-13 |  | -0.0273 | 0.1242 | 0.8259 | 54.99536081 |
| Herpesviral infections | rs12332680 | G | A | 0.3323 | -0.114082 | 0.0242868 | 2.64E-06 |  | -0.0052 | 0.038 | 0.8917 | 22.06447812 |
| Herpesviral infections | rs12449010 | T | C | 0.08764 | -0.207737 | 0.043937 | 2.27E-06 |  | 0.0282 | 0.063 | 0.6546 | 22.35460039 |
| Herpesviral infections | rs12482260 | G | A | 0.01532 | 0.446356 | 0.085082 | 1.55E-07 |  | -0.1674 | 0.1471 | 0.255 | 27.52246789 |
| Herpesviral infections | rs146122326 | A | G | 0.03014 | 0.446578 | 0.0820452 | 5.24E-08 |  | -0.033 | 0.1062 | 0.756 | 29.62704197 |
| Herpesviral infections | rs2110922 | A | C | 0.4572 | 0.106743 | 0.0230252 | 3.55E-06 |  | 0.0542 | 0.0359 | 0.131 | 21.49176021 |
| Herpesviral infections | rs2288369 | A | G | 0.6777 | 0.127081 | 0.0257983 | 8.40E-07 |  | 0.064 | 0.0381 | 0.0934501 | 24.26493151 |
| Herpesviral infections | rs2330761 | C | A | 0.4653 | 0.108058 | 0.0223539 | 1.34E-06 |  | -0.0198 | 0.0357 | 0.5802 | 23.3672305 |
| Herpesviral infections | rs30356 | C | T | 0.2755 | -0.12131 | 0.0236614 | 2.95E-07 |  | -0.0215 | 0.0401 | 0.5923 | 26.28526323 |
| Herpesviral infections | rs35312516 | T | C | 0.04687 | 0.206088 | 0.0449243 | 4.49E-06 |  | 0.05 | 0.0839 | 0.5512 | 21.04470158 |
| Herpesviral infections | rs3761121 | C | T | 0.09092 | 0.24109 | 0.0345589 | 3.03E-12 |  | 0.0826 | 0.0623 | 0.185 | 48.66744792 |
| Herpesviral infections | rs3844166 | A | G | 0.06303 | -0.234127 | 0.0512792 | 4.98E-06 |  | -0.0781 | 0.0735 | 0.2881 | 20.84589367 |
| Herpesviral infections | rs4149909 | G | A | 0.0316 | 0.363112 | 0.0689955 | 1.42E-07 |  | 0.3165 | 0.1019 | 0.00189802 | 27.69744257 |
| Herpesviral infections | rs4150221 | C | T | 0.3195 | -0.123268 | 0.0253274 | 1.13E-06 |  | -0.0044 | 0.0381 | 0.9089 | 23.68751382 |
| Herpesviral infections | rs4984741 | A | G | 0.1707 | 0.118358 | 0.0257405 | 4.26E-06 |  | -0.064 | 0.0477 | 0.1793 | 21.14274045 |
| Herpesviral infections | rs56064154 | A | C | 0.2182 | -0.156413 | 0.0323743 | 1.36E-06 |  | -0.0246 | 0.0431 | 0.5674 | 23.34236835 |
| Herpesviral infections | rs56168929 | A | G | 0.2825 | 0.125714 | 0.0261174 | 1.48E-06 |  | -0.0156 | 0.0396 | 0.6944 | 23.16900638 |
| Herpesviral infections | rs5995517 | C | T | 0.4198 | 0.106013 | 0.0226246 | 2.79E-06 |  | -0.032 | 0.0361 | 0.3754 | 21.95616216 |
| Herpesviral infections | rs6006421 | A | G | 0.1324 | -0.138601 | 0.0295874 | 2.81E-06 |  | 0.027 | 0.0527 | 0.6087 | 21.94416809 |
| Herpesviral infections | rs6082722 | A | G | 0.00851 | 0.417034 | 0.0913098 | 4.94E-06 |  | 0.1105 | 0.1974 | 0.5755 | 20.85970413 |
| Herpesviral infections | rs62556523 | A | G | 0.04679 | 0.253426 | 0.0521249 | 1.16E-06 |  | -0.0393 | 0.0878 | 0.6544 | 23.63806204 |
| Herpesviral infections | rs74631778 | G | T | 0.1344 | -0.171312 | 0.0365695 | 2.81E-06 |  | 0.0285 | 0.052 | 0.5829 | 21.94509806 |
| Herpesviral infections | rs75675564 | A | G | 0.0549 | 0.190796 | 0.0414533 | 4.17E-06 |  | 0.0071 | 0.0786 | 0.9278 | 21.18460215 |
| Herpesviral infections | rs7718658 | T | C | 0.2761 | 0.11229 | 0.0243882 | 4.14E-06 |  | 0.0569 | 0.0398 | 0.1535 | 21.199356 |
| Herpesviral infections | rs7789987 | C | T | 0.6645 | 0.111305 | 0.0240291 | 3.62E-06 |  | 0.0034 | 0.0378 | 0.9287 | 21.45627557 |
| Herpesviral infections | rs9514622 | A | G | 0.3124 | 0.109803 | 0.023249 | 2.32E-06 |  | 0.0037 | 0.0386 | 0.923 | 22.30590544 |
| HIV diease | rs10889496 | G | A | 0.4694 | -0.11761 | 0.0242264 | 1.21E-06 |  | 0.0397 | 0.0761 | 0.601399 | 23.56734898 |
| HIV diease | rs1122530 | G | A | 0.1235 | 0.147371 | 0.0316843 | 3.30E-06 |  | 0.1487 | 0.1147 | 0.195 | 21.63395029 |
| HIV diease | rs113089273 | T | C | 0.04231 | 0.25776 | 0.0551957 | 3.01E-06 |  | -0.2565 | 0.1864 | 0.1689 | 21.80823685 |
| HIV diease | rs114531477 | A | G | 0.03815 | 0.324092 | 0.069409 | 3.02E-06 |  | -0.2003 | 0.1954 | 0.3054 | 21.80243734 |
| HIV diease | rs11551684 | T | C | 0.1705 | -0.147887 | 0.0301716 | 9.51E-07 |  | 0.0453 | 0.1011 | 0.653699 | 24.02499551 |
| HIV diease | rs117157710 | G | A | 0.04292 | 0.271495 | 0.0585753 | 3.57E-06 |  | 0.0102 | 0.1851 | 0.956 | 21.48298516 |
| HIV diease | rs117265989 | C | T | 0.02143 | -0.467117 | 0.0629887 | 1.21E-13 |  | -0.1662 | 0.2654 | 0.5311 | 54.99536081 |
| HIV diease | rs12332680 | G | A | 0.3323 | -0.114082 | 0.0242868 | 2.64E-06 |  | 0.0418 | 0.0802 | 0.601999 | 22.06447812 |
| HIV diease | rs12449010 | T | C | 0.08771 | -0.207737 | 0.043937 | 2.27E-06 |  | -0.1659 | 0.1351 | 0.2193 | 22.35460039 |
| HIV diease | rs12482260 | G | A | 0.01529 | 0.446356 | 0.085082 | 1.55E-07 |  | 0.2717 | 0.3083 | 0.3781 | 27.52246789 |
| HIV diease | rs146122326 | A | G | 0.03015 | 0.446578 | 0.0820452 | 5.24E-08 |  | 0.0166 | 0.2246 | 0.9411 | 29.62704197 |
| HIV diease | rs2110922 | A | C | 0.4574 | 0.106743 | 0.0230252 | 3.55E-06 |  | -0.05 | 0.0758 | 0.5091 | 21.49176021 |
| HIV diease | rs2288369 | A | G | 0.6777 | 0.127081 | 0.0257983 | 8.40E-07 |  | 0.0182 | 0.0809 | 0.8218 | 24.26493151 |
| HIV diease | rs2330761 | C | A | 0.4653 | 0.108058 | 0.0223539 | 1.34E-06 |  | 0.0273 | 0.0756 | 0.718199 | 23.3672305 |
| HIV diease | rs30356 | C | T | 0.2755 | -0.12131 | 0.0236614 | 2.95E-07 |  | -0.0335 | 0.0847 | 0.6927 | 26.28526323 |
| HIV diease | rs35312516 | T | C | 0.04687 | 0.206088 | 0.0449243 | 4.49E-06 |  | 0.3159 | 0.1768 | 0.0740508 | 21.04470158 |
| HIV diease | rs3761121 | C | T | 0.09095 | 0.24109 | 0.0345589 | 3.03E-12 |  | -0.0916 | 0.1326 | 0.4897 | 48.66744792 |
| HIV diease | rs3844166 | A | G | 0.06307 | -0.234127 | 0.0512792 | 4.98E-06 |  | 0.1397 | 0.1551 | 0.3677 | 20.84589367 |
| HIV diease | rs4149909 | G | A | 0.03155 | 0.363112 | 0.0689955 | 1.42E-07 |  | 0.1172 | 0.2159 | 0.5874 | 27.69744257 |
| HIV diease | rs4150221 | C | T | 0.3194 | -0.123268 | 0.0253274 | 1.13E-06 |  | -0.0543 | 0.0808 | 0.501601 | 23.68751382 |
| HIV diease | rs4984741 | A | G | 0.1707 | 0.118358 | 0.0257405 | 4.26E-06 |  | -0.0681 | 0.1012 | 0.501 | 21.14274045 |
| HIV diease | rs56064154 | A | C | 0.2181 | -0.156413 | 0.0323743 | 1.36E-06 |  | 0.0237 | 0.0914 | 0.7955 | 23.34236835 |
| HIV diease | rs56168929 | A | G | 0.2824 | 0.125714 | 0.0261174 | 1.48E-06 |  | -0.0031 | 0.0839 | 0.9706 | 23.16900638 |
| HIV diease | rs5995517 | C | T | 0.4196 | 0.106013 | 0.0226246 | 2.79E-06 |  | -0.0372 | 0.0764 | 0.6265 | 21.95616216 |
| HIV diease | rs6006421 | A | G | 0.1322 | -0.138601 | 0.0295874 | 2.81E-06 |  | -0.0943 | 0.1127 | 0.4025 | 21.94416809 |
| HIV diease | rs6082722 | A | G | 0.008506 | 0.417034 | 0.0913098 | 4.94E-06 |  | 0.3289 | 0.4179 | 0.4312 | 20.85970413 |
| HIV diease | rs62556523 | A | G | 0.04683 | 0.253426 | 0.0521249 | 1.16E-06 |  | 0.1991 | 0.1857 | 0.2835 | 23.63806204 |
| HIV diease | rs74631778 | G | T | 0.1344 | -0.171312 | 0.0365695 | 2.81E-06 |  | -0.0815 | 0.1109 | 0.4622 | 21.94509806 |
| HIV diease | rs75675564 | A | G | 0.05486 | 0.190796 | 0.0414533 | 4.17E-06 |  | -0.0694 | 0.1671 | 0.677801 | 21.18460215 |
| HIV diease | rs7718658 | T | C | 0.2761 | 0.11229 | 0.0243882 | 4.14E-06 |  | -0.0289 | 0.0851 | 0.7345 | 21.199356 |
| HIV diease | rs7789987 | C | T | 0.6645 | 0.111305 | 0.0240291 | 3.62E-06 |  | -0.0037 | 0.08 | 0.9635 | 21.45627557 |
| HIV diease | rs9514622 | A | G | 0.3123 | 0.109803 | 0.023249 | 2.32E-06 |  | -0.1425 | 0.0818 | 0.0813992 | 22.30590544 |
| HPV | rs2110922 | A | C | 0.1706 | 0.106743 | 0.0230252 | 3.55E-06 |  | -0.03068 | 0.04728 | 0.5165 | 21.49176021 |
| HPV | rs56168929 | A | G | 0.2506 | 0.125714 | 0.0261174 | 1.48E-06 |  | -0.1926 | 0.136596 | 0.1588 | 23.16900638 |
| HPV | rs7718658 | T | C | 0.03014 | 0.11229 | 0.0243882 | 4.14E-06 |  | 0.002803 | 0.0471251 | 0.9526 | 21.199356 |
| HPV | rs12449010 | T | C | 0.00851 | -0.207737 | 0.043937 | 2.27E-06 |  | -0.09263 | 0.0823378 | 0.2607 | 22.35460039 |
| HPV | rs6082722 | A | G | 0.09148 | 0.417034 | 0.0913098 | 4.94E-06 |  | -0.003607 | 0.15362 | 0.9813 | 20.85970413 |
| measles | rs10889496 | G | A | 0.4693 | -0.11761 | 0.0242264 | 1.21E-06 |  | -0.0169 | 0.1064 | 0.8737 | 23.56734898 |
| measles | rs1122530 | G | A | 0.1235 | 0.147371 | 0.0316843 | 3.30E-06 |  | -0.0617 | 0.1607 | 0.7012 | 21.63395029 |
| measles | rs113089273 | T | C | 0.04237 | 0.25776 | 0.0551957 | 3.01E-06 |  | 0.2427 | 0.2618 | 0.3538 | 21.80823685 |
| measles | rs114531477 | A | G | 0.03814 | 0.324092 | 0.069409 | 3.02E-06 |  | -0.0673 | 0.2764 | 0.8075 | 21.80243734 |
| measles | rs11551684 | T | C | 0.1706 | -0.147887 | 0.0301716 | 9.51E-07 |  | 0.041 | 0.1417 | 0.772499 | 24.02499551 |
| measles | rs117157710 | G | A | 0.04299 | 0.271495 | 0.0585753 | 3.57E-06 |  | 0.1195 | 0.2608 | 0.6469 | 21.48298516 |
| measles | rs117265989 | C | T | 0.0214 | -0.467117 | 0.0629887 | 1.21E-13 |  | -0.0938 | 0.3663 | 0.7979 | 54.99536081 |
| measles | rs12332680 | G | A | 0.3323 | -0.114082 | 0.0242868 | 2.64E-06 |  | -0.0099 | 0.1126 | 0.9302 | 22.06447812 |
| measles | rs12449010 | T | C | 0.0876 | -0.207737 | 0.043937 | 2.27E-06 |  | -0.3361 | 0.1863 | 0.0712492 | 22.35460039 |
| measles | rs12482260 | G | A | 0.01535 | 0.446356 | 0.085082 | 1.55E-07 |  | 0.7308 | 0.4357 | 0.0935104 | 27.52246789 |
| measles | rs146122326 | A | G | 0.03015 | 0.446578 | 0.0820452 | 5.24E-08 |  | -0.1734 | 0.3125 | 0.579001 | 29.62704197 |
| measles | rs2110922 | A | C | 0.4572 | 0.106743 | 0.0230252 | 3.55E-06 |  | 0.1077 | 0.1063 | 0.3111 | 21.49176021 |
| measles | rs2288369 | A | G | 0.6776 | 0.127081 | 0.0257983 | 8.40E-07 |  | 0.0041 | 0.1131 | 0.9707 | 24.26493151 |
| measles | rs2330761 | C | A | 0.4653 | 0.108058 | 0.0223539 | 1.34E-06 |  | -0.0866 | 0.1061 | 0.4145 | 23.3672305 |
| measles | rs30356 | C | T | 0.2755 | -0.12131 | 0.0236614 | 2.95E-07 |  | -0.0807 | 0.1189 | 0.497299 | 26.28526323 |
| measles | rs35312516 | T | C | 0.04686 | 0.206088 | 0.0449243 | 4.49E-06 |  | 0.3217 | 0.2493 | 0.1969 | 21.04470158 |
| measles | rs3761121 | C | T | 0.09087 | 0.24109 | 0.0345589 | 3.03E-12 |  | 0.0263 | 0.1844 | 0.8865 | 48.66744792 |
| measles | rs3844166 | A | G | 0.06307 | -0.234127 | 0.0512792 | 4.98E-06 |  | 0.0998 | 0.2169 | 0.645601 | 20.84589367 |
| measles | rs4149909 | G | A | 0.03153 | 0.363112 | 0.0689955 | 1.42E-07 |  | -0.1216 | 0.3023 | 0.687401 | 27.69744257 |
| measles | rs4150221 | C | T | 0.3195 | -0.123268 | 0.0253274 | 1.13E-06 |  | 0.0174 | 0.1129 | 0.8776 | 23.68751382 |
| measles | rs4984741 | A | G | 0.1707 | 0.118358 | 0.0257405 | 4.26E-06 |  | -0.1282 | 0.1411 | 0.3638 | 21.14274045 |
| measles | rs56064154 | A | C | 0.2182 | -0.156413 | 0.0323743 | 1.36E-06 |  | -0.0634 | 0.1276 | 0.6193 | 23.34236835 |
| measles | rs56168929 | A | G | 0.2825 | 0.125714 | 0.0261174 | 1.48E-06 |  | -0.1291 | 0.1175 | 0.2719 | 23.16900638 |
| measles | rs5995517 | C | T | 0.4198 | 0.106013 | 0.0226246 | 2.79E-06 |  | -0.0262 | 0.1072 | 0.8069 | 21.95616216 |
| measles | rs6006421 | A | G | 0.1323 | -0.138601 | 0.0295874 | 2.81E-06 |  | -0.0065 | 0.1568 | 0.967 | 21.94416809 |
| measles | rs6082722 | A | G | 0.008498 | 0.417034 | 0.0913098 | 4.94E-06 |  | -0.7089 | 0.583 | 0.224 | 20.85970413 |
| measles | rs62556523 | A | G | 0.0468 | 0.253426 | 0.0521249 | 1.16E-06 |  | -0.2428 | 0.2596 | 0.3497 | 23.63806204 |
| measles | rs74631778 | G | T | 0.1344 | -0.171312 | 0.0365695 | 2.81E-06 |  | 0.2515 | 0.1544 | 0.1033 | 21.94509806 |
| measles | rs75675564 | A | G | 0.05491 | 0.190796 | 0.0414533 | 4.17E-06 |  | 0.0897 | 0.2341 | 0.701601 | 21.18460215 |
| measles | rs7718658 | T | C | 0.276 | 0.11229 | 0.0243882 | 4.14E-06 |  | -0.079 | 0.1181 | 0.5036 | 21.199356 |
| measles | rs7789987 | C | T | 0.6645 | 0.111305 | 0.0240291 | 3.62E-06 |  | 0.0393 | 0.1119 | 0.7252 | 21.45627557 |
| measles | rs9514622 | A | G | 0.3124 | 0.109803 | 0.023249 | 2.32E-06 |  | 0.0779 | 0.1147 | 0.4968 | 22.30590544 |
| mumps | rs10889496 | G | A | 0.4696 | -0.11761 | 0.0242264 | 1.21E-06 |  | 0.0653 | 0.0684 | 0.3401 | 23.56734898 |
| mumps | rs1122530 | G | A | 0.1236 | 0.147371 | 0.0316843 | 3.30E-06 |  | -0.1608 | 0.1034 | 0.1198 | 21.63395029 |
| mumps | rs113089273 | T | C | 0.04235 | 0.25776 | 0.0551957 | 3.01E-06 |  | 0.1274 | 0.1678 | 0.4476 | 21.80823685 |
| mumps | rs114531477 | A | G | 0.03825 | 0.324092 | 0.069409 | 3.02E-06 |  | -0.0293 | 0.178 | 0.8691 | 21.80243734 |
| mumps | rs11551684 | T | C | 0.1705 | -0.147887 | 0.0301716 | 9.51E-07 |  | -0.0076 | 0.0912 | 0.9332 | 24.02499551 |
| mumps | rs117157710 | G | A | 0.04296 | 0.271495 | 0.0585753 | 3.57E-06 |  | 0.0186 | 0.1677 | 0.9118 | 21.48298516 |
| mumps | rs117265989 | C | T | 0.02139 | -0.467117 | 0.0629887 | 1.21E-13 |  | -0.0146 | 0.2364 | 0.9508 | 54.99536081 |
| mumps | rs12332680 | G | A | 0.3323 | -0.114082 | 0.0242868 | 2.64E-06 |  | 0.0824 | 0.0725 | 0.2556 | 22.06447812 |
| mumps | rs12449010 | T | C | 0.08776 | -0.207737 | 0.043937 | 2.27E-06 |  | 0.001 | 0.1194 | 0.9934 | 22.35460039 |
| mumps | rs12482260 | G | A | 0.01529 | 0.446356 | 0.085082 | 1.55E-07 |  | 0.324 | 0.2799 | 0.2471 | 27.52246789 |
| mumps | rs146122326 | A | G | 0.03015 | 0.446578 | 0.0820452 | 5.24E-08 |  | -0.0131 | 0.2015 | 0.9483 | 29.62704197 |
| mumps | rs2110922 | A | C | 0.4573 | 0.106743 | 0.0230252 | 3.55E-06 |  | -0.0201 | 0.0684 | 0.768399 | 21.49176021 |
| mumps | rs2288369 | A | G | 0.6775 | 0.127081 | 0.0257983 | 8.40E-07 |  | -0.088 | 0.0727 | 0.2262 | 24.26493151 |
| mumps | rs2330761 | C | A | 0.4654 | 0.108058 | 0.0223539 | 1.34E-06 |  | -0.0613 | 0.0681 | 0.3678 | 23.3672305 |
| mumps | rs30356 | C | T | 0.2756 | -0.12131 | 0.0236614 | 2.95E-07 |  | -0.0051 | 0.0763 | 0.9467 | 26.28526323 |
| mumps | rs35312516 | T | C | 0.04688 | 0.206088 | 0.0449243 | 4.49E-06 |  | 0.087 | 0.1595 | 0.5854 | 21.04470158 |
| mumps | rs3761121 | C | T | 0.09085 | 0.24109 | 0.0345589 | 3.03E-12 |  | -0.0225 | 0.1187 | 0.8495 | 48.66744792 |
| mumps | rs3844166 | A | G | 0.0632 | -0.234127 | 0.0512792 | 4.98E-06 |  | 0.0998 | 0.1394 | 0.4741 | 20.84589367 |
| mumps | rs4149909 | G | A | 0.03156 | 0.363112 | 0.0689955 | 1.42E-07 |  | 0.0623 | 0.1945 | 0.7487 | 27.69744257 |
| mumps | rs4150221 | C | T | 0.3194 | -0.123268 | 0.0253274 | 1.13E-06 |  | 0.0734 | 0.0725 | 0.3111 | 23.68751382 |
| mumps | rs4984741 | A | G | 0.1708 | 0.118358 | 0.0257405 | 4.26E-06 |  | 0.0101 | 0.0907 | 0.9111 | 21.14274045 |
| mumps | rs56064154 | A | C | 0.2181 | -0.156413 | 0.0323743 | 1.36E-06 |  | 0.1853 | 0.0819 | 0.0236102 | 23.34236835 |
| mumps | rs56168929 | A | G | 0.2822 | 0.125714 | 0.0261174 | 1.48E-06 |  | -0.0456 | 0.0754 | 0.5457 | 23.16900638 |
| mumps | rs5995517 | C | T | 0.4195 | 0.106013 | 0.0226246 | 2.79E-06 |  | -0.0212 | 0.0689 | 0.7583 | 21.95616216 |
| mumps | rs6006421 | A | G | 0.1322 | -0.138601 | 0.0295874 | 2.81E-06 |  | -0.0952 | 0.1008 | 0.3449 | 21.94416809 |
| mumps | rs6082722 | A | G | 0.008494 | 0.417034 | 0.0913098 | 4.94E-06 |  | 0.5633 | 0.3791 | 0.1374 | 20.85970413 |
| mumps | rs62556523 | A | G | 0.04687 | 0.253426 | 0.0521249 | 1.16E-06 |  | -0.2585 | 0.1683 | 0.1245 | 23.63806204 |
| mumps | rs74631778 | G | T | 0.1344 | -0.171312 | 0.0365695 | 2.81E-06 |  | 0.1748 | 0.0989 | 0.0771898 | 21.94509806 |
| mumps | rs75675564 | A | G | 0.05485 | 0.190796 | 0.0414533 | 4.17E-06 |  | 0.019 | 0.1503 | 0.8996 | 21.18460215 |
| mumps | rs7718658 | T | C | 0.276 | 0.11229 | 0.0243882 | 4.14E-06 |  | -0.095 | 0.0758 | 0.2103 | 21.199356 |
| mumps | rs7789987 | C | T | 0.6645 | 0.111305 | 0.0240291 | 3.62E-06 |  | -0.0678 | 0.0719 | 0.3456 | 21.45627557 |
| mumps | rs9514622 | A | G | 0.3124 | 0.109803 | 0.023249 | 2.32E-06 |  | -0.0687 | 0.0737 | 0.3512 | 22.30590544 |
| poliovirus | rs10889496 | G | A | 0.4694 | -0.11761 | 0.0242264 | 1.21E-06 |  | 0.0842 | 0.0944 | 0.3721 | 23.56734898 |
| poliovirus | rs1122530 | G | A | 0.1235 | 0.147371 | 0.0316843 | 3.30E-06 |  | 0.0671 | 0.1426 | 0.637699 | 21.63395029 |
| poliovirus | rs113089273 | T | C | 0.04232 | 0.25776 | 0.0551957 | 3.01E-06 |  | -0.1943 | 0.2314 | 0.4012 | 21.80823685 |
| poliovirus | rs114531477 | A | G | 0.03816 | 0.324092 | 0.069409 | 3.02E-06 |  | -0.2966 | 0.2424 | 0.2212 | 21.80243734 |
| poliovirus | rs11551684 | T | C | 0.1705 | -0.147887 | 0.0301716 | 9.51E-07 |  | -0.0039 | 0.1255 | 0.9752 | 24.02499551 |
| poliovirus | rs117157710 | G | A | 0.04293 | 0.271495 | 0.0585753 | 3.57E-06 |  | -0.3175 | 0.229 | 0.1656 | 21.48298516 |
| poliovirus | rs117265989 | C | T | 0.02144 | -0.467117 | 0.0629887 | 1.21E-13 |  | 0.7475 | 0.3279 | 0.0226298 | 54.99536081 |
| poliovirus | rs12332680 | G | A | 0.3323 | -0.114082 | 0.0242868 | 2.64E-06 |  | 0.1419 | 0.1 | 0.1557 | 22.06447812 |
| poliovirus | rs12449010 | T | C | 0.08772 | -0.207737 | 0.043937 | 2.27E-06 |  | -0.0595 | 0.1658 | 0.7199 | 22.35460039 |
| poliovirus | rs12482260 | G | A | 0.0153 | 0.446356 | 0.085082 | 1.55E-07 |  | 0.1261 | 0.386 | 0.7438 | 27.52246789 |
| poliovirus | rs146122326 | A | G | 0.03015 | 0.446578 | 0.0820452 | 5.24E-08 |  | -0.2041 | 0.2804 | 0.4666 | 29.62704197 |
| poliovirus | rs2110922 | A | C | 0.4574 | 0.106743 | 0.0230252 | 3.55E-06 |  | -0.0225 | 0.0942 | 0.8114 | 21.49176021 |
| poliovirus | rs2288369 | A | G | 0.6777 | 0.127081 | 0.0257983 | 8.40E-07 |  | -0.0215 | 0.1 | 0.8301 | 24.26493151 |
| poliovirus | rs2330761 | C | A | 0.4654 | 0.108058 | 0.0223539 | 1.34E-06 |  | 0.1056 | 0.0938 | 0.26 | 23.3672305 |
| poliovirus | rs30356 | C | T | 0.2755 | -0.12131 | 0.0236614 | 2.95E-07 |  | 0.0321 | 0.1052 | 0.7602 | 26.28526323 |
| poliovirus | rs35312516 | T | C | 0.04687 | 0.206088 | 0.0449243 | 4.49E-06 |  | -0.2293 | 0.2195 | 0.2962 | 21.04470158 |
| poliovirus | rs3761121 | C | T | 0.09098 | 0.24109 | 0.0345589 | 3.03E-12 |  | -0.1128 | 0.1649 | 0.494 | 48.66744792 |
| poliovirus | rs3844166 | A | G | 0.06306 | -0.234127 | 0.0512792 | 4.98E-06 |  | 0.1698 | 0.1945 | 0.3825 | 20.84589367 |
| poliovirus | rs4149909 | G | A | 0.03157 | 0.363112 | 0.0689955 | 1.42E-07 |  | -0.6126 | 0.265 | 0.0207802 | 27.69744257 |
| poliovirus | rs4150221 | C | T | 0.3194 | -0.123268 | 0.0253274 | 1.13E-06 |  | -0.0161 | 0.0998 | 0.872 | 23.68751382 |
| poliovirus | rs4984741 | A | G | 0.1707 | 0.118358 | 0.0257405 | 4.26E-06 |  | 0.168 | 0.1258 | 0.182 | 21.14274045 |
| poliovirus | rs56064154 | A | C | 0.2182 | -0.156413 | 0.0323743 | 1.36E-06 |  | -0.1185 | 0.1131 | 0.2951 | 23.34236835 |
| poliovirus | rs56168929 | A | G | 0.2824 | 0.125714 | 0.0261174 | 1.48E-06 |  | -0.0888 | 0.1039 | 0.3926 | 23.16900638 |
| poliovirus | rs5995517 | C | T | 0.4196 | 0.106013 | 0.0226246 | 2.79E-06 |  | 0.0062 | 0.0949 | 0.9477 | 21.95616216 |
| poliovirus | rs6006421 | A | G | 0.1322 | -0.138601 | 0.0295874 | 2.81E-06 |  | 0.1899 | 0.139 | 0.1718 | 21.94416809 |
| poliovirus | rs6082722 | A | G | 0.008509 | 0.417034 | 0.0913098 | 4.94E-06 |  | -0.2346 | 0.5267 | 0.6561 | 20.85970413 |
| poliovirus | rs62556523 | A | G | 0.04685 | 0.253426 | 0.0521249 | 1.16E-06 |  | -0.0246 | 0.2324 | 0.9156 | 23.63806204 |
| poliovirus | rs74631778 | G | T | 0.1345 | -0.171312 | 0.0365695 | 2.81E-06 |  | -0.0314 | 0.1361 | 0.8176 | 21.94509806 |
| poliovirus | rs75675564 | A | G | 0.05487 | 0.190796 | 0.0414533 | 4.17E-06 |  | 0.0137 | 0.2082 | 0.9475 | 21.18460215 |
| poliovirus | rs7718658 | T | C | 0.2761 | 0.11229 | 0.0243882 | 4.14E-06 |  | -0.033 | 0.105 | 0.7535 | 21.199356 |
| poliovirus | rs7789987 | C | T | 0.6645 | 0.111305 | 0.0240291 | 3.62E-06 |  | -0.1426 | 0.0992 | 0.1507 | 21.45627557 |
| poliovirus | rs9514622 | A | G | 0.3122 | 0.109803 | 0.023249 | 2.32E-06 |  | 0.0288 | 0.1015 | 0.776599 | 22.30590544 |
| Rubella | rs10889496 | G | A | 0.4693 | -0.11761 | 0.0242264 | 1.21E-06 |  | 0.0027 | 0.0614 | 0.9653 | 23.56734898 |
| Rubella | rs1122530 | G | A | 0.1235 | 0.147371 | 0.0316843 | 3.30E-06 |  | 0.0131 | 0.0929 | 0.8881 | 21.63395029 |
| Rubella | rs113089273 | T | C | 0.0424 | 0.25776 | 0.0551957 | 3.01E-06 |  | 0.2797 | 0.1504 | 0.0628999 | 21.80823685 |
| Rubella | rs114531477 | A | G | 0.03813 | 0.324092 | 0.069409 | 3.02E-06 |  | -0.0862 | 0.1586 | 0.5867 | 21.80243734 |
| Rubella | rs11551684 | T | C | 0.1706 | -0.147887 | 0.0301716 | 9.51E-07 |  | -0.0372 | 0.0817 | 0.649 | 24.02499551 |
| Rubella | rs117157710 | G | A | 0.04296 | 0.271495 | 0.0585753 | 3.57E-06 |  | -0.2639 | 0.1496 | 0.0776909 | 21.48298516 |
| Rubella | rs117265989 | C | T | 0.02141 | -0.467117 | 0.0629887 | 1.21E-13 |  | 0.3449 | 0.2138 | 0.1067 | 54.99536081 |
| Rubella | rs12332680 | G | A | 0.3324 | -0.114082 | 0.0242868 | 2.64E-06 |  | 0.1248 | 0.0648 | 0.0539002 | 22.06447812 |
| Rubella | rs12449010 | T | C | 0.08765 | -0.207737 | 0.043937 | 2.27E-06 |  | 0.1093 | 0.1079 | 0.3113 | 22.35460039 |
| Rubella | rs12482260 | G | A | 0.01532 | 0.446356 | 0.085082 | 1.55E-07 |  | -0.3424 | 0.2508 | 0.1721 | 27.52246789 |
| Rubella | rs146122326 | A | G | 0.03015 | 0.446578 | 0.0820452 | 5.24E-08 |  | -0.0384 | 0.1819 | 0.833 | 29.62704197 |
| Rubella | rs2110922 | A | C | 0.4571 | 0.106743 | 0.0230252 | 3.55E-06 |  | -0.0525 | 0.0613 | 0.3914 | 21.49176021 |
| Rubella | rs2288369 | A | G | 0.6776 | 0.127081 | 0.0257983 | 8.40E-07 |  | -0.0151 | 0.0652 | 0.8173 | 24.26493151 |
| Rubella | rs2330761 | C | A | 0.4654 | 0.108058 | 0.0223539 | 1.34E-06 |  | 0.0201 | 0.061 | 0.742299 | 23.3672305 |
| Rubella | rs30356 | C | T | 0.2755 | -0.12131 | 0.0236614 | 2.95E-07 |  | 0.0487 | 0.0683 | 0.4761 | 26.28526323 |
| Rubella | rs35312516 | T | C | 0.04685 | 0.206088 | 0.0449243 | 4.49E-06 |  | -0.0089 | 0.1429 | 0.9505 | 21.04470158 |
| Rubella | rs3761121 | C | T | 0.09089 | 0.24109 | 0.0345589 | 3.03E-12 |  | 0.1158 | 0.1071 | 0.2795 | 48.66744792 |
| Rubella | rs3844166 | A | G | 0.06308 | -0.234127 | 0.0512792 | 4.98E-06 |  | 0.0523 | 0.1252 | 0.6762 | 20.84589367 |
| Rubella | rs4149909 | G | A | 0.03155 | 0.363112 | 0.0689955 | 1.42E-07 |  | 0.297 | 0.1757 | 0.0909997 | 27.69744257 |
| Rubella | rs4150221 | C | T | 0.3195 | -0.123268 | 0.0253274 | 1.13E-06 |  | -0.0531 | 0.065 | 0.4142 | 23.68751382 |
| Rubella | rs4984741 | A | G | 0.1708 | 0.118358 | 0.0257405 | 4.26E-06 |  | 0.0542 | 0.0818 | 0.5078 | 21.14274045 |
| Rubella | rs56064154 | A | C | 0.2183 | -0.156413 | 0.0323743 | 1.36E-06 |  | 0.0907 | 0.0738 | 0.2189 | 23.34236835 |
| Rubella | rs56168929 | A | G | 0.2825 | 0.125714 | 0.0261174 | 1.48E-06 |  | -0.0807 | 0.0677 | 0.2331 | 23.16900638 |
| Rubella | rs5995517 | C | T | 0.4198 | 0.106013 | 0.0226246 | 2.79E-06 |  | -0.0977 | 0.0619 | 0.1143 | 21.95616216 |
| Rubella | rs6006421 | A | G | 0.1323 | -0.138601 | 0.0295874 | 2.81E-06 |  | -0.106 | 0.0903 | 0.2402 | 21.94416809 |
| Rubella | rs6082722 | A | G | 0.00849 | 0.417034 | 0.0913098 | 4.94E-06 |  | -0.6078 | 0.3432 | 0.0765808 | 20.85970413 |
| Rubella | rs62556523 | A | G | 0.04681 | 0.253426 | 0.0521249 | 1.16E-06 |  | 0.0662 | 0.1502 | 0.6591 | 23.63806204 |
| Rubella | rs74631778 | G | T | 0.1344 | -0.171312 | 0.0365695 | 2.81E-06 |  | -0.0642 | 0.0894 | 0.472799 | 21.94509806 |
| Rubella | rs75675564 | A | G | 0.05493 | 0.190796 | 0.0414533 | 4.17E-06 |  | 0.201 | 0.1341 | 0.1339 | 21.18460215 |
| Rubella | rs7718658 | T | C | 0.2761 | 0.11229 | 0.0243882 | 4.14E-06 |  | 0.046 | 0.0682 | 0.4998 | 21.199356 |
| Rubella | rs7789987 | C | T | 0.6645 | 0.111305 | 0.0240291 | 3.62E-06 |  | -0.08 | 0.0644 | 0.2139 | 21.45627557 |
| Rubella | rs9514622 | A | G | 0.3124 | 0.109803 | 0.023249 | 2.32E-06 |  | 0.0308 | 0.066 | 0.6402 | 22.30590544 |
| Herpes zoster | rs10889496 | G | A | 0.4694 | -0.11761 | 0.0242264 | 1.21E-06 |  | 0.0176 | 0.0317 | 0.5772 | 23.56734898 |
| Herpes zoster | rs1122530 | G | A | 0.1236 | 0.147371 | 0.0316843 | 3.30E-06 |  | 0.0902 | 0.0478 | 0.0591494 | 21.63395029 |
| Herpes zoster | rs113089273 | T | C | 0.04234 | 0.25776 | 0.0551957 | 3.01E-06 |  | -0.0698 | 0.0777 | 0.3694 | 21.80823685 |
| Herpes zoster | rs114531477 | A | G | 0.03815 | 0.324092 | 0.069409 | 3.02E-06 |  | 0.028 | 0.0825 | 0.734401 | 21.80243734 |
| Herpes zoster | rs11551684 | T | C | 0.1706 | -0.147887 | 0.0301716 | 9.51E-07 |  | -0.0047 | 0.0421 | 0.9114 | 24.02499551 |
| Herpes zoster | rs117157710 | G | A | 0.04297 | 0.271495 | 0.0585753 | 3.57E-06 |  | -0.0491 | 0.0774 | 0.526 | 21.48298516 |
| Herpes zoster | rs117265989 | C | T | 0.02141 | -0.467117 | 0.0629887 | 1.21E-13 |  | 0.071 | 0.1093 | 0.515901 | 54.99536081 |
| Herpes zoster | rs12332680 | G | A | 0.3323 | -0.114082 | 0.0242868 | 2.64E-06 |  | -0.0113 | 0.0335 | 0.7365 | 22.06447812 |
| Herpes zoster | rs12449010 | T | C | 0.08769 | -0.207737 | 0.043937 | 2.27E-06 |  | 0.078 | 0.0555 | 0.1597 | 22.35460039 |
| Herpes zoster | rs12482260 | G | A | 0.01531 | 0.446356 | 0.085082 | 1.55E-07 |  | -0.1642 | 0.1282 | 0.2003 | 27.52246789 |
| Herpes zoster | rs146122326 | A | G | 0.03017 | 0.446578 | 0.0820452 | 5.24E-08 |  | 0.0874 | 0.0938 | 0.3513 | 29.62704197 |
| Herpes zoster | rs2110922 | A | C | 0.4573 | 0.106743 | 0.0230252 | 3.55E-06 |  | 0.0403 | 0.0316 | 0.2023 | 21.49176021 |
| Herpes zoster | rs2288369 | A | G | 0.6776 | 0.127081 | 0.0257983 | 8.40E-07 |  | -0.0379 | 0.0336 | 0.2583 | 24.26493151 |
| Herpes zoster | rs2330761 | C | A | 0.4655 | 0.108058 | 0.0223539 | 1.34E-06 |  | 0.052 | 0.0314 | 0.09794 | 23.3672305 |
| Herpes zoster | rs30356 | C | T | 0.2755 | -0.12131 | 0.0236614 | 2.95E-07 |  | 0.0024 | 0.0352 | 0.9459 | 26.28526323 |
| Herpes zoster | rs35312516 | T | C | 0.04683 | 0.206088 | 0.0449243 | 4.49E-06 |  | -0.0419 | 0.0736 | 0.569399 | 21.04470158 |
| Herpes zoster | rs3761121 | C | T | 0.09088 | 0.24109 | 0.0345589 | 3.03E-12 |  | 0.0222 | 0.0551 | 0.687401 | 48.66744792 |
| Herpes zoster | rs3844166 | A | G | 0.06306 | -0.234127 | 0.0512792 | 4.98E-06 |  | -0.006 | 0.0648 | 0.9262 | 20.84589367 |
| Herpes zoster | rs4149909 | G | A | 0.03147 | 0.363112 | 0.0689955 | 1.42E-07 |  | -0.1784 | 0.09 | 0.0474297 | 27.69744257 |
| Herpes zoster | rs4150221 | C | T | 0.3194 | -0.123268 | 0.0253274 | 1.13E-06 |  | -0.0425 | 0.0335 | 0.205 | 23.68751382 |
| Herpes zoster | rs4984741 | A | G | 0.1707 | 0.118358 | 0.0257405 | 4.26E-06 |  | 0.0078 | 0.042 | 0.8523 | 21.14274045 |
| Herpes zoster | rs56064154 | A | C | 0.2182 | -0.156413 | 0.0323743 | 1.36E-06 |  | 0.002 | 0.0379 | 0.9575 | 23.34236835 |
| Herpes zoster | rs56168929 | A | G | 0.2826 | 0.125714 | 0.0261174 | 1.48E-06 |  | 0.0154 | 0.0348 | 0.658999 | 23.16900638 |
| Herpes zoster | rs5995517 | C | T | 0.4198 | 0.106013 | 0.0226246 | 2.79E-06 |  | -0.0175 | 0.0318 | 0.583201 | 21.95616216 |
| Herpes zoster | rs6006421 | A | G | 0.1323 | -0.138601 | 0.0295874 | 2.81E-06 |  | -0.0782 | 0.0463 | 0.0915293 | 21.94416809 |
| Herpes zoster | rs6082722 | A | G | 0.008519 | 0.417034 | 0.0913098 | 4.94E-06 |  | 0.2014 | 0.1739 | 0.2466 | 20.85970413 |
| Herpes zoster | rs62556523 | A | G | 0.04681 | 0.253426 | 0.0521249 | 1.16E-06 |  | 0.0362 | 0.078 | 0.6427 | 23.63806204 |
| Herpes zoster | rs74631778 | G | T | 0.1345 | -0.171312 | 0.0365695 | 2.81E-06 |  | 0.0443 | 0.0457 | 0.3316 | 21.94509806 |
| Herpes zoster | rs75675564 | A | G | 0.05488 | 0.190796 | 0.0414533 | 4.17E-06 |  | -0.0413 | 0.0691 | 0.550201 | 21.18460215 |
| Herpes zoster | rs7718658 | T | C | 0.276 | 0.11229 | 0.0243882 | 4.14E-06 |  | -0.0368 | 0.0351 | 0.2943 | 21.199356 |
| Herpes zoster | rs7789987 | C | T | 0.6645 | 0.111305 | 0.0240291 | 3.62E-06 |  | -0.0205 | 0.0333 | 0.5373 | 21.45627557 |
| Herpes zoster | rs9514622 | A | G | 0.3124 | 0.109803 | 0.023249 | 2.32E-06 |  | -0.0221 | 0.034 | 0.5157 | 22.30590544 |

**Additional file 6: Table S3**. Single SNP analysis of the association of all-glioma on viral infection

|  |  |  |  |  | **Glioma** | | |  | **Virus infection** | | |  |
| --- | --- | --- | --- | --- | --- | --- | --- | --- | --- | --- | --- | --- |
| **Outcome** | **SNP** | **Effect allele** | **Other allele** | **Effect allele frequency** | **Beta** | **Standard error** | **P-value** |  | **Beta *** | **Standard error *** | **P-value *** | **F-statistics** |
| Cytomegaloviral disease | rs10060827 | T | G | 0.3318 | -0.094712 | 0.0197935 | 1.71E-06 |  | -0.0325 | 0.0922 | 0.7247 | 22.89627456 |
| Cytomegaloviral disease | rs10889496 | G | A | 0.4695 | -0.0932824 | 0.019739 | 2.29E-06 |  | 0.0122 | 0.087 | 0.8881 | 22.33310606 |
| Cytomegaloviral disease | rs10981985 | A | G | 0.1502 | -0.138288 | 0.0293504 | 2.46E-06 |  | 0.0197 | 0.1208 | 0.8705 | 22.19938497 |
| Cytomegaloviral disease | rs11002922 | T | C | 0.3116 | 0.0954619 | 0.0202804 | 2.51E-06 |  | 0.1124 | 0.0929 | 0.2265 | 22.15680395 |
| Cytomegaloviral disease | rs111384268 | A | G | 0.1133 | 0.15554 | 0.0312497 | 6.45E-07 |  | 0.1325 | 0.1377 | 0.3359 | 24.77379185 |
| Cytomegaloviral disease | rs11143886 | A | C | 0.04455 | 0.185149 | 0.0403842 | 4.55E-06 |  | -0.1325 | 0.2079 | 0.5239 | 21.01937379 |
| Cytomegaloviral disease | rs11233250 | T | C | 0.119 | -0.135681 | 0.0294397 | 4.05E-06 |  | 0.0397 | 0.1327 | 0.7651 | 21.2408216 |
| Cytomegaloviral disease | rs11576512 | A | G | 0.1714 | -0.117617 | 0.0241214 | 1.08E-06 |  | 0.0698 | 0.1166 | 0.549301 | 23.77580196 |
| Cytomegaloviral disease | rs117135784 | A | G | 0.02854 | 0.349187 | 0.0663151 | 1.40E-07 |  | 0.123 | 0.2593 | 0.635199 | 27.72625901 |
| Cytomegaloviral disease | rs12449010 | T | C | 0.08774 | -0.174121 | 0.0361339 | 1.44E-06 |  | -0.112 | 0.1516 | 0.4598 | 23.22055677 |
| Cytomegaloviral disease | rs12482260 | G | A | 0.01528 | 0.333816 | 0.0690714 | 1.35E-06 |  | -0.1483 | 0.3523 | 0.673701 | 23.35703869 |
| Cytomegaloviral disease | rs12573103 | G | A | 0.6113 | -0.0905768 | 0.0187059 | 1.28E-06 |  | 0.0309 | 0.0887 | 0.727601 | 23.44643025 |
| Cytomegaloviral disease | rs12602989 | T | C | 0.1929 | 0.117641 | 0.0236795 | 6.76E-07 |  | -0.0256 | 0.1092 | 0.8146 | 24.68154599 |
| Cytomegaloviral disease | rs12924608 | C | T | 0.3768 | 0.0927112 | 0.0189619 | 1.01E-06 |  | -0.0908 | 0.0895 | 0.3099 | 23.90565794 |
| Cytomegaloviral disease | rs12995271 | C | T | 0.2139 | -0.0982709 | 0.0214526 | 4.63E-06 |  | 0.2062 | 0.1048 | 0.0491904 | 20.98408307 |
| Cytomegaloviral disease | rs13267723 | A | G | 0.199 | -0.107534 | 0.0217893 | 8.01E-07 |  | -0.1114 | 0.1085 | 0.3047 | 24.35594837 |
| Cytomegaloviral disease | rs1476275 | T | C | 0.02037 | 0.26341 | 0.0547143 | 1.48E-06 |  | -0.2802 | 0.3086 | 0.3639 | 23.17729892 |
| Cytomegaloviral disease | rs1835841 | T | C | 0.4163 | -0.0886655 | 0.0184343 | 1.51E-06 |  | -0.0105 | 0.0878 | 0.9049 | 23.13428242 |
| Cytomegaloviral disease | rs2047469 | T | C | 0.2822 | 0.114101 | 0.0232276 | 9.00E-07 |  | 0.0907 | 0.0966 | 0.3477 | 24.13071689 |
| Cytomegaloviral disease | rs2241545 | G | A | 0.3281 | -0.0968023 | 0.0210325 | 4.17E-06 |  | 0.0108 | 0.092 | 0.9069 | 21.18310199 |
| Cytomegaloviral disease | rs2293607 | C | T | 0.2729 | -0.0989059 | 0.0214803 | 4.13E-06 |  | 0.0324 | 0.0968 | 0.7376 | 21.20135965 |
| Cytomegaloviral disease | rs2330764 | C | T | 0.4581 | 0.0890894 | 0.0183096 | 1.14E-06 |  | -0.1794 | 0.0868 | 0.0386403 | 23.67523807 |
| Cytomegaloviral disease | rs34126012 | A | G | 0.3217 | -0.124931 | 0.0270073 | 3.73E-06 |  | -0.0259 | 0.0923 | 0.7788 | 21.39824202 |
| Cytomegaloviral disease | rs4149909 | G | A | 0.03154 | 0.276204 | 0.0556336 | 6.88E-07 |  | -0.5091 | 0.2468 | 0.0391598 | 24.64822219 |
| Cytomegaloviral disease | rs4150221 | C | T | 0.3194 | -0.0985601 | 0.0206477 | 1.81E-06 |  | -0.0436 | 0.0923 | 0.6363 | 22.78551809 |
| Cytomegaloviral disease | rs4443239 | G | T | 0.1667 | -0.0966618 | 0.0210859 | 4.56E-06 |  | 0.1291 | 0.1162 | 0.2669 | 21.01481017 |
| Cytomegaloviral disease | rs4606014 | T | C | 0.468 | 0.0841955 | 0.0181002 | 3.29E-06 |  | 0.0519 | 0.0865 | 0.5485 | 21.63769593 |
| Cytomegaloviral disease | rs4754296 | G | T | 0.2235 | -0.128209 | 0.0267998 | 1.72E-06 |  | 0.0199 | 0.104 | 0.8479 | 22.88621216 |
| Cytomegaloviral disease | rs4938513 | C | T | 0.5835 | -0.0923986 | 0.0190957 | 1.31E-06 |  | 0.1285 | 0.0879 | 0.1438 | 23.4131375 |
| Cytomegaloviral disease | rs4975620 | C | T | 0.348 | 0.0901892 | 0.0196214 | 4.30E-06 |  | 0.0613 | 0.0906 | 0.4986 | 21.12754749 |
| Cytomegaloviral disease | rs56795824 | C | T | 0.02746 | 0.375227 | 0.0811956 | 3.81E-06 |  | 0.4792 | 0.2658 | 0.0714595 | 21.35616027 |
| Cytomegaloviral disease | rs5756908 | T | C | 0.5576 | -0.0853171 | 0.018151 | 2.60E-06 |  | -0.0667 | 0.0869 | 0.4429 | 22.09383246 |
| Cytomegaloviral disease | rs61364850 | G | A | 0.02451 | -0.193051 | 0.0419428 | 4.17E-06 |  | -0.1514 | 0.2794 | 0.587801 | 21.18503921 |
| Cytomegaloviral disease | rs62137639 | G | A | 0.02132 | 0.268696 | 0.0551093 | 1.08E-06 |  | 0.0334 | 0.2999 | 0.9112 | 23.77237723 |
| Cytomegaloviral disease | rs62556523 | A | G | 0.04691 | 0.19645 | 0.0423733 | 3.55E-06 |  | 0.4367 | 0.2126 | 0.0399402 | 21.49411116 |
| Cytomegaloviral disease | rs7679589 | A | C | 0.025 | -0.251704 | 0.0542147 | 3.44E-06 |  | -0.0586 | 0.2773 | 0.8327 | 21.55490518 |
| Cytomegaloviral disease | rs78341235 | C | T | 0.3243 | 0.138307 | 0.0292407 | 2.25E-06 |  | 0.0083 | 0.0925 | 0.9284 | 22.37241116 |
| Cytomegaloviral disease | rs926771 | T | C | 0.07873 | -0.161931 | 0.0332555 | 1.12E-06 |  | 0.0244 | 0.1604 | 0.8792 | 23.71008069 |
| Cytomegaloviral disease | rs9909962 | C | T | 0.09664 | 0.147003 | 0.0311733 | 2.41E-06 |  | 0.0698 | 0.1463 | 0.6334 | 22.23754488 |
| COVID-19 hospitalized | rs10060827 | T | G | 0.3002 | -0.094712 | 0.0197935 | 1.71E-06 |  | 0.019073 | 0.01097 | 0.08209 | 22.89627456 |
| COVID-19 hospitalized | rs10889496 | G | A | 0.3638 | -0.0932824 | 0.019739 | 2.29E-06 |  | 0.025603 | 0.010633 | 0.0160398 | 22.33310606 |
| COVID-19 hospitalized | rs10981985 | A | G | 0.1354 | -0.138288 | 0.0293504 | 2.46E-06 |  | -0.016017 | 0.015443 | 0.2997 | 22.19938497 |
| COVID-19 hospitalized | rs11002922 | T | C | 0.2968 | 0.0954619 | 0.0202804 | 2.51E-06 |  | 0.015391 | 0.010869 | 0.1568 | 22.15680395 |
| COVID-19 hospitalized | rs111384268 | A | G | 0.1043 | 0.15554 | 0.0312497 | 6.45E-07 |  | 0.0029499 | 0.016875 | 0.8612 | 24.77379185 |
| COVID-19 hospitalized | rs11143886 | A | C | 0.05815 | 0.185149 | 0.0403842 | 4.55E-06 |  | 0.011281 | 0.020929 | 0.589899 | 21.01937379 |
| COVID-19 hospitalized | rs11233250 | T | C | 0.1273 | -0.135681 | 0.0294397 | 4.05E-06 |  | 0.028594 | 0.015073 | 0.0578203 | 21.2408216 |
| COVID-19 hospitalized | rs117135784 | A | G | 0.03451 | 0.349187 | 0.0663151 | 1.40E-07 |  | 0.0027973 | 0.035126 | 0.9365 | 27.72625901 |
| COVID-19 hospitalized | rs12449010 | T | C | 0.08424 | -0.174121 | 0.0361339 | 1.44E-06 |  | 0.0018507 | 0.018499 | 0.9203 | 23.22055677 |
| COVID-19 hospitalized | rs12482260 | G | A | 0.02858 | 0.333816 | 0.0690714 | 1.35E-06 |  | 0.027763 | 0.033703 | 0.4101 | 23.35703869 |
| COVID-19 hospitalized | rs12573103 | G | A | 0.5266 | -0.0905768 | 0.0187059 | 1.28E-06 |  | -0.0018761 | 0.0097049 | 0.8467 | 23.44643025 |
| COVID-19 hospitalized | rs12602989 | T | C | 0.1887 | 0.117641 | 0.0236795 | 6.76E-07 |  | 0.013065 | 0.01236 | 0.2905 | 24.68154599 |
| COVID-19 hospitalized | rs12924608 | C | T | 0.3905 | 0.0927112 | 0.0189619 | 1.01E-06 |  | 0.0037061 | 0.01036 | 0.7205 | 23.90565794 |
| COVID-19 hospitalized | rs12995271 | C | T | 0.2302 | -0.0982709 | 0.0214526 | 4.63E-06 |  | 0.0037644 | 0.011148 | 0.7356 | 20.98408307 |
| COVID-19 hospitalized | rs13267723 | A | G | 0.2233 | -0.107534 | 0.0217893 | 8.01E-07 |  | 0.0042551 | 0.012663 | 0.736801 | 24.35594837 |
| COVID-19 hospitalized | rs1476275 | T | C | 0.0422 | 0.26341 | 0.0547143 | 1.48E-06 |  | -0.011915 | 0.026468 | 0.6526 | 23.17729892 |
| COVID-19 hospitalized | rs1835841 | T | C | 0.4972 | -0.0886655 | 0.0184343 | 1.51E-06 |  | 0.00050509 | 0.0095219 | 0.9577 | 23.13428242 |
| COVID-19 hospitalized | rs2047469 | T | C | 0.2201 | 0.114101 | 0.0232276 | 9.00E-07 |  | 0.0016347 | 0.011829 | 0.8901 | 24.13071689 |
| COVID-19 hospitalized | rs2241545 | G | A | 0.2764 | -0.0968023 | 0.0210325 | 4.17E-06 |  | -0.0065221 | 0.010875 | 0.5487 | 21.18310199 |
| COVID-19 hospitalized | rs2293607 | C | T | 0.2465 | -0.0989059 | 0.0214803 | 4.13E-06 |  | 0.003961 | 0.010975 | 0.718199 | 21.20135965 |
| COVID-19 hospitalized | rs2330764 | C | T | 0.48 | 0.0890894 | 0.0183096 | 1.14E-06 |  | -0.025124 | 0.01008 | 0.01269 | 23.67523807 |
| COVID-19 hospitalized | rs34126012 | A | G | 0.3234 | -0.124931 | 0.0270073 | 3.73E-06 |  | 0.012814 | 0.010928 | 0.2409 | 21.39824202 |
| COVID-19 hospitalized | rs4149909 | G | A | 0.03957 | 0.276204 | 0.0556336 | 6.88E-07 |  | 0.0035869 | 0.027459 | 0.8961 | 24.64822219 |
| COVID-19 hospitalized | rs4150221 | C | T | 0.2813 | -0.0985601 | 0.0206477 | 1.81E-06 |  | -0.0070457 | 0.010631 | 0.5075 | 22.78551809 |
| COVID-19 hospitalized | rs4443239 | G | T | 0.2308 | -0.0966618 | 0.0210859 | 4.56E-06 |  | -0.013655 | 0.011757 | 0.2455 | 21.01481017 |
| COVID-19 hospitalized | rs4606014 | T | C | 0.4898 | 0.0841955 | 0.0181002 | 3.29E-06 |  | -0.0030481 | 0.0097096 | 0.7536 | 21.63769593 |
| COVID-19 hospitalized | rs4754296 | G | T | 0.1619 | -0.128209 | 0.0267998 | 1.72E-06 |  | 0.0021766 | 0.013951 | 0.876 | 22.88621216 |
| COVID-19 hospitalized | rs4809332 | G | A | 0.5864 | -0.0936529 | 0.018632 | 5.00E-07 |  | 0.0059049 | 0.010856 | 0.5865 | 25.26524471 |
| COVID-19 hospitalized | rs4938513 | C | T | 0.5555 | -0.0923986 | 0.0190957 | 1.31E-06 |  | -0.0073559 | 0.010179 | 0.4699 | 23.4131375 |
| COVID-19 hospitalized | rs4975620 | C | T | 0.3387 | 0.0901892 | 0.0196214 | 4.30E-06 |  | 0.0039796 | 0.010597 | 0.707301 | 21.12754749 |
| COVID-19 hospitalized | rs56795824 | C | T | 0.03034 | 0.375227 | 0.0811956 | 3.81E-06 |  | 0.0063659 | 0.038153 | 0.8675 | 21.35616027 |
| COVID-19 hospitalized | rs5756908 | T | C | 0.5041 | -0.0853171 | 0.018151 | 2.60E-06 |  | 0.0099172 | 0.0095063 | 0.2968 | 22.09383246 |
| COVID-19 hospitalized | rs61364850 | G | A | 0.04953 | -0.193051 | 0.0419428 | 4.17E-06 |  | 0.0092848 | 0.022615 | 0.6814 | 21.18503921 |
| COVID-19 hospitalized | rs62137639 | G | A | 0.03798 | 0.268696 | 0.0551093 | 1.08E-06 |  | 0.013541 | 0.026782 | 0.6131 | 23.77237723 |
| COVID-19 hospitalized | rs62556523 | A | G | 0.05471 | 0.19645 | 0.0423733 | 3.55E-06 |  | -0.0025997 | 0.026024 | 0.9204 | 21.49411116 |
| COVID-19 hospitalized | rs72752071 | A | G | 0.1079 | 0.139856 | 0.030303 | 3.93E-06 |  | 0.010671 | 0.016781 | 0.5248 | 21.3005567 |
| COVID-19 hospitalized | rs7679589 | A | C | 0.03348 | -0.251704 | 0.0542147 | 3.44E-06 |  | -0.025169 | 0.028997 | 0.3854 | 21.55490518 |
| COVID-19 hospitalized | rs926771 | T | C | 0.08755 | -0.161931 | 0.0332555 | 1.12E-06 |  | 0.01349 | 0.01736 | 0.4371 | 23.71008069 |
| COVID-19 hospitalized | rs9909962 | C | T | 0.1017 | 0.147003 | 0.0311733 | 2.41E-06 |  | -0.0085783 | 0.015843 | 0.5882 | 22.23754488 |
| Infectious mononucleosis | rs10060827 | T | G | 0.3318 | -0.094712 | 0.0197935 | 1.71E-06 |  | -0.0106 | 0.0436 | 0.8078 | 22.89627456 |
| Infectious mononucleosis | rs10889496 | G | A | 0.4694 | -0.0932824 | 0.019739 | 2.29E-06 |  | -0.1004 | 0.0413 | 0.0150501 | 22.33310606 |
| Infectious mononucleosis | rs10981985 | A | G | 0.1502 | -0.138288 | 0.0293504 | 2.46E-06 |  | -0.0379 | 0.0574 | 0.5099 | 22.19938497 |
| Infectious mononucleosis | rs11002922 | T | C | 0.3115 | 0.0954619 | 0.0202804 | 2.51E-06 |  | -0.0192 | 0.044 | 0.6624 | 22.15680395 |
| Infectious mononucleosis | rs111384268 | A | G | 0.1133 | 0.15554 | 0.0312497 | 6.45E-07 |  | 0.0994 | 0.0657 | 0.1303 | 24.77379185 |
| Infectious mononucleosis | rs11143886 | A | C | 0.04454 | 0.185149 | 0.0403842 | 4.55E-06 |  | -0.072 | 0.0992 | 0.4682 | 21.01937379 |
| Infectious mononucleosis | rs11233250 | T | C | 0.119 | -0.135681 | 0.0294397 | 4.05E-06 |  | -0.0288 | 0.0634 | 0.6503 | 21.2408216 |
| Infectious mononucleosis | rs11576512 | A | G | 0.1714 | -0.117617 | 0.0241214 | 1.08E-06 |  | -0.0077 | 0.0552 | 0.8895 | 23.77580196 |
| Infectious mononucleosis | rs117135784 | A | G | 0.02855 | 0.349187 | 0.0663151 | 1.40E-07 |  | 0.0417 | 0.1225 | 0.7334 | 27.72625901 |
| Infectious mononucleosis | rs12449010 | T | C | 0.08777 | -0.174121 | 0.0361339 | 1.44E-06 |  | 0.0333 | 0.0722 | 0.6447 | 23.22055677 |
| Infectious mononucleosis | rs12482260 | G | A | 0.01528 | 0.333816 | 0.0690714 | 1.35E-06 |  | 0.0047 | 0.1666 | 0.9774 | 23.35703869 |
| Infectious mononucleosis | rs12573103 | G | A | 0.6113 | -0.0905768 | 0.0187059 | 1.28E-06 |  | 0.027 | 0.042 | 0.5203 | 23.44643025 |
| Infectious mononucleosis | rs12602989 | T | C | 0.1929 | 0.117641 | 0.0236795 | 6.76E-07 |  | -0.037 | 0.052 | 0.4764 | 24.68154599 |
| Infectious mononucleosis | rs12924608 | C | T | 0.3769 | 0.0927112 | 0.0189619 | 1.01E-06 |  | 0.0835 | 0.0425 | 0.0492901 | 23.90565794 |
| Infectious mononucleosis | rs12995271 | C | T | 0.2138 | -0.0982709 | 0.0214526 | 4.63E-06 |  | -0.0653 | 0.0497 | 0.1892 | 20.98408307 |
| Infectious mononucleosis | rs13267723 | A | G | 0.1992 | -0.107534 | 0.0217893 | 8.01E-07 |  | 0.1257 | 0.0512 | 0.0140699 | 24.35594837 |
| Infectious mononucleosis | rs1476275 | T | C | 0.02038 | 0.26341 | 0.0547143 | 1.48E-06 |  | -0.0003 | 0.1483 | 0.9984 | 23.17729892 |
| Infectious mononucleosis | rs1835841 | T | C | 0.4163 | -0.0886655 | 0.0184343 | 1.51E-06 |  | 0.0086 | 0.0417 | 0.8375 | 23.13428242 |
| Infectious mononucleosis | rs2047469 | T | C | 0.2824 | 0.114101 | 0.0232276 | 9.00E-07 |  | 0.1254 | 0.0457 | 0.00609397 | 24.13071689 |
| Infectious mononucleosis | rs2241545 | G | A | 0.3281 | -0.0968023 | 0.0210325 | 4.17E-06 |  | -0.0137 | 0.0436 | 0.752899 | 21.18310199 |
| Infectious mononucleosis | rs2293607 | C | T | 0.2729 | -0.0989059 | 0.0214803 | 4.13E-06 |  | -0.0303 | 0.0459 | 0.5092 | 21.20135965 |
| Infectious mononucleosis | rs2330764 | C | T | 0.4583 | 0.0890894 | 0.0183096 | 1.14E-06 |  | 0.091 | 0.0412 | 0.0272998 | 23.67523807 |
| Infectious mononucleosis | rs34126012 | A | G | 0.3217 | -0.124931 | 0.0270073 | 3.73E-06 |  | 0.0133 | 0.0437 | 0.7601 | 21.39824202 |
| Infectious mononucleosis | rs4149909 | G | A | 0.03156 | 0.276204 | 0.0556336 | 6.88E-07 |  | 0.0446 | 0.1188 | 0.707499 | 24.64822219 |
| Infectious mononucleosis | rs4150221 | C | T | 0.3194 | -0.0985601 | 0.0206477 | 1.81E-06 |  | -0.004 | 0.0438 | 0.9265 | 22.78551809 |
| Infectious mononucleosis | rs4443239 | G | T | 0.1667 | -0.0966618 | 0.0210859 | 4.56E-06 |  | 0.0058 | 0.0552 | 0.916 | 21.01481017 |
| Infectious mononucleosis | rs4606014 | T | C | 0.468 | 0.0841955 | 0.0181002 | 3.29E-06 |  | 0.0287 | 0.041 | 0.4839 | 21.63769593 |
| Infectious mononucleosis | rs4754296 | G | T | 0.2235 | -0.128209 | 0.0267998 | 1.72E-06 |  | 0.0145 | 0.0494 | 0.769501 | 22.88621216 |
| Infectious mononucleosis | rs4938513 | C | T | 0.5835 | -0.0923986 | 0.0190957 | 1.31E-06 |  | 0.0257 | 0.0418 | 0.5377 | 23.4131375 |
| Infectious mononucleosis | rs4975620 | C | T | 0.3479 | 0.0901892 | 0.0196214 | 4.30E-06 |  | -0.0658 | 0.0431 | 0.1268 | 21.12754749 |
| Infectious mononucleosis | rs56795824 | C | T | 0.02745 | 0.375227 | 0.0811956 | 3.81E-06 |  | 0.0391 | 0.1269 | 0.758099 | 21.35616027 |
| Infectious mononucleosis | rs5756908 | T | C | 0.5576 | -0.0853171 | 0.018151 | 2.60E-06 |  | -0.0075 | 0.0413 | 0.857 | 22.09383246 |
| Infectious mononucleosis | rs61364850 | G | A | 0.02453 | -0.193051 | 0.0419428 | 4.17E-06 |  | 0.0549 | 0.1319 | 0.6773 | 21.18503921 |
| Infectious mononucleosis | rs62137639 | G | A | 0.02134 | 0.268696 | 0.0551093 | 1.08E-06 |  | 0.2394 | 0.1417 | 0.0911108 | 23.77237723 |
| Infectious mononucleosis | rs62556523 | A | G | 0.04687 | 0.19645 | 0.0423733 | 3.55E-06 |  | -0.1389 | 0.1 | 0.1648 | 21.49411116 |
| Infectious mononucleosis | rs7679589 | A | C | 0.025 | -0.251704 | 0.0542147 | 3.44E-06 |  | -0.0313 | 0.1306 | 0.8107 | 21.55490518 |
| Infectious mononucleosis | rs78341235 | C | T | 0.3242 | 0.138307 | 0.0292407 | 2.25E-06 |  | -0.0784 | 0.0439 | 0.0741993 | 22.37241116 |
| Infectious mononucleosis | rs926771 | T | C | 0.07874 | -0.161931 | 0.0332555 | 1.12E-06 |  | 0.0245 | 0.0759 | 0.7472 | 23.71008069 |
| Infectious mononucleosis | rs9909962 | C | T | 0.09662 | 0.147003 | 0.0311733 | 2.41E-06 |  | -0.0221 | 0.0695 | 0.750699 | 22.23754488 |
| Hepatitis | rs10060827 | T | G | 0.3319 | -0.094712 | 0.0197935 | 1.71E-06 |  | -0.0633 | 0.0439 | 0.1489 | 22.89627456 |
| Hepatitis | rs10889496 | G | A | 0.4694 | -0.0932824 | 0.019739 | 2.29E-06 |  | -0.0181 | 0.0415 | 0.663399 | 22.33310606 |
| Hepatitis | rs10981985 | A | G | 0.1503 | -0.138288 | 0.0293504 | 2.46E-06 |  | -0.0931 | 0.0578 | 0.1072 | 22.19938497 |
| Hepatitis | rs11002922 | T | C | 0.3114 | 0.0954619 | 0.0202804 | 2.51E-06 |  | -0.0021 | 0.0445 | 0.9617 | 22.15680395 |
| Hepatitis | rs111384268 | A | G | 0.1134 | 0.15554 | 0.0312497 | 6.45E-07 |  | -0.0407 | 0.0653 | 0.5326 | 24.77379185 |
| Hepatitis | rs11143886 | A | C | 0.04454 | 0.185149 | 0.0403842 | 4.55E-06 |  | 0.2293 | 0.0989 | 0.0204 | 21.01937379 |
| Hepatitis | rs11233250 | T | C | 0.119 | -0.135681 | 0.0294397 | 4.05E-06 |  | -0.0249 | 0.0639 | 0.6974 | 21.2408216 |
| Hepatitis | rs11576512 | A | G | 0.1714 | -0.117617 | 0.0241214 | 1.08E-06 |  | 0.0058 | 0.0562 | 0.9176 | 23.77580196 |
| Hepatitis | rs117135784 | A | G | 0.02855 | 0.349187 | 0.0663151 | 1.40E-07 |  | 0.063 | 0.1256 | 0.616 | 27.72625901 |
| Hepatitis | rs12449010 | T | C | 0.08771 | -0.174121 | 0.0361339 | 1.44E-06 |  | 0.0399 | 0.073 | 0.5847 | 23.22055677 |
| Hepatitis | rs12482260 | G | A | 0.01529 | 0.333816 | 0.0690714 | 1.35E-06 |  | 0.0255 | 0.169 | 0.8802 | 23.35703869 |
| Hepatitis | rs12573103 | G | A | 0.6113 | -0.0905768 | 0.0187059 | 1.28E-06 |  | 0.0204 | 0.0423 | 0.629901 | 23.44643025 |
| Hepatitis | rs12602989 | T | C | 0.1928 | 0.117641 | 0.0236795 | 6.76E-07 |  | -0.01 | 0.0523 | 0.8489 | 24.68154599 |
| Hepatitis | rs12924608 | C | T | 0.3768 | 0.0927112 | 0.0189619 | 1.01E-06 |  | -0.0036 | 0.0427 | 0.9337 | 23.90565794 |
| Hepatitis | rs12995271 | C | T | 0.2138 | -0.0982709 | 0.0214526 | 4.63E-06 |  | -0.0121 | 0.0498 | 0.8077 | 20.98408307 |
| Hepatitis | rs13267723 | A | G | 0.1991 | -0.107534 | 0.0217893 | 8.01E-07 |  | -0.0023 | 0.0516 | 0.9638 | 24.35594837 |
| Hepatitis | rs1476275 | T | C | 0.0204 | 0.26341 | 0.0547143 | 1.48E-06 |  | 0.1177 | 0.145 | 0.417 | 23.17729892 |
| Hepatitis | rs1835841 | T | C | 0.4163 | -0.0886655 | 0.0184343 | 1.51E-06 |  | 0.012 | 0.042 | 0.7757 | 23.13428242 |
| Hepatitis | rs2047469 | T | C | 0.2823 | 0.114101 | 0.0232276 | 9.00E-07 |  | 0.0049 | 0.0459 | 0.9155 | 24.13071689 |
| Hepatitis | rs2241545 | G | A | 0.328 | -0.0968023 | 0.0210325 | 4.17E-06 |  | 0.0221 | 0.0439 | 0.6152 | 21.18310199 |
| Hepatitis | rs2293607 | C | T | 0.2729 | -0.0989059 | 0.0214803 | 4.13E-06 |  | 0.0202 | 0.0464 | 0.6629 | 21.20135965 |
| Hepatitis | rs2330764 | C | T | 0.4581 | 0.0890894 | 0.0183096 | 1.14E-06 |  | -0.0266 | 0.0414 | 0.519501 | 23.67523807 |
| Hepatitis | rs34126012 | A | G | 0.3216 | -0.124931 | 0.0270073 | 3.73E-06 |  | -0.0964 | 0.0443 | 0.0296497 | 21.39824202 |
| Hepatitis | rs4149909 | G | A | 0.03155 | 0.276204 | 0.0556336 | 6.88E-07 |  | -0.2148 | 0.1184 | 0.0696899 | 24.64822219 |
| Hepatitis | rs4150221 | C | T | 0.3194 | -0.0985601 | 0.0206477 | 1.81E-06 |  | -0.0031 | 0.0441 | 0.9432 | 22.78551809 |
| Hepatitis | rs4443239 | G | T | 0.1668 | -0.0966618 | 0.0210859 | 4.56E-06 |  | -0.0099 | 0.0552 | 0.8569 | 21.01481017 |
| Hepatitis | rs4606014 | T | C | 0.4679 | 0.0841955 | 0.0181002 | 3.29E-06 |  | -0.0277 | 0.0412 | 0.501 | 21.63769593 |
| Hepatitis | rs4754296 | G | T | 0.2235 | -0.128209 | 0.0267998 | 1.72E-06 |  | 0.0447 | 0.0495 | 0.3659 | 22.88621216 |
| Hepatitis | rs4938513 | C | T | 0.5834 | -0.0923986 | 0.0190957 | 1.31E-06 |  | 0.0697 | 0.0421 | 0.0981703 | 23.4131375 |
| Hepatitis | rs4975620 | C | T | 0.348 | 0.0901892 | 0.0196214 | 4.30E-06 |  | 0.0068 | 0.0434 | 0.8762 | 21.12754749 |
| Hepatitis | rs56795824 | C | T | 0.02745 | 0.375227 | 0.0811956 | 3.81E-06 |  | 0.0669 | 0.1284 | 0.6024 | 21.35616027 |
| Hepatitis | rs5756908 | T | C | 0.5576 | -0.0853171 | 0.018151 | 2.60E-06 |  | 0.0112 | 0.0416 | 0.787 | 22.09383246 |
| Hepatitis | rs61364850 | G | A | 0.02451 | -0.193051 | 0.0419428 | 4.17E-06 |  | 0.1418 | 0.1347 | 0.2926 | 21.18503921 |
| Hepatitis | rs62137639 | G | A | 0.02136 | 0.268696 | 0.0551093 | 1.08E-06 |  | 0.0763 | 0.1428 | 0.593 | 23.77237723 |
| Hepatitis | rs62556523 | A | G | 0.04683 | 0.19645 | 0.0423733 | 3.55E-06 |  | -0.1344 | 0.1024 | 0.1897 | 21.49411116 |
| Hepatitis | rs7679589 | A | C | 0.02505 | -0.251704 | 0.0542147 | 3.44E-06 |  | -0.031 | 0.1332 | 0.8159 | 21.55490518 |
| Hepatitis | rs78341235 | C | T | 0.3242 | 0.138307 | 0.0292407 | 2.25E-06 |  | -0.046 | 0.0444 | 0.3008 | 22.37241116 |
| Hepatitis | rs926771 | T | C | 0.07869 | -0.161931 | 0.0332555 | 1.12E-06 |  | -0.0569 | 0.0765 | 0.4569 | 23.71008069 |
| Hepatitis | rs9909962 | C | T | 0.09654 | 0.147003 | 0.0311733 | 2.41E-06 |  | -0.0809 | 0.0691 | 0.2418 | 22.23754488 |
| Herpesviral infections | rs10060827 | T | G | 0.3319 | -0.094712 | 0.0197935 | 1.71E-06 |  | -0.0073 | 0.038 | 0.8471 | 22.89627456 |
| Herpesviral infections | rs10889496 | G | A | 0.4693 | -0.0932824 | 0.019739 | 2.29E-06 |  | -0.0423 | 0.0359 | 0.2389 | 22.33310606 |
| Herpesviral infections | rs10981985 | A | G | 0.1503 | -0.138288 | 0.0293504 | 2.46E-06 |  | 0.0676 | 0.0499 | 0.1757 | 22.19938497 |
| Herpesviral infections | rs11002922 | T | C | 0.3116 | 0.0954619 | 0.0202804 | 2.51E-06 |  | -0.0366 | 0.0384 | 0.3409 | 22.15680395 |
| Herpesviral infections | rs111384268 | A | G | 0.1134 | 0.15554 | 0.0312497 | 6.45E-07 |  | -0.0913 | 0.0566 | 0.1067 | 24.77379185 |
| Herpesviral infections | rs11143886 | A | C | 0.04452 | 0.185149 | 0.0403842 | 4.55E-06 |  | -0.0101 | 0.0859 | 0.9063 | 21.01937379 |
| Herpesviral infections | rs11233250 | T | C | 0.119 | -0.135681 | 0.0294397 | 4.05E-06 |  | 0.0616 | 0.055 | 0.2627 | 21.2408216 |
| Herpesviral infections | rs11576512 | A | G | 0.1714 | -0.117617 | 0.0241214 | 1.08E-06 |  | -0.0894 | 0.0483 | 0.06418 | 23.77580196 |
| Herpesviral infections | rs117135784 | A | G | 0.02851 | 0.349187 | 0.0663151 | 1.40E-07 |  | 0.0078 | 0.108 | 0.9427 | 27.72625901 |
| Herpesviral infections | rs12449010 | T | C | 0.08764 | -0.174121 | 0.0361339 | 1.44E-06 |  | 0.0282 | 0.063 | 0.6546 | 23.22055677 |
| Herpesviral infections | rs12482260 | G | A | 0.01532 | 0.333816 | 0.0690714 | 1.35E-06 |  | -0.1674 | 0.1471 | 0.255 | 23.35703869 |
| Herpesviral infections | rs12573103 | G | A | 0.6111 | -0.0905768 | 0.0187059 | 1.28E-06 |  | 0.011 | 0.0366 | 0.7637 | 23.44643025 |
| Herpesviral infections | rs12602989 | T | C | 0.1927 | 0.117641 | 0.0236795 | 6.76E-07 |  | -0.0544 | 0.0453 | 0.2298 | 24.68154599 |
| Herpesviral infections | rs12924608 | C | T | 0.3767 | 0.0927112 | 0.0189619 | 1.01E-06 |  | -0.0589 | 0.037 | 0.1116 | 23.90565794 |
| Herpesviral infections | rs12995271 | C | T | 0.2139 | -0.0982709 | 0.0214526 | 4.63E-06 |  | 0.0127 | 0.0432 | 0.768799 | 20.98408307 |
| Herpesviral infections | rs13267723 | A | G | 0.199 | -0.107534 | 0.0217893 | 8.01E-07 |  | -0.0465 | 0.0447 | 0.2984 | 24.35594837 |
| Herpesviral infections | rs1476275 | T | C | 0.02044 | 0.26341 | 0.0547143 | 1.48E-06 |  | -0.0313 | 0.127 | 0.805 | 23.17729892 |
| Herpesviral infections | rs1835841 | T | C | 0.4162 | -0.0886655 | 0.0184343 | 1.51E-06 |  | -0.0815 | 0.0363 | 0.0247201 | 23.13428242 |
| Herpesviral infections | rs2047469 | T | C | 0.2822 | 0.114101 | 0.0232276 | 9.00E-07 |  | -0.0044 | 0.0397 | 0.9124 | 24.13071689 |
| Herpesviral infections | rs2241545 | G | A | 0.3279 | -0.0968023 | 0.0210325 | 4.17E-06 |  | -0.0697 | 0.0379 | 0.0660602 | 21.18310199 |
| Herpesviral infections | rs2293607 | C | T | 0.2729 | -0.0989059 | 0.0214803 | 4.13E-06 |  | 0.0178 | 0.0401 | 0.656401 | 21.20135965 |
| Herpesviral infections | rs2330764 | C | T | 0.4581 | 0.0890894 | 0.0183096 | 1.14E-06 |  | -0.033 | 0.0358 | 0.3566 | 23.67523807 |
| Herpesviral infections | rs34126012 | A | G | 0.3215 | -0.124931 | 0.0270073 | 3.73E-06 |  | 0.0408 | 0.0381 | 0.2845 | 21.39824202 |
| Herpesviral infections | rs4149909 | G | A | 0.0316 | 0.276204 | 0.0556336 | 6.88E-07 |  | 0.3165 | 0.1019 | 0.00189802 | 24.64822219 |
| Herpesviral infections | rs4150221 | C | T | 0.3195 | -0.0985601 | 0.0206477 | 1.81E-06 |  | -0.0044 | 0.0381 | 0.9089 | 22.78551809 |
| Herpesviral infections | rs4443239 | G | T | 0.1669 | -0.0966618 | 0.0210859 | 4.56E-06 |  | 0.0175 | 0.048 | 0.7149 | 21.01481017 |
| Herpesviral infections | rs4606014 | T | C | 0.468 | 0.0841955 | 0.0181002 | 3.29E-06 |  | -0.0109 | 0.0357 | 0.7604 | 21.63769593 |
| Herpesviral infections | rs4754296 | G | T | 0.2235 | -0.128209 | 0.0267998 | 1.72E-06 |  | -0.0035 | 0.0428 | 0.9343 | 22.88621216 |
| Herpesviral infections | rs4938513 | C | T | 0.5835 | -0.0923986 | 0.0190957 | 1.31E-06 |  | -0.0203 | 0.0364 | 0.575999 | 23.4131375 |
| Herpesviral infections | rs4975620 | C | T | 0.348 | 0.0901892 | 0.0196214 | 4.30E-06 |  | 0.0516 | 0.0374 | 0.168 | 21.12754749 |
| Herpesviral infections | rs56795824 | C | T | 0.0274 | 0.375227 | 0.0811956 | 3.81E-06 |  | -0.1715 | 0.1115 | 0.1238 | 21.35616027 |
| Herpesviral infections | rs5756908 | T | C | 0.5575 | -0.0853171 | 0.018151 | 2.60E-06 |  | 0.0012 | 0.036 | 0.9738 | 22.09383246 |
| Herpesviral infections | rs61364850 | G | A | 0.02447 | -0.193051 | 0.0419428 | 4.17E-06 |  | -0.1082 | 0.1166 | 0.3535 | 21.18503921 |
| Herpesviral infections | rs62137639 | G | A | 0.02141 | 0.268696 | 0.0551093 | 1.08E-06 |  | 0.0145 | 0.1247 | 0.9077 | 23.77237723 |
| Herpesviral infections | rs62556523 | A | G | 0.04679 | 0.19645 | 0.0423733 | 3.55E-06 |  | -0.0393 | 0.0878 | 0.6544 | 21.49411116 |
| Herpesviral infections | rs7679589 | A | C | 0.0251 | -0.251704 | 0.0542147 | 3.44E-06 |  | 0.1026 | 0.1148 | 0.3715 | 21.55490518 |
| Herpesviral infections | rs78341235 | C | T | 0.3242 | 0.138307 | 0.0292407 | 2.25E-06 |  | -0.0121 | 0.0383 | 0.7509 | 22.37241116 |
| Herpesviral infections | rs926771 | T | C | 0.07868 | -0.161931 | 0.0332555 | 1.12E-06 |  | 0.073 | 0.0664 | 0.272 | 23.71008069 |
| Herpesviral infections | rs9909962 | C | T | 0.09652 | 0.147003 | 0.0311733 | 2.41E-06 |  | -0.0535 | 0.0604 | 0.3757 | 22.23754488 |
| HIV diease | rs10060827 | T | G | 0.3319 | -0.094712 | 0.0197935 | 1.71E-06 |  | 0.0444 | 0.0802 | 0.58 | 22.89627456 |
| HIV diease | rs10889496 | G | A | 0.4694 | -0.0932824 | 0.019739 | 2.29E-06 |  | 0.0397 | 0.0761 | 0.601399 | 22.33310606 |
| HIV diease | rs10981985 | A | G | 0.1503 | -0.138288 | 0.0293504 | 2.46E-06 |  | -0.0836 | 0.1059 | 0.4302 | 22.19938497 |
| HIV diease | rs11002922 | T | C | 0.3114 | 0.0954619 | 0.0202804 | 2.51E-06 |  | -0.1432 | 0.0816 | 0.0792301 | 22.15680395 |
| HIV diease | rs111384268 | A | G | 0.1134 | 0.15554 | 0.0312497 | 6.45E-07 |  | -0.0791 | 0.1201 | 0.5101 | 24.77379185 |
| HIV diease | rs11143886 | A | C | 0.04454 | 0.185149 | 0.0403842 | 4.55E-06 |  | 0.2431 | 0.1825 | 0.1828 | 21.01937379 |
| HIV diease | rs11233250 | T | C | 0.119 | -0.135681 | 0.0294397 | 4.05E-06 |  | -0.0356 | 0.1175 | 0.7616 | 21.2408216 |
| HIV diease | rs11576512 | A | G | 0.1714 | -0.117617 | 0.0241214 | 1.08E-06 |  | 0.0381 | 0.1008 | 0.7052 | 23.77580196 |
| HIV diease | rs117135784 | A | G | 0.02855 | 0.349187 | 0.0663151 | 1.40E-07 |  | -0.0675 | 0.2273 | 0.766399 | 27.72625901 |
| HIV diease | rs12449010 | T | C | 0.08771 | -0.174121 | 0.0361339 | 1.44E-06 |  | -0.1659 | 0.1351 | 0.2193 | 23.22055677 |
| HIV diease | rs12482260 | G | A | 0.01529 | 0.333816 | 0.0690714 | 1.35E-06 |  | 0.2717 | 0.3083 | 0.3781 | 23.35703869 |
| HIV diease | rs12573103 | G | A | 0.6113 | -0.0905768 | 0.0187059 | 1.28E-06 |  | -0.0522 | 0.0776 | 0.501 | 23.44643025 |
| HIV diease | rs12602989 | T | C | 0.1928 | 0.117641 | 0.0236795 | 6.76E-07 |  | -0.0477 | 0.0963 | 0.6201 | 24.68154599 |
| HIV diease | rs12924608 | C | T | 0.3768 | 0.0927112 | 0.0189619 | 1.01E-06 |  | -0.0838 | 0.0783 | 0.284 | 23.90565794 |
| HIV diease | rs12995271 | C | T | 0.2138 | -0.0982709 | 0.0214526 | 4.63E-06 |  | -0.092 | 0.0916 | 0.3152 | 20.98408307 |
| HIV diease | rs13267723 | A | G | 0.1991 | -0.107534 | 0.0217893 | 8.01E-07 |  | -0.1585 | 0.0937 | 0.0906108 | 24.35594837 |
| HIV diease | rs1476275 | T | C | 0.0204 | 0.26341 | 0.0547143 | 1.48E-06 |  | 0.1036 | 0.2673 | 0.6983 | 23.17729892 |
| HIV diease | rs1835841 | T | C | 0.4163 | -0.0886655 | 0.0184343 | 1.51E-06 |  | -0.0303 | 0.0771 | 0.6948 | 23.13428242 |
| HIV diease | rs2047469 | T | C | 0.2823 | 0.114101 | 0.0232276 | 9.00E-07 |  | 0.1096 | 0.084 | 0.1921 | 24.13071689 |
| HIV diease | rs2241545 | G | A | 0.328 | -0.0968023 | 0.0210325 | 4.17E-06 |  | -0.0167 | 0.0804 | 0.835 | 21.18310199 |
| HIV diease | rs2293607 | C | T | 0.2729 | -0.0989059 | 0.0214803 | 4.13E-06 |  | -0.0218 | 0.0852 | 0.7984 | 21.20135965 |
| HIV diease | rs2330764 | C | T | 0.4581 | 0.0890894 | 0.0183096 | 1.14E-06 |  | 0.0176 | 0.0758 | 0.8167 | 23.67523807 |
| HIV diease | rs34126012 | A | G | 0.3216 | -0.124931 | 0.0270073 | 3.73E-06 |  | 0.0673 | 0.0808 | 0.4044 | 21.39824202 |
| HIV diease | rs4149909 | G | A | 0.03155 | 0.276204 | 0.0556336 | 6.88E-07 |  | 0.1172 | 0.2159 | 0.5874 | 24.64822219 |
| HIV diease | rs4150221 | C | T | 0.3194 | -0.0985601 | 0.0206477 | 1.81E-06 |  | -0.0543 | 0.0808 | 0.501601 | 22.78551809 |
| HIV diease | rs4443239 | G | T | 0.1668 | -0.0966618 | 0.0210859 | 4.56E-06 |  | -0.0734 | 0.1014 | 0.4693 | 21.01481017 |
| HIV diease | rs4606014 | T | C | 0.4679 | 0.0841955 | 0.0181002 | 3.29E-06 |  | -0.019 | 0.0755 | 0.801 | 21.63769593 |
| HIV diease | rs4754296 | G | T | 0.2235 | -0.128209 | 0.0267998 | 1.72E-06 |  | -0.0104 | 0.0911 | 0.9088 | 22.88621216 |
| HIV diease | rs4938513 | C | T | 0.5834 | -0.0923986 | 0.0190957 | 1.31E-06 |  | 0.0232 | 0.0767 | 0.762801 | 23.4131375 |
| HIV diease | rs4975620 | C | T | 0.348 | 0.0901892 | 0.0196214 | 4.30E-06 |  | -0.0429 | 0.0796 | 0.5898 | 21.12754749 |
| HIV diease | rs56795824 | C | T | 0.02745 | 0.375227 | 0.0811956 | 3.81E-06 |  | -0.1636 | 0.2363 | 0.4887 | 21.35616027 |
| HIV diease | rs5756908 | T | C | 0.5576 | -0.0853171 | 0.018151 | 2.60E-06 |  | -0.0092 | 0.076 | 0.9036 | 22.09383246 |
| HIV diease | rs61364850 | G | A | 0.02451 | -0.193051 | 0.0419428 | 4.17E-06 |  | 0.0478 | 0.2467 | 0.8465 | 21.18503921 |
| HIV diease | rs62137639 | G | A | 0.02136 | 0.268696 | 0.0551093 | 1.08E-06 |  | 0.511 | 0.2599 | 0.0493503 | 23.77237723 |
| HIV diease | rs62556523 | A | G | 0.04683 | 0.19645 | 0.0423733 | 3.55E-06 |  | 0.1991 | 0.1857 | 0.2835 | 21.49411116 |
| HIV diease | rs7679589 | A | C | 0.02505 | -0.251704 | 0.0542147 | 3.44E-06 |  | 0.1015 | 0.2394 | 0.6716 | 21.55490518 |
| HIV diease | rs78341235 | C | T | 0.3242 | 0.138307 | 0.0292407 | 2.25E-06 |  | -0.2218 | 0.0813 | 0.00636502 | 22.37241116 |
| HIV diease | rs926771 | T | C | 0.07869 | -0.161931 | 0.0332555 | 1.12E-06 |  | -0.1334 | 0.1392 | 0.3378 | 23.71008069 |
| HIV diease | rs9909962 | C | T | 0.09654 | 0.147003 | 0.0311733 | 2.41E-06 |  | -0.1271 | 0.1273 | 0.3183 | 22.23754488 |
| HPV | rs10981985 | A | G | 0.1503 | -0.138288 | 0.0293504 | 2.46E-06 |  | 0.09816 | 0.0687877 | 0.154 | 22.19938497 |
| HPV | rs12449010 | T | C | 0.08771 | -0.174121 | 0.0361339 | 1.44E-06 |  | -0.09263 | 0.0823378 | 0.2607 | 23.22055677 |
| HPV | rs1476275 | T | C | 0.0204 | 0.26341 | 0.0547143 | 1.48E-06 |  | 0.2544 | 0.147907 | 0.0857393 | 23.17729892 |
| HPV | rs4149909 | G | A | 0.03155 | 0.276204 | 0.0556336 | 6.88E-07 |  | -0.1926 | 0.136596 | 0.1588 | 24.64822219 |
| measles | rs10060827 | T | G | 0.3319 | -0.094712 | 0.0197935 | 1.71E-06 |  | -0.0103 | 0.1126 | 0.9271 | 22.89627456 |
| measles | rs10889496 | G | A | 0.4693 | -0.0932824 | 0.019739 | 2.29E-06 |  | -0.0169 | 0.1064 | 0.8737 | 22.33310606 |
| measles | rs10981985 | A | G | 0.1502 | -0.138288 | 0.0293504 | 2.46E-06 |  | -2.00E-04 | 0.1483 | 0.9988 | 22.19938497 |
| measles | rs11002922 | T | C | 0.3117 | 0.0954619 | 0.0202804 | 2.51E-06 |  | 0.0707 | 0.1138 | 0.534399 | 22.15680395 |
| measles | rs111384268 | A | G | 0.1134 | 0.15554 | 0.0312497 | 6.45E-07 |  | -0.1001 | 0.168 | 0.551501 | 24.77379185 |
| measles | rs11143886 | A | C | 0.04454 | 0.185149 | 0.0403842 | 4.55E-06 |  | 0.387 | 0.2549 | 0.129 | 21.01937379 |
| measles | rs11233250 | T | C | 0.119 | -0.135681 | 0.0294397 | 4.05E-06 |  | 0.2686 | 0.1631 | 0.0996093 | 21.2408216 |
| measles | rs11576512 | A | G | 0.1715 | -0.117617 | 0.0241214 | 1.08E-06 |  | -0.1344 | 0.1432 | 0.3479 | 23.77580196 |
| measles | rs117135784 | A | G | 0.02851 | 0.349187 | 0.0663151 | 1.40E-07 |  | -0.0338 | 0.3178 | 0.9154 | 27.72625901 |
| measles | rs12449010 | T | C | 0.0876 | -0.174121 | 0.0361339 | 1.44E-06 |  | -0.3361 | 0.1863 | 0.0712492 | 23.22055677 |
| measles | rs12482260 | G | A | 0.01535 | 0.333816 | 0.0690714 | 1.35E-06 |  | 0.7308 | 0.4357 | 0.0935104 | 23.35703869 |
| measles | rs12573103 | G | A | 0.6111 | -0.0905768 | 0.0187059 | 1.28E-06 |  | -0.1824 | 0.1085 | 0.0927299 | 23.44643025 |
| measles | rs12602989 | T | C | 0.1928 | 0.117641 | 0.0236795 | 6.76E-07 |  | -0.1398 | 0.1343 | 0.2979 | 24.68154599 |
| measles | rs12924608 | C | T | 0.3768 | 0.0927112 | 0.0189619 | 1.01E-06 |  | -0.0725 | 0.1097 | 0.5087 | 23.90565794 |
| measles | rs12995271 | C | T | 0.2139 | -0.0982709 | 0.0214526 | 4.63E-06 |  | -0.2131 | 0.1283 | 0.0965895 | 20.98408307 |
| measles | rs13267723 | A | G | 0.199 | -0.107534 | 0.0217893 | 8.01E-07 |  | -0.032 | 0.1325 | 0.8089 | 24.35594837 |
| measles | rs1476275 | T | C | 0.02044 | 0.26341 | 0.0547143 | 1.48E-06 |  | -0.1188 | 0.3812 | 0.7552 | 23.17729892 |
| measles | rs1835841 | T | C | 0.4163 | -0.0886655 | 0.0184343 | 1.51E-06 |  | -0.0526 | 0.1076 | 0.625299 | 23.13428242 |
| measles | rs2047469 | T | C | 0.2822 | 0.114101 | 0.0232276 | 9.00E-07 |  | -0.0412 | 0.1178 | 0.7264 | 24.13071689 |
| measles | rs2241545 | G | A | 0.328 | -0.0968023 | 0.0210325 | 4.17E-06 |  | 0.0612 | 0.1125 | 0.5866 | 21.18310199 |
| measles | rs2293607 | C | T | 0.2729 | -0.0989059 | 0.0214803 | 4.13E-06 |  | -0.2168 | 0.1188 | 0.0680096 | 21.20135965 |
| measles | rs2330764 | C | T | 0.4581 | 0.0890894 | 0.0183096 | 1.14E-06 |  | -0.0679 | 0.1064 | 0.5231 | 23.67523807 |
| measles | rs34126012 | A | G | 0.3215 | -0.124931 | 0.0270073 | 3.73E-06 |  | -0.1237 | 0.113 | 0.2739 | 21.39824202 |
| measles | rs4149909 | G | A | 0.03153 | 0.276204 | 0.0556336 | 6.88E-07 |  | -0.1216 | 0.3023 | 0.687401 | 24.64822219 |
| measles | rs4150221 | C | T | 0.3195 | -0.0985601 | 0.0206477 | 1.81E-06 |  | 0.0174 | 0.1129 | 0.8776 | 22.78551809 |
| measles | rs4443239 | G | T | 0.1668 | -0.0966618 | 0.0210859 | 4.56E-06 |  | -0.1963 | 0.1427 | 0.1692 | 21.01481017 |
| measles | rs4606014 | T | C | 0.468 | 0.0841955 | 0.0181002 | 3.29E-06 |  | -0.0784 | 0.1058 | 0.4587 | 21.63769593 |
| measles | rs4754296 | G | T | 0.2235 | -0.128209 | 0.0267998 | 1.72E-06 |  | -0.0674 | 0.1269 | 0.5953 | 22.88621216 |
| measles | rs4938513 | C | T | 0.5836 | -0.0923986 | 0.0190957 | 1.31E-06 |  | 0.3058 | 0.1079 | 0.00461604 | 23.4131375 |
| measles | rs4975620 | C | T | 0.3479 | 0.0901892 | 0.0196214 | 4.30E-06 |  | -0.1042 | 0.1109 | 0.3472 | 21.12754749 |
| measles | rs56795824 | C | T | 0.02744 | 0.375227 | 0.0811956 | 3.81E-06 |  | 0.3817 | 0.3276 | 0.244 | 21.35616027 |
| measles | rs5756908 | T | C | 0.5575 | -0.0853171 | 0.018151 | 2.60E-06 |  | -0.0606 | 0.1067 | 0.5703 | 22.09383246 |
| measles | rs61364850 | G | A | 0.02448 | -0.193051 | 0.0419428 | 4.17E-06 |  | -0.2039 | 0.3467 | 0.5565 | 21.18503921 |
| measles | rs62137639 | G | A | 0.0214 | 0.268696 | 0.0551093 | 1.08E-06 |  | -0.2312 | 0.3731 | 0.5354 | 23.77237723 |
| measles | rs62556523 | A | G | 0.0468 | 0.19645 | 0.0423733 | 3.55E-06 |  | -0.2428 | 0.2596 | 0.3497 | 21.49411116 |
| measles | rs7679589 | A | C | 0.02507 | -0.251704 | 0.0542147 | 3.44E-06 |  | -0.5788 | 0.3394 | 0.0881008 | 21.55490518 |
| measles | rs78341235 | C | T | 0.3242 | 0.138307 | 0.0292407 | 2.25E-06 |  | -0.0745 | 0.1134 | 0.5112 | 22.37241116 |
| measles | rs926771 | T | C | 0.07865 | -0.161931 | 0.0332555 | 1.12E-06 |  | 0.0852 | 0.1963 | 0.6642 | 23.71008069 |
| measles | rs9909962 | C | T | 0.09654 | 0.147003 | 0.0311733 | 2.41E-06 |  | -0.284 | 0.1793 | 0.1131 | 22.23754488 |
| mumps | rs10060827 | T | G | 0.3319 | -0.094712 | 0.0197935 | 1.71E-06 |  | 0.0783 | 0.0724 | 0.2795 | 22.89627456 |
| mumps | rs10889496 | G | A | 0.4696 | -0.0932824 | 0.019739 | 2.29E-06 |  | 0.0653 | 0.0684 | 0.3401 | 22.33310606 |
| mumps | rs10981985 | A | G | 0.1502 | -0.138288 | 0.0293504 | 2.46E-06 |  | 0.0835 | 0.0952 | 0.3802 | 22.19938497 |
| mumps | rs11002922 | T | C | 0.3115 | 0.0954619 | 0.0202804 | 2.51E-06 |  | -0.0574 | 0.0731 | 0.4326 | 22.15680395 |
| mumps | rs111384268 | A | G | 0.1133 | 0.15554 | 0.0312497 | 6.45E-07 |  | -0.0904 | 0.1082 | 0.4034 | 24.77379185 |
| mumps | rs11143886 | A | C | 0.04457 | 0.185149 | 0.0403842 | 4.55E-06 |  | 0.0941 | 0.1634 | 0.5648 | 21.01937379 |
| mumps | rs11233250 | T | C | 0.119 | -0.135681 | 0.0294397 | 4.05E-06 |  | 0.225 | 0.1046 | 0.0313899 | 21.2408216 |
| mumps | rs11576512 | A | G | 0.1714 | -0.117617 | 0.0241214 | 1.08E-06 |  | 0.039 | 0.0921 | 0.672 | 23.77580196 |
| mumps | rs117135784 | A | G | 0.02853 | 0.349187 | 0.0663151 | 1.40E-07 |  | -0.0493 | 0.2048 | 0.8099 | 27.72625901 |
| mumps | rs12449010 | T | C | 0.08776 | -0.174121 | 0.0361339 | 1.44E-06 |  | 0.001 | 0.1194 | 0.9934 | 23.22055677 |
| mumps | rs12482260 | G | A | 0.01529 | 0.333816 | 0.0690714 | 1.35E-06 |  | 0.324 | 0.2799 | 0.2471 | 23.35703869 |
| mumps | rs12573103 | G | A | 0.6112 | -0.0905768 | 0.0187059 | 1.28E-06 |  | -0.0854 | 0.0698 | 0.2212 | 23.44643025 |
| mumps | rs12602989 | T | C | 0.1929 | 0.117641 | 0.0236795 | 6.76E-07 |  | -0.0445 | 0.0863 | 0.6058 | 24.68154599 |
| mumps | rs12924608 | C | T | 0.3768 | 0.0927112 | 0.0189619 | 1.01E-06 |  | -0.0321 | 0.0705 | 0.6486 | 23.90565794 |
| mumps | rs12995271 | C | T | 0.2138 | -0.0982709 | 0.0214526 | 4.63E-06 |  | 0.0369 | 0.0824 | 0.654 | 20.98408307 |
| mumps | rs13267723 | A | G | 0.199 | -0.107534 | 0.0217893 | 8.01E-07 |  | -0.1483 | 0.0852 | 0.0819691 | 24.35594837 |
| mumps | rs1476275 | T | C | 0.02036 | 0.26341 | 0.0547143 | 1.48E-06 |  | -0.4211 | 0.2434 | 0.0835391 | 23.17729892 |
| mumps | rs1835841 | T | C | 0.4162 | -0.0886655 | 0.0184343 | 1.51E-06 |  | -0.1783 | 0.0692 | 0.00999908 | 23.13428242 |
| mumps | rs2047469 | T | C | 0.2822 | 0.114101 | 0.0232276 | 9.00E-07 |  | -0.0047 | 0.0757 | 0.9502 | 24.13071689 |
| mumps | rs2241545 | G | A | 0.3282 | -0.0968023 | 0.0210325 | 4.17E-06 |  | 0.1058 | 0.0724 | 0.1437 | 21.18310199 |
| mumps | rs2293607 | C | T | 0.2729 | -0.0989059 | 0.0214803 | 4.13E-06 |  | -0.0104 | 0.0763 | 0.8917 | 21.20135965 |
| mumps | rs2330764 | C | T | 0.4582 | 0.0890894 | 0.0183096 | 1.14E-06 |  | -0.047 | 0.0682 | 0.4912 | 23.67523807 |
| mumps | rs34126012 | A | G | 0.3217 | -0.124931 | 0.0270073 | 3.73E-06 |  | -0.0141 | 0.0727 | 0.8463 | 21.39824202 |
| mumps | rs4149909 | G | A | 0.03156 | 0.276204 | 0.0556336 | 6.88E-07 |  | 0.0623 | 0.1945 | 0.7487 | 24.64822219 |
| mumps | rs4150221 | C | T | 0.3194 | -0.0985601 | 0.0206477 | 1.81E-06 |  | 0.0734 | 0.0725 | 0.3111 | 22.78551809 |
| mumps | rs4443239 | G | T | 0.1667 | -0.0966618 | 0.0210859 | 4.56E-06 |  | -0.1008 | 0.0919 | 0.2728 | 21.01481017 |
| mumps | rs4606014 | T | C | 0.468 | 0.0841955 | 0.0181002 | 3.29E-06 |  | -0.0313 | 0.0681 | 0.6462 | 21.63769593 |
| mumps | rs4754296 | G | T | 0.2235 | -0.128209 | 0.0267998 | 1.72E-06 |  | -0.0342 | 0.0815 | 0.675 | 22.88621216 |
| mumps | rs4938513 | C | T | 0.5834 | -0.0923986 | 0.0190957 | 1.31E-06 |  | -0.1197 | 0.0693 | 0.0841105 | 23.4131375 |
| mumps | rs4975620 | C | T | 0.348 | 0.0901892 | 0.0196214 | 4.30E-06 |  | -0.0494 | 0.0712 | 0.487401 | 21.12754749 |
| mumps | rs56795824 | C | T | 0.02744 | 0.375227 | 0.0811956 | 3.81E-06 |  | -0.0186 | 0.2108 | 0.9298 | 21.35616027 |
| mumps | rs5756908 | T | C | 0.5576 | -0.0853171 | 0.018151 | 2.60E-06 |  | 0.0653 | 0.0685 | 0.3402 | 22.09383246 |
| mumps | rs61364850 | G | A | 0.02451 | -0.193051 | 0.0419428 | 4.17E-06 |  | -0.1973 | 0.2231 | 0.3765 | 21.18503921 |
| mumps | rs62137639 | G | A | 0.02132 | 0.268696 | 0.0551093 | 1.08E-06 |  | 0.2612 | 0.2412 | 0.2789 | 23.77237723 |
| mumps | rs62556523 | A | G | 0.04687 | 0.19645 | 0.0423733 | 3.55E-06 |  | -0.2585 | 0.1683 | 0.1245 | 21.49411116 |
| mumps | rs7679589 | A | C | 0.02502 | -0.251704 | 0.0542147 | 3.44E-06 |  | 0.3991 | 0.2191 | 0.0684794 | 21.55490518 |
| mumps | rs78341235 | C | T | 0.3244 | 0.138307 | 0.0292407 | 2.25E-06 |  | 0.0573 | 0.0728 | 0.4317 | 22.37241116 |
| mumps | rs926771 | T | C | 0.07874 | -0.161931 | 0.0332555 | 1.12E-06 |  | 0.0471 | 0.1257 | 0.707499 | 23.71008069 |
| mumps | rs9909962 | C | T | 0.09662 | 0.147003 | 0.0311733 | 2.41E-06 |  | -0.0482 | 0.1151 | 0.675499 | 22.23754488 |
| poliovirus | rs10060827 | T | G | 0.3319 | -0.094712 | 0.0197935 | 1.71E-06 |  | 0.1338 | 0.0999 | 0.1802 | 22.89627456 |
| poliovirus | rs10889496 | G | A | 0.4694 | -0.0932824 | 0.019739 | 2.29E-06 |  | 0.0842 | 0.0944 | 0.3721 | 22.33310606 |
| poliovirus | rs10981985 | A | G | 0.1503 | -0.138288 | 0.0293504 | 2.46E-06 |  | 0.2073 | 0.1311 | 0.114 | 22.19938497 |
| poliovirus | rs11002922 | T | C | 0.3115 | 0.0954619 | 0.0202804 | 2.51E-06 |  | 0.1486 | 0.1011 | 0.1417 | 22.15680395 |
| poliovirus | rs111384268 | A | G | 0.1134 | 0.15554 | 0.0312497 | 6.45E-07 |  | -0.1805 | 0.1483 | 0.2235 | 24.77379185 |
| poliovirus | rs11143886 | A | C | 0.04454 | 0.185149 | 0.0403842 | 4.55E-06 |  | -0.1868 | 0.2246 | 0.4056 | 21.01937379 |
| poliovirus | rs11233250 | T | C | 0.1191 | -0.135681 | 0.0294397 | 4.05E-06 |  | -0.0413 | 0.1445 | 0.774801 | 21.2408216 |
| poliovirus | rs11576512 | A | G | 0.1714 | -0.117617 | 0.0241214 | 1.08E-06 |  | -0.0175 | 0.1271 | 0.8907 | 23.77580196 |
| poliovirus | rs117135784 | A | G | 0.02856 | 0.349187 | 0.0663151 | 1.40E-07 |  | -0.3183 | 0.2853 | 0.2645 | 27.72625901 |
| poliovirus | rs12449010 | T | C | 0.08772 | -0.174121 | 0.0361339 | 1.44E-06 |  | -0.0595 | 0.1658 | 0.7199 | 23.22055677 |
| poliovirus | rs12482260 | G | A | 0.0153 | 0.333816 | 0.0690714 | 1.35E-06 |  | 0.1261 | 0.386 | 0.7438 | 23.35703869 |
| poliovirus | rs12573103 | G | A | 0.6113 | -0.0905768 | 0.0187059 | 1.28E-06 |  | -0.0185 | 0.0962 | 0.8476 | 23.44643025 |
| poliovirus | rs12602989 | T | C | 0.1928 | 0.117641 | 0.0236795 | 6.76E-07 |  | -0.0465 | 0.1196 | 0.6971 | 24.68154599 |
| poliovirus | rs12924608 | C | T | 0.3768 | 0.0927112 | 0.0189619 | 1.01E-06 |  | 0.1927 | 0.0973 | 0.0477101 | 23.90565794 |
| poliovirus | rs12995271 | C | T | 0.2139 | -0.0982709 | 0.0214526 | 4.63E-06 |  | 0.113 | 0.1132 | 0.3183 | 20.98408307 |
| poliovirus | rs13267723 | A | G | 0.1991 | -0.107534 | 0.0217893 | 8.01E-07 |  | 0.0649 | 0.117 | 0.579399 | 24.35594837 |
| poliovirus | rs1476275 | T | C | 0.0204 | 0.26341 | 0.0547143 | 1.48E-06 |  | -0.5049 | 0.3355 | 0.1324 | 23.17729892 |
| poliovirus | rs1835841 | T | C | 0.4163 | -0.0886655 | 0.0184343 | 1.51E-06 |  | -0.0358 | 0.0952 | 0.7071 | 23.13428242 |
| poliovirus | rs2047469 | T | C | 0.2823 | 0.114101 | 0.0232276 | 9.00E-07 |  | 0.0535 | 0.1039 | 0.606599 | 24.13071689 |
| poliovirus | rs2241545 | G | A | 0.328 | -0.0968023 | 0.0210325 | 4.17E-06 |  | 0.0119 | 0.0995 | 0.9049 | 21.18310199 |
| poliovirus | rs2293607 | C | T | 0.2729 | -0.0989059 | 0.0214803 | 4.13E-06 |  | 0.0469 | 0.1056 | 0.657201 | 21.20135965 |
| poliovirus | rs2330764 | C | T | 0.4581 | 0.0890894 | 0.0183096 | 1.14E-06 |  | 0.0991 | 0.094 | 0.2919 | 23.67523807 |
| poliovirus | rs34126012 | A | G | 0.3216 | -0.124931 | 0.0270073 | 3.73E-06 |  | 0.0819 | 0.1003 | 0.4139 | 21.39824202 |
| poliovirus | rs4149909 | G | A | 0.03157 | 0.276204 | 0.0556336 | 6.88E-07 |  | -0.6126 | 0.265 | 0.0207802 | 24.64822219 |
| poliovirus | rs4150221 | C | T | 0.3194 | -0.0985601 | 0.0206477 | 1.81E-06 |  | -0.0161 | 0.0998 | 0.872 | 22.78551809 |
| poliovirus | rs4443239 | G | T | 0.1668 | -0.0966618 | 0.0210859 | 4.56E-06 |  | 0.058 | 0.1264 | 0.6462 | 21.01481017 |
| poliovirus | rs4606014 | T | C | 0.4679 | 0.0841955 | 0.0181002 | 3.29E-06 |  | -0.0881 | 0.0939 | 0.3481 | 21.63769593 |
| poliovirus | rs4754296 | G | T | 0.2235 | -0.128209 | 0.0267998 | 1.72E-06 |  | 0.0147 | 0.1122 | 0.8956 | 22.88621216 |
| poliovirus | rs4938513 | C | T | 0.5834 | -0.0923986 | 0.0190957 | 1.31E-06 |  | -0.0554 | 0.0955 | 0.5623 | 23.4131375 |
| poliovirus | rs4975620 | C | T | 0.348 | 0.0901892 | 0.0196214 | 4.30E-06 |  | -0.1243 | 0.0983 | 0.2063 | 21.12754749 |
| poliovirus | rs56795824 | C | T | 0.02745 | 0.375227 | 0.0811956 | 3.81E-06 |  | 0.1357 | 0.2937 | 0.6441 | 21.35616027 |
| poliovirus | rs5756908 | T | C | 0.5575 | -0.0853171 | 0.018151 | 2.60E-06 |  | 0.1173 | 0.0945 | 0.2144 | 22.09383246 |
| poliovirus | rs61364850 | G | A | 0.02454 | -0.193051 | 0.0419428 | 4.17E-06 |  | -0.4553 | 0.312 | 0.1444 | 21.18503921 |
| poliovirus | rs62137639 | G | A | 0.02136 | 0.268696 | 0.0551093 | 1.08E-06 |  | 0.4622 | 0.3352 | 0.168 | 23.77237723 |
| poliovirus | rs62556523 | A | G | 0.04685 | 0.19645 | 0.0423733 | 3.55E-06 |  | -0.0246 | 0.2324 | 0.9156 | 21.49411116 |
| poliovirus | rs7679589 | A | C | 0.02505 | -0.251704 | 0.0542147 | 3.44E-06 |  | 0.274 | 0.3023 | 0.3646 | 21.55490518 |
| poliovirus | rs78341235 | C | T | 0.3242 | 0.138307 | 0.0292407 | 2.25E-06 |  | 0.0626 | 0.1007 | 0.534 | 22.37241116 |
| poliovirus | rs926771 | T | C | 0.0787 | -0.161931 | 0.0332555 | 1.12E-06 |  | 0.2336 | 0.1737 | 0.1786 | 23.71008069 |
| poliovirus | rs9909962 | C | T | 0.09649 | 0.147003 | 0.0311733 | 2.41E-06 |  | 0.1707 | 0.1591 | 0.2833 | 22.23754488 |
| Rubella | rs10060827 | T | G | 0.332 | -0.094712 | 0.0197935 | 1.71E-06 |  | 0.122 | 0.0647 | 0.0593895 | 22.89627456 |
| Rubella | rs10889496 | G | A | 0.4693 | -0.0932824 | 0.019739 | 2.29E-06 |  | 0.0027 | 0.0614 | 0.9653 | 22.33310606 |
| Rubella | rs10981985 | A | G | 0.1502 | -0.138288 | 0.0293504 | 2.46E-06 |  | 0.0662 | 0.0856 | 0.4389 | 22.19938497 |
| Rubella | rs11002922 | T | C | 0.3117 | 0.0954619 | 0.0202804 | 2.51E-06 |  | 0.0277 | 0.0654 | 0.6724 | 22.15680395 |
| Rubella | rs111384268 | A | G | 0.1134 | 0.15554 | 0.0312497 | 6.45E-07 |  | 0.0888 | 0.0969 | 0.3595 | 24.77379185 |
| Rubella | rs11143886 | A | C | 0.04452 | 0.185149 | 0.0403842 | 4.55E-06 |  | -0.0664 | 0.1466 | 0.6506 | 21.01937379 |
| Rubella | rs11233250 | T | C | 0.119 | -0.135681 | 0.0294397 | 4.05E-06 |  | 0.0297 | 0.0945 | 0.753401 | 21.2408216 |
| Rubella | rs11576512 | A | G | 0.1715 | -0.117617 | 0.0241214 | 1.08E-06 |  | 0.0183 | 0.0829 | 0.8254 | 23.77580196 |
| Rubella | rs117135784 | A | G | 0.02851 | 0.349187 | 0.0663151 | 1.40E-07 |  | -0.039 | 0.1844 | 0.8326 | 27.72625901 |
| Rubella | rs12449010 | T | C | 0.08765 | -0.174121 | 0.0361339 | 1.44E-06 |  | 0.1093 | 0.1079 | 0.3113 | 23.22055677 |
| Rubella | rs12482260 | G | A | 0.01532 | 0.333816 | 0.0690714 | 1.35E-06 |  | -0.3424 | 0.2508 | 0.1721 | 23.35703869 |
| Rubella | rs12573103 | G | A | 0.6112 | -0.0905768 | 0.0187059 | 1.28E-06 |  | 0.0841 | 0.0626 | 0.1795 | 23.44643025 |
| Rubella | rs12602989 | T | C | 0.1928 | 0.117641 | 0.0236795 | 6.76E-07 |  | -0.0099 | 0.0773 | 0.8982 | 24.68154599 |
| Rubella | rs12924608 | C | T | 0.3769 | 0.0927112 | 0.0189619 | 1.01E-06 |  | 0.1219 | 0.0633 | 0.0540804 | 23.90565794 |
| Rubella | rs12995271 | C | T | 0.2139 | -0.0982709 | 0.0214526 | 4.63E-06 |  | -0.1058 | 0.0739 | 0.152 | 20.98408307 |
| Rubella | rs13267723 | A | G | 0.1991 | -0.107534 | 0.0217893 | 8.01E-07 |  | 0.0759 | 0.0759 | 0.3173 | 24.35594837 |
| Rubella | rs1476275 | T | C | 0.02043 | 0.26341 | 0.0547143 | 1.48E-06 |  | -0.164 | 0.218 | 0.4519 | 23.17729892 |
| Rubella | rs1835841 | T | C | 0.4164 | -0.0886655 | 0.0184343 | 1.51E-06 |  | 0.0606 | 0.062 | 0.3282 | 23.13428242 |
| Rubella | rs2047469 | T | C | 0.2823 | 0.114101 | 0.0232276 | 9.00E-07 |  | 0.0395 | 0.0678 | 0.56 | 24.13071689 |
| Rubella | rs2241545 | G | A | 0.328 | -0.0968023 | 0.0210325 | 4.17E-06 |  | 0.0169 | 0.0649 | 0.7939 | 21.18310199 |
| Rubella | rs2293607 | C | T | 0.2729 | -0.0989059 | 0.0214803 | 4.13E-06 |  | 0.0088 | 0.0686 | 0.8974 | 21.20135965 |
| Rubella | rs2330764 | C | T | 0.4581 | 0.0890894 | 0.0183096 | 1.14E-06 |  | 0.0166 | 0.0612 | 0.7862 | 23.67523807 |
| Rubella | rs34126012 | A | G | 0.3215 | -0.124931 | 0.0270073 | 3.73E-06 |  | 0.0061 | 0.0652 | 0.926 | 21.39824202 |
| Rubella | rs4149909 | G | A | 0.03155 | 0.276204 | 0.0556336 | 6.88E-07 |  | 0.297 | 0.1757 | 0.0909997 | 24.64822219 |
| Rubella | rs4150221 | C | T | 0.3195 | -0.0985601 | 0.0206477 | 1.81E-06 |  | -0.0531 | 0.065 | 0.4142 | 22.78551809 |
| Rubella | rs4443239 | G | T | 0.1668 | -0.0966618 | 0.0210859 | 4.56E-06 |  | -0.017 | 0.0821 | 0.8356 | 21.01481017 |
| Rubella | rs4606014 | T | C | 0.4679 | 0.0841955 | 0.0181002 | 3.29E-06 |  | -0.0936 | 0.061 | 0.1249 | 21.63769593 |
| Rubella | rs4754296 | G | T | 0.2235 | -0.128209 | 0.0267998 | 1.72E-06 |  | -0.0047 | 0.0729 | 0.9491 | 22.88621216 |
| Rubella | rs4938513 | C | T | 0.5835 | -0.0923986 | 0.0190957 | 1.31E-06 |  | -0.028 | 0.0622 | 0.6522 | 23.4131375 |
| Rubella | rs4975620 | C | T | 0.3479 | 0.0901892 | 0.0196214 | 4.30E-06 |  | -0.0026 | 0.064 | 0.9679 | 21.12754749 |
| Rubella | rs56795824 | C | T | 0.02742 | 0.375227 | 0.0811956 | 3.81E-06 |  | -0.186 | 0.19 | 0.3275 | 21.35616027 |
| Rubella | rs5756908 | T | C | 0.5575 | -0.0853171 | 0.018151 | 2.60E-06 |  | 0.0083 | 0.0613 | 0.8928 | 22.09383246 |
| Rubella | rs61364850 | G | A | 0.02449 | -0.193051 | 0.0419428 | 4.17E-06 |  | 0.0242 | 0.2002 | 0.9037 | 21.18503921 |
| Rubella | rs62137639 | G | A | 0.0214 | 0.268696 | 0.0551093 | 1.08E-06 |  | -0.1536 | 0.2133 | 0.4715 | 23.77237723 |
| Rubella | rs62556523 | A | G | 0.04681 | 0.19645 | 0.0423733 | 3.55E-06 |  | 0.0662 | 0.1502 | 0.6591 | 21.49411116 |
| Rubella | rs7679589 | A | C | 0.02507 | -0.251704 | 0.0542147 | 3.44E-06 |  | -0.2179 | 0.1954 | 0.2649 | 21.55490518 |
| Rubella | rs78341235 | C | T | 0.3242 | 0.138307 | 0.0292407 | 2.25E-06 |  | -0.0723 | 0.0654 | 0.2688 | 22.37241116 |
| Rubella | rs926771 | T | C | 0.07862 | -0.161931 | 0.0332555 | 1.12E-06 |  | -0.1161 | 0.1141 | 0.3089 | 23.71008069 |
| Rubella | rs9909962 | C | T | 0.09656 | 0.147003 | 0.0311733 | 2.41E-06 |  | -0.0104 | 0.1015 | 0.9186 | 22.23754488 |
| Herpes zoster | rs10060827 | T | G | 0.3319 | -0.094712 | 0.0197935 | 1.71E-06 |  | -0.01 | 0.0335 | 0.764999 | 22.89627456 |
| Herpes zoster | rs10889496 | G | A | 0.4694 | -0.0932824 | 0.019739 | 2.29E-06 |  | 0.0176 | 0.0317 | 0.5772 | 22.33310606 |
| Herpes zoster | rs10981985 | A | G | 0.1502 | -0.138288 | 0.0293504 | 2.46E-06 |  | -0.0481 | 0.044 | 0.2752 | 22.19938497 |
| Herpes zoster | rs11002922 | T | C | 0.3115 | 0.0954619 | 0.0202804 | 2.51E-06 |  | -0.0611 | 0.0338 | 0.0706903 | 22.15680395 |
| Herpes zoster | rs111384268 | A | G | 0.1134 | 0.15554 | 0.0312497 | 6.45E-07 |  | 0 | 0.0498 | 0.9993 | 24.77379185 |
| Herpes zoster | rs11143886 | A | C | 0.04453 | 0.185149 | 0.0403842 | 4.55E-06 |  | -0.0014 | 0.0755 | 0.985 | 21.01937379 |
| Herpes zoster | rs11233250 | T | C | 0.119 | -0.135681 | 0.0294397 | 4.05E-06 |  | 0.031 | 0.0483 | 0.5206 | 21.2408216 |
| Herpes zoster | rs11576512 | A | G | 0.1715 | -0.117617 | 0.0241214 | 1.08E-06 |  | -0.031 | 0.0426 | 0.4662 | 23.77580196 |
| Herpes zoster | rs117135784 | A | G | 0.02855 | 0.349187 | 0.0663151 | 1.40E-07 |  | 0.1468 | 0.0953 | 0.1232 | 27.72625901 |
| Herpes zoster | rs12449010 | T | C | 0.08769 | -0.174121 | 0.0361339 | 1.44E-06 |  | 0.078 | 0.0555 | 0.1597 | 23.22055677 |
| Herpes zoster | rs12482260 | G | A | 0.01531 | 0.333816 | 0.0690714 | 1.35E-06 |  | -0.1642 | 0.1282 | 0.2003 | 23.35703869 |
| Herpes zoster | rs12573103 | G | A | 0.6112 | -0.0905768 | 0.0187059 | 1.28E-06 |  | 0.028 | 0.0322 | 0.384 | 23.44643025 |
| Herpes zoster | rs12602989 | T | C | 0.1929 | 0.117641 | 0.0236795 | 6.76E-07 |  | 0.046 | 0.0398 | 0.2485 | 24.68154599 |
| Herpes zoster | rs12924608 | C | T | 0.3769 | 0.0927112 | 0.0189619 | 1.01E-06 |  | 0.0253 | 0.0326 | 0.4371 | 23.90565794 |
| Herpes zoster | rs12995271 | C | T | 0.2139 | -0.0982709 | 0.0214526 | 4.63E-06 |  | -0.0081 | 0.038 | 0.8308 | 20.98408307 |
| Herpes zoster | rs13267723 | A | G | 0.1991 | -0.107534 | 0.0217893 | 8.01E-07 |  | 0.0288 | 0.0394 | 0.4647 | 24.35594837 |
| Herpes zoster | rs1476275 | T | C | 0.02039 | 0.26341 | 0.0547143 | 1.48E-06 |  | -0.2748 | 0.1118 | 0.0139499 | 23.17729892 |
| Herpes zoster | rs1835841 | T | C | 0.4165 | -0.0886655 | 0.0184343 | 1.51E-06 |  | 0.0585 | 0.0319 | 0.0670903 | 23.13428242 |
| Herpes zoster | rs2047469 | T | C | 0.2822 | 0.114101 | 0.0232276 | 9.00E-07 |  | -0.0224 | 0.035 | 0.522801 | 24.13071689 |
| Herpes zoster | rs2241545 | G | A | 0.3281 | -0.0968023 | 0.0210325 | 4.17E-06 |  | 0.0332 | 0.0334 | 0.3205 | 21.18310199 |
| Herpes zoster | rs2293607 | C | T | 0.2731 | -0.0989059 | 0.0214803 | 4.13E-06 |  | 0.0951 | 0.0353 | 0.00707294 | 21.20135965 |
| Herpes zoster | rs2330764 | C | T | 0.4583 | 0.0890894 | 0.0183096 | 1.14E-06 |  | 0.0496 | 0.0315 | 0.1157 | 23.67523807 |
| Herpes zoster | rs34126012 | A | G | 0.3216 | -0.124931 | 0.0270073 | 3.73E-06 |  | 0.0717 | 0.0336 | 0.0327198 | 21.39824202 |
| Herpes zoster | rs4149909 | G | A | 0.03147 | 0.276204 | 0.0556336 | 6.88E-07 |  | -0.1784 | 0.09 | 0.0474297 | 24.64822219 |
| Herpes zoster | rs4150221 | C | T | 0.3194 | -0.0985601 | 0.0206477 | 1.81E-06 |  | -0.0425 | 0.0335 | 0.205 | 22.78551809 |
| Herpes zoster | rs4443239 | G | T | 0.1669 | -0.0966618 | 0.0210859 | 4.56E-06 |  | 0.049 | 0.0423 | 0.2461 | 21.01481017 |
| Herpes zoster | rs4606014 | T | C | 0.468 | 0.0841955 | 0.0181002 | 3.29E-06 |  | -0.0113 | 0.0315 | 0.7204 | 21.63769593 |
| Herpes zoster | rs4754296 | G | T | 0.2235 | -0.128209 | 0.0267998 | 1.72E-06 |  | 5.00E-04 | 0.0377 | 0.9899 | 22.88621216 |
| Herpes zoster | rs4938513 | C | T | 0.5835 | -0.0923986 | 0.0190957 | 1.31E-06 |  | 0.0024 | 0.032 | 0.9411 | 23.4131375 |
| Herpes zoster | rs4975620 | C | T | 0.3479 | 0.0901892 | 0.0196214 | 4.30E-06 |  | -0.0276 | 0.0329 | 0.4026 | 21.12754749 |
| Herpes zoster | rs56795824 | C | T | 0.02745 | 0.375227 | 0.0811956 | 3.81E-06 |  | 0.0834 | 0.0984 | 0.3969 | 21.35616027 |
| Herpes zoster | rs5756908 | T | C | 0.5575 | -0.0853171 | 0.018151 | 2.60E-06 |  | 0.0028 | 0.0317 | 0.9287 | 22.09383246 |
| Herpes zoster | rs61364850 | G | A | 0.02456 | -0.193051 | 0.0419428 | 4.17E-06 |  | 0.3437 | 0.1036 | 0.00091071 | 21.18503921 |
| Herpes zoster | rs62137639 | G | A | 0.02137 | 0.268696 | 0.0551093 | 1.08E-06 |  | -0.1521 | 0.1106 | 0.1691 | 23.77237723 |
| Herpes zoster | rs62556523 | A | G | 0.04681 | 0.19645 | 0.0423733 | 3.55E-06 |  | 0.0362 | 0.078 | 0.6427 | 21.49411116 |
| Herpes zoster | rs7679589 | A | C | 0.02508 | -0.251704 | 0.0542147 | 3.44E-06 |  | -0.021 | 0.1012 | 0.8355 | 21.55490518 |
| Herpes zoster | rs78341235 | C | T | 0.3242 | 0.138307 | 0.0292407 | 2.25E-06 |  | -0.0011 | 0.0338 | 0.9743 | 22.37241116 |
| Herpes zoster | rs926771 | T | C | 0.07865 | -0.161931 | 0.0332555 | 1.12E-06 |  | 0.027 | 0.0587 | 0.645 | 23.71008069 |
| Herpes zoster | rs9909962 | C | T | 0.0966 | 0.147003 | 0.0311733 | 2.41E-06 |  | 0.0527 | 0.0532 | 0.3219 | 22.23754488 |
