# Supplementary material for: Association between viral infections and glioma risk: a two-sample bidirectional Mendelian randomization analysis
Source: BMC Med. 2023 Dec 5;21:487. doi: 10.1186/s12916-023-03142-9 (PMC10698979; doi:10.1186/s12916-023-03142-9)
Supplement: Supplementary file 7 — Additional file 7. Mendelian randomization results of weighted median and MR–Egger methods, sensitivity analysis, for GBM, LGG, and all-glioma on viral infection. Abbreviations: P (heterogeneity): P value of Cochrane’s Q value in heterogeneity test; P (pleiotropy): P value of MR–Egger intercept. [file 12916_2023_3142_MOESM7_ESM.docx]

**Additional file 7.** Mendelian randomization results of weighted median and MR‒Egger methods, sensitivity analysis, for GBM, LGG, and all-glioma on viral infection. Abbreviations: P (heterogeneity): P value of Cochrane’s Q value in heterogeneity test; P (pleiotropy): P value of MR‒Egger intercept.

|  |  |  |  | **Weighted median** | | | |  | **MR Egger** | | | |  | **Heterogeneity test** | | |  |
| --- | --- | --- | --- | --- | --- | --- | --- | --- | --- | --- | --- | --- | --- | --- | --- | --- | --- |
| **Exposure** | **Outcome** | **Data source** | **NSNP** | **Pvalue** | **OR** | **95%LCI** | **95%UCI** |  | **Pvalue** | **OR** | **95%LCI** | **95%UCI** |  | **P(pleiotropy)** | **Q** | **P(heterogeneity)** | **Power** |
| LGG | Herpesviral infections | FINN | 40 | 0.2873 | 0.9356 | 0.8277 | 1.0576 |  | 0.4412 | 0.1623 | 0.6865 | 1.1762 |  | 0.4401 | 32.1892 | 0.7717 | 0.0600 |
|  | Herpes zoster | FINN | 40 | 0.0569 | 1.1144 | 0.9968 | 1.2459 |  | 0.1621 | 1.1883 | 0.9374 | 1.5064 |  | 0.5438 | 35.1605 | 0.6457 | 0.9900 |
|  | Mumps | FINN | 40 | 0.3524 | 1.1176 | 0.8841 | 1.4127 |  | 0.9823 | 0.9942 | 0.5949 | 1.6613 |  | 0.8385 | 32.8809 | 0.7442 | 0.4600 |
|  | Infectious mononucleosis | FINN | 40 | 0.5153 | 0.9513 | 0.8185 | 1.1057 |  | 0.8767 | 0.9734 | 0.6938 | 1.3656 |  | 0.7730 | 45.6629 | 0.2148 | 0.1300 |
|  | Cytomegaloviral disease | FINN | 40 | 0.8417 | 1.0311 | 0.7631 | 1.3933 |  | 0.8019 | 0.9110 | 0.4418 | 1.8782 |  | 0.4999 | 47.4833 | 0.1653 | 1.0000 |
|  | COVID-19 hospitalized | COVID-19 HGI | 41 | 0.7826 | 0.9952 | 0.9619 | 1.0297 |  | 0.9279 | 0.9966 | 0.9272 | 1.0713 |  | 0.6569 | 42.2952 | 0.3722 | 0.0800 |
|  | HPV | Suhre | 4 | 0.4145 | 0.8171 | 0.5030 | 1.3274 |  | 0.6337 | 12.4355 | 0.0017 | 88708.7281 |  | 0.5985 | 7.5527 | 0.0562 | 0.9200 |
|  | Acute poliomyelitis | FINN | 40 | 0.5052 | 1.1189 | 0.8041 | 1.5570 |  | 0.5992 | 0.8172 | 0.3873 | 1.7243 |  | 0.3549 | 43.2584 | 0.2944 | 1.0000 |
|  | HIV disease | FINN | 40 | 0.1030 | 0.8056 | 0.6213 | 1.0446 |  | 0.8657 | 1.0509 | 0.5935 | 1.8610 |  | 0.5228 | 35.7217 | 0.6202 | 1.0000 |
|  | Viral hepatitis | FINN | 40 | 0.9826 | 1.0017 | 0.8624 | 1.1635 |  | 0.3207 | 1.2785 | 0.9085 | 1.7990 |  | 0.1138 | 48.7104 | 0.1370 | 0.1400 |
|  | Rubella | FINN | 40 | 0.4681 | 0.9226 | 0.7421 | 1.1470 |  | 0.3207 | 0.7840 | 0.4881 | 1.2593 |  | 0.5938 | 40.3789 | 0.4092 | 1.0000 |
|  | Measles | FINN | 40 | 0.2424 | 1.2435 | 0.8629 | 1.7921 |  | 0.2870 | 0.6330 | 0.2761 | 1.4515 |  | 0.2759 | 42.4046 | 0.3264 | 0.0900 |
| GBM | Herpesviral infections | FINN | 32 | 0.5823 | 1.0454 | 0.8925 | 1.2245 |  | 0.5823 | 1.0651 | 0.8045 | 1.4102 |  | 0.4401 | 32.1892 | 0.7717 | 0.7400 |
|  | Herpes zoster | FINN | 32 | 0.8385 | 0.9852 | 0.8532 | 1.1375 |  | 0.2596 | 0.8650 | 0.6755 | 1.1077 |  | 0.5438 | 35.1605 | 0.6457 | 0.0900 |
|  | Mumps | FINN | 32 | 0.4819 | 0.9038 | 0.6819 | 1.1980 |  | 0.2414 | 1.3854 | 0.8117 | 2.3644 |  | 0.8385 | 32.8809 | 0.7442 | 1.0000 |
|  | Infectious mononucleosis | FINN | 32 | 0.7835 | 0.9727 | 0.7986 | 1.1848 |  | 0.0565 | 0.6769 | 0.4603 | 0.9954 |  | 0.7730 | 45.6629 | 0.2148 | 0.1400 |
|  | Cytomegaloviral disease | FINN | 32 | 0.6092 | 0.9039 | 0.6137 | 1.3315 |  | 0.7394 | 0.8904 | 0.4522 | 1.7532 |  | 0.8721 | 29.6589 | 0.5350 | 1.0000 |
|  | COVID-19 hospitalized | COVID-19 HGI | 32 | 0.6892 | 1.0086 | 0.9673 | 1.0516 |  | 0.6465 | 1.0186 | 0.9421 | 1.1014 |  | 0.6569 | 42.2952 | 0.3722 | 0.0600 |
|  | HPV | Suhre | 5 | 0.9715 | 0.9917 | 0.6267 | 1.5693 |  | 0.7679 | 0.8799 | 0.4049 | 1.9122 |  | 0.8836 | 3.5118 | 0.4761 | 0.5800 |
|  | Acute poliomyelitis | FINN | 32 | 0.1349 | 0.7376 | 0.4949 | 1.0993 |  | 0.0120 | 0.3655 | 0.1748 | 0.7645 |  | 0.3549 | 43.2584 | 0.2944 | 1.0000 |
|  | HIV disease | FINN | 32 | 0.9595 | 1.0084 | 0.7299 | 1.3931 |  | 0.3178 | 1.3606 | 0.7512 | 2.4642 |  | 0.5228 | 35.7217 | 0.6202 | 0.1000 |
|  | Viral hepatitis | FINN | 32 | 0.6185 | 1.0479 | 0.8716 | 1.2599 |  | 0.6939 | 1.0706 | 0.7646 | 1.4991 |  | 0.1138 | 48.7104 | 0.1370 | 0.3200 |
|  | Rubella | FINN | 32 | 0.5428 | 0.9152 | 0.6880 | 1.2175 |  | 0.7923 | 0.9302 | 0.5453 | 1.5867 |  | 0.5938 | 40.3789 | 0.4092 | 0.9100 |
|  | Measles | FINN | 32 | 0.8701 | 1.0389 | 0.6572 | 1.6423 |  | 0.7511 | 1.1452 | 0.4993 | 2.6266 |  | 0.2759 | 42.4046 | 0.3264 | 0.0800 |
| all-glioma | Herpesviral infections | FINN | 39 | 0.1739 | 0.8860 | 0.7442 | 1.0549 |  | 0.2254 | 0.7796 | 0.5248 | 1.1582 |  | 0.3013 | 46.6896 | 0.1575 | 0.4300 |
|  | Herpes zoster | FINN | 39 | 0.7695 | 0.9763 | 0.8290 | 1.1499 |  | 0.5709 | 0.8915 | 0.6014 | 1.3215 |  | 0.9536 | 57.7177 | 0.0211 | 0.9900 |
|  | Mumps | FINN | 39 | 0.2222 | 0.8179 | 0.5758 | 1.1617 |  | 0.6271 | 0.8326 | 0.4001 | 1.7327 |  | 0.8441 | 42.9577 | 0.2671 | 0.9800 |
|  | Infectious mononucleosis | FINN | 39 | 0.6445 | 1.0503 | 0.8493 | 1.2988 |  | 0.8136 | 0.9461 | 0.5987 | 1.4951 |  | 0.5825 | 46.7069 | 0.1571 | 0.6700 |
|  | Cytomegaloviral disease | FINN | 39 | 0.6703 | 1.0977 | 0.7068 | 1.7048 |  | 0.3150 | 1.5641 | 0.6614 | 3.6990 |  | 0.3050 | 34.0475 | 0.6528 | 0.1000 |
|  | COVID-19 hospitalized | COVID-19 HGI | 39 | 0.6879 | 1.0099 | 0.9624 | 1.0597 |  | 0.3806 | 1.0452 | 0.9480 | 1.1524 |  | 0.2509 | 32.4808 | 0.7221 | 0.0700 |
|  | HPV | Suhre | 4 | 0.5498 | 0.8320 | 0.4554 | 1.5201 |  | 0.7582 | 1.8870 | 0.0552 | 64.4673 |  | 0.7426 | 8.2436 | 0.0412 | 0.0600 |
|  | Acute poliomyelitis | FINN | 39 | 0.6069 | 0.8845 | 0.5596 | 1.3982 |  | 0.2807 | 0.5902 | 0.2297 | 1.5169 |  | 0.5020 | 37.6550 | 0.4853 | 1.0000 |
|  | HIV disease | FINN | 39 | 0.6384 | 1.0917 | 0.7572 | 1.5738 |  | 0.4566 | 1.3361 | 0.6281 | 2.8420 |  | 0.4939 | 34.3965 | 0.6368 | 0.3000 |
|  | Viral hepatitis | FINN | 39 | 0.8118 | 0.9759 | 0.7980 | 1.1934 |  | 0.5986 | 1.1184 | 0.7400 | 1.6905 |  | 0.5394 | 31.8969 | 0.7466 | 0.0700 |
|  | Rubella | FINN | 39 | 0.5419 | 0.9152 | 0.6884 | 1.2167 |  | 0.7158 | 0.8919 | 0.4841 | 1.6432 |  | 0.9101 | 29.8379 | 0.8251 | 0.8300 |
|  | Measles | FINN | 39 | 0.8336 | 0.9449 | 0.5572 | 1.6025 |  | 0.3679 | 1.7270 | 0.5335 | 5.5903 |  | 0.4527 | 46.3376 | 0.1662 | 0.9900 |
