# Supplementary material for: Association between viral infections and glioma risk: a two-sample bidirectional Mendelian randomization analysis
Source: BMC Med. 2023 Dec 5;21:487. doi: 10.1186/s12916-023-03142-9 (PMC10698979; doi:10.1186/s12916-023-03142-9)
Supplement: Supplementary file 8 — Additional file 8. Leave-one-out plots, forest plots, and scatter plots in the reverse MR estimate. [file 12916_2023_3142_MOESM8_ESM.docx]

**Additional file 8.** Leave-one-out plots, forest plots, and scatter plots in the reverse MR estimate

**Figure S1.** The leave-one-out plot, forest plot, and scatter plot for the association of Herpes zoster and LGG in the reverse analysis. Data from FINN.


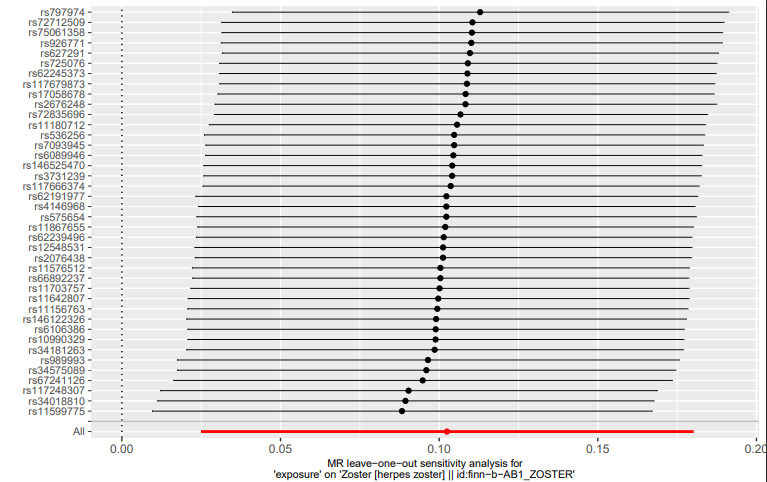


**
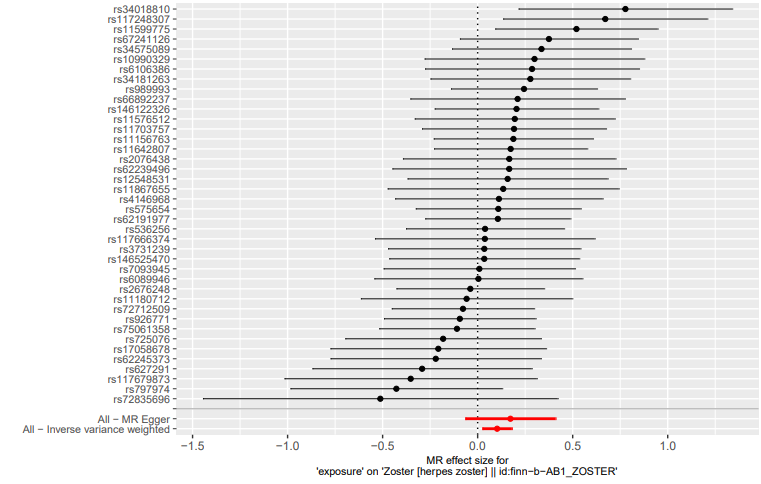
**

**
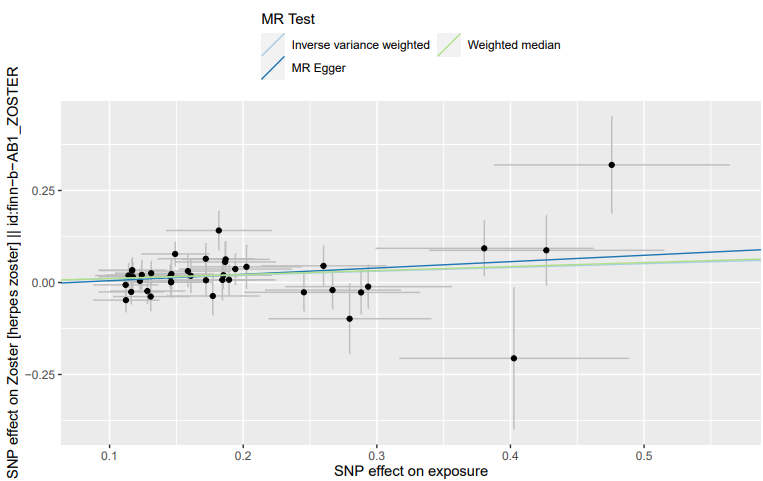
**

**Figure S2.** The leave-one-out plot, forest plot, and scatter plot for the association of mumps virus infection and LGG in the reverse analysis. Data from FINN.


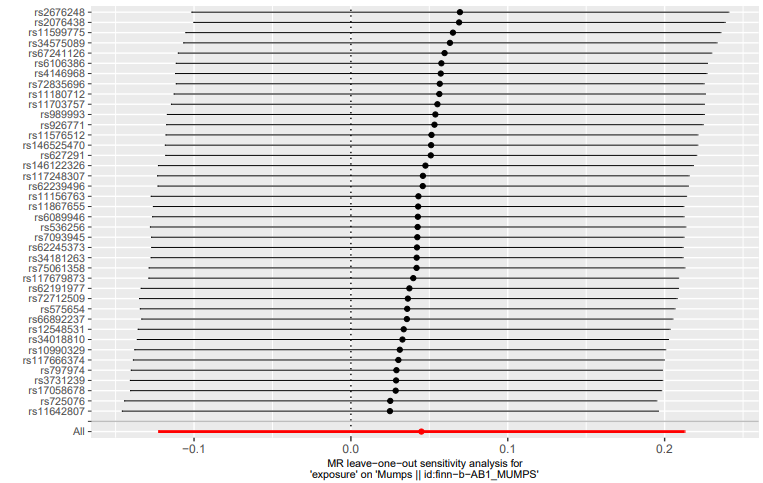


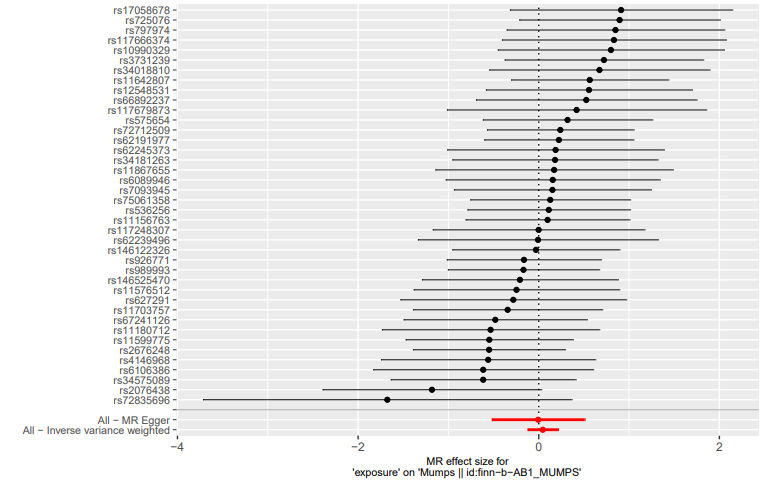


**
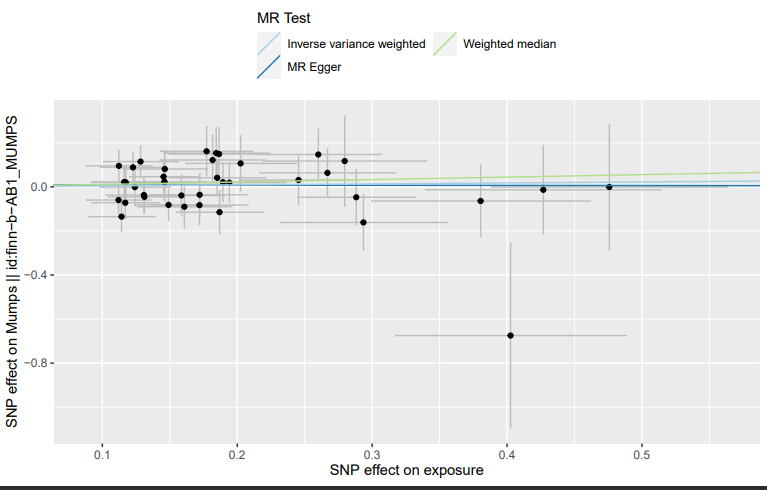
**

**Figure S3.** The leave-one-out plot, forest plot, and scatter plot for the association of HSV infection and LGG in the reverse analysis. Data from FINN.


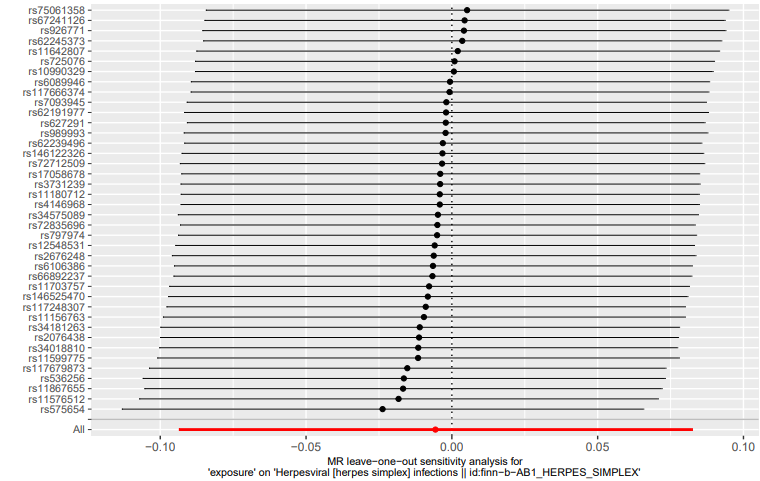


**
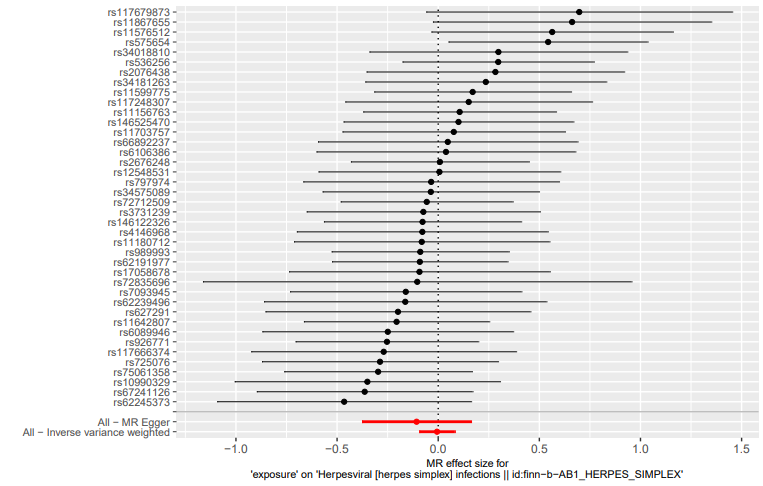
**

**
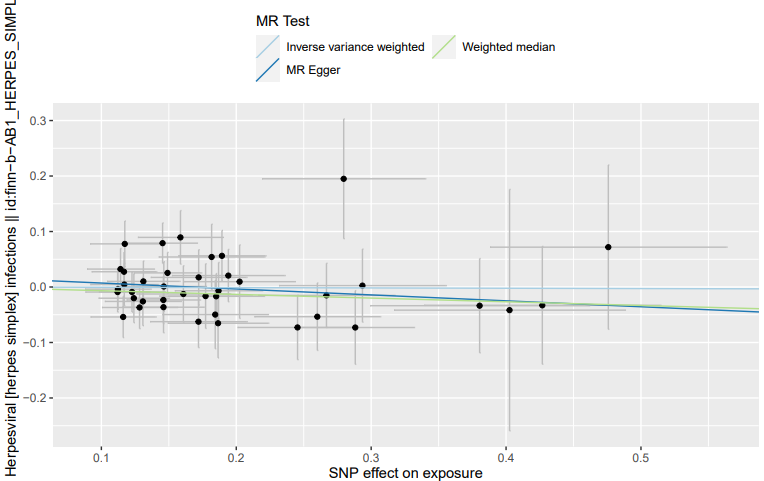
**

**Figure S4.** The leave-one-out plot, forest plot, and scatter plot for the association of HPV infection and LGG in the reverse analysis. Data from SUHRE.


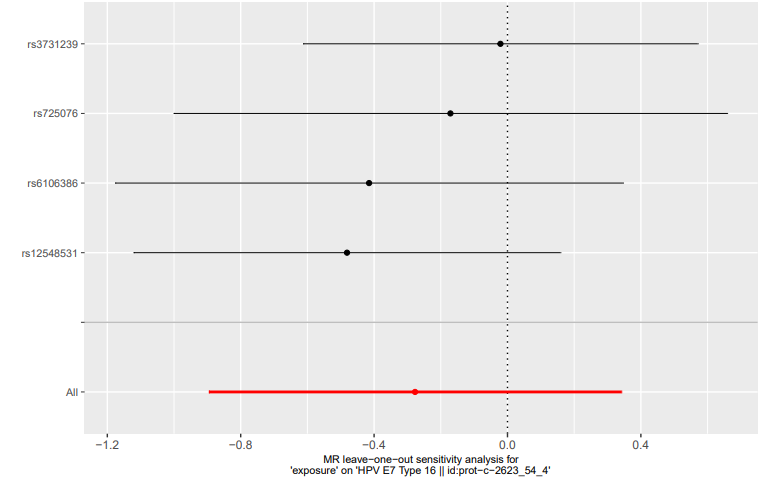


**
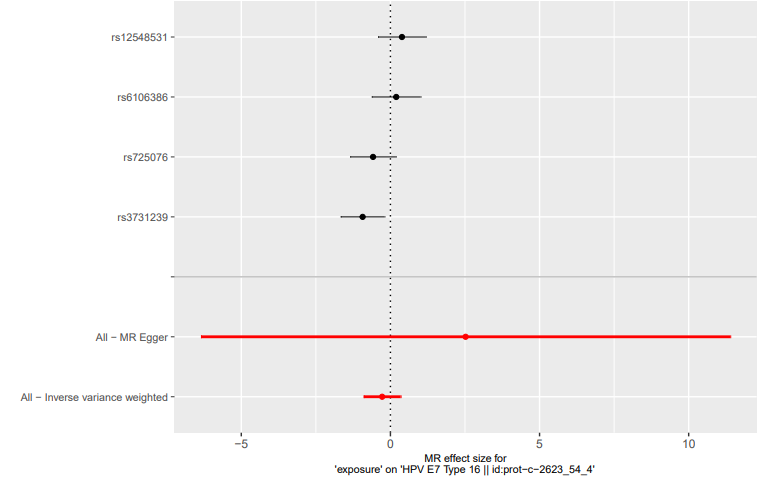
**

**
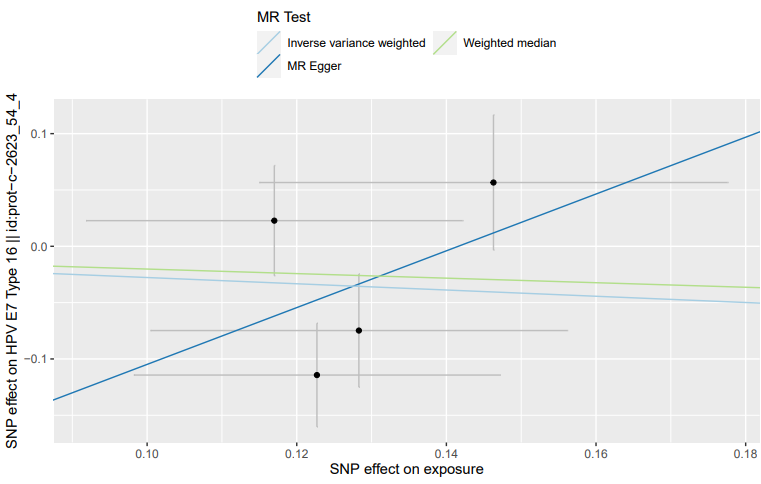
**

**Figure S5.** The leave-one-out plot, forest plot, and scatter plot for the association of EBV infection and LGG in the reverse analysis. Data from FINN.


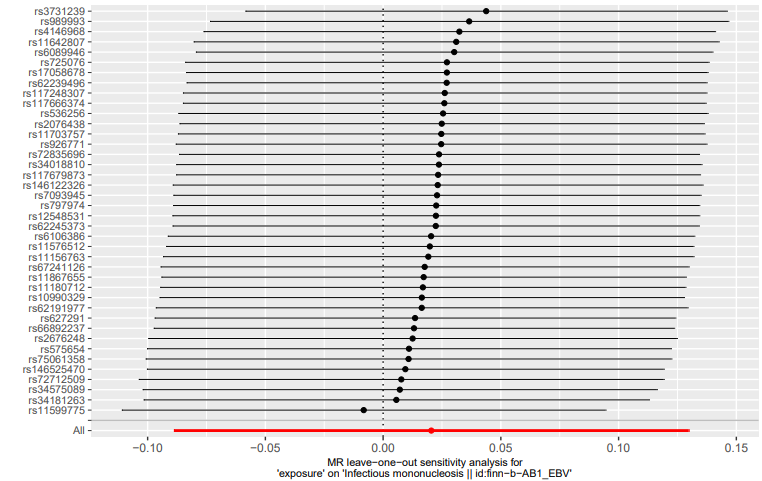


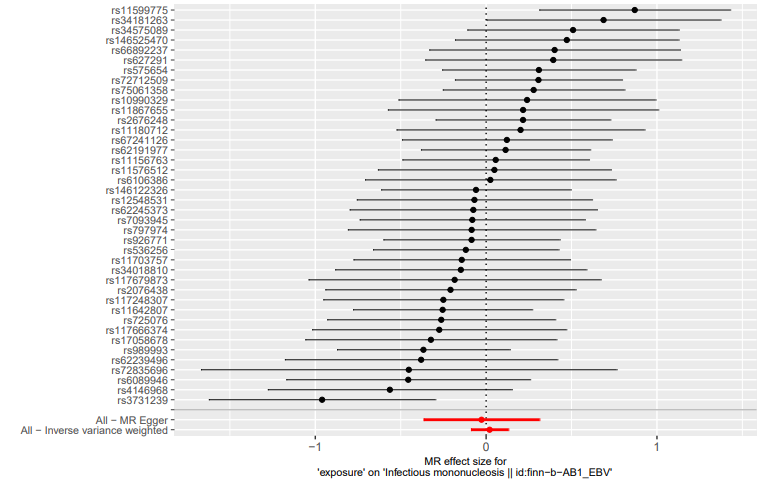


**
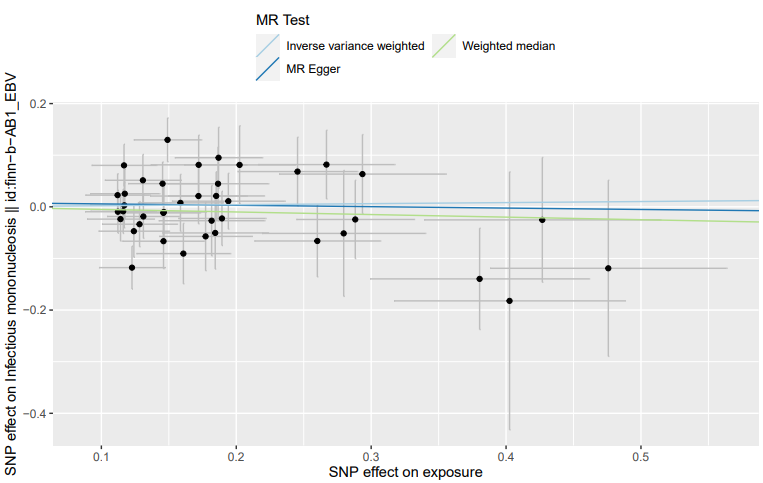
**

**Figure S6.** The leave-one-out plot, forest plot, and scatter plot for the association of COVID-19 infection and LGG in the reverse analysis. Data from COVID-19 HGI.


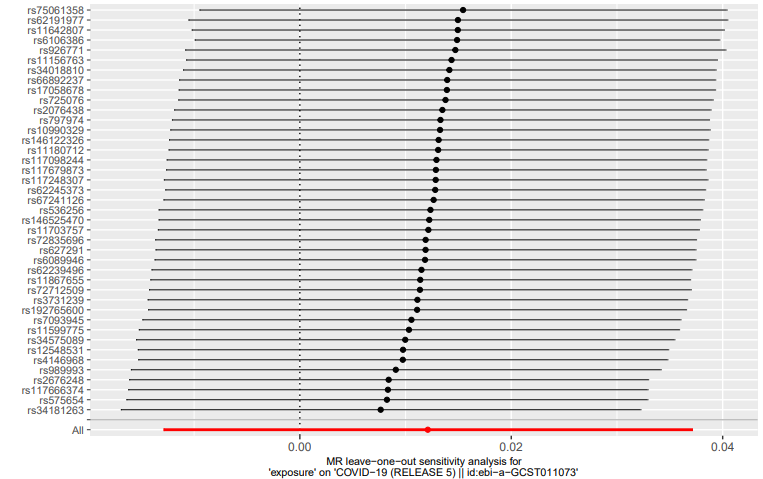


**
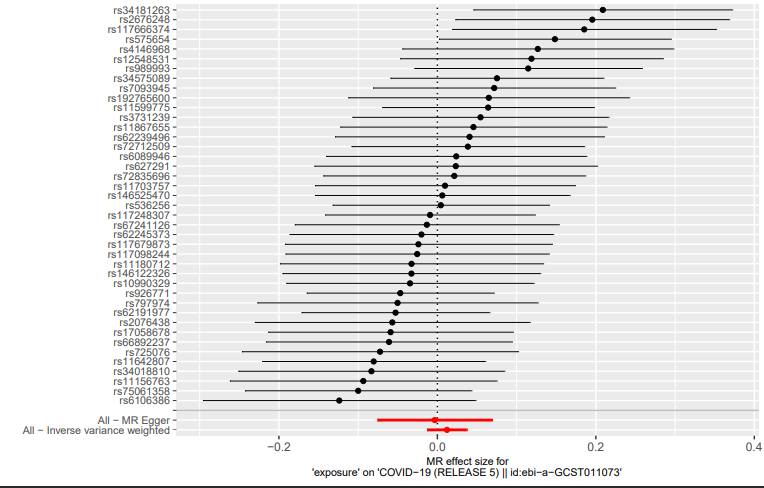
**

**
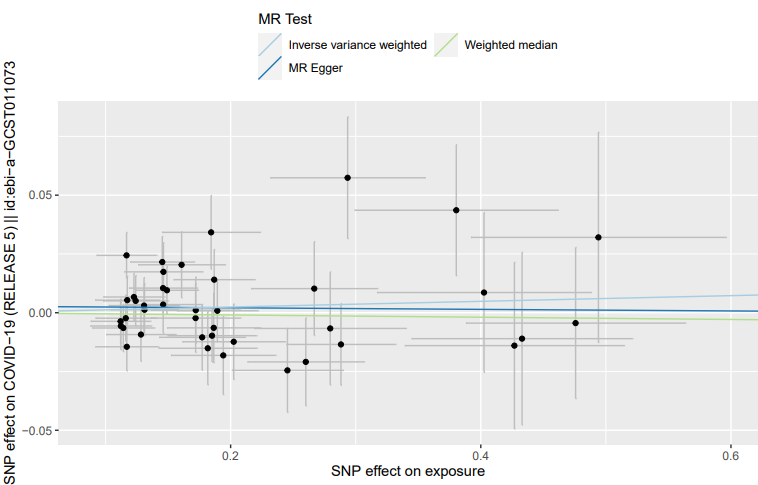
**

**Figure S7.** The leave-one-out plot, forest plot, and scatter plot for the association of HCMV infection and LGG in the reverse analysis. Data from FINN.


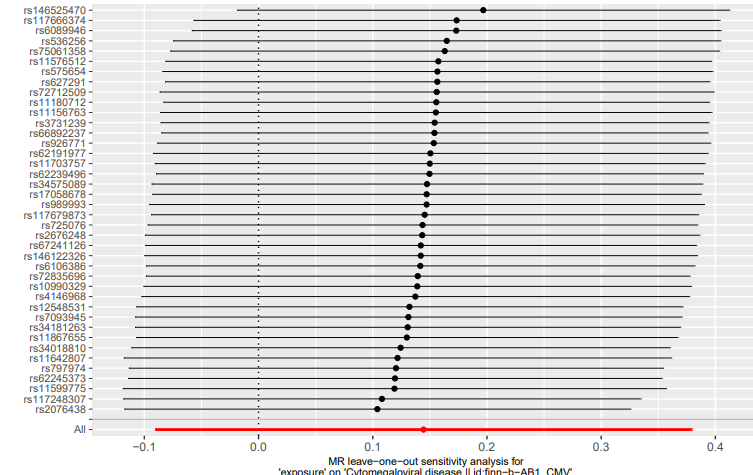


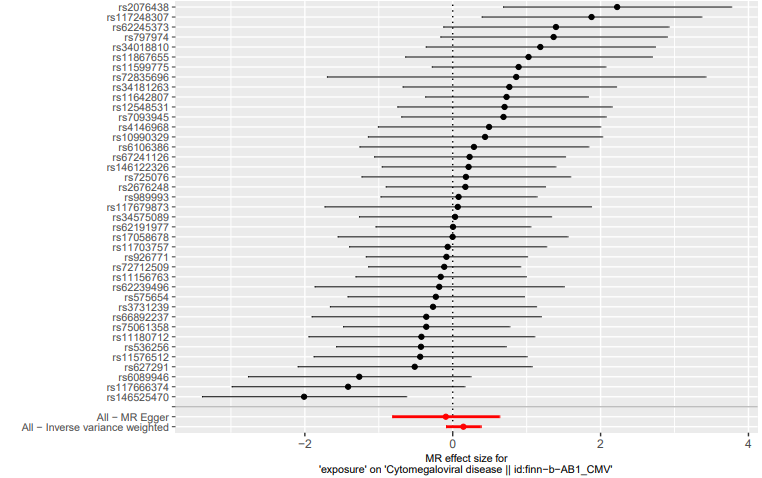


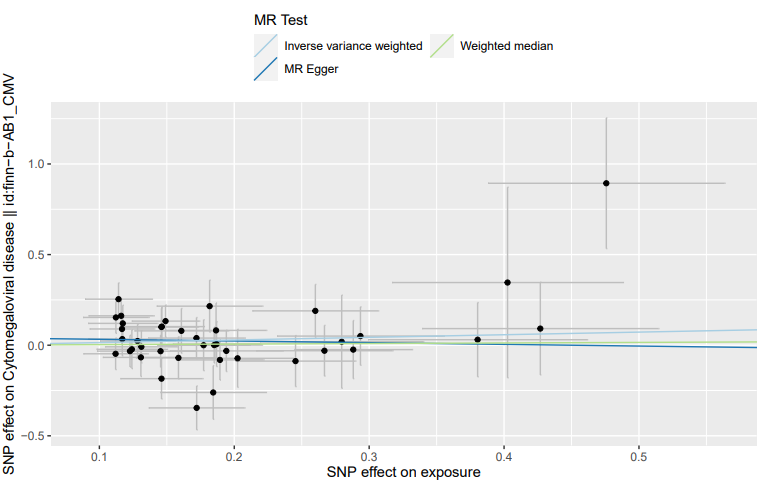


**Figure S8.** The leave-one-out plot, forest plot, and scatter plot for the association of Hepatitis infection and LGG in the reverse analysis. Data from FINN.


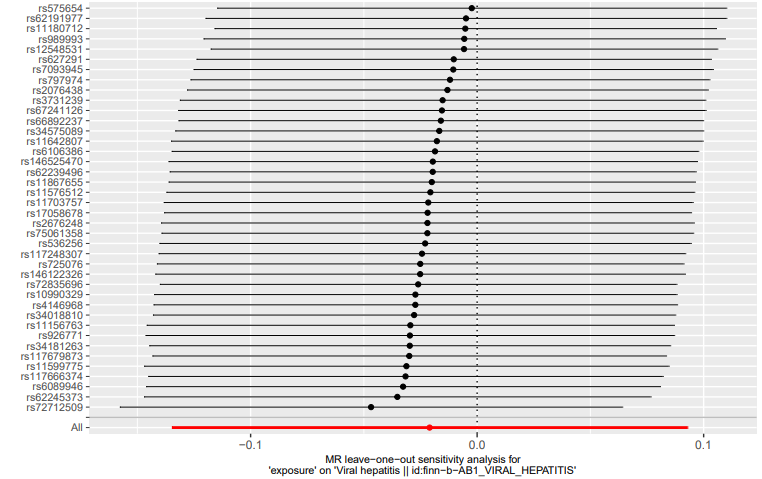


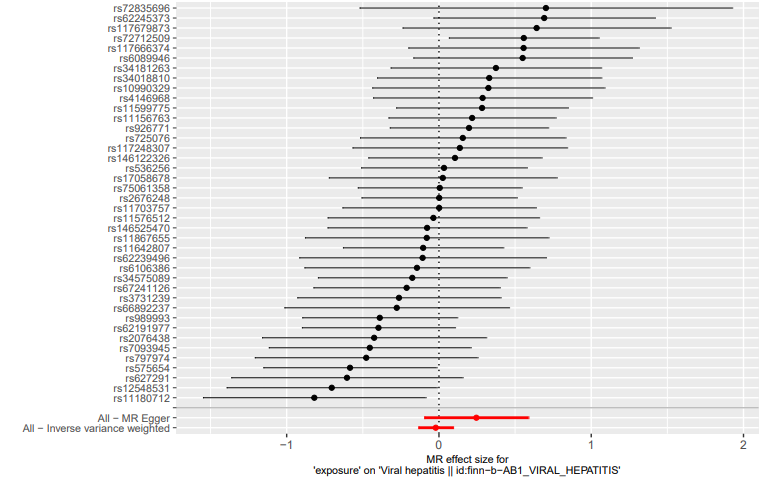


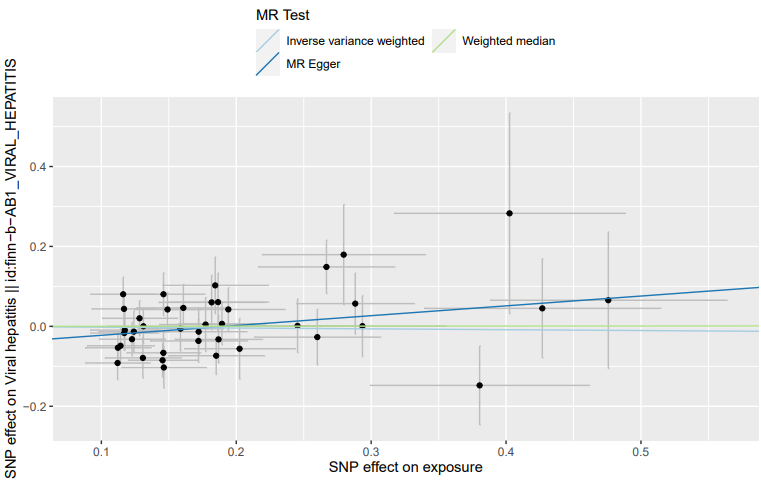


**Figure S9.** The leave-one-out plot, forest plot, and scatter plot for the association of HIV infection and LGG in the reverse analysis. Data from FINN.


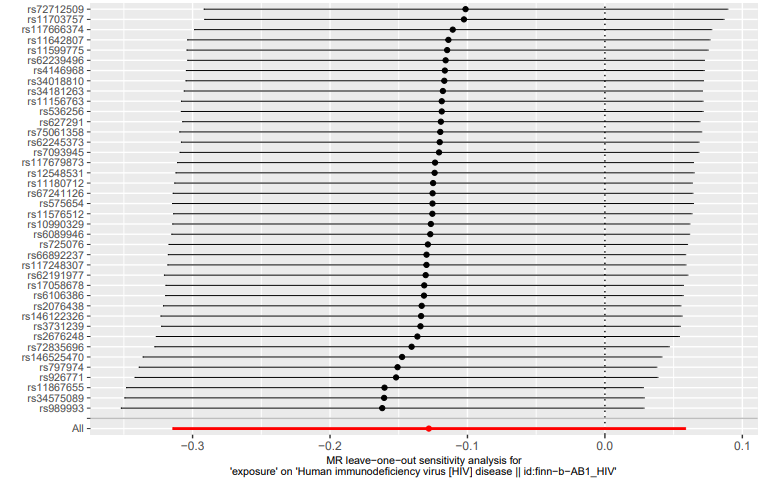


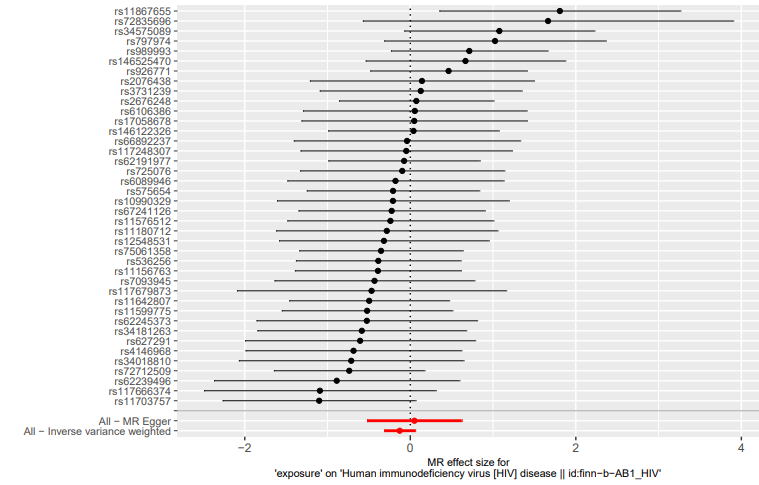


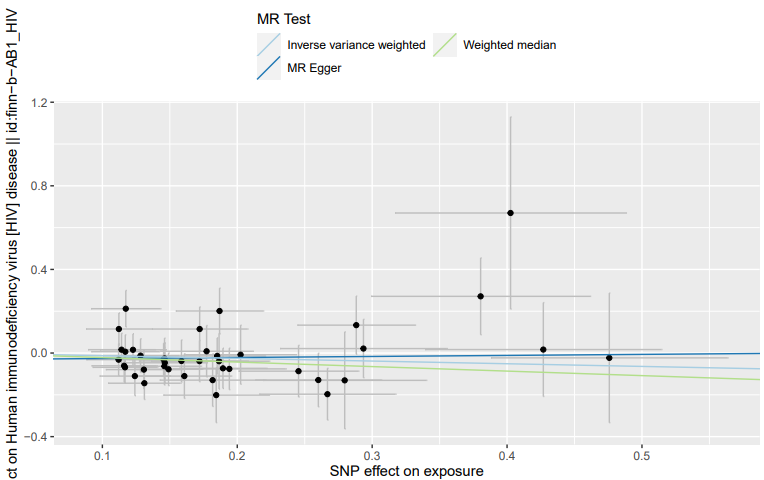


**Figure S10.** The leave-one-out plot, forest plot, and scatter plot for the association of measles infection and LGG in the reverse analysis. Data from FINN.


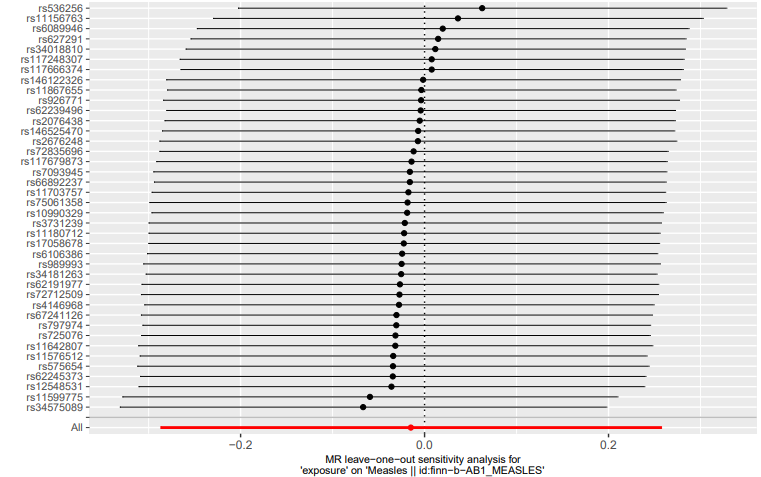


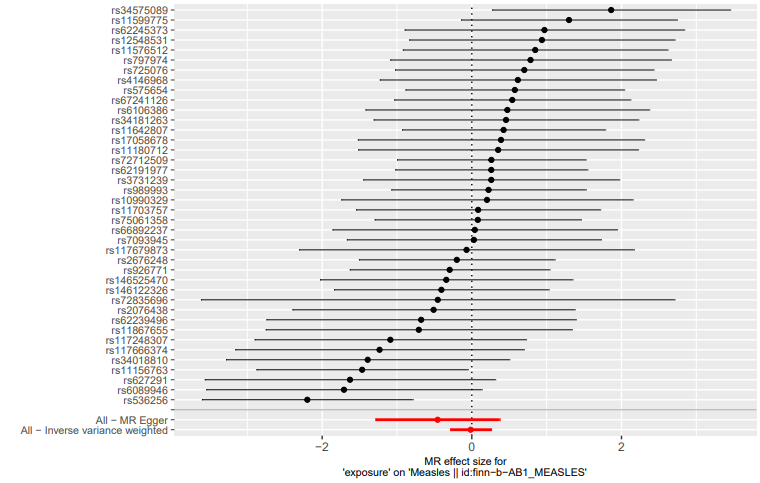


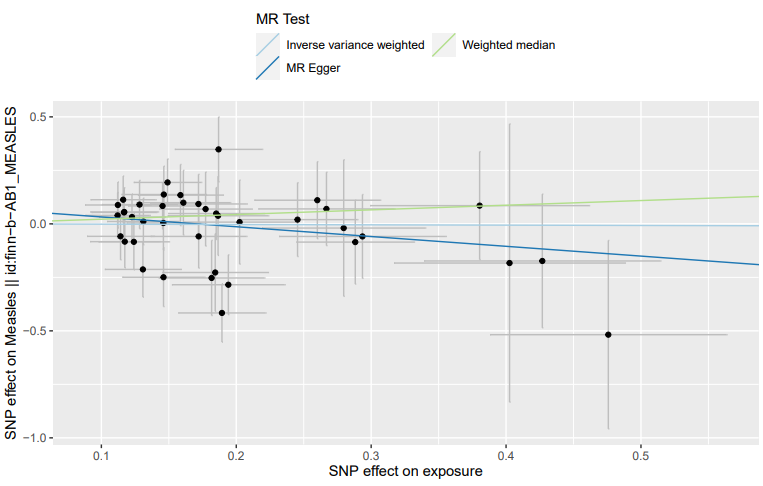


**Figure S11.** The leave-one-out plot, forest plot, and scatter plot for the association of Poliovirus infection and LGG in the reverse analysis. Data from FINN.


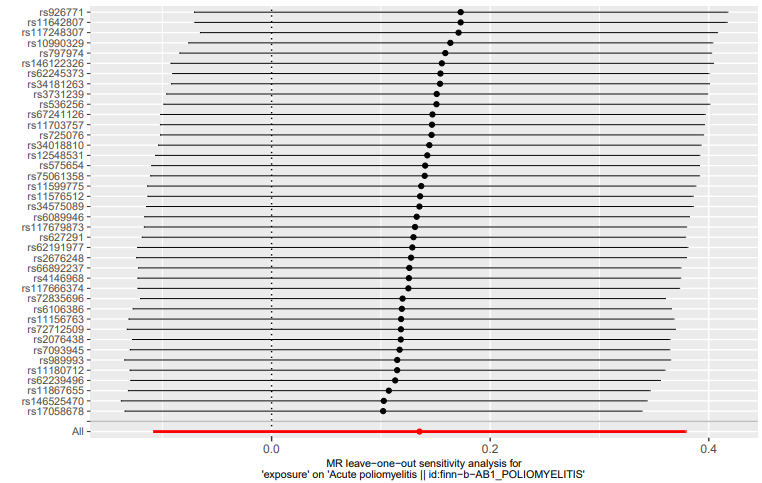


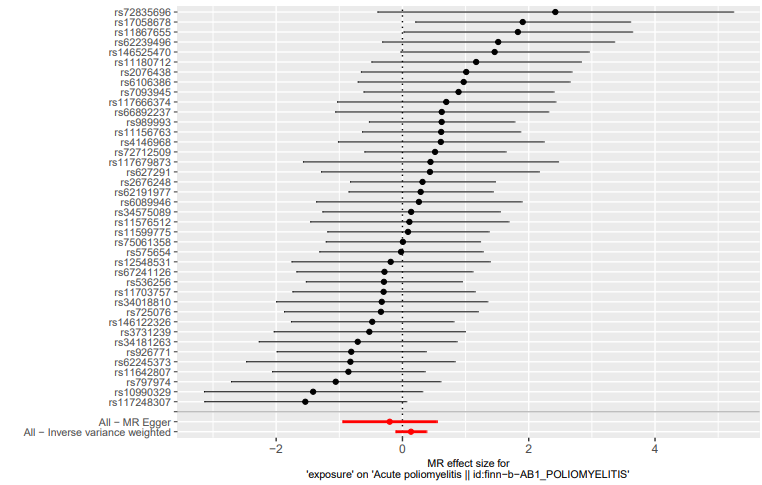


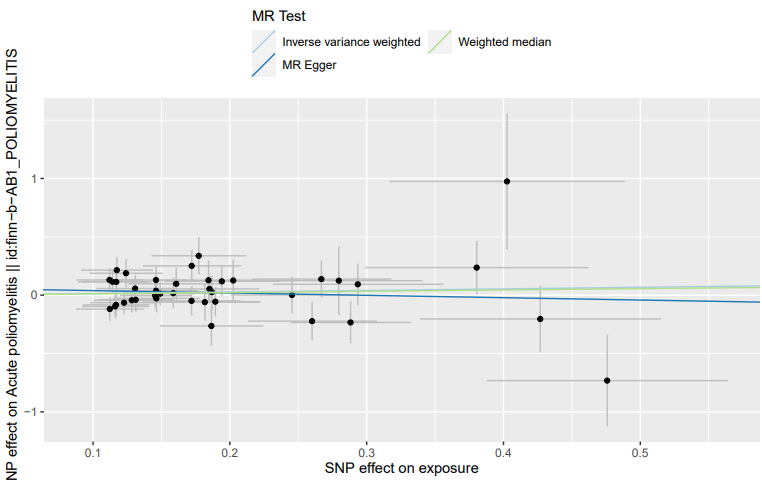


**Figure S12.** The leave-one-out plot, forest plot, and scatter plot for the association of Rubella infection and LGG in the reverse analysis. Data from FINN.


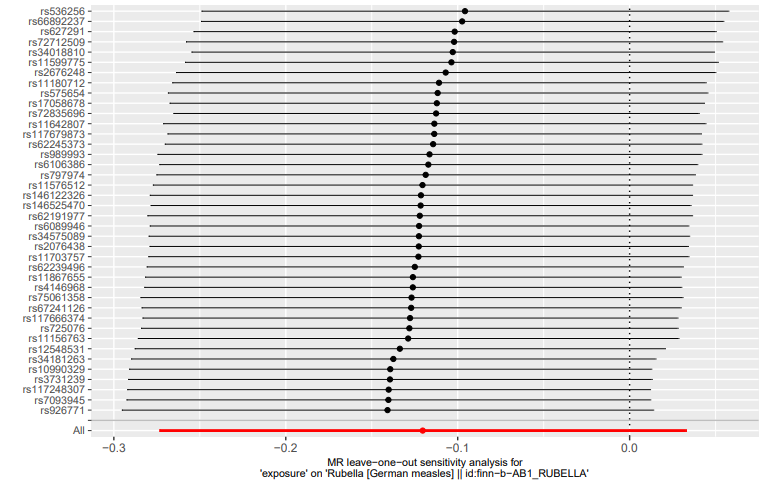


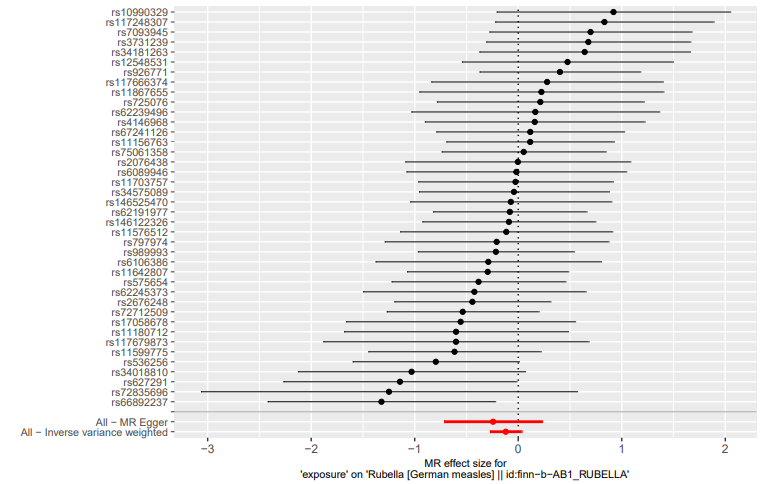


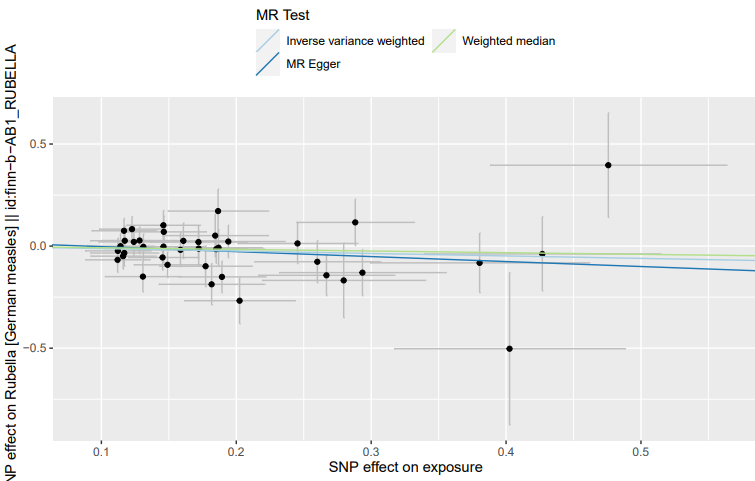


**Figure S13.** The leave-one-out plot, forest plot, and scatter plot for the association of Herpes zoster and GBM in the reverse analysis. Data from FINN.


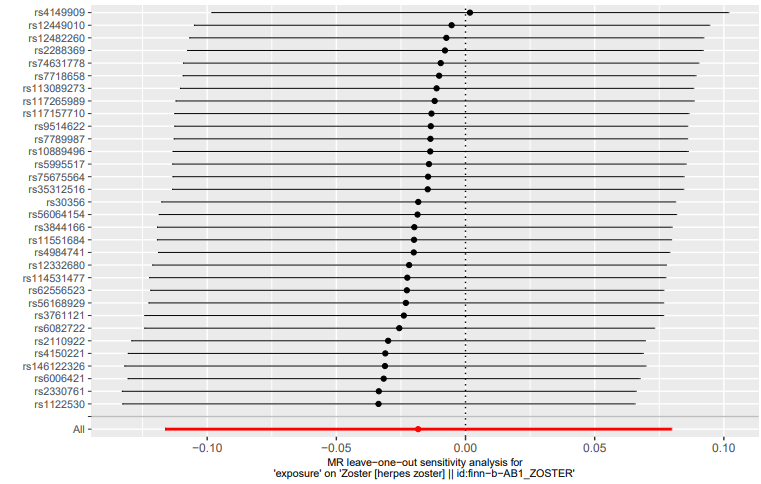


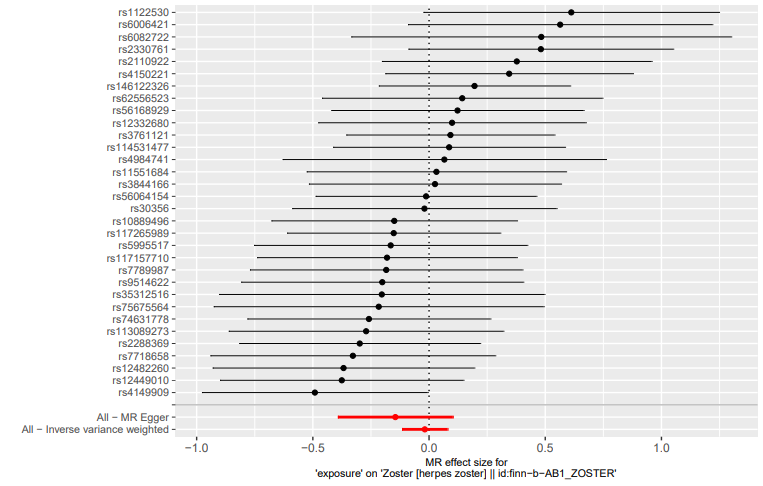


**
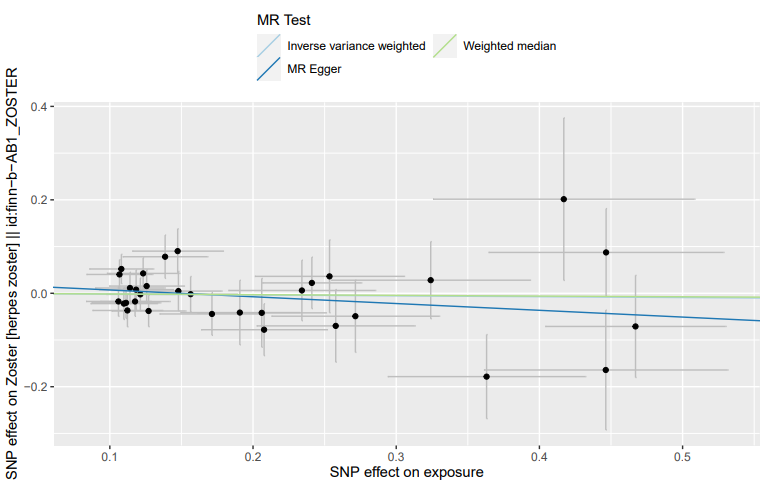
**

**Figure S14.** The leave-one-out plot, forest plot, and scatter plot for the association of mumps virus infection and GBM in the reverse analysis. Data from FINN.


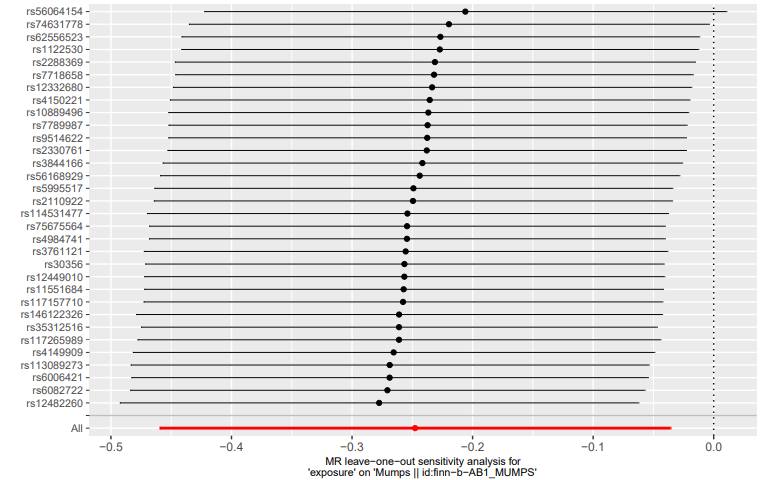


**
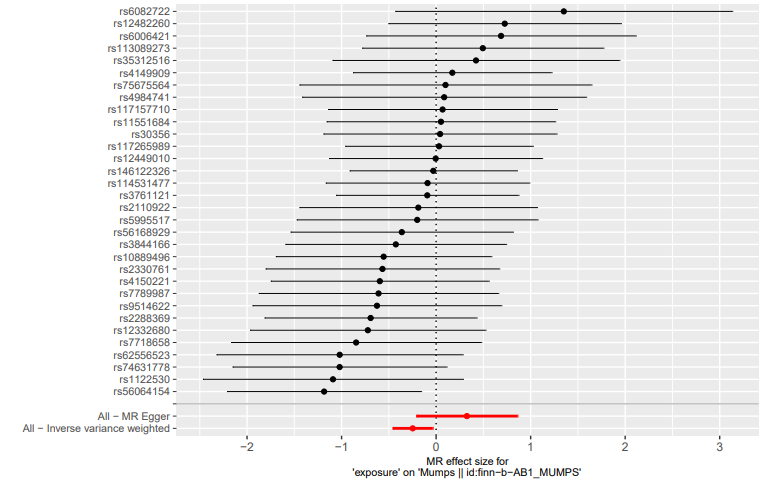
**

**
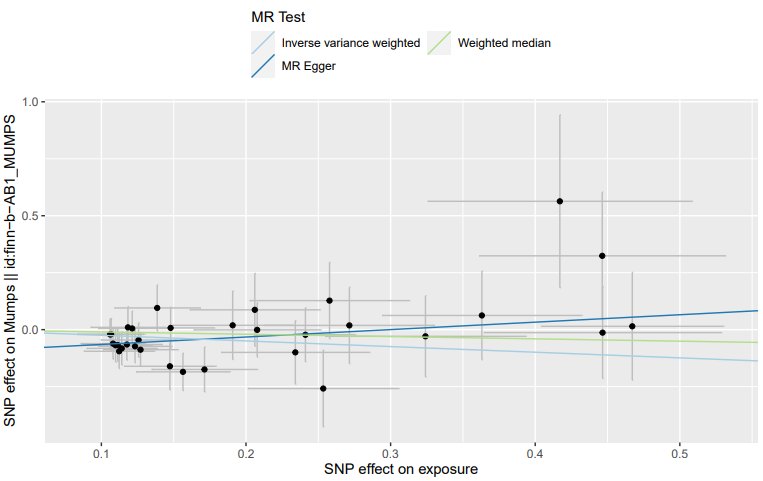
**

**Figure S15.** The leave-one-out plot, forest plot, and scatter plot for the association of HSV infection and GBM in the reverse analysis. Data from FINN.


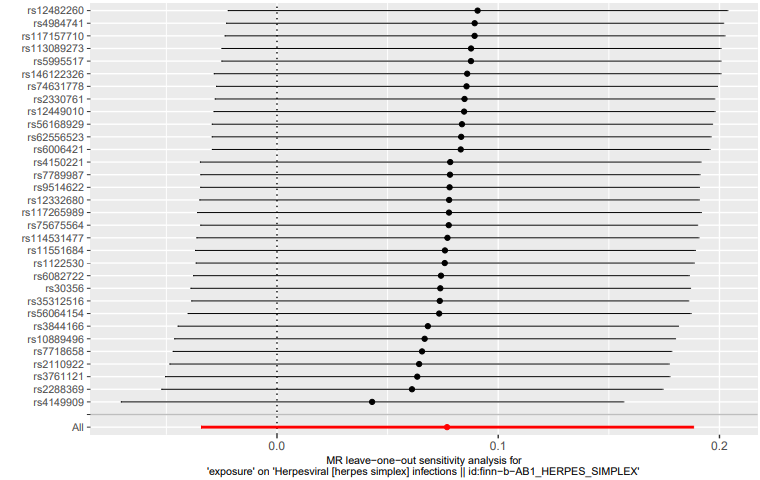


**
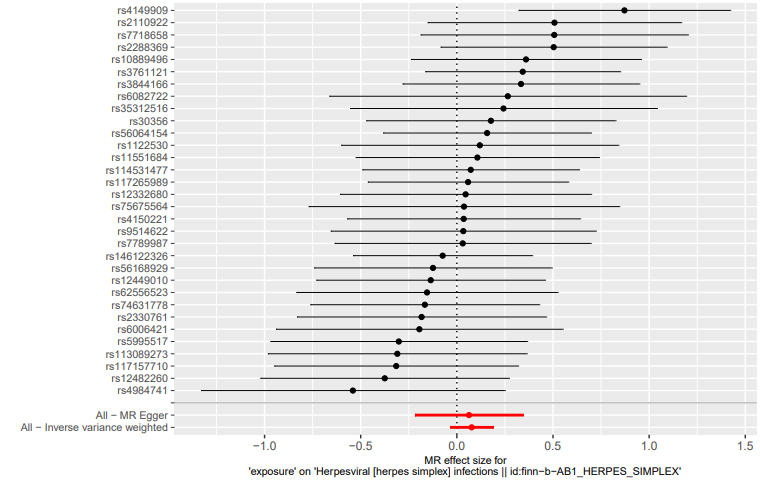
**

**
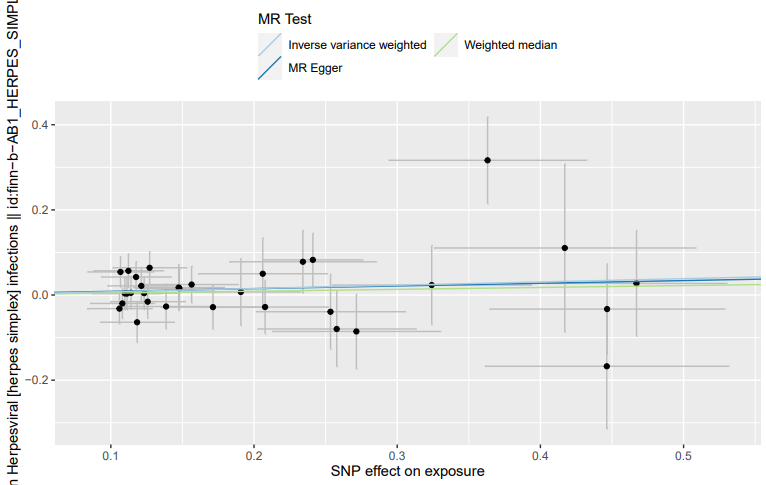
**

**Figure S16.** The leave-one-out plot, forest plot, and scatter plot for the association of HPV infection and GBM in the reverse analysis. Data from SUHRE.

**
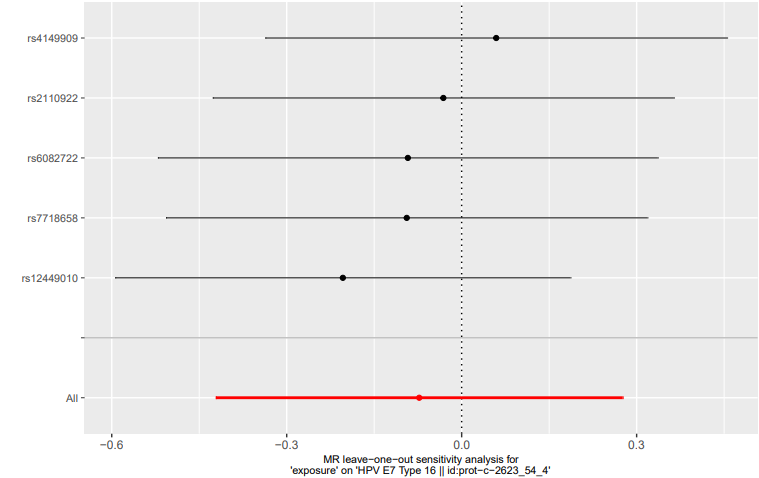
**

**
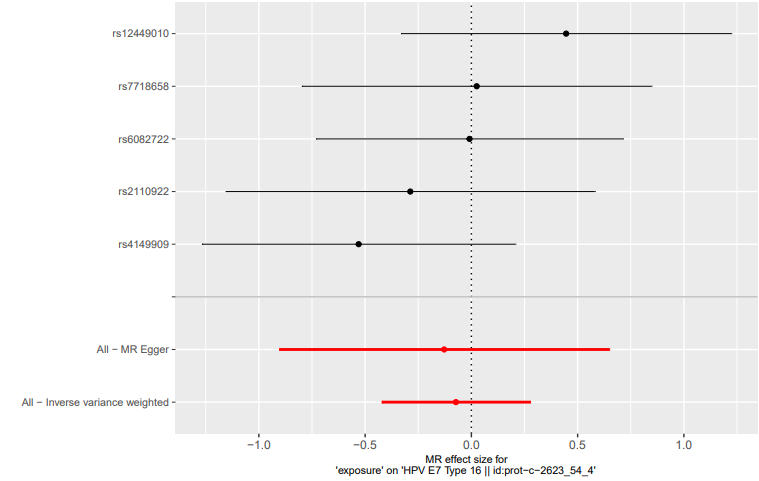
**

**
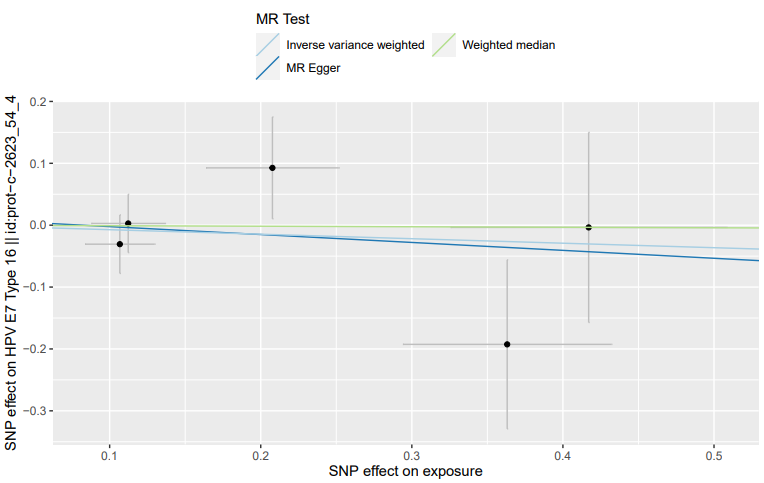
**

**Figure S17.** The leave-one-out plot, forest plot, and scatter plot for the association of EBV infection and GBM in the reverse analysis. Data from FINN.

**
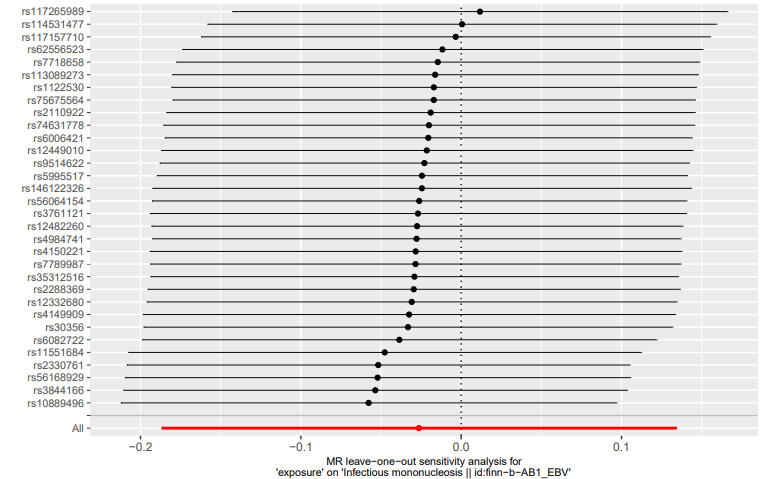
**

**
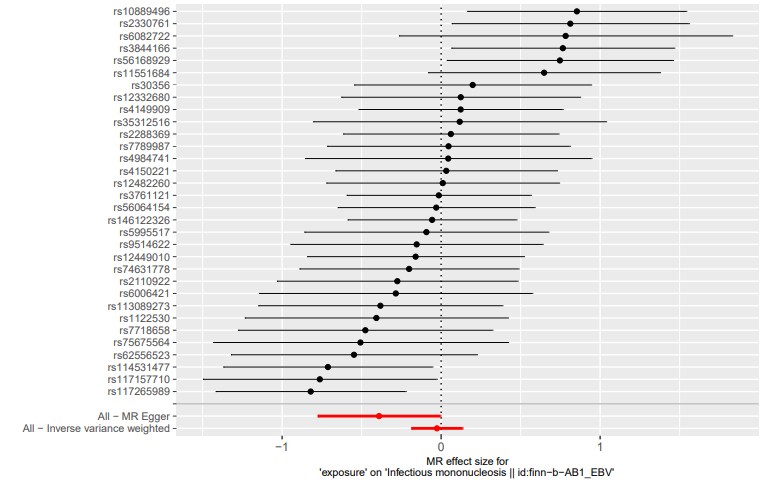
**

**
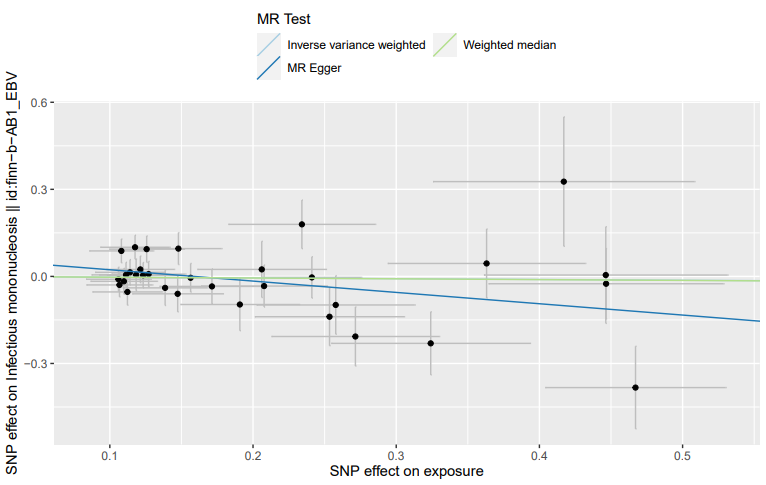
**

**Figure S18.** The leave-one-out plot, forest plot, and scatter plot for the association of COVID-19 infection and GBM in the reverse analysis. Data from COVID-19 HGI.

**
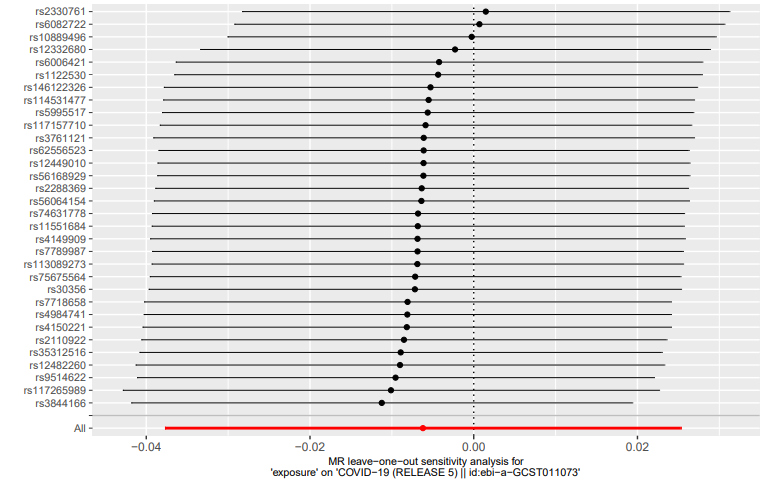
**

**
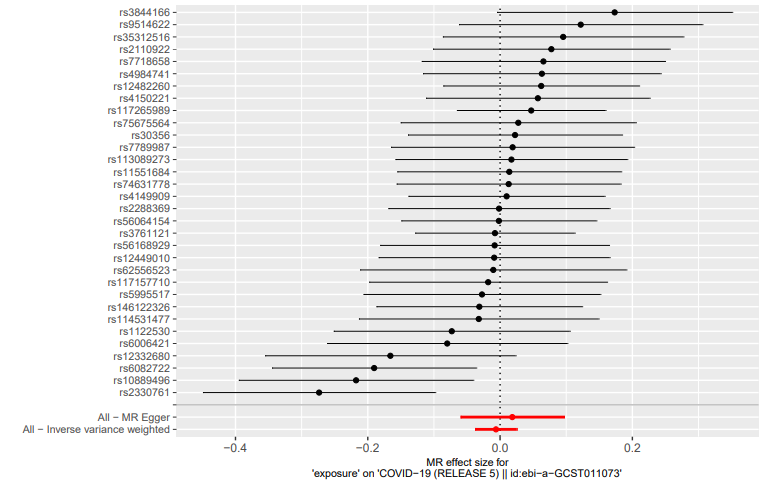
**

**
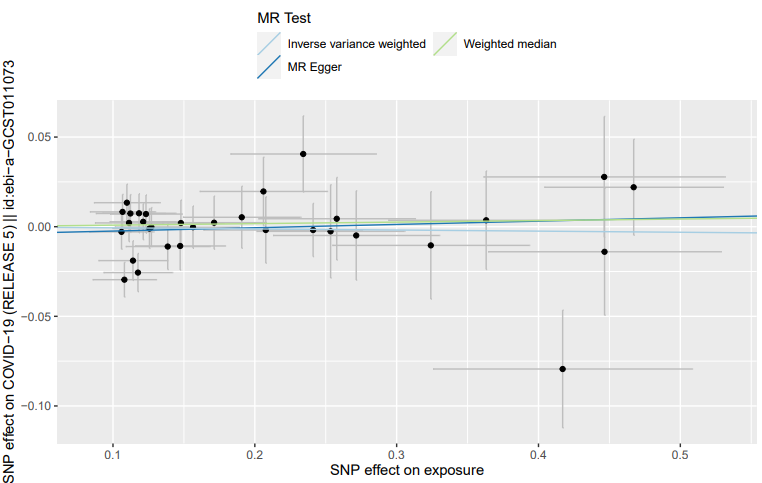
**

**Figure S19.** The leave-one-out plot, forest plot, and scatter plot for the association of HCMV infection and GBM in the reverse analysis. Data from FINN.


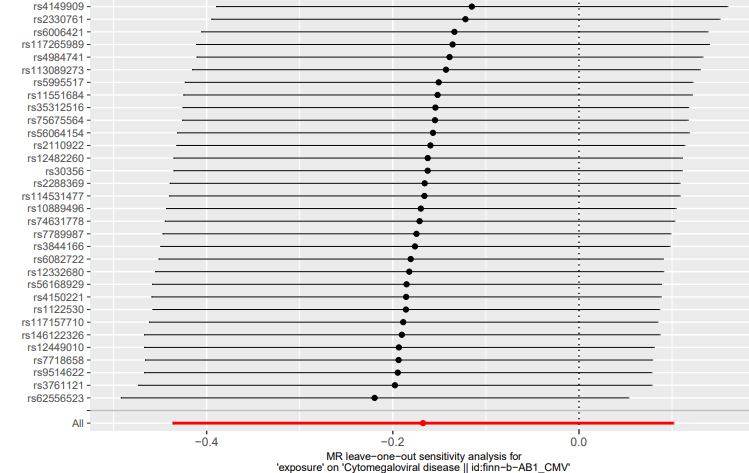


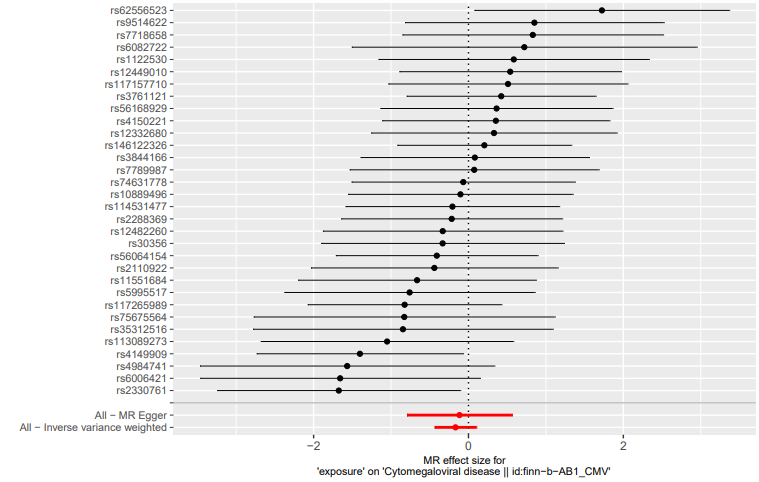


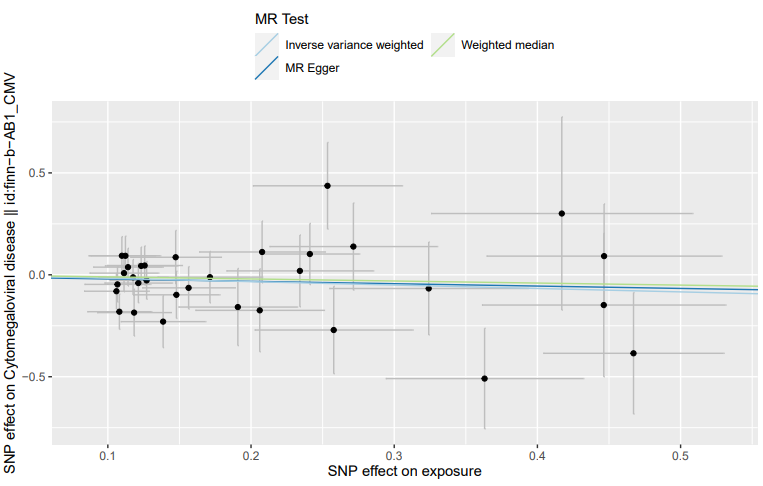


**Figure S20.** The leave-one-out plot, forest plot, and scatter plot for the association of Hepatitis infection and GBM in the reverse analysis. Data from FINN.


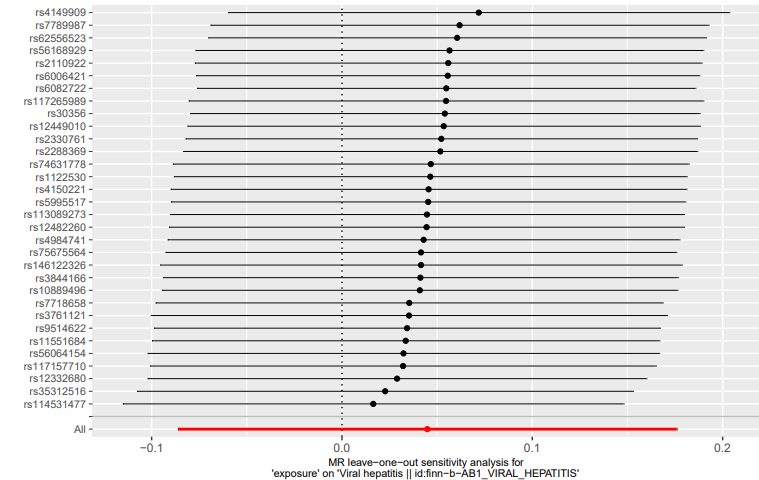


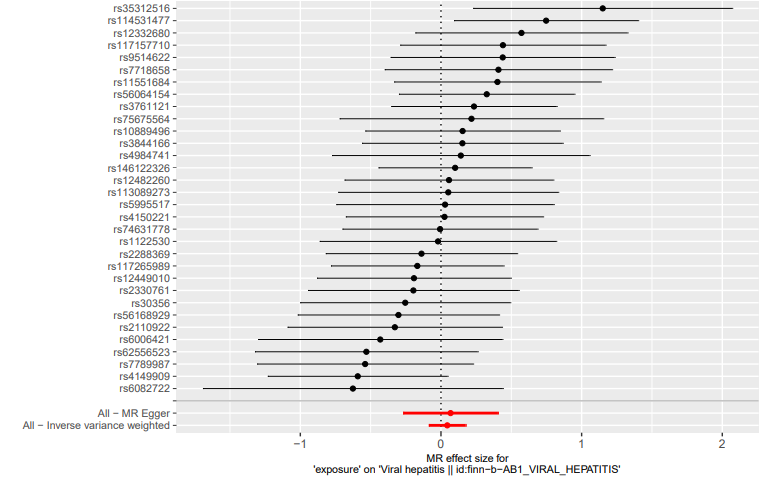


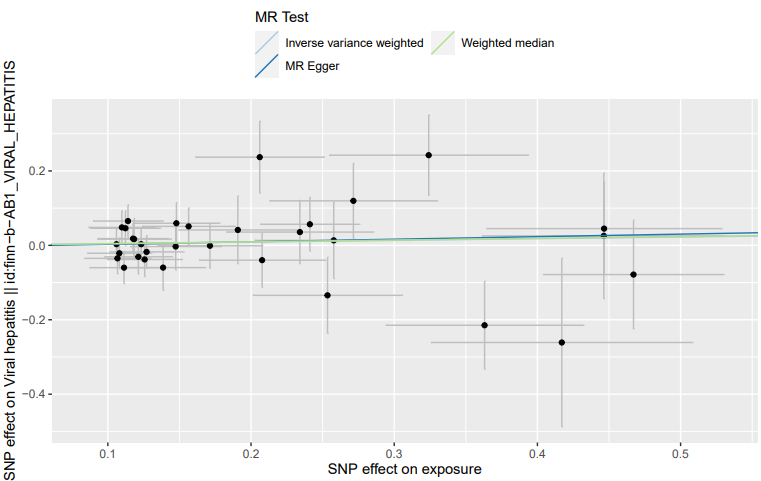


**Figure S21.** The leave-one-out plot, forest plot, and scatter plot for the association of HIV infection and GBM in the reverse analysis. Data from FINN.


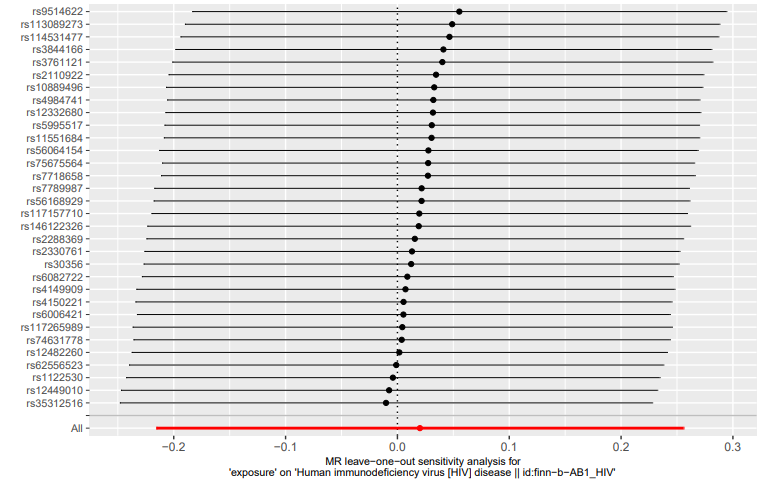


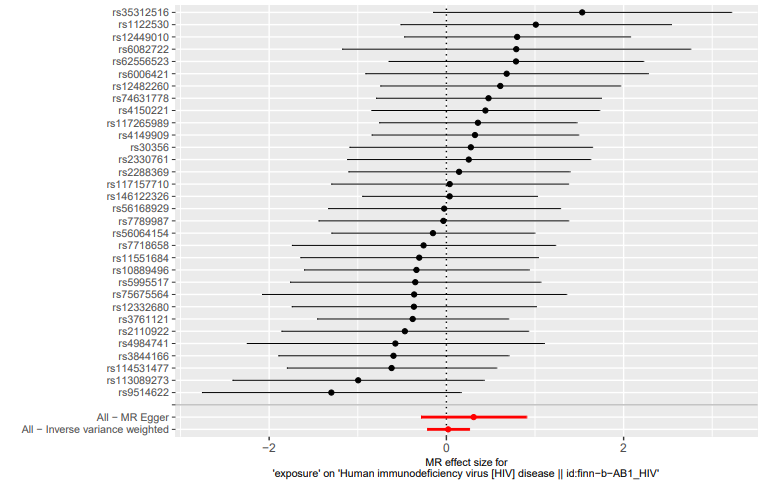


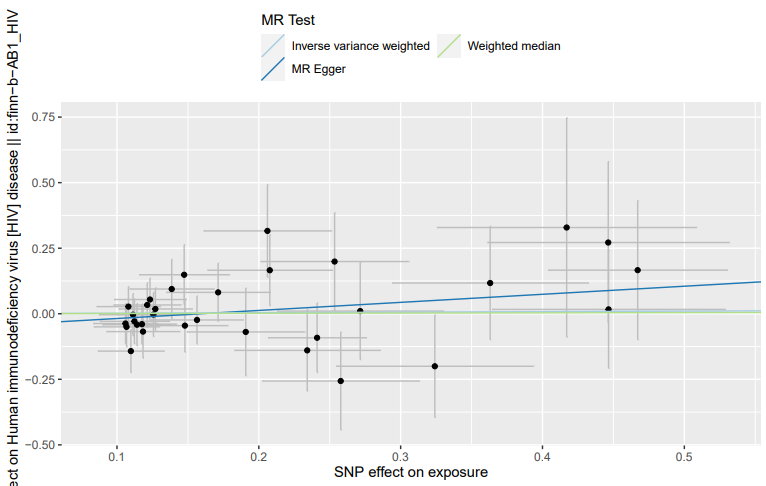


**Figure S22.** The leave-one-out plot, forest plot, and scatter plot for the association of measles infection and GBM in the reverse analysis. Data from FINN.


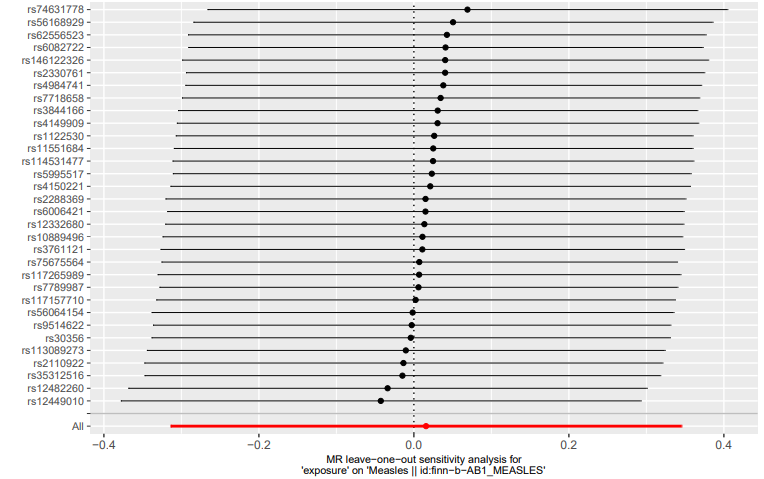


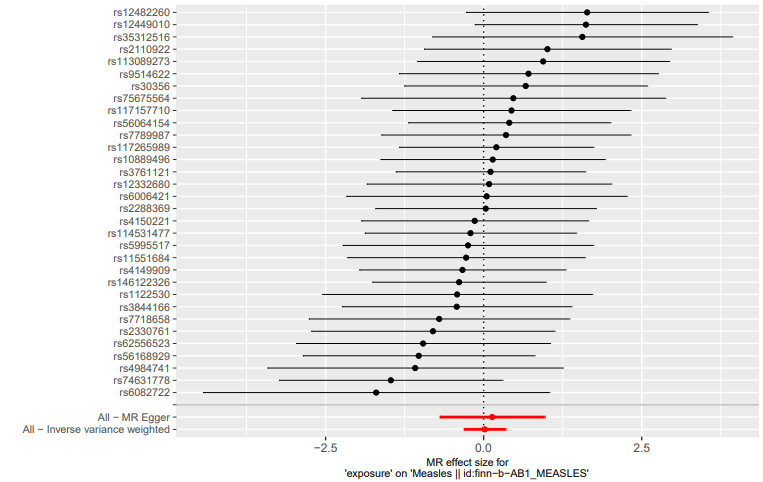


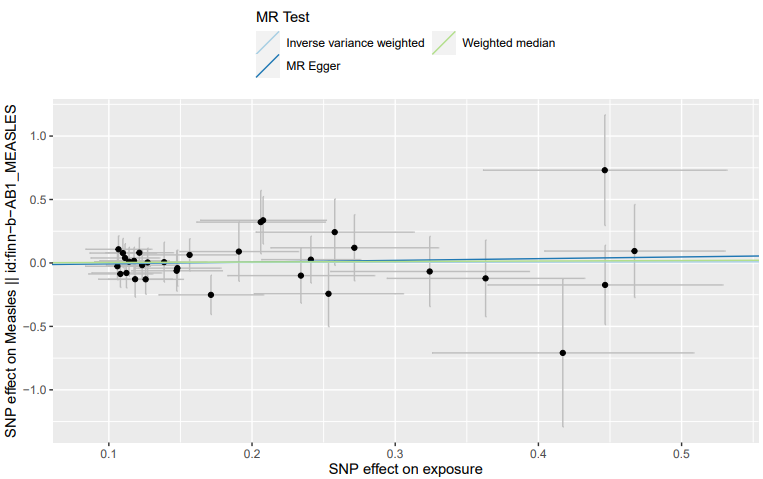


**Figure S23.** The leave-one-out plot, forest plot, and scatter plot for the association of Poliovirus infection and GBM in the reverse analysis. Data from FINN.


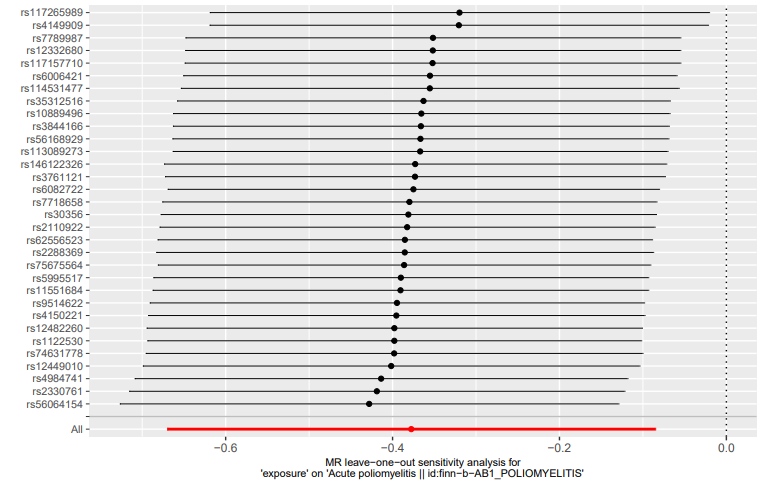


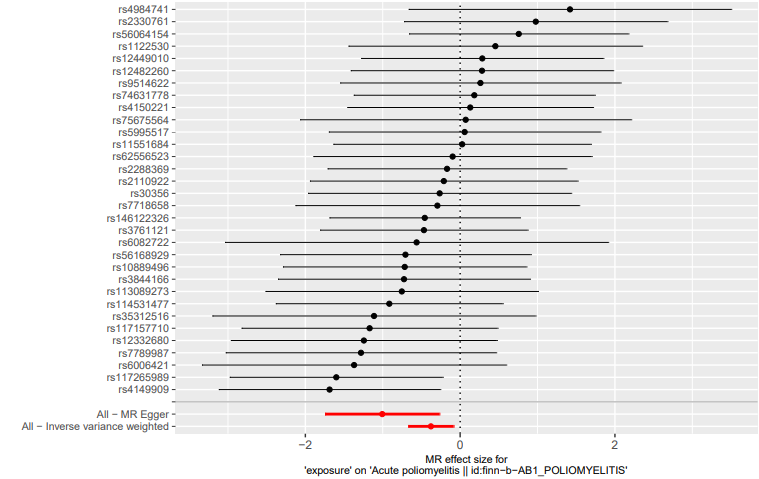


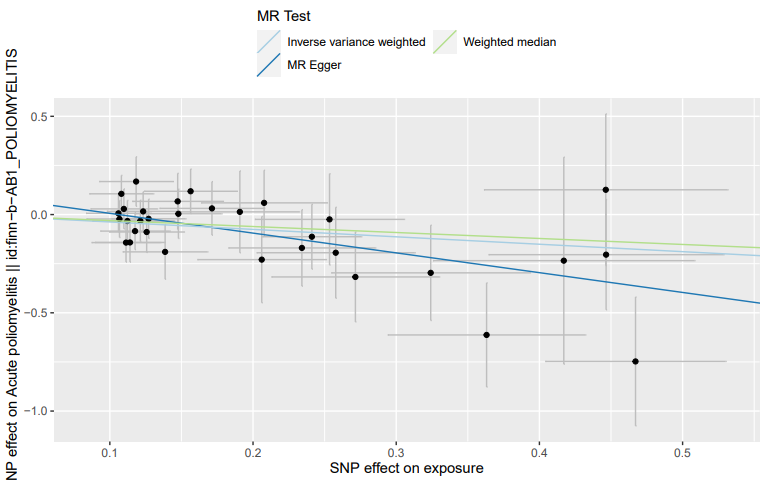


**Figure S24.** The leave-one-out plot, forest plot, and scatter plot for the association of Rubella infection and GBM in the reverse analysis. Data from FINN.


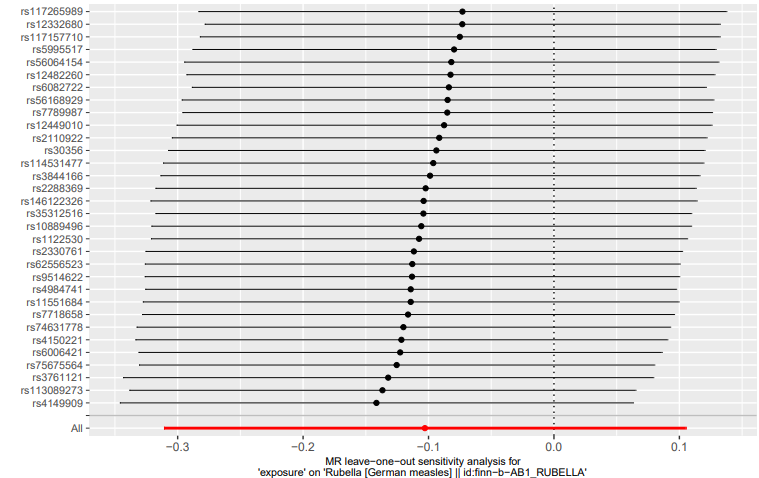


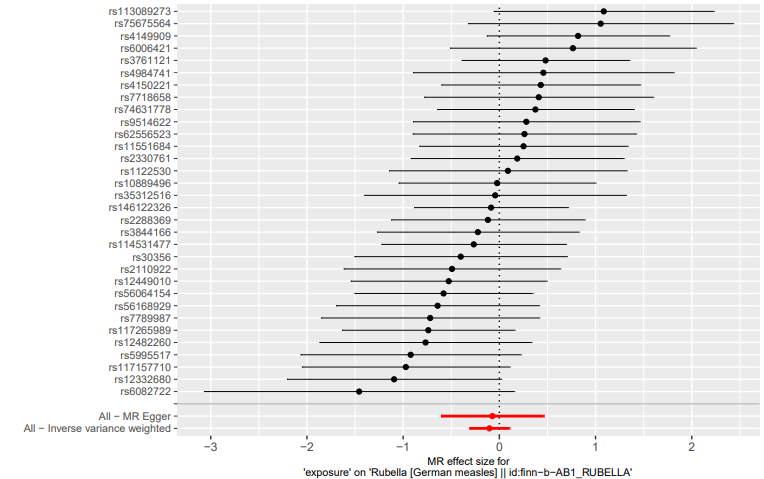


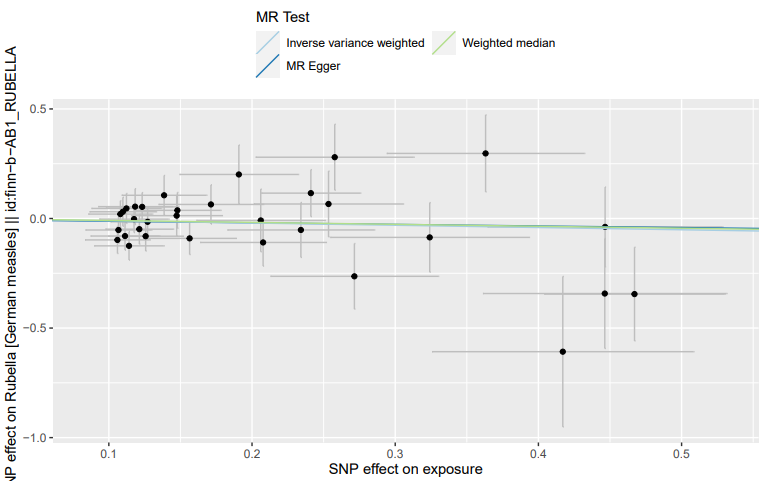


**Figure S25.** The leave-one-out plot, forest plot, and scatter plot for the association of Herpes zoster and all-glioma in the reverse analysis. Date from FINN.

**
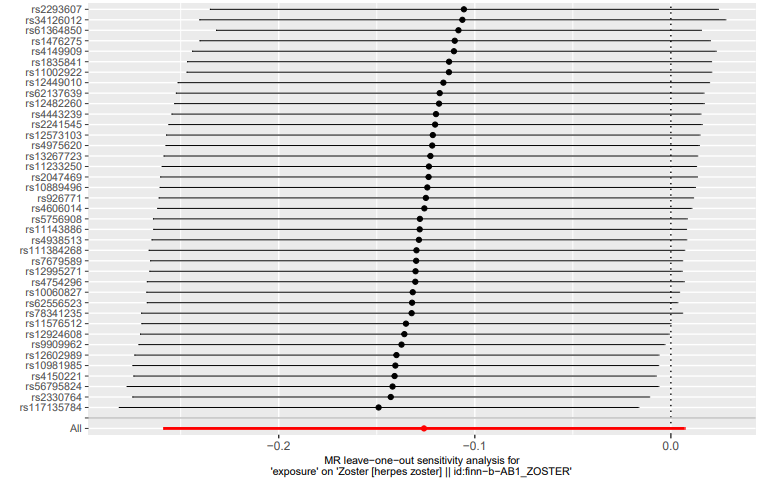
**

**
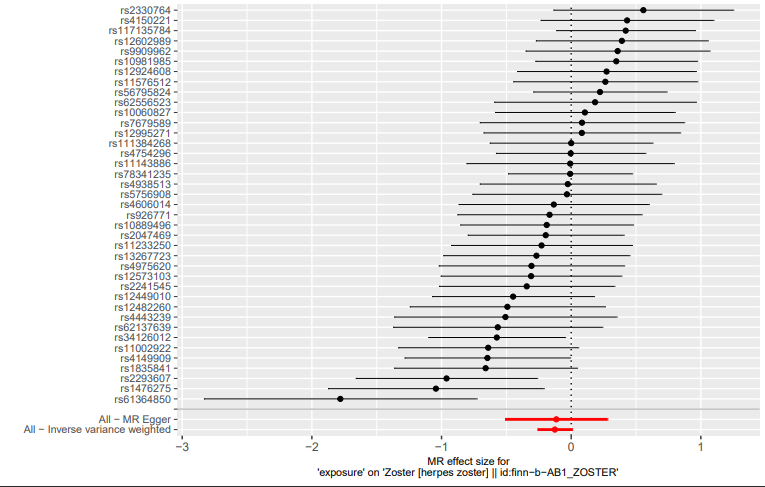
**

**
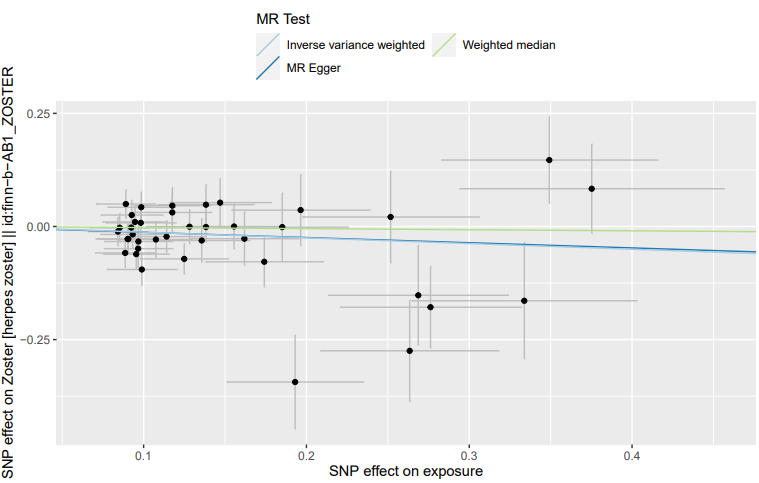
**

**Figure S26.** The leave-one-out plot, forest plot, and scatter plot for the association of mumps virus infection and all-glioma in the reverse analysis. Date from FINN.

**
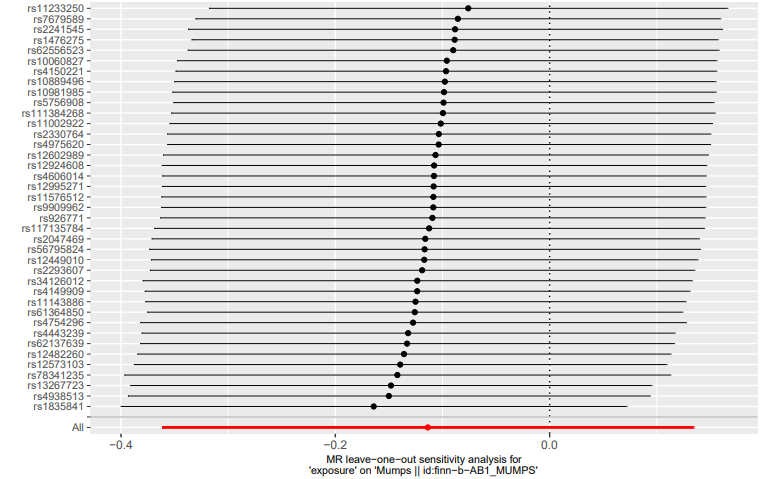
**

**
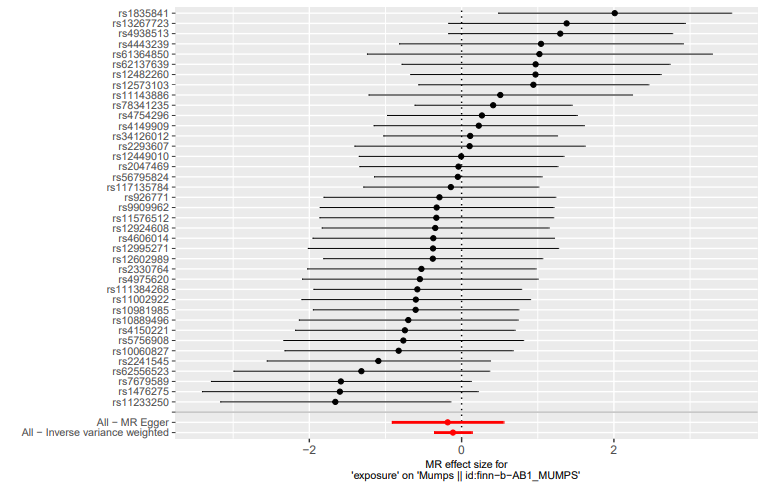
**

**
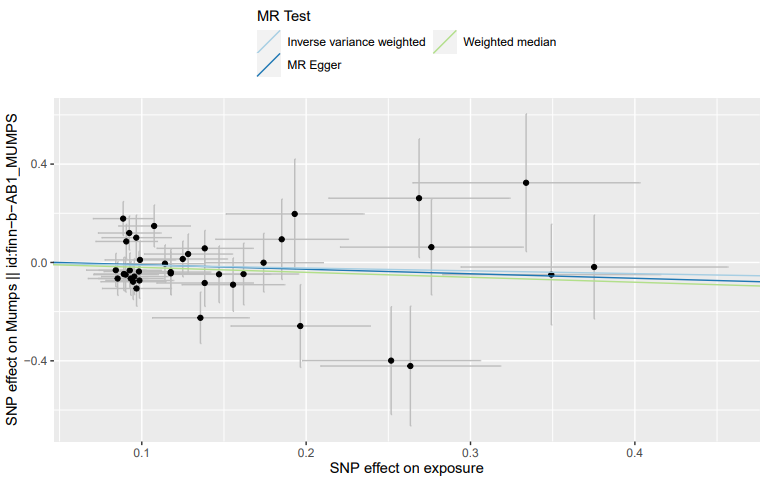
**

**Figure S27.** The leave-one-out plot, forest plot, and scatter plot for the association of HSV infection and all-glioma in the reverse analysis. Data from FINN.

**
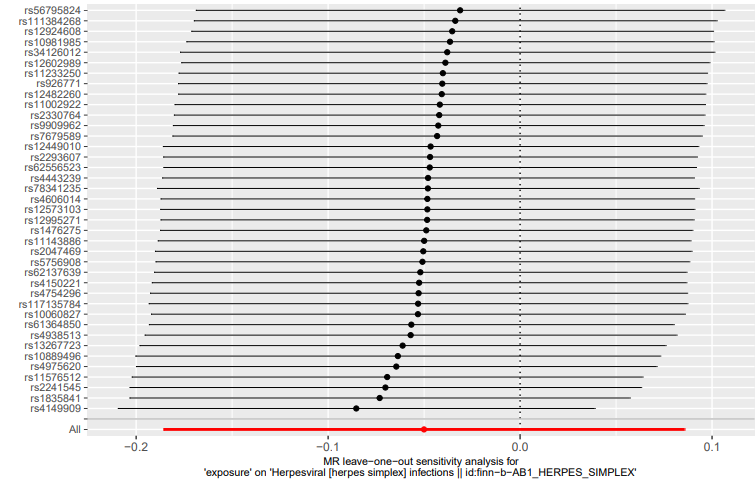
**

**
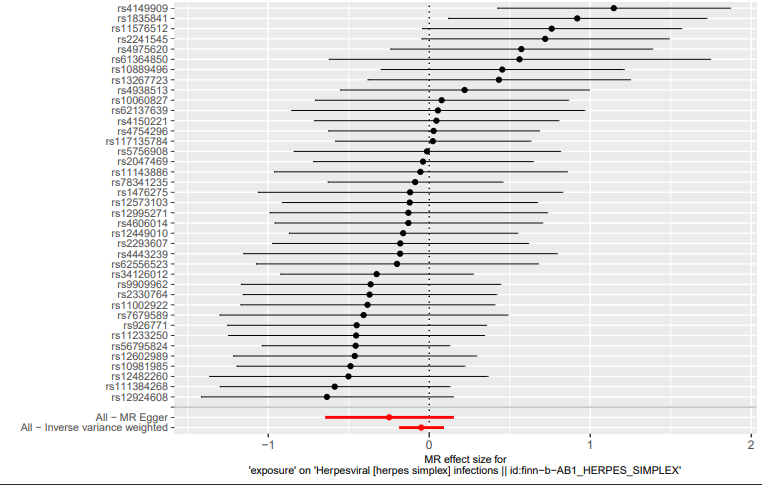
**

**
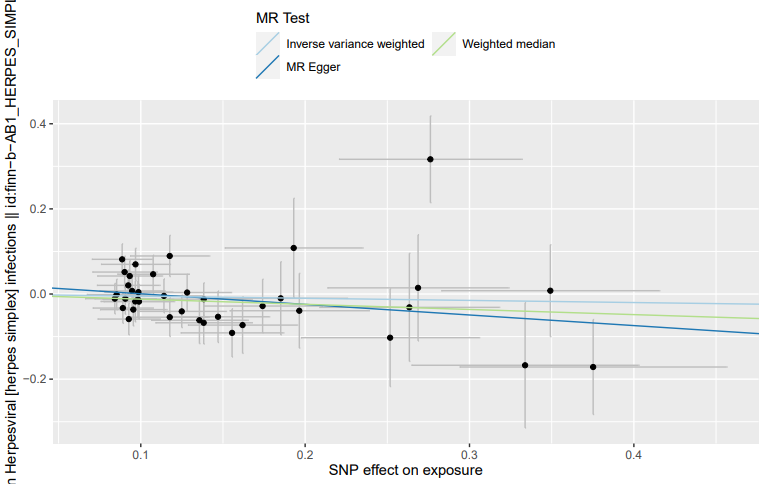
**

**Figure S28.** The leave-one-out plot, forest plot, and scatter plot for the association of HPV infection and all-glioma in the reverse analysis. Data from SUHRE.


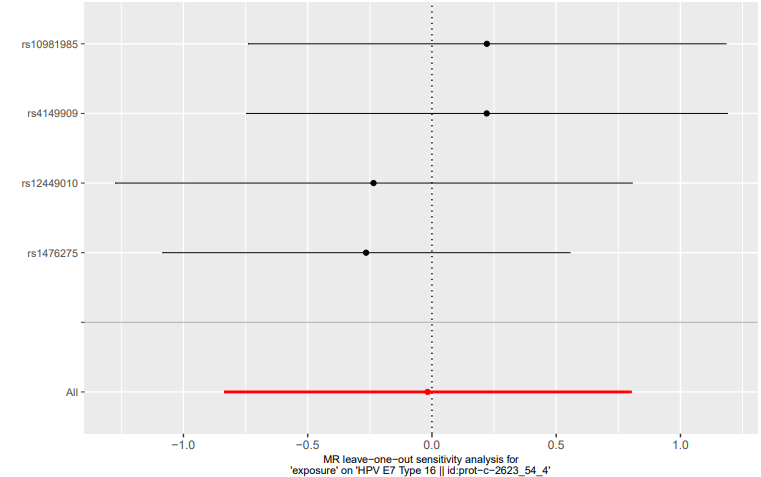


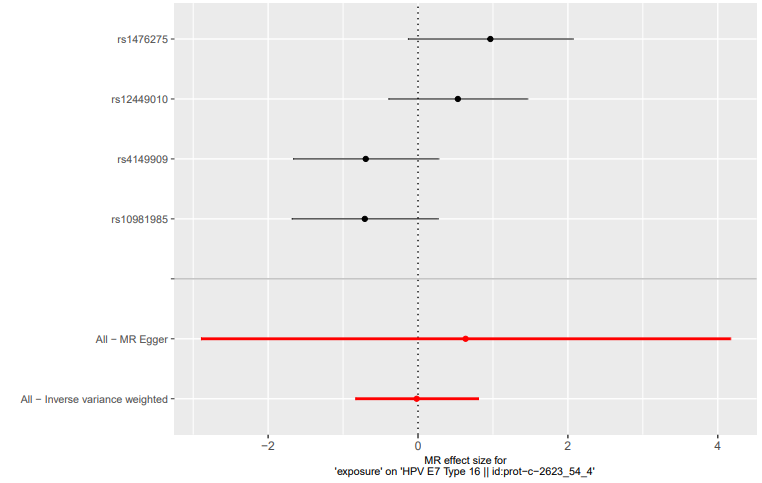


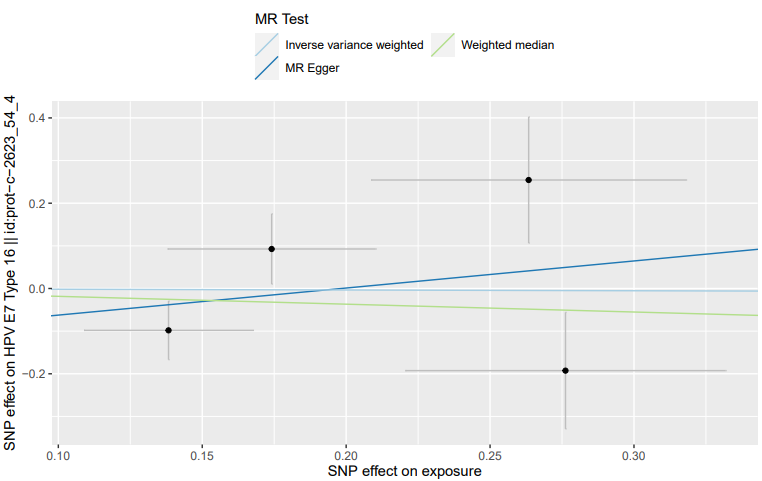


**Figure S29.** The leave-one-out plot, forest plot, and scatter plot for the association of EBV infection and all-glioma in the reverse analysis. Data from FINN.

**
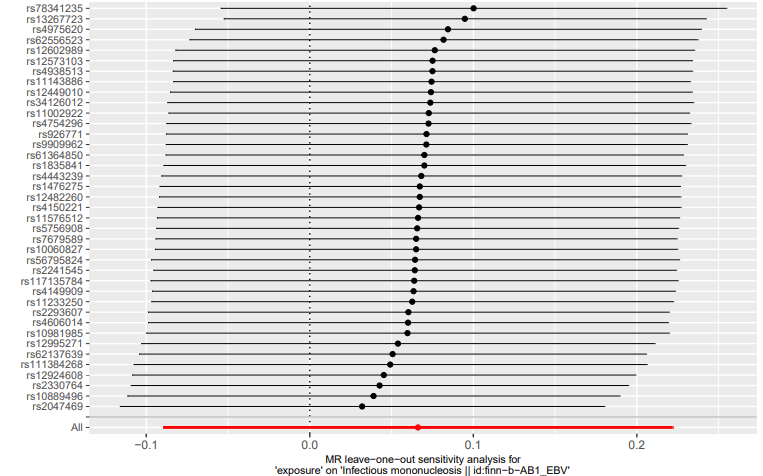
**

**
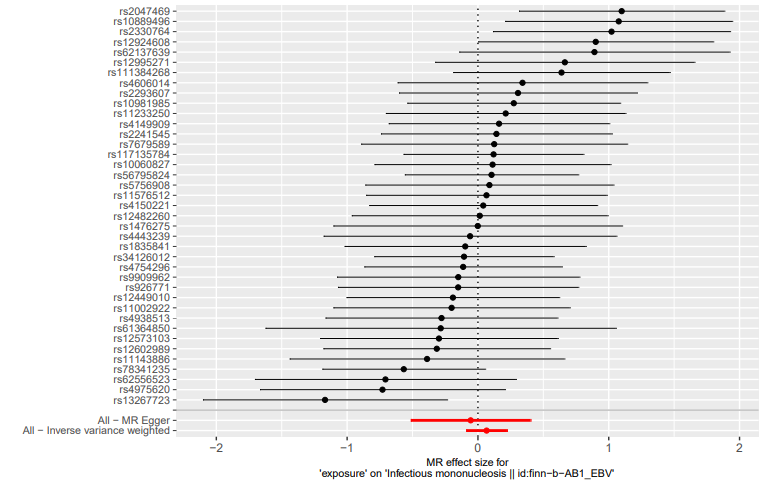
**

**
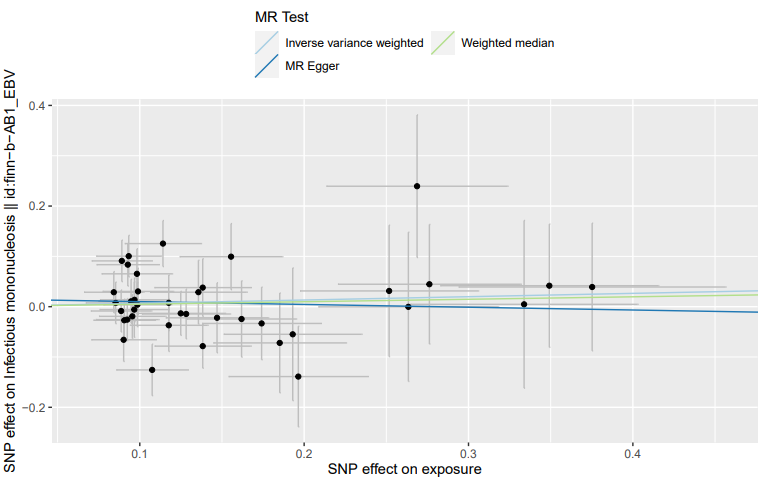
**

**Figure S30.** The leave-one-out plot, forest plot, and scatter plot for the association of COVID-19 infection and all-glioma in the reverse analysis. Data from COVID-19 HGI.

**
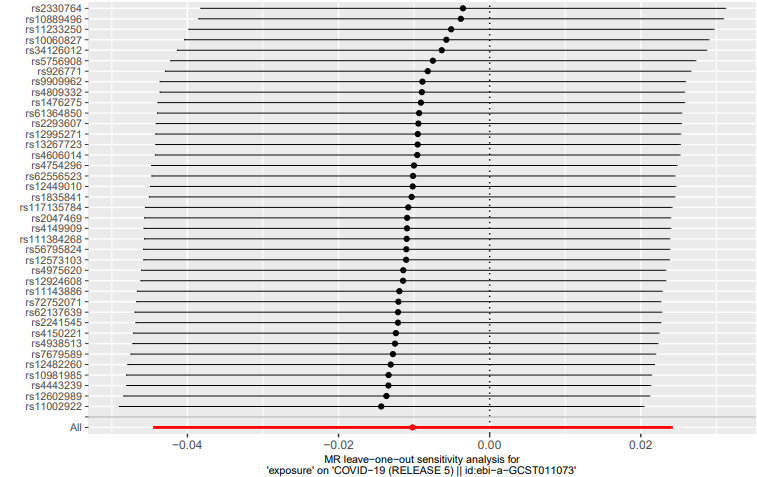
**

**
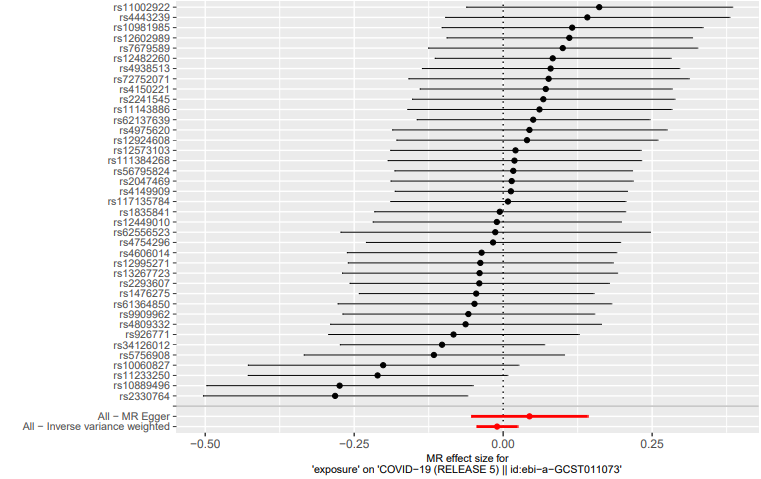
**

**
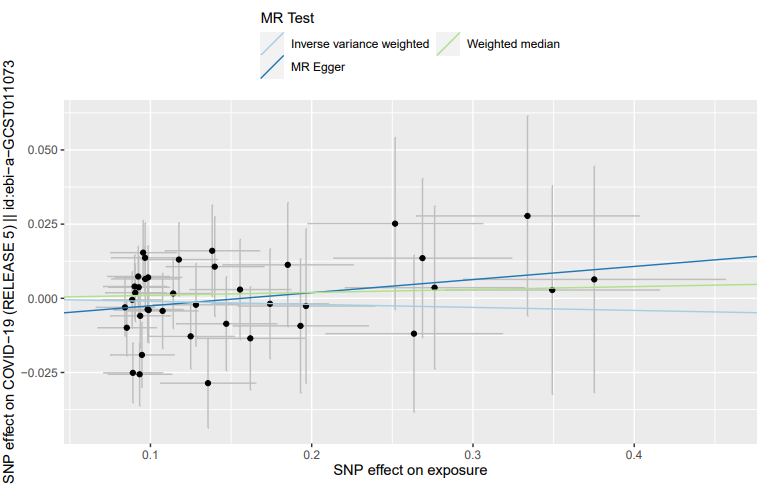
**

**Figure S31.** The leave-one-out plot, forest plot, and scatter plot for the association of HCMV infection and all-glioma in the reverse analysis. Data from FINN.


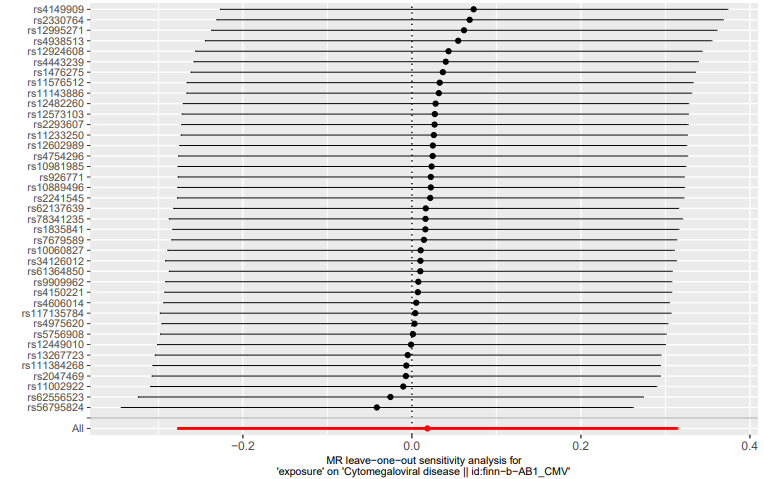


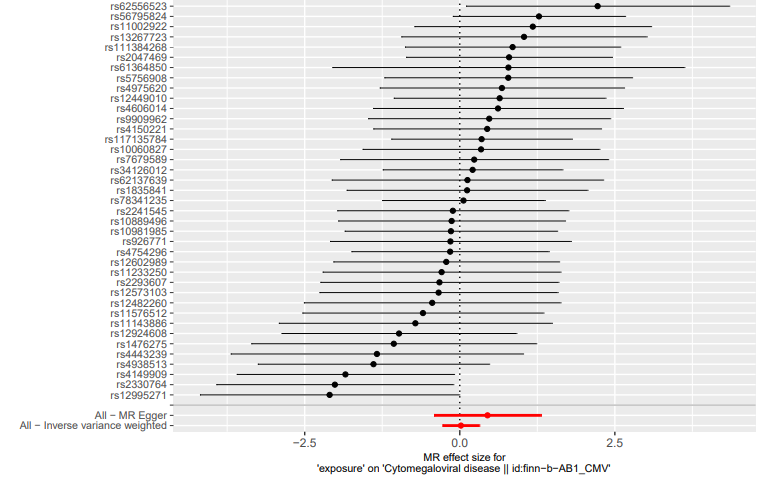


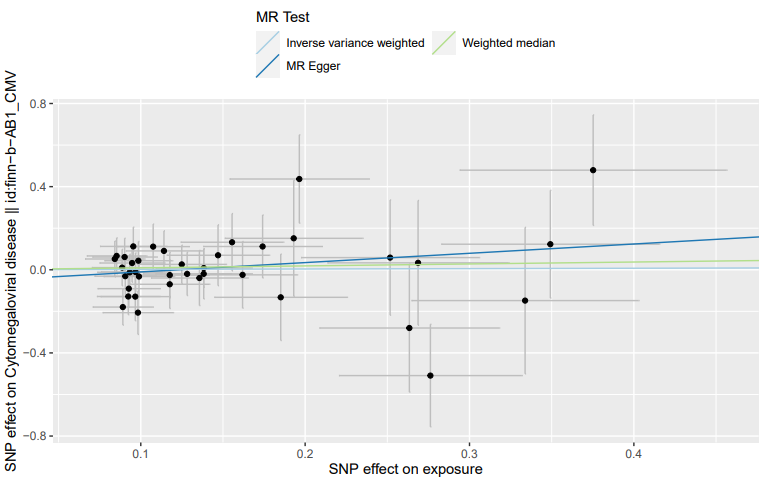


**Figure S32.** The leave-one-out plot, forest plot, and scatter plot for the association of Hepatitis infection and all-glioma in the reverse analysis. Data from FINN.


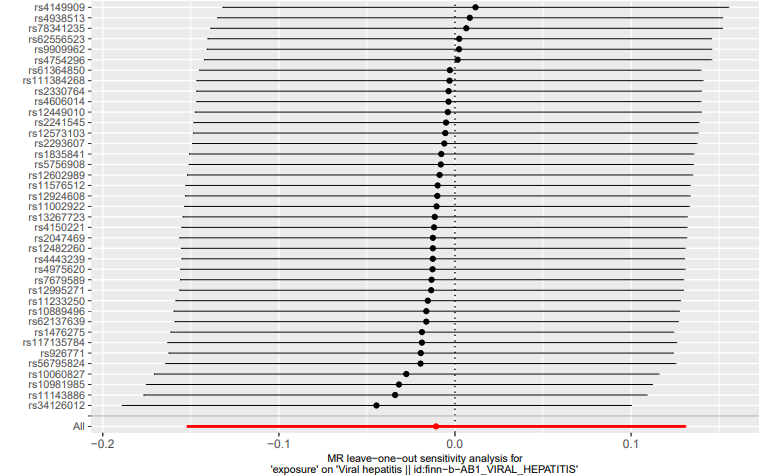


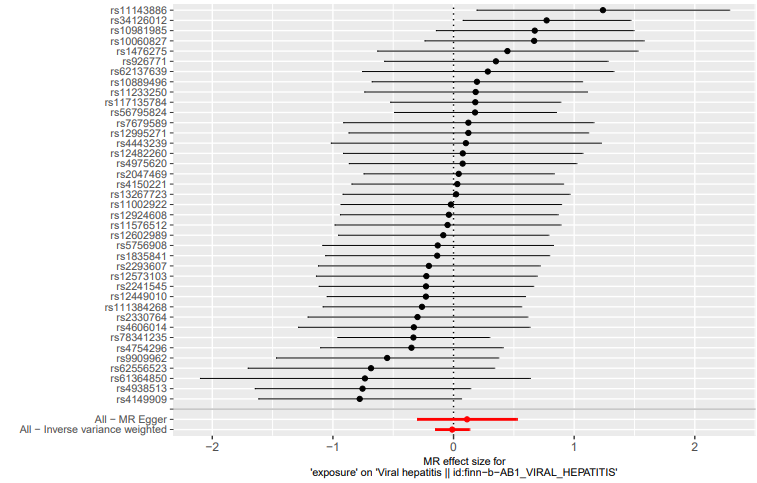


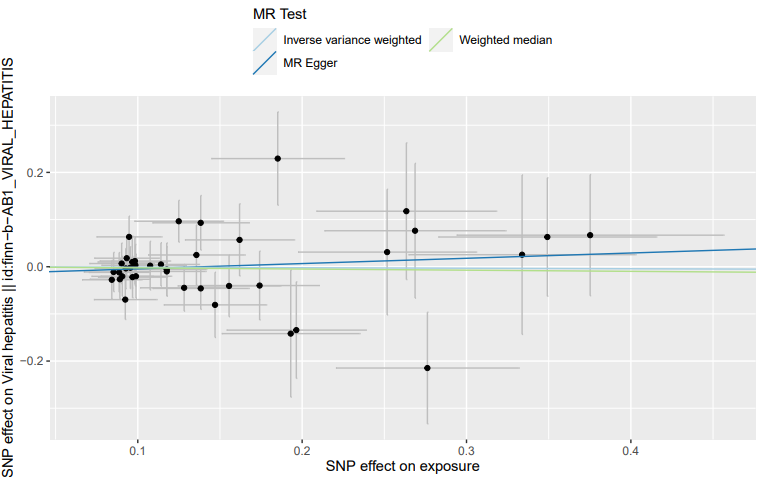


**Figure S33.** The leave-one-out plot, forest plot, and scatter plot for the association of HIV infection and all-glioma in the reverse analysis. Data from FINN.


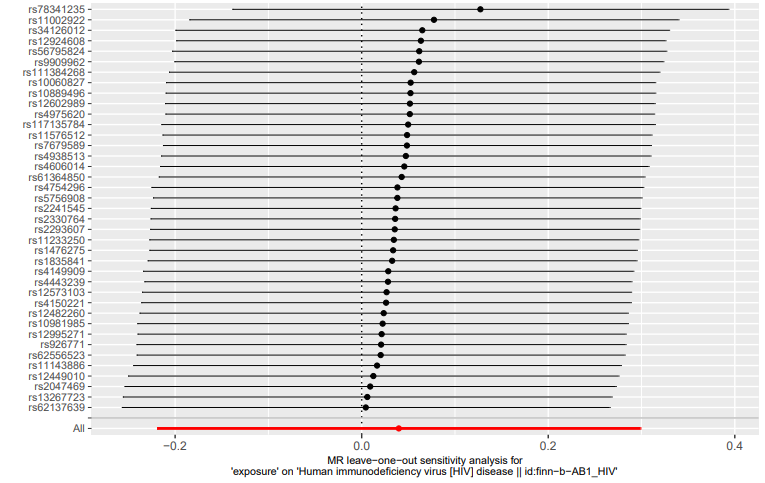


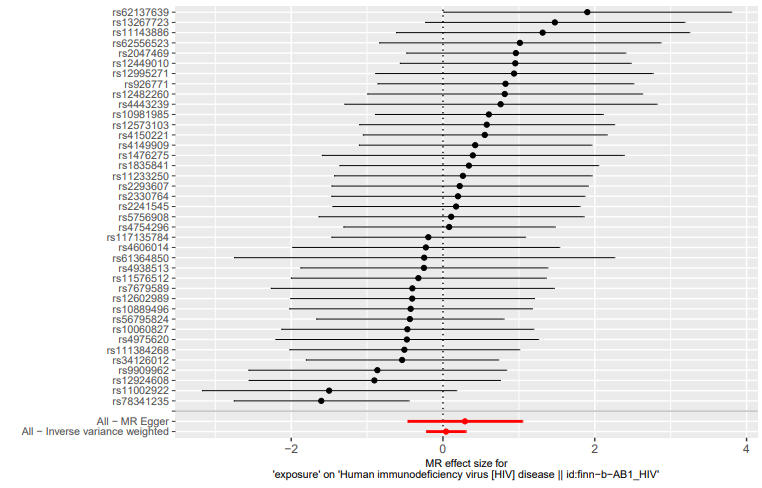


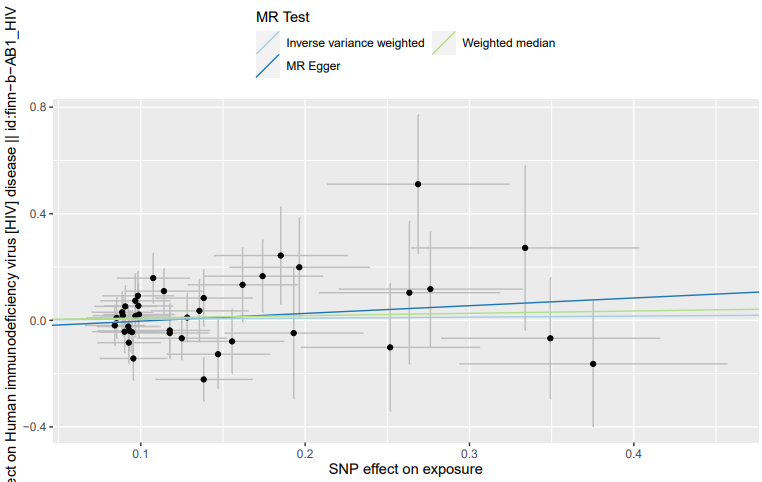


**Figure S34.** The leave-one-out plot, forest plot, and scatter plot for the association of measles infection and all-glioma in the reverse analysis. Data from FINN.


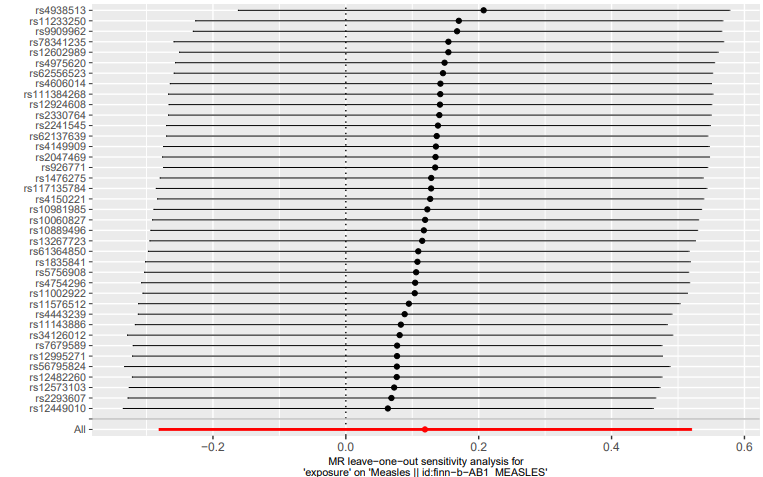


**Figure S35.** The leave-one-out plot, forest plot, and scatter plot for the association of Poliovirus infection and all-glioma in the reverse analysis. Data from FINN.

**Figure S36.** The leave-one-out plot, forest plot, and scatter plot for the association of Rubella infection and all-glioma in the reverse analysis. Data from FINN.
