# Supplementary material for: Association between viral infections and glioma risk: a two-sample bidirectional Mendelian randomization analysis
Source: BMC Med. 2023 Dec 5;21:487. doi: 10.1186/s12916-023-03142-9 (PMC10698979; doi:10.1186/s12916-023-03142-9)
Supplement: Supplementary file 9 — Additional file 9. Source code. [file 12916_2023_3142_MOESM9_ESM.docx]

**Additional file 9 Source code**

library(TwoSampleMR)

library(data.table)

cyto <- extract_instruments(outcomes = 'finn-b-AB1_ZOSTER',p1= 5e-06,clump = TRUE,r2= 0.001,kb=10000,access_token = NULL)

setwd("C:\\Users\\LEGION\\Desktop\\virus-GBM\\glioma_MR")

Glioma <- fread('GBM.txt',header = T)

Glioma$phenotype <-'GBM'

Glioma_out <- format_data(dat=Glioma,type = "outcome",

snps = cyto$SNP,

header = TRUE,

phenotype_col = "phenotype",

snp_col = "SNP",

beta_col = "BETA",

se_col = "SE",

effect_allele_col = "A1",

other_allele_col = "A2",

pval_col = "P",

chr_col = "CHROMOSOME",

pos_col = "POSITION")

mydata <- harmonise_data(exposure_dat=cyto,outcome_dat=Glioma_out,action= 2)

result <- mr(mydata, method_list=c("mr_egger_regression", "mr_ivw","mr_weighted_median"))

generate_odds_ratios(result)

MR<-generate_odds_ratios(result)

het <- mr_heterogeneity(mydata) #

res_single <- mr_singlesnp(mydata)

pleio <- mr_pleiotropy_test(mydata)

single <- mr_leaveoneout(mydata)###leaveoneout analysis mydata,NbDistribution = 3000

presso <- run_mr_presso(mydata)

setwd("C:\\Users\\LEGION\\Desktop\\virus-GBM\\results\\HIV\\FINN\\GBM")

pdf('Result.pdf',width = 8,height = 5.1)

mr_scatter_plot(mr_results = mr(mydata,method_list = c("mr_egger_regression", "mr_ivw","mr_weighted_median")),mydata)

mr_forest_plot(res_single)

mr_funnel_plot(res_single)

mr_leaveoneout_plot(single)

dev.off()

write.table(het,file="het.csv",sep=",",quote=F,row.names=F)

write.table(pleio,file="pleio.csv",sep=",",quote=F,row.names=F)

write.table(MR,file="result.csv",sep=",",quote=F,row.names=F)

write.table(Glioma_out,file="Outcome.csv",sep=",",quote=F,row.names=F)

write.table(presso,file="presso.csv",sep=",",quote=F,row.names=F)

write.table(cyto,file="exposure.csv",sep=",",quote=F,row.names=F)

write.table(mydata,file="harmonise.csv",sep=",",quote=F,row.names=F)

##############################

library(data.table)

library(TwoSampleMR)

setwd("C:\\Users\\LEGION\\Desktop\\virus-GBM\\23andme")

a <- fread('mumps.txt',header = T)

b <-subset(a,P.value<5e-6)

write.csv(b, file="exposure(measles).csv")

cyto<-read_exposure_data(filename ="exposure(singles).csv",sep = ",",snp_col = "SNP",beta_col = "beta",se_col = "se",effect_allele_col = "effect_allele",other_allele_col = "other_allele",eaf_col = "eaf",pval_col = "p",clump = TRUE)

setwd("C:\\Users\\LEGION\\Desktop\\virus-GBM\\glioma_MR")

Glioma <- fread('non_GBM.txt',header = T)

Glioma$phenotype <-'GBM'

Glioma_out <- format_data(dat=Glioma,type = "outcome",

snps = cyto$SNP,

header = TRUE,

phenotype_col = "phenotype",

snp_col = "SNP",

beta_col = "BETA",

se_col = "SE",

effect_allele_col = "A1",

other_allele_col = "A2",

pval_col = "P",

chr_col = "CHROMOSOME",

pos_col = "POSITION")

mydata <- harmonise_data(exposure_dat=cyto,outcome_dat=Glioma_out,action= 2)

result <- mr(mydata, method_list=c("mr_egger_regression", "mr_ivw","mr_weighted_median"))

generate_odds_ratios(result)

MR<-generate_odds_ratios(result)

het <- mr_heterogeneity(mydata)

res_single <- mr_singlesnp(mydata)

pleio <- mr_pleiotropy_test(mydata)

single <- mr_leaveoneout(mydata)###leaveoneout analysis ,NbDistribution = 3000

presso <- run_mr_presso(mydata)

setwd("C:\\Users\\LEGION\\Desktop\\virus-GBM\\results\\mumps\\FINN\\glioma")

pdf('Result.pdf',width = 8,height = 5.1)

mr_scatter_plot(mr_results = mr(mydata,method_list = c("mr_egger_regression", "mr_ivw","mr_weighted_median")),mydata)

mr_forest_plot(res_single)

mr_funnel_plot(res_single)

mr_leaveoneout_plot(single)

dev.off()

write.table(het,file="het.csv",sep=",",quote=F,row.names=F)

write.table(pleio,file="pleio.csv",sep=",",quote=F,row.names=F)

write.table(MR,file="result.csv",sep=",",quote=F,row.names=F)

write.table(Glioma_out,file="Outcome.csv",sep=",",quote=F,row.names=F)

write.table(presso,file="presso.csv",sep=",",quote=F,row.names=F)

write.table(cyto,file="exposure.csv",sep=",",quote=F,row.names=F)

write.table(mydata,file="harmonise.csv",sep=",",quote=F,row.names=F)

exposure <- extract_instruments(outcomes = 'ieu-a-1013')

cyto <- extract_instruments(outcomes = 'ieu-b-5070',p1= 5e-06,clump = TRUE,r2= 0.001,kb=10000,access_token = NULL)

Glioma_out <- extract_outcome_data(snps=cyto$SNP,outcomes = 'ebi-a-GCST011081')

#############meta

install.packages("openxlsx")

library(openxlsx)

library("meta")

setwd("C:\\Users\\LEGION\\Desktop\\virus-GBM")

data1=read.table("data1.txt", header = TRUE,sep="\t")

data1=read.xlsx("data1.xlsx")

bin.metagen<- data1

bin.metagen$OR <- log(bin.metagen$OR)

bin.metagen$LCI <- log(bin.metagen$LCI)

bin.metagen$UCI <- log(bin.metagen$UCI)

bin.metagen$seTE <- (bin.metagen$UCI - bin.metagen$LCI)/3.92

fit<-metagen(OR,

seTE,

studlab = Author,

method.tau = "SJ",

sm = "OR",

data = bin.metagen)

forest(fit)

fit

############Reverse MR

library(TwoSampleMR)

library(data.table)

setwd("C:\\Users\\LEGION\\Desktop\\virus-GBM\\glioma_MR")

a <- fread('non_GBM.txt',header = T)

a$phenotype<- 'Glioma'

a <-subset(a,P<5e-06)

cyto<-clump_data(a,clump_r2=0.001,clump_kb=10000)

cyto <- format_data(cyto,

type='exposure',

snp_col = "SNP",

beta_col = "BETA",

se_col = "SE",

effect_allele_col ="A1",

other_allele_col = "A2",

pval_col = "P")

id_outcome<- "finn-b-AB1_ZOSTER"

outcome_dat <- extract_outcome_data(snps=cyto$SNP,

id_outcome,

proxies = FALSE,

maf_threshold = 0.01,

access_token = NULL,

)

mydata <- harmonise_data(exposure_dat=cyto,outcome_dat=outcome_dat)

result <- mr(mydata, method_list=c("mr_egger_regression", "mr_ivw","mr_weighted_median"))

generate_odds_ratios(result)

MR<-generate_odds_ratios(result)

het <- mr_heterogeneity(mydata) ###

res_single <- mr_singlesnp(mydata)

pleio <- mr_pleiotropy_test(mydata)###

single <- mr_leaveoneout(mydata)###leaveoneout analysis ,NbDistribution = 3000

presso <- run_mr_presso(mydata,NbDistribution = 3000)

setwd("C:\\Users\\LEGION\\Desktop\\virus-GBM\\results\\VZV\\VZV")

pdf('Result.pdf',width = 8,height = 5.1)

mr_scatter_plot(mr_results = mr(mydata,method_list = c("mr_egger_regression", "mr_ivw","mr_weighted_median")),mydata)

mr_forest_plot(res_single)

mr_funnel_plot(res_single)

mr_leaveoneout_plot(single)

dev.off()

write.table(het,file="het.csv",sep=",",quote=F,row.names=F)

write.table(pleio,file="pleio.csv",sep=",",quote=F,row.names=F)

write.table(MR,file="result.csv",sep=",",quote=F,row.names=F)

write.table(outcome_dat ,file="Outcome.csv",sep=",",quote=F,row.names=F)

write.table(cyto,file="exposure.csv",sep=",",quote=F,row.names=F)

write.table(presso,file="presso.csv",sep=",",quote=F,row.names=F)
