# Supplementary figures and images for: Association between viral infections and glioma risk: a two-sample bidirectional Mendelian randomization analysis
Source: BMC Med. 2023 Dec 5;21:487. doi: 10.1186/s12916-023-03142-9 (PMC10698979; doi:10.1186/s12916-023-03142-9)

**Additional file 10 Retouching certificate.**


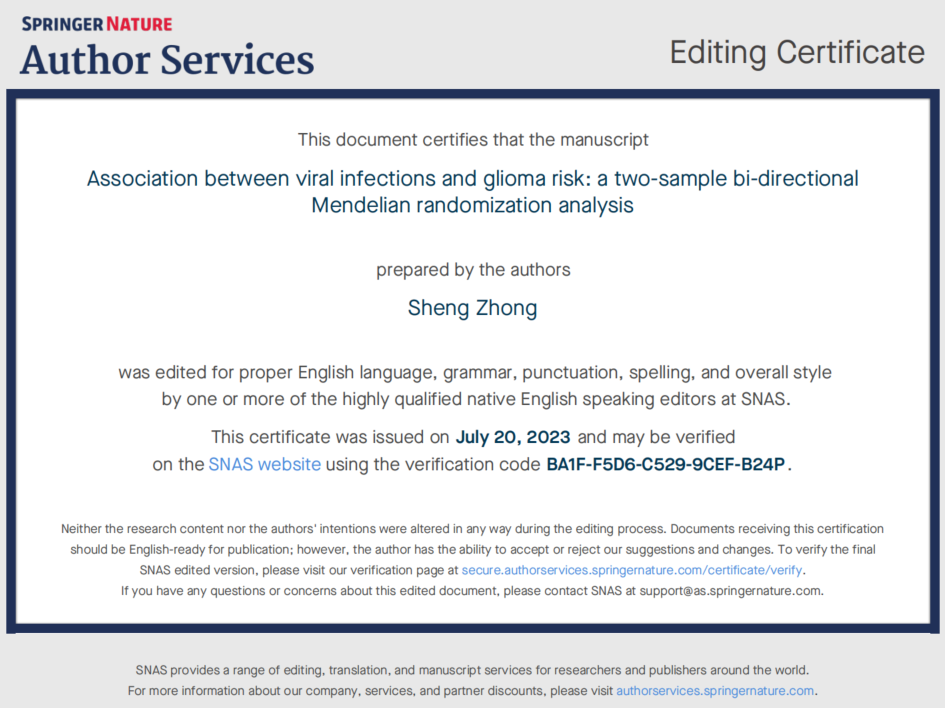

Supplement: Supplementary file 10 — Additional file 10. Retouching certificate. [file 12916_2023_3142_MOESM10_ESM.docx]
